# An annulus fibrosus anchored nanovector reprograms osteopontin driven macrophages in preclinical disc degeneration models

**DOI:** 10.1038/s41467-026-75720-3

**Published:** 2026-07-21

**Authors:** Zilong Li, Yucheng Xue, Zhenyu Li, Miaojie Fang, Yiwen Xu, Kelei Wang, Hong Liu, Zhenzhong Chen, Jianbin Xu, Ning Zhang

**Affiliations:** 1 Department of Orthopedic Surgery, The Second Affiliated Hospital, Zhejiang University School of Medicine, Hangzhou, China; 2 Orthopedics Research Institute of Zhejiang University, Hangzhou, China; 3 Key Laboratory of Motor System Disease Research and Precision Therapy of Zhejiang Province, Hangzhou, China; 4 Zhejiang Provincial Clinical Medical Research Center for Motor System Diseases, Hangzhou, China; 5 International Chinese Musculoskeletal Research Society, Hangzhou, China; 6 University of California, Santa Barbara, California, CA USA; 7 Department of Orthopedics,The Fourth Affiliated Hospital, Zhejiang University School of Medicine, Yiwu, China; 8 Department of Orthopedics, Jiangnan Hospital, Zhejiang Chinese Medical University, Hangzhou, China; 9 Department of Orthopedics, Lishui Municipal Central Hospital, Lishui, China

**Keywords:** Preclinical research, Inflammation, Bone

## Abstract

Intervertebral disc degeneration is a major cause of low back pain and is associated with inflammatory macrophages that promote annulus fibrosus degeneration via osteopontin signaling. Here we report an annulus fibrosus anchored nanovector carrying small interfering RNA targeting osteopontin. The nanovector first binds annulus fibrosus cells via a targeting peptide and is subsequently activated by reactive oxygen species in inflammatory microenvironments to release macrophage targeted hybrid nanovesicles. This sequential design enables local retention in disc tissue and subsequent delivery to osteopontin expressing macrophages. Using human single cell transcriptomic analysis, in vitro macrophage and annulus fibrosus cell coculture assays, mouse macrophage depletion and infiltration models, and rat disc injury models, we show that this strategy reduces pro inflammatory macrophage polarization, preserves extracellular matrix integrity, and improves disc structure. These findings support an inflammation responsive immunomodulatory approach for disc repair in preclinical models.

## Abstract (web summary)

This study develops a smart nanoparticle that anchors to intervertebral disc tissue and releases therapy to modulate inflammatory macrophages, reducing disc degeneration and promoting tissue repair in preclinical models.

## Introduction

Intervertebral disc degeneration (IVDD) is a major cause of chronic low back pain and disability, posing a substantial socio-economic burden worldwide^1–5^. The difficulty in treating IVDD lies in its unique biological barriers^6–9^. As the largest avascular tissue in the human body, the intervertebral disc (IVD) has limited nutrient transport and immune accessibility^10,11^. The progressive degradation of the extracellular matrix (ECM) and the formation of a chronic inflammatory microenvironment make it difficult to reverse matrix destruction or regulate inflammation^12,13^. Specifically, the annulus fibrosus (AF), with its dense and highly organized fibrous structure, resists passive molecular penetration and is prone to structural breakdown due to degeneration^14,15^, thereby promoting ECM degradation and the formation of an inflammatory microenvironment. Moreover, the AF has a poor intrinsic regenerative capacity, further complicating attempts to restore disc homeostasis^16^. Several studies have shown that microstructural damage to the AF is not only one of the early features of IVDD but also exacerbates nucleus pulposus (NP) herniation, induces local immune activation, and amplifies inflammatory responses, thereby accelerating the degenerative process^17–20^. Damaged AF breaks the immune privilege of the NP, exposing antigenic components and triggering infiltration of T cells and macrophages along with a persistent inflammatory cascade^21,22^. In addition, AF cells undergo phenotypic changes and decreased activity during degeneration, with impaired responsiveness to mechanical stimuli, leading to disorganized fiber arrangement, rupture, and further initiating a vicious cycle^23,24^. Therefore, developing innovative therapeutic strategies that can overcome the IVD’s unique biological barriers and specifically repair the damaged AF is an important strategy for achieving targeted intervention and stable modulation of the inflammatory microenvironment, as well as matrix regeneration and repair, to address the current clinical challenges of IVDD.

Recent studies suggest that immune dysregulation in the disc’s pathological microenvironment is a key driver of AF destruction^25,26^. Among these, macrophages play a critical role in the pathological process. Due to the functional loss mediated by the imbalance of macrophage inflammatory states, features such as angiogenesis and matrix degradation are triggered^27,28^. In this study, our single-cell analysis confirmed significant macrophage accumulation in the degenerated IVDs and that macrophages exhibit substantial heterogeneity in the immune environment of degenerated discs. Through re-clustering analysis of macrophages, we identified two major subsets: pro-inflammatory and anti-inflammatory macrophages, with pro-inflammatory macrophages predominating in degenerated tissues. Further functional analysis revealed that pro-inflammatory macrophages are enriched in various immune-activation-related pathways, including Toll-like receptor signaling, PI3K-Akt signaling, and ECM-receptor interactions, all of which are closely related to the pathological process of disc degeneration. Previous studies have also confirmed that macrophages in degenerated discs predominantly polarize into the pro-inflammatory M1 phenotype, secreting cytokines such as TNF-α and IL-1β, activating matrix metalloproteinases (MMPs), and inducing apoptosis in NP cells^29–31^. In contrast, the M2 macrophage phenotype, associated with tissue repair, anti-inflammatory cytokine secretion, and ECM remodeling, is less frequently observed in degenerated discs^32–34^. Our findings further confirm that secreted phosphoprotein 1 (*SPP1*) is highly expressed in pro-inflammatory macrophages. The expression of *SPP1* is almost exclusively enriched in these macrophage subsets and is closely associated with tissue remodeling and chronic inflammation. Knockdown of *SPP1* significantly reduces the proportion of M1 macrophages, alleviates the inflammatory response in disc degeneration, and may potentially reverse the degenerative process by modulating macrophage function. Therefore, the imbalance of macrophage states serves as a key driver of inflammation and further degeneration, and targeting *SPP1*-mediated macrophage function may represent an important strategy for reversing the progression of IVDD. Moreover, targeted modulation of macrophage function in the AF has emerged as an approach to regulate the local immune microenvironment and halt IVDD progression.

However, the anatomical structure and microenvironmental characteristics of the AF pose major challenges to targeting *SPP1*-mediated macrophage functions^35,36^. The very low tissue permeability within the AF greatly limits the long-term retention and diffusion of therapeutic agents. In addition, the ECM of the AF lacks sufficient cell adhesion sites or receptors, making it difficult for most drugs or delivery systems to achieve anchored binding^37–40^. In particular, macrophage infiltration exhibits stress-triggered and time-dependent—typically occurring only after mechanical injury, matrix rupture, or disruption of the immune barrier—rendering static delivery strategies ineffective for dynamic and precise regulation^41,42^. To address these challenges, gene therapy based on nucleic acid drugs targeting *SPP1* has emerged as a powerful tool for modulating macrophage function, owing to its high specificity and programmability. However, siRNA molecules are unstable in the complex disc microenvironment, lack protective systems, and cannot actively target and penetrate the AF lesion areas, which significantly limits their practical intervention effects in vivo^43^. Studies have demonstrated that bacterial outer membrane vesicles (OMVs), as natural delivery systems enriched with pathogen-associated molecular patterns, can be specifically recognized by macrophage surface receptors^44,45^, making them promising platforms for siRNA-based interventions. Nevertheless, in highly immune-privileged tissues such as the IVD, OMVs may trigger additional inflammatory responses. Therefore, developing OMV-based nucleic acid delivery systems tailored for IVDD therapy holds great promise for regulating gene expression (e.g., SPP1) and reprogramming specific macrophage subpopulations within the complex pathological microenvironment of the IVD.

In this study, we identified the critical role of *SPP1* as a key regulator of macrophage-mediated inflammation and ECM remodeling in IVDD. Its high expression in pro-inflammatory M1 macrophages promotes matrix degradation and inflammatory responses during the degeneration process. Overexpression of *SPP1* not only exacerbates immune cell activation but also accelerates the degeneration of IVDs by activating MMPs and inducing apoptosis of NP cells. Additionally, we used phage display technology to screen and identify a peptide that specifically targets AF cells, with the sequence SNTALPSKFYGY. Furthermore, to overcome the delivery barriers within the AF microenvironment and achieve precise regulation of macrophage function, we designed and constructed a responsive, targeted nanotherapy platform, named AF-PTK@LEV@SPP1. This platform uses an AF cell-specific peptide for stable anchoring and long-term retention in the AF region. Additionally, we utilized ROS-responsive thioketone linkers to sense local oxidative stress levels and trigger rapid drug release upon macrophage infiltration and inflammation, thereby enabling condition-dependent therapeutic activation. Moreover, by fusing detoxified bacterial OMVs with liposomes, we created stable membrane fusion nanoparticles (LEV), significantly improving siRNA encapsulation and delivery efficiency. This system effectively targets inflammatory macrophages in lesion areas and silences the SPP1 gene. This delivery system not only achieves specific recognition and precise enrichment of target cells in space but also provides dynamic responsiveness over time, effectively blocking the pro-inflammatory polarization of macrophages mediated by *SPP1*, alleviating local inflammation, protecting matrix structures, and promoting tissue regeneration. Overall, our study highlights the potential of macrophage-centered immune modulation strategies for IVDD treatment, providing a therapeutic approach for immune function remodeling and disc regeneration through a programmable, targeted, and dynamically responsive siRNA delivery system.

## Results

### Intervertebral disc degeneration is accompanied by the accumulation of macrophages

**Fig. 1:**
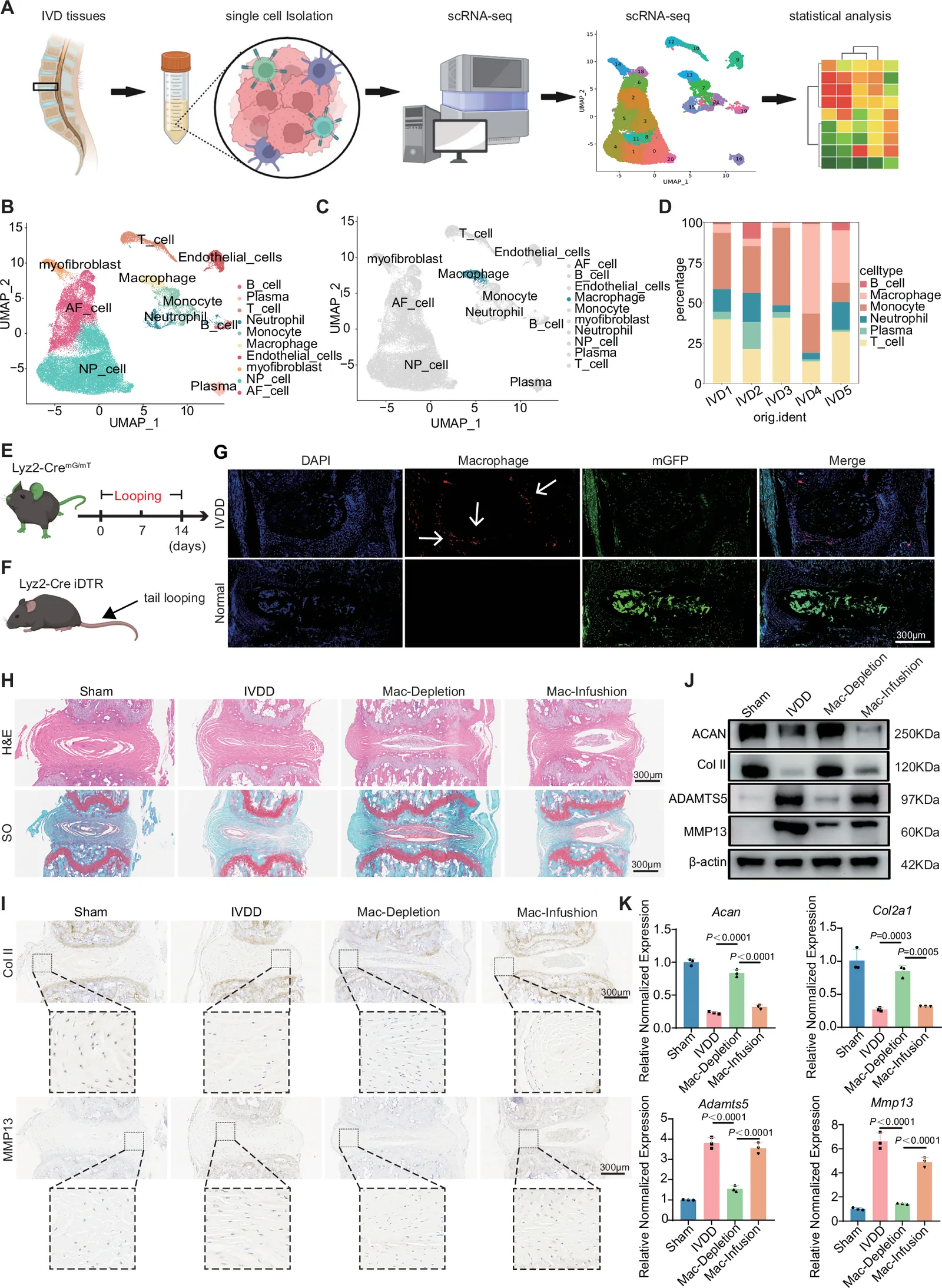
Macrophage infiltration in degenerating intervertebral discs. **A** Schematic representation of human intervertebral disc single-cell sequencing(Created in BioRender. Chai, X. (2026) https://BioRender.com/1jbzc0w). **B** UMAP plot of various cell clusters in intervertebral disc cells. **C** UMAP plot showing macrophage distribution in intervertebral disc cells. **D** Statistical identification of immune cell types across different sample groups. **E** Schematic diagram of macrophage-specific fluorescent mice (Lyz2-Cre;mG/mT)(Created in BioRender. Chai, X. (2026) https://BioRender.com/1jbzc0w). **F** Schematic diagram of macrophage-specific knockout mice (Lyz2-Cre;DTR)(Created in BioRender. Chai, X. (2026) https://BioRender.com/1jbzc0w). **G** Fluorescent imaging of frozen sections from macrophage-specific fluorescent mice after tail-loop surgery (IVDD:Lyz2-Cre;mG/mT mice with tail-loop surgery;Normal:Lyz2-Cre;mG/mT mice without tail-loop surgery). **H** H&E and Safranin O staining of intervertebral discs in mice treated with different interventions(Sham: WT mice without tail-loop surgery; IVDD: WT mice with tail-loop surgery; Mac-Depletion:Lyz2-Cre;iDTR mice with tail-loop surgery; Mac-Infusion:Lyz2-Cre;iDTR mice receiving macrophage infusion after tail-loop surgery). **I** Representative immunohistochemical staining of Col II and MMP13 in intervertebral discs of different intervention groups. **J** Western blot analysis of ACAN, Col II, ADAMTS5, and MMP13 protein expression in annulus fibrosus tissue of intervertebral discs from different intervention groups. **K** qRT-PCR analysis of Acan, Col2a1, Adamts5, and Mmp13 gene expression in annulus fibrosus tissue of intervertebral discs from different intervention groups. Data are presented as mean ± SD. *P* value was determined from one-way ANOVA with a Tukey post-hoc test (**K**). Representative images are shown. Quantitative analyses were performed using three independent animals per group (*n* = 3). Source data are provided as a Source Data file.

To investigate the cellular composition of the IVD, we collected degenerated disc samples from five patients aged between 65 and 80 years, whose representative magnetic resonance imaging (MRI) features are shown in Supplementary Fig. 1A. Detailed patient information, diagnosis, clinical treatment methods, and Pfirrmann grading are presented in Supplementary Fig. 1B. Single-cell suspensions were prepared from the whole degenerated discs and subjected to single-cell RNA sequencing (scRNA-seq). Utilizing the 10x Genomics platform in combination with microfluidics, we successfully isolated individual cells and performed high-throughput sequencing using the Illumina platform. This enabled rapid and efficient transcriptomic profiling and downstream analysis of thousands of single cells at high resolution (Fig. 1A).

In total, we obtained transcriptomic profiles from 53,416 cells across five samples, with 9751 to 11,532 cells captured per sample. The median number of detected genes per cell ranged from 1350 to 2438. After quality filtering, between 7814 and 9298 high-quality cells were retained per sample. Unsupervised clustering analysis identified 21 distinct cell populations. Based on canonical marker expression and transcriptional profiles, the IVD microenvironment was classified into ten major cell types: NP cells, AF cells, macrophages, endothelial cells, plasma cells, myofibroblasts, monocytes, neutrophils, B cells, and T cells (Fig. 1B). Notably, aside from NP and AF cells, macrophages accounted for a substantial proportion of cells (Fig. 1C), constituting approximately 12.27% of all immune cells (Fig. 1D). These findings provide a comprehensive single-cell landscape of the degenerated IVD, highlighting the cellular heterogeneity and implicating macrophages as a prominent immune cell population potentially involved in disc pathology.

To further explore the role of macrophages in degenerative disc disease, we generated a macrophage-specific fluorescent reporter mouse model (Lyz2-Cre;mG/mT), in which macrophages express membrane-localized tdTomato (red fluorescence), while all other cell types express membrane-localized GFP (green fluorescence), thereby allowing specific visualization of macrophages (Fig. 1E). To validate the genetic background of the model, genotyping by PCR was performed and confirmed the presence of both Lyz2-iCre and mG/mT alleles (Supplementary Fig. 2B, D). Strong GFP fluorescence was observed in the ears, paws, tail, and skin, consistent with Cre activity under the control of the Lyz2 promoter (Supplementary Fig. 2E), as indicated by the red arrows. Lyz2-Cre;mG/mT mice were divided into two groups: a normal control (Normal) group without tail-loop surgery and a tail-loop-induced IVDD group. The tail-loop model and its validation are shown in Supplementary Fig. 2F, G, and the modeling process was maintained for two weeks. Notably, minimal macrophage infiltration was observed in healthy IVDs, whereas prominent tdTomato-positive macrophages were detected within degenerated discs two weeks post tail-loop induction (Fig. 1G). Using a macrophage-specific fluorescent reporter mouse model (Lyz2-Cre;mG/mT), we demonstrated that macrophage infiltration is markedly increased in IVDs following tail-loop-induced disc degeneration, in contrast to the minimal presence observed in healthy discs. This model enables precise in vivo visualization of macrophages and provides a robust platform for investigating immune cell dynamics during disc degeneration.

To investigate the functional relevance of macrophages during IVDD progression, we established a macrophage-depletion mouse model (Lyz2-Cre;iDTR) that allows selective ablation of macrophages via diphtheria toxin (DT) administration (Fig. 1F). Genotyping confirmed the successful generation of Lyz2-Cre;iDTR double-positive mice (Supplementary Fig. 2A, C). Mice were divided into four experimental groups: Sham: wild-type (WT) mice without tail looping；IVDD:WT mice with tail-loop induced disc degeneration；Mac-Depletion: Lyz2-Cre;iDTR mice receiving DT-mediated macrophage ablation followed by tail looping, and Mac-Infusion: Lyz2-Cre;iDTR mice subjected to macrophage depletion and tail looping, followed by macrophage infusion (2 μL of freshly isolated bone marrow–derived macrophages [BMDMs], ~2 × 10⁵ cells, microinjected into the AF and NP region on postoperative day 3 using a 34 G microsyringe).

To evaluate the structural and molecular consequences of macrophage depletion or supplementation during IVDD, we performed histological and immunohistochemical analyses(Fig. 1H). Histological examination (H&E and Safranin O staining) revealed that the sham and Mac-Depletion groups preserved disc architecture following tail-loop surgery, while the IVDD and Mac-Infusion groups exhibited characteristic degenerative features, including NP tissue loss, disorganized AF structure, and reduced disc height (Fig. 1H). Consistently, histological degeneration scoring (Masuda score, Supplementary Fig. 3A) showed a significantly higher score in the IVDD group and Mac-Infusion group, indicating severe degeneration, which was attenuated in the Mac-Depletion group. Immunohistochemical analysis showed significantly increased type II collagen (Col II) and decreased matrix metalloproteinase-13 (MMP13) expression in the Mac-Depletion group compared to the IVDD group and Mac-Infusion, suggesting a protective effect mediated by macrophage depletion (Fig. 1I and Supplementary Fig. 3B, C).

To further investigate the impact of macrophage depletion or supplementation on disc matrix metabolism, we specifically dissected the AF tissue from mouse IVDs for molecular analysis. Western blotting and quantitative PCR were subsequently performed to assess the expression of key anabolic and catabolic matrix markers (Fig. 1J, K). At the protein level (Fig. 1J), ACAN and Col II were significantly downregulated in the IVDD group compared to the Sham group, while ADAMTS5 and MMP13 were markedly upregulated, indicating matrix degradation. Notably, these degenerative changes were further exacerbated in the Mac-Infusion group following macrophage supplementation. In contrast, macrophage depletion (Mac-Depletion group) resulted in partial restoration of ACAN and Col II levels and suppression of ADAMTS5 and MMP13 expression, suggesting a protective effect of macrophage ablation. Quantitative protein data are presented in Supplementary Fig. 3D–G. This pattern was further validated at the transcriptional level (Fig. 1K). Quantitative PCR showed that *Acan* and *Col2a1* mRNA expression was elevated in the Mac-Depletion group compared to the IVDD group, while the expression of *Adamts5* and *Mmp13* was reduced. Conversely, macrophage supplementation again led to enhanced catabolic marker expression and reduced matrix synthesis genes. Collectively, these results demonstrate that macrophage accumulation exacerbates disc degeneration, while selective depletion of macrophages protects disc matrix integrity—highlighting a detrimental role of macrophages in the progression of IVDD.

To rule out the possibility that the observed disc phenotypes were caused by off-target depletion of osteoclasts due to Lyz2 expression, we further examined the vertebrae adjacent to the degenerated discs. TRAP staining was performed to detect osteoclast activity (Supplementary Fig. 4A). The number of TRAP-positive multinucleated osteoclasts remained comparably low across all groups, including Mac-Depletion and Mac-Infusion, suggesting that the Lyz2-Cre;iDTR model did not induce substantial osteoclast ablation. In addition, micro-computed tomography (Micro-CT) analysis was conducted to evaluate the structural integrity of the adjacent vertebral bone (Supplementary Fig. 4B). Quantitative analysis revealed no significant differences in bone volume fraction (BV/TV) or bone mineral density (BMD) among the experimental groups (Supplementary Fig. 4C, D). These findings confirm that vertebral bone mass and osteoclast activity were not significantly affected by macrophage depletion, thereby excluding indirect bone-driven effects on disc degeneration. Together with the aggravated degeneration observed upon macrophage infusion and the structural preservation after macrophage depletion, our data provide compelling evidence that macrophages directly regulate IVD degeneration.

### High expression of *SPP1*⁺ macrophages in intervertebral disc degeneration and functional inhibition of M1 polarization via SPP1 knockdown

**Fig. 2:**
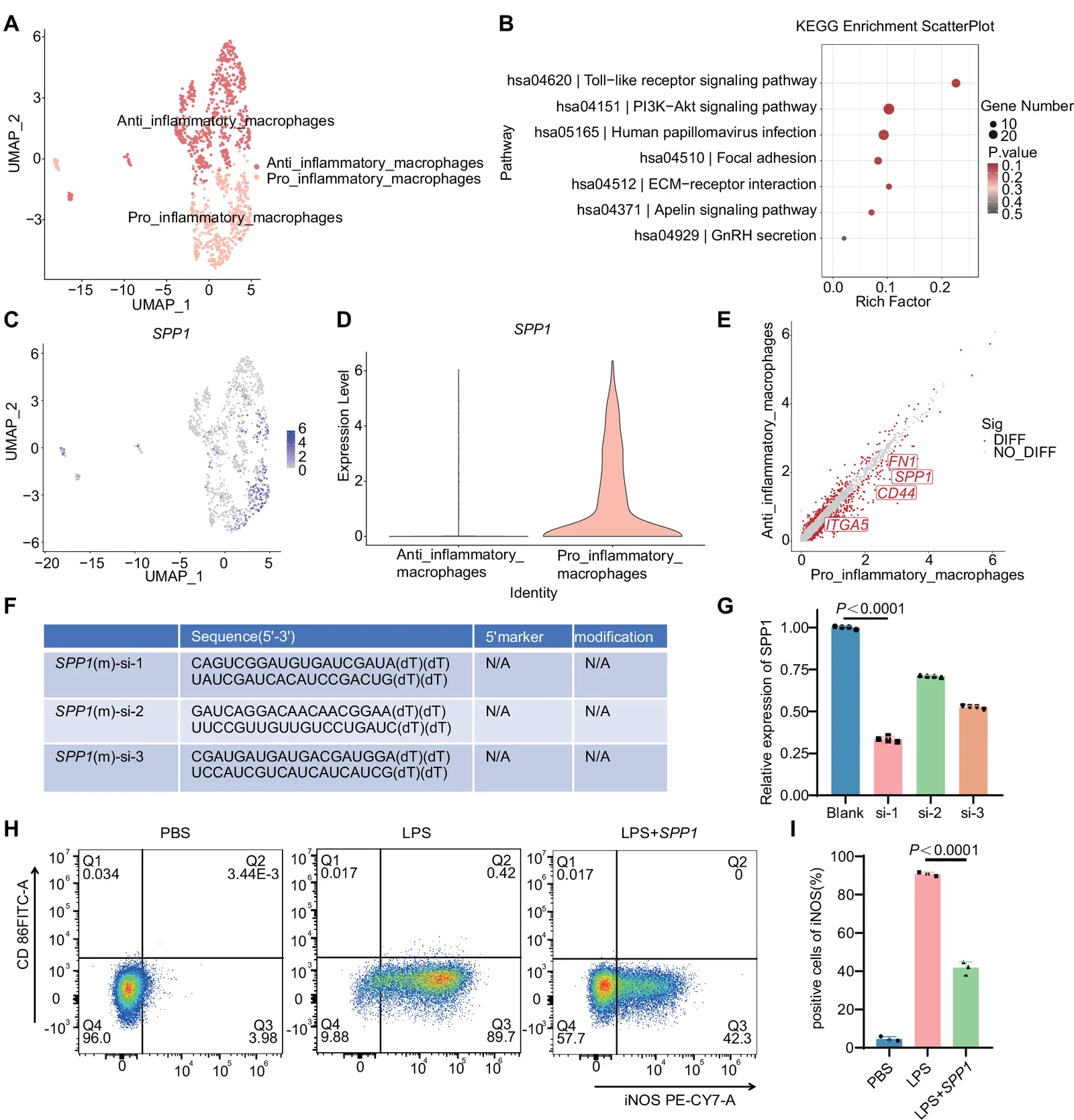
High expression of SPP1⁺ macrophages in intervertebral disc degeneration and downregulation of SPP1 inhibits M1 polarization. **A** UMAP plot showing distinct clustering of pro-inflammatory and anti-inflammatory macrophage subpopulations. **B** KEGG pathway enrichment analysis of genes upregulated in pro-inflammatory macrophages. A two-sided hypergeometric test was used, and *P* values were FDR corrected. The significance threshold for pathway enrichment was set to an adjusted *P* value < 0.05. **C** UMAP visualization of SPP1 expression across macrophage subsets. **D** Violin plot illustrating the differential expression of SPP1 in pro- versus anti-inflammatory macrophages. **E** Volcano plot showing differentially expressed genes, with SPP1, FN1, CD44, and ITGA5 significantly upregulated in pro-inflammatory macrophages. **F** Design and sequences of three siRNAs targeting Spp1. **G** qRT-PCR analysis showing effective knockdown of SPP1 expression, with si-1 demonstrating the highest efficiency. *n* = 4 biologically independent cell cultures (mean ± SD). **H** Flow cytometry analysis of CD86 and iNOS expression in bone marrow–derived macrophages treated with PBS, LPS, or LPS + SPP1 siRNA. **I** Quantification of iNOS⁺ cells, indicating reduced M1 polarization following SPP1 knockdown. *n* = 3 biologically independent cell cultures (mean ± SD). *P* value was determined from one-way ANOVA with a Tukey post-hoc test (**G**, **I**). All in vitro experiments were independently repeated at least three times with similar results. Source data are provided as a Source Data file.

To further dissect the immune heterogeneity within the degenerated IVD, we performed reclustering of macrophages using scRNA-seq data. Based on distinct transcriptomic signatures, macrophages were classified into pro-inflammatory and anti-inflammatory subtypes (Fig. 2A). Functional pathway analysis of differentially expressed genes between these two subsets revealed that pro-inflammatory macrophages were enriched for immune activation-related pathways, including Toll-like receptor signaling, PI3K-Akt signaling, and ECM–receptor interaction (Fig. 2B), all of which are known to be involved in the pathogenesis of tissue degeneration.

Among the differentially expressed genes, Secreted Phosphoprotein 1 (*SPP1*) emerged as a top candidate, highly expressed in pro-inflammatory macrophages. UMAP projection and violin plot analysis demonstrated that SPP1 expression was almost exclusively enriched in the pro-inflammatory subset, whereas anti-inflammatory macrophages showed negligible expression (Fig. 2C, D). A volcano plot further confirmed *SPP1*, along with *FN1*, *CD44*, and *ITGA5*, as significantly upregulated markers in this subset (Fig. 2E). *SPP1*, also known as osteopontin, has previously been implicated in tissue remodeling, macrophage chemotaxis, and chronic inflammation, suggesting that it may play a critical role in mediating disc degeneration through immune activation and matrix degradation.

To functionally investigate the role of *SPP1* in macrophage polarization, we designed three small interfering RNAs (siRNAs) targeting *SPP1* mRNA and evaluated their knockdown efficiency (Fig. 2F). Quantitative RT-PCR confirmed that siRNA-1 (si-1) was the most effective in suppressing *SPP1* transcript levels (Fig. 2G). We then utilized this construct in an in vitro macrophage polarization model, where bone marrow–derived macrophages were stimulated with LPS, with or without *SPP1* knockdown. Flow cytometry analysis revealed that *SPP1* silencing significantly reduced the population of CD86⁺ iNOS⁺ M1-polarized macrophages (Fig. 2H), and quantitative analysis showed a marked decline in iNOS⁺ cell percentages (Fig. 2I), indicating effective suppression of pro-inflammatory activation.

Taken together, these results demonstrate that *SPP1*⁺ macrophages represent a distinct pro-inflammatory subpopulation in degenerated IVD tissue, and that *SPP1* is functionally required for M1 polarization. Its knockdown attenuates pro-inflammatory macrophage responses, suggesting that *SPP1* may serve as a promising therapeutic target for modulating immune-mediated tissue degeneration in IVD disease.

### Identification and characterization of annulus fibrosus (AF)-targeting peptides using phage display and fluorescence imaging

**Fig. 3:**
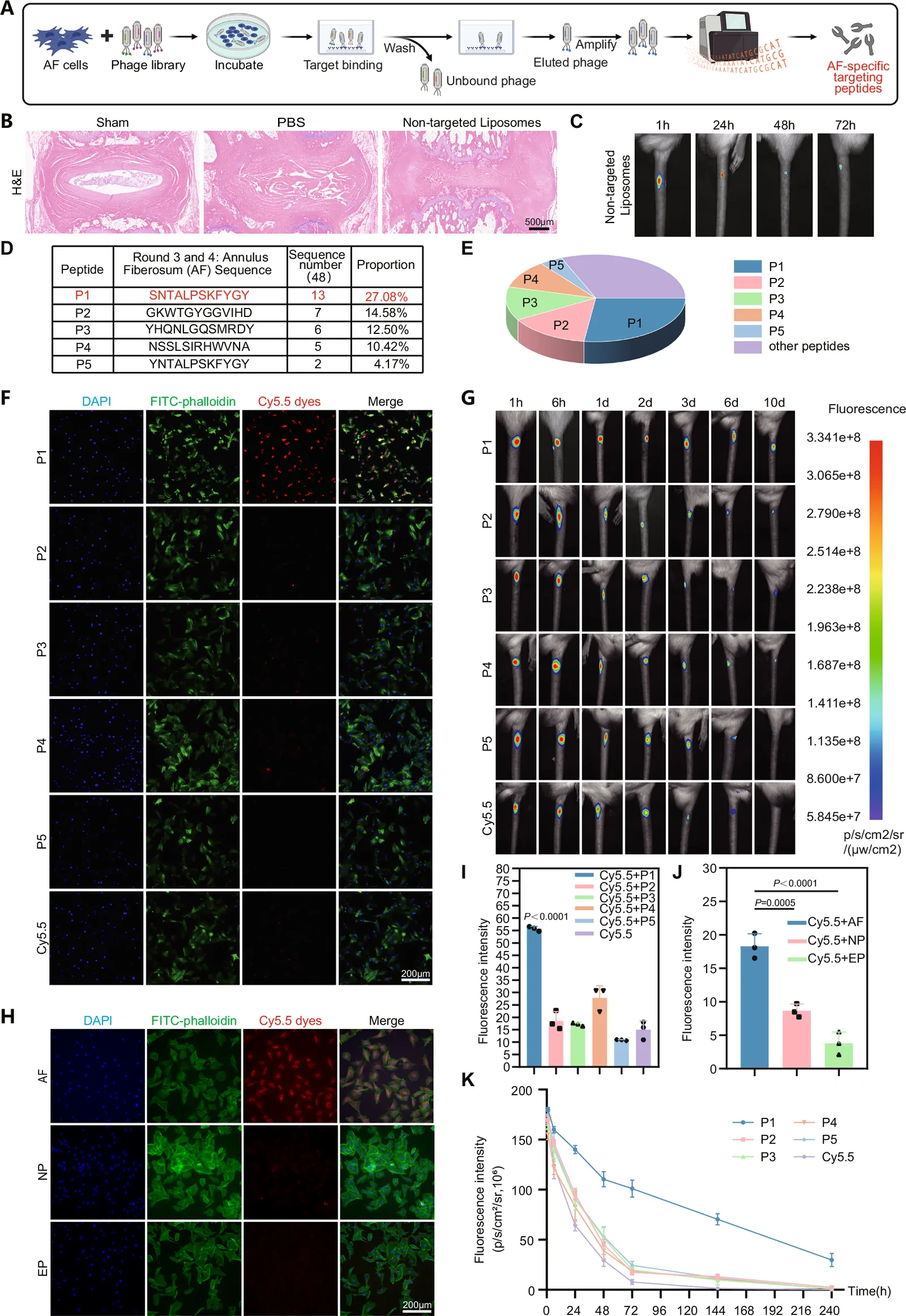
Identification and characterization of annulus fibrosus (AF)-targeting peptides using phage display and fluorescence imaging. **A** Schematic illustration of phage display bio-panning used to select specific targeted peptides for annulus fibrosus (AF) cells(Created in BioRender. Chai, X. (2026) https://BioRender.com/1jbzc0w). **B** H&E staining of degenerated intervertebral discs after treatment with conventional non-targeted liposomes. **C** In vivo imaging of DiR-labeled non-targeted liposomes in rat discs at indicated time points. Summary of peptide sequences (**D**) and frequency (**E**) in rounds 3 and 4 of phage display selection targeting AF cells. **F** Confocal images showing binding of Cy5.5-labeled peptides 1–5 (10 μM, 30 min) to FITC-phalloidin-labeled AF cells. **G** Time-course in vivo imaging of Cy5.5-labeled peptides in rat intervertebral discs over 10 days. **H** Confocal microscopy to determine the specificity of P1. Cy5.5-P1 was incubated with FITC-phalloidin-labeled AF, NP, and EP cells (10 μM, 30 min). **I** Fluorescence intensity of AF cells stained with Cy5.5-peptides. **J** Fluorescence intensity of Cy5.5-P1 binding to AF cells, NP cells, and cartilage endplate cells. **K** Time-dependent decay of fluorescence signal from in vivo imaging. Representative images are shown. Data are presented as mean ± SD (*n* = 3 biologically independent samples per group). *P* value was determined from one-way ANOVA followed by Dunnett’s post hoc test (**I**, **J**). Source data are provided as a Source Data file.

The IVD, the largest avascular organ in the human body, currently lacks effective targeted therapies. Conventional liposomes, though widely used as drug delivery carriers, exhibit poor retention and limited therapeutic efficacy in the IVD due to its unique anatomical and biological barriers. To evaluate this limitation, we employed an acupuncture-induced IVDD model in 8-week-old rats, randomly assigning them to Sham (mock surgery), PBS, or conventional liposome treatment groups. After 8 weeks, the animals were euthanized, and disc tissues were collected for histological analysis using hematoxylin and eosin (H&E) staining (Fig. 3B). The severity of disc degeneration was quantitatively assessed using the Masuda scoring system (Supplementary Fig. 5A). To evaluate the targeting capacity and retention behavior of conventional liposomes in vivo, we labeled them with the near-infrared fluorescent dye DiR and performed fluorescence imaging. As shown in Fig. 3C, conventional (non-targeted) liposomes exhibited no significant difference in fluorescence distribution compared to free DiR dye (Supplementary Fig. 5B), indicating minimal targeting capability. By 72 h post-injection, the fluorescence signal in the disc region had nearly diminished to the detection limit (Supplementary Fig. 5C), suggesting rapid clearance of the liposomes from the IVD. These findings underscore the necessity for engineering liposomal systems with enhanced tissue specificity and prolonged retention to overcome the delivery challenges posed by the disc microenvironment.

To address this issue, we initially identified peptides targeting AF cells through four rounds of in vitro phage display biopanning (Fig. 3A). Phages from the third and fourth rounds of in vitro output were sequenced, revealing five potential high-affinity peptides from a total of 48 clones: SNTALPSKFYGY (13/48), GKWTGYGGVIHD (7/48), YHQNLGQSMRDY (6/48), NSSLSIRHWNA (5/48) (denoted as peptide 1, peptide 2, peptide 3, peptide 4 and peptide 5, respectively) (Fig. 3D). These peptides were selected as potential targeting ligands for AF cells (Fig. 3D).

To facilitate further labeling and modification, the peptides were synthesized with a serine (S) and cysteine (C) at the C-terminus for conjugation. Mass spectrometry and chromatograms confirmed the successful synthesis of these peptides.

To identify the peptide with the highest targeting efficacy for AF cells, we tested the binding capacity of the candidate peptides in both in vitro and in vivo models. All peptides, including peptides 1, 2, 3, 4, 5, were labeled with the Cy5.5 dye for detection upon binding to AF cells. As shown in Fig. 3F, only the peptide 1 group exhibited strong Cy5.5 fluorescence signals, while the other groups showed weak background fluorescence. The average fluorescence intensity (MFI) of peptide 1 (55.715) was 3.01 times higher than peptide 2 (18.531), 3.24 times higher than peptide 3 (17.198), 2.00 times higher than peptide 4 (27.869), and 5.16 times higher than peptide 5 (10.807) (Fig. 3F).

To validate the cell-type specificity of peptide 1 (P1), we co-cultured Cy5.5-labeled P1 with AF cells, NP cells, and cartilage endplate (EP) cells. As shown in Fig. 3H, P1 displayed robust binding to AF cells, whereas minimal fluorescence was observed in NP and EP cells. Quantitative analysis of fluorescence intensity (Fig. 3J) confirmed the selective affinity of P1 for AF cells, supporting its potential as a targeting ligand for AF tissue.

Next, we assessed the in vivo targeting ability of the candidate peptides using a needle injection approach. Peptide-Cy5.5 conjugates and free Cy5.5 dye were intradiscally injected into rat IVDs, and the retention of fluorescent signal was monitored using the AniView multi-modal in vivo imaging system (BLT Biotech) (Fig. 3G). Fluorescence intensity was recorded at multiple time points: 1 h, 6 h, 24 h, 2 days, 3 days, 6 days, and 10 days post-injection. At the initial time point, peptides 1 through 5 exhibited comparable fluorescence intensities (average:172.31). However, by day 3 (72 h), the signals from peptides 2 through 5 had largely diminished (fluorescence intensity < 5), whereas peptide 1 maintained a detectable signal even on day 10, with a remaining intensity of 29.67 (Fig. 3K). In summary, peptide 1 exhibited superior targeting specificity for AF cells, both in vitro and in vivo, with sustained retention in the IVD.

To rigorously assess the targeting specificity of peptide 1 (P1), we synthesized a scrambled version of the peptide (control sequence: YLNSYSFAGKPT) and evaluated its binding behavior in both in vitro and in vivo models. In cultured AF cells, we incubated Cy5.5-labeled P1, scrambled P1, and a no-peptide control at 10 μM for 30 min, followed by imaging via confocal microscopy (Supplementary Fig. 6A). Fluorescent signals revealed that P1 showed strong surface binding and cytoplasmic accumulation in AF cells, whereas the scrambled sequence and dye-only controls resulted in weak or negligible fluorescence, indicating minimal non-specific uptake. To quantitatively compare targeting performance, we measured the relative fluorescence intensity in each group (Supplementary Fig. 6D). P1-treated cells exhibited a significantly higher mean fluorescence intensity (MFI) than both scrambled P1 and no-peptide controls (*p* < 0.0001), demonstrating the sequence-dependent specificity of P1 binding to AF cells.

Next, we performed in vivo validation using a rat intradiscal injection model. Cy5.5-labeled P1, scrambled P1, and free dye were injected into the IVDs, and fluorescent retention was longitudinally monitored using small animal imaging (Supplementary Fig. 6B). While all three groups showed comparable initial signals at 1 h, signal from scrambled P1 and free dye diminished rapidly after 24–48 h. In contrast, P1 remained detectable in the disc space up to 10 days post-injection, with an average fluorescence intensity of ~34.06 units at 240 h (Supplementary Fig. 6E), confirming its superior in vivo retention and targeting stability.

We further investigated whether P1 could recognize AF cells across different species. Confocal microscopy after incubation with Cy5.5-labeled P1 showed robust binding to rat, human, and mouse AF cells (Supplementary Fig. 6C). Quantitative analysis (Supplementary Fig. 6F) revealed that although signal intensity was highest in rat cells, human and mouse AF cells also demonstrated consistent uptake, supporting the cross-species translational potential of P1 as an AF-targeting moiety.

Together, these results provide compelling evidence that P1 exhibits high affinity and specificity for AF cells in a sequence-dependent, cell-type–specific, and cross-species–compatible manner. Its prolonged in vivo retention and negligible off-target accumulation further support its application as a promising delivery vehicle for targeted therapeutic strategies in IVDD.

### Characterization of AF-PTK@LEV@SPP1 membrane fusion bacterial EVs

**Fig. 4:**
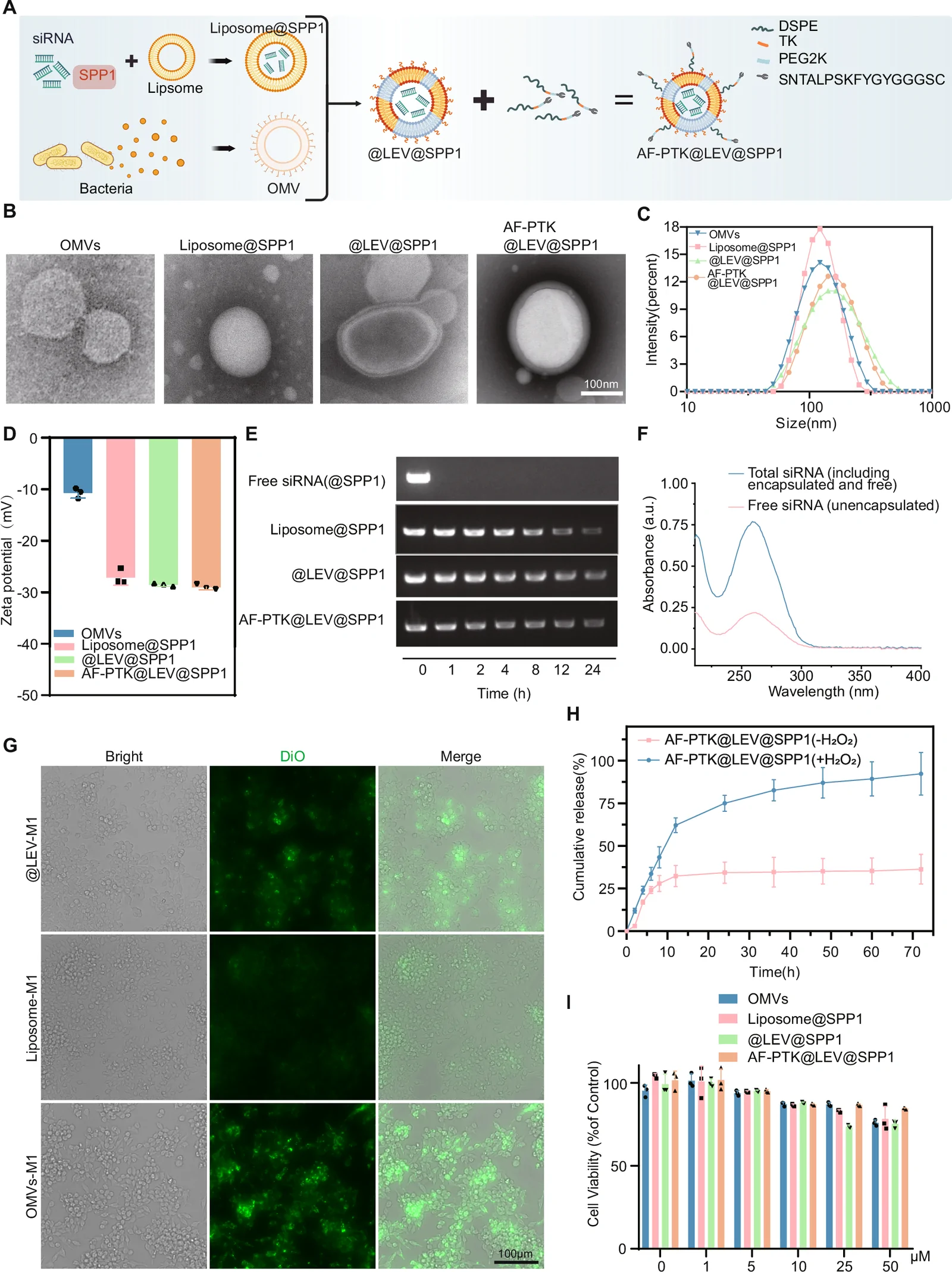
Characterization of AF-PTK@LEV@SPP1 membrane fusion bacterial extracellular vesicles. **A** Schematic illustration of the synthesis of AF-PTK@LEV@SPP1:Schematic representation of the construction process of AF-PTK@LEV@SPP1 nanoparticles, integrating liposome-encapsulated siRNA, outer membrane vesicles (OMVs), and annulus fibrosus (AF)-binding peptide via ROS-responsive thioketal linkers(Created in BioRender. Chai, X. (2026) https://BioRender.com/1jbzc0w). **B** Transmission electron microscopy (TEM) images of OMVs, Liposome@SPP1, @LEV@SPP1, and AF-PTK@LEV@SPP1. Scale bar = 100 nm. **C** Dynamic light scattering (DLS) analysis of particle size distribution profiles for the individual components and the AF-PTK@LEV@SPP1. **D** Zeta potential measurements of OMVs, Liposome@SPP1, @LEV@SPP1, and AF-PTK@LEV@SPP1. (*n* = 3 biologically independent samples per group, mean ± SD). **E** Agarose gel electrophoresis assessing siRNA stability in different formulations over time under enzymatic degradation conditions. Four groups were tested: free siRNA, Liposome@SPP1, @LEV@SPP1, and AF-PTK@LEV@SPP1. **F** UV–visible spectroscopy showing absorbance profiles of total siRNA (encapsulated and unencapsulated) and free siRNA alone. **G** Fluorescence microscopy images of M1 macrophages and AF cells incubated with DiO-labeled LEV and liposome formulations to assess cellular uptake specificity. Scale bar = 100 μm. **H** In vitro cumulative siRNA release from AF-PTK@LEV@SPP1 in the presence and absence of hydrogen peroxide (H₂O₂) at 100 μM, monitored over time. (*n* = 3 biologically independent samples per group, mean ± SD) **I** CCK-8 assay assessing the cytocompatibility of OMVs, Liposome@SPP1, @LEV@SPP1, and AF-PTK@LEV@SPP1 at various concentrations. (*n* = 3 biologically independent samples per group, mean ± SD). All experiments were independently repeated at least three times with similar results. Source data are provided as a Source Data file.

To precisely modulate macrophage function within the degenerated AF, we engineered a multi-functional delivery platform—AF-PTK@LEV@SPP1—that integrates^1^ siRNA cargo against *SPP1*^2^, macrophage-tropic bacterial OMVs^3^, ROS-sensitive thioketal (TK) linkers, and^4^ an annulus fibrosus–targeting peptide (AF-P) (Fig. 4A). This modular design enables tissue-specific accumulation, inflammatory signal–responsive release, and cell-type–specific uptake.

We initially constructed Liposome@SPP1 nanoparticles to encapsulate siRNA via lipid self-assembly, followed by isolation of OMVs from genetically attenuated E. coli (BL21-ΔmsbB). Through membrane extrusion and sonication, liposomes and OMVs were fused into hybrid vesicles (@LEV@SPP1), exhibiting uniform morphology with a mean size of ~114 nm. Next, AF-P was conjugated to @LEV@SPP1 using a PEG2K spacer and TK linker, resulting in the final formulation AF-PTK@LEV@SPP1.

Transmission electron microscopy (TEM) revealed that the nanoparticles exhibited clear bilayered spherical structures. Dynamic light scattering (DLS) measurements showed that the average particle size was approximately 114.6 nm for OMVs, 115.6 nm for Liposome@SPP1, and increased to around 161.6 nm for AF-PTK@LEV@SPP1, with all formulations displaying a polydispersity index (PDI) below 0.2, indicating good dispersion uniformity (Fig. 4B–C). Zeta potential analysis demonstrated that all nanoparticles were negatively charged, with values ranging from −28 to −32 mV, consistent with good stability in suspension (Fig. 4D). Agarose gel electrophoresis (Fig. 4E) further demonstrated that AF-PTK@LEV@SPP1 protected siRNA from enzymatic degradation for up to 24 h, significantly outperforming free siRNA(Supplementary Fig. 12). UV–visible spectroscopy confirmed siRNA encapsulation efficiency (Fig. 4F) and absorbance shifts associated with nanocomposite formation (Supplementary Fig. 7B). SEM-EDS analysis visualized the overall morphology and verified the elemental composition of the final nanoparticles (Supplementary Fig. 7A).

To assess cellular uptake specificity across different nanoparticle formulations, we incubated DiO-labeled @LEV, Liposome@SPP1, and OMVs with M1-polarized macrophages. As shown in Fig. 4G, @LEV exhibited preferential uptake by macrophages, with markedly less internalization observed in Liposome@SPP1, indicating macrophage-specific targeting conferred by the OMV component. In contrast, liposomes alone showed poor uptake in M1-polarized macrophages, highlighting the necessity of membrane fusion with OMVs to enhance cellular entry. Notably, OMVs demonstrated the strongest phagocytosis among the three groups, suggesting that bacterial membrane-derived vesicles possess innate macrophage-targeting capacity, which is partially retained in the @LEV formulation. Quantification of DiO fluorescence intensity in M1 macrophages after incubation with DiO-labeled @LEV, liposome, or OMVs formulations is in Supplementary Fig. 7E. To assess cellular uptake specificity across different cells, we incubated DiO-labeled @LEV@SPP1 with M1 macrophages and AF cells. As shown in Supplementary Fig. 7C, @LEV@SPP1 displayed preferential accumulation in macrophages. Quantification of fluorescence intensity (Supplementary Fig. 7D) confirmed significantly higher uptake in macrophages than in AF cells.

We next evaluated the ROS responsiveness of AF-PTK@LEV@SPP1. Upon exposure to 100 μM hydrogen peroxide, the cumulative release of siRNA increased significantly, reaching ~89% at 72 h, whereas only ~34% was released under basal conditions (Fig. 4H). This dynamic responsiveness was enabled by the thioketal linker, allowing controlled release in oxidative environments.

To ensure biocompatibility, AF cells were incubated with increasing concentrations (0–50 μM) of OMVs, Liposome@SPP1, @LEV@SPP1, and AF-PTK@LEV@SPP1. The CCK-8 assay revealed minimal cytotoxicity across all treatment groups, with cell viability exceeding 85% under all conditions (Fig. 4I), supporting favorable biosafety for biological applications.

Collectively, these results establish that AF-PTK@LEV@SPP1 is a rationally designed, AF-targeted, and ROS-activated nanoparticle capable of delivering siRNA specifically to macrophages, with high structural stability, efficient encapsulation, and excellent biosafety (Fig. 4 and Supplementary Fig. 7)

### ROS-responsive intercellular trafficking of AF-PTK@LEV@SPP1 nanoparticles reprograms macrophage polarization and restores annulus fibrosus cell function

**Fig. 5:**
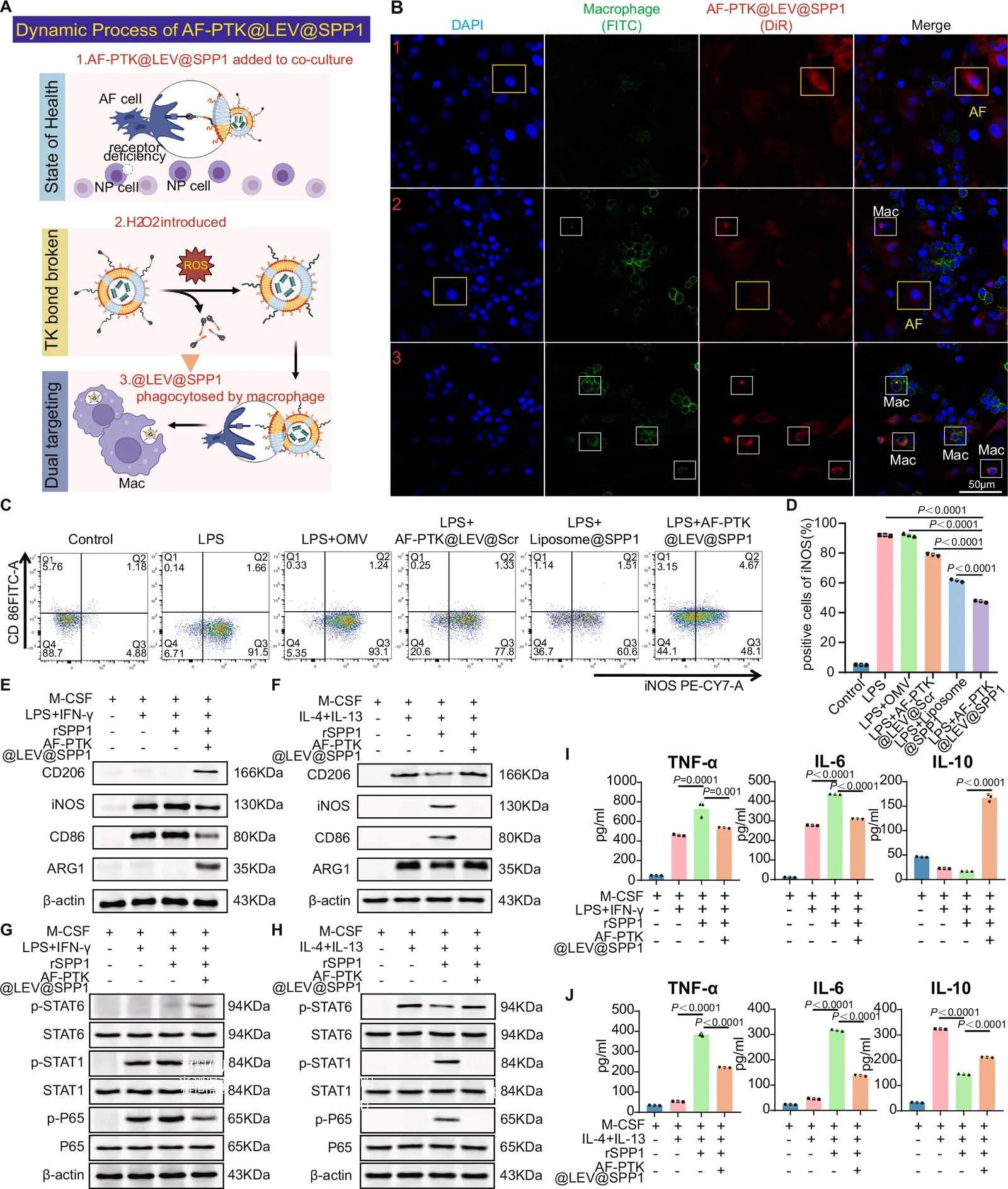
ROS-responsive intercellular transfer of AF-PTK@LEV@SPP1 nanoparticles reprograms macrophage polarization to mitigate intervertebral disc degeneration. **A** Schematic illustration of the dynamic phagocytosis process of AF-PTK@LEV@SPP1 by annulus fibrosus cells and bone-marrow-derived M1 macrophages (BMDM-M1) in sequence(Created in BioRender. Chai, X. (2026) https://BioRender.com/1jbzc0w). **B** Confocal microscopy showing the phagocytosis of AF-PTK@LEV@SPP1: nanoparticles were initially taken up by AF cells and subsequently engulfed by macrophages after co-culture. DAPI (nuclei, blue), FITC (macrophages, green), and DiR (AF-PTK@LEV@SPP1, red). Scale bar = 50 μm. **C** Flow cytometric analysis of macrophages stained for CD86 and iNOS under different treatment conditions. **D** Quantification of iNOS-positive macrophages based on flow cytometry from (**C**). (*n* = 3 biologically independent samples per group, mean ± SD). **E**–**H** Representative Western blot results showing total and phosphorylated levels of STAT1, STAT6, and P65, as well as M1/M2 markers (iNOS, CD86, CD206, and ARG1) in BMDMs under the indicated treatments. (*n* = 3 biologically independent samples per group, mean ± SD). **I**, **J** ELISA results showing TNF-α, IL-6, and IL-10 secretion in different groups. (*n* = 3 biologically independent samples per group, mean ± SD). Representative images are shown. All in vitro experiments were independently repeated at least three times with comparable results. Data are presented as mean ± SD from at least three independent experiments. *P* value was determined from one-way ANOVA followed by Dunnett’s post hoc test (**D**) and Tukey post-hoc test (**I**, **J**). Source data are provided as a Source Data file.

To elucidate the dynamic phagocytic behavior of AF-PTK@LEV@SPP1 nanoparticles and their role in intercellular signal transmission within the degenerative disc microenvironment, we employed a two-dimensional co-culture model composed of AF cells and bone-marrow-derived M1 macrophages (BMDM-M1). To enable clear cell-type distinction and precise tracking of nanoparticle transfer, we applied a three-channel fluorescence labeling strategy: macrophages were pre-labeled with FITC (green), all nuclei were counterstained with DAPI (blue), and AF-PTK@LEV@SPP1 nanoparticles were tagged with DiR (red). At baseline (Fig. 5B, Row 1), DiR fluorescence was exclusively localized within AF cells, with no overlap with FITC-labeled macrophages, confirming initial cell-specific targeting. Upon introduction of 100 μM H₂O₂ for 1 h to mimic elevated ROS levels (Row 2), we observed partial redistribution of the DiR signal. By 3 h post-stimulation (Row 3), DiR fluorescence had significantly diminished in AF cells and increased within FITC-positive macrophages, indicating ROS-induced cleavage of the thioketal linker and subsequent release and phagocytic uptake of the nanoparticles by macrophages. These findings provide strong evidence for the sequential, ROS-responsive delivery mechanism of our engineered nanosystem—initial targeting of AF cells followed by secondary re-targeting to macrophages—supporting its potential to bridge intercellular communication during disc degeneration.

To assess the regulatory effects on macrophage polarization, bone-marrow-derived macrophages (BMDMs) were divided into six groups: Control (no LPS stimulation), LPS (100 ng/mL), LPS + OMV, LPS + AF-PTK@LEV@Scr (Scr denotes a scrambled SPP1-siRNA; sense 5′-GAUGACGUACCUCGGAUUA-dTdT, antisense 5′-UAAUCCGAGGUACGUCAUC-dTdT-3′), LPS + Liposome@SPP1, and LPS + AF-PTK@LEV@SPP1. Flow cytometric analysis showed that LPS stimulation markedly elevated the expression of iNOS in BMDM-M1, a typical M1 macrophage marker, with over 90% of cells being iNOS-positive. Treatment with AF-PTK@LEV@SPP1 significantly reduced this proportion to approximately 48%, which was substantially lower than in the AF-PTK@LEV@Scr (77.8%) and Liposome@SPP1 (60.6%) control groups (Fig. 5C, D). These results indicate that AF-PTK@LEV@SPP1 effectively inhibits the pro-inflammatory M1 polarization of macrophages.

To explore the effect of SPP1 on macrophage polarization, we used bone marrow-derived macrophages (BMDMs) and induced M1 polarization by LPS and IFN-γ, and M2 polarization by IL-4 and IL-13.In these induced macrophages, we treated them with recombinant SPP1 protein or our AF-PTK@LEV@SPP1 delivery system, respectively. We analyzed M1 and M2 type signature proteins (CD86, iNOS, ARG1, CD206) and polarization-related transcription factors (phosphorylation levels of STAT1, STAT6, p65) by Western blot (Fig. 5E–H). In addition, ELISA measured the secretion of inflammatory factors such as TNF-α, IL-6, and IL-10(Fig. 5I, J). Western blot analysis showed that in M1 macrophages, overexpression of SPP1 significantly enhanced the expression of CD86 and iNOS, while in M2 macrophages, overexpression of SPP1 decreased the expression of ARG1 and CD206. In addition, after treatment with AF-PTK@LEV@SPP1, the expression levels of CD86 and iNOS in M1 macrophages were reduced, while the expressions of ARG1 and CD206 in M2 macrophages were slightly increased. ELISA results further proved that high expression of SPP1 can promote the release of pro-inflammatory cytokines such as TNF-α and IL-6. At the same time, after treatment with AF-PTK@LEV@SPP1, the secretion of TNF-α and IL-6 was significantly reduced, while the secretion of IL-10 was increased. To further verify the role of NF-κB in macrophage polarization, we performed nuclear translocation experiments of NF-κB (Supplementary Fig. 8A, B). In the experiment, the nuclear translocation of macrophages induced by M1 polarization was detected under different treatment conditions (rSPP1 and AF-PTK@LEV@SPP1). The results showed that SPP1 treatment significantly increased the nuclear translocation of NF-kB p65, while AF-PTK@LEV@SPP1 treatment reduced the nuclear translocation of NF-kB, further supporting the role of SPP1 in M1 polarization (Supplementary Fig. 8C, D).

To further assess how macrophage polarization states influence AF cells, we employed a Transwell-based co-culture system (Supplementary Fig. 8E) involving three groups: pro-inflammatory BMDM-M1 alone, pro-inflammatory BMDM-M1 with AF-PTK@LEV@Scr, and pro-inflammatory BMDM-M1 with AF-PTK@LEV@SPP1. Co-culture with inflammatory macrophages led to a marked reduction in aggrecan (ACAN) and collagen type II (Col II) expression in AF cells, accompanied by a significant increase in MMP13, indicating ECM degradation. Notably, treatment with AF-PTK@LEV@SPP1 restored ACAN and Col II expression and suppressed MMP13 upregulation, suggesting that this nanoplatform protects the matrix function of AF cells by modulating macrophage phenotype. In contrast, AF-PTK@LEV@Scr failed to exert a similar effect (Supplementary Fig. 8F, H). Comprehensive quantitative analyses of protein levels are provided in Supplementary Fig. 8J.

Moreover, we observed that exposure to pro-inflammatory BMDM-M1 downregulated the expression of stemness-related genes c-MYC, OCT4, and SOX2 in AF cells. This abnormal expression profile was not significantly altered by AF-PTK@LEV@Scr treatment. However, AF-PTK@LEV@SPP1 reversed the trend, leading to a significant upregulation of these stemness markers, suggesting that the system helps maintain the stem-like properties of AF cells under inflammatory conditions (Supplementary Fig. 8G, I). Comprehensive quantitative analyses of protein levels are provided in Supplementary Fig. 8K.

In summary, AF-PTK@LEV@SPP1 not only enables ROS-responsive intercellular nanoparticle trafficking but also alleviates IVDD at multiple levels by suppressing M1 macrophage polarization and improving the functional status of AF cells.

### In vitro and in vivo assessment of the affinity and specificity of AF-PTK@LEV@SPP1

**Fig. 6:**
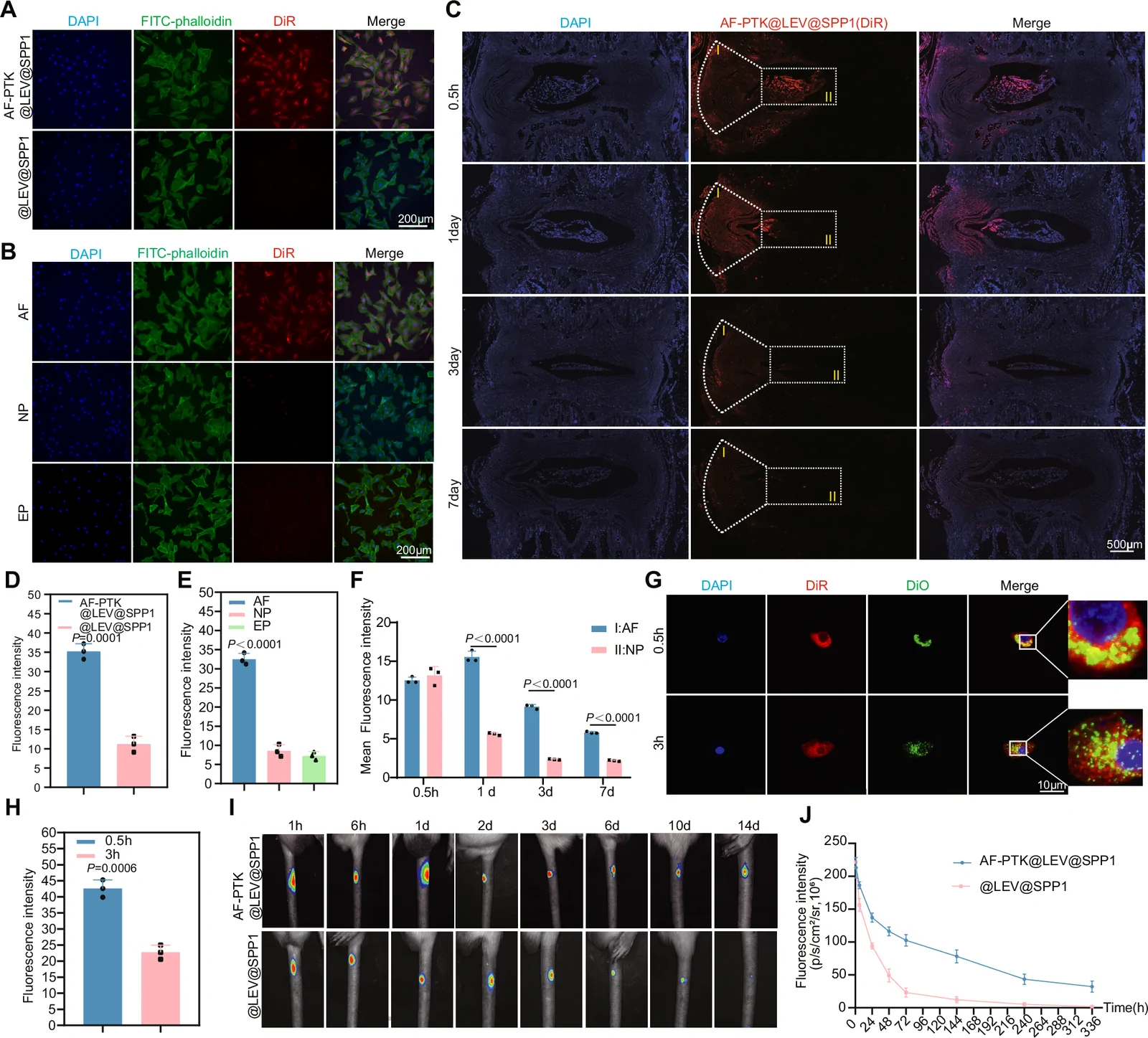
In vitro and in vivo fluorescence imaging and quantification of AF-PTK@LEV@SPP1 binding in cultured cells and frozen tissue sections of rat intervertebral discs. **A** Confocal microscopy showing the affinity of AF-PTK@LEV@SPP1 (DiR-labeled) with FITC-phalloidin-labeled AF cells after incubation at 10 μM for 30 min. Scale bar = 200 μm. **B** Confocal microscopy demonstrating the specificity of AF-PTK@LEV@SPP1. AF-PTK@LEV@SPP1 (DiR-labeled) incubated with FITC-phalloidin-labeled AF cells, NP cells, and cartilage endplate cells at 10 μM for 30 min. Scale bar = 200 μm. **C** Representative fluorescence images of frozen tissue sections from rat intervertebral discs at different time points (0.5 h, 1 day, 3 days, and 7 days) after the injection of AF-PTK@LEV@SPP1(DiR-labeled). Scale bar = 200 μm. **D** Fluorescence intensity of AF cells stained with DIR-labeled AF-PTK@LEV@SPP1. **E** Fluorescence intensity of AF-PTK@LEV@SPP1 with AF cells, NP cells, and cartilage endplate cells. **F** Quantification of mean fluorescence intensity in the frozen tissue sections at different time points (0.5 h, 1 day, 3 days, and 7 days) following AF-PTK@LEV@SPP1 injection, for two experimental groups: I:AF (blue bars) and II:NP (pink bars). **G** Confocal microscopy showing the binding of AF-PTK@LEV@SPP1 with annulus fibrosus cells at 0.5 h and 3 h. DiR-labeled annulus fibrosus cells and DiO-labeled AF-PTK@LEV@SPP1. **H** Fluorescence intensity of AF-PTK@LEV@SPP1 binding with annulus fibrosus cells at 0.5 h and 3 h. **I** In vivo fluorescence imaging of rat intervertebral discs at 1 h to 14 days post-injection with DiR-labeled AF-PTK@LEV@SPP1. **J** Fluorescence intensity of AF-PTK@LEV@SPP1 and @LEV@SPP1 in rat intervertebral discs. Representative images are shown. Quantification is based on three independent animals per group (*n* = 3 biologically independent samples per group). Data are presented as mean ± SD, with each point representing an independent sample. *P* value was determined using a two-sided unpaired Student’s *t* test (**D**, **H**) and Tukey post-hoc test (**F**). Source data are provided as a Source Data file.

To rigorously evaluate the targeting affinity and specificity of the engineered nanoparticle AF-PTK@LEV@SPP1 toward AF cells, we conducted confocal fluorescence imaging after incubating DiR-labeled AF-PTK@LEV@SPP1 (10 μM) with FITC-phalloidin-labeled primary AF cells for 30 min. Robust red fluorescence signals were observed exclusively in the AF-PTK@LEV@SPP1-treated group, indicating high cellular uptake and strong binding affinity (Fig. 6A). Quantitative analysis confirmed this finding, as the MFI reached 35.22 ± 2.00, demonstrating effective engagement with AF cells (Fig. 6D).

We next assessed the cell-type specificity of AF-PTK@LEV@SPP1 by comparing its binding performance across three major disc-derived cell types: AF cells, NP cells, and EP cells. After 30-min incubations, confocal microscopy revealed that the fluorescence signal was predominantly localized within AF cells, while NP and EP cells exhibited only minimal signal (Fig. 6B). Quantification supported these observations, showing significantly elevated fluorescence in AF cells (32.52 ± 1.53) compared to NP (8.60 ± 1.62) and EP (7.30 ± 1.04) groups (Fig. 6E, *p* < 0.0001). These results highlight the strong selectivity of the targeting construct toward AF tissue.

To determine in vivo localization and retention characteristics, we injected DiR-labeled AF-PTK@LEV@SPP1 into the IVDs of rats and performed frozen-section imaging at 0.5 h, 1 day, 3 days, and 7 days post-injection. Fluorescence signals were initially observed in both the AF and NP regions at 0.5 h; however, as time progressed, signals became increasingly restricted to the AF region. By day 7, distinct retention was evident in AF tissue, while fluorescence in NP regions diminished to background levels (Fig. 6C). Quantitative comparisons between AF (region I) and NP (region II) zones showed significantly higher fluorescence in AF at each time point (Fig. 6F), indicating selective accumulation and prolonged tissue retention in the AF.

To further visualize the dynamic interaction of AF-PTK@LEV@SPP1 with its cellular target, DiO-labeled AF-PTK@LEV@SPP1 was co-incubated with DiR-labeled AF cells, and confocal imaging was performed at 0.5 and 3 h. Co-localization of green and red fluorescence was evident at both time points (Fig. 6G), and quantitative intensity analysis showed that nanoparticle binding was sustained over the observed period (0.5 h: 42.57 ± 2.70; 3 h: 22.74 ± 2.21, *p* < 0.01), indicating time-dependent but stable interaction with AF cells (Fig. 6H).

In parallel, in vivo live imaging was performed using the AniView multi-modal in vivo imaging system (BLT Biotech) to monitor fluorescent signal retention in disc tissues over a two-week period. DiR-labeled AF-PTK@LEV@SPP1 and @LEV@SPP1 were injected into rat IVDs, and fluorescence signals were recorded from 1 h to 14 days post-injection. While both groups exhibited comparable signal intensities at early time points (1–6 h), signal decay in the @LEV@SPP1 group was notably faster. By day 3, fluorescence from @LEV@SPP1 was nearly undetectable, whereas AF-PTK@LEV@SPP1 continued to emit detectable signals through day 14 (Fig. 6I). Statistical analysis of temporal fluorescence trends (Fig. 6J) confirmed significantly longer retention of AF-PTK@LEV@SPP1 in disc tissue compared to the untargeted control (*p* < 0.0001 at day 7 and beyond).

Together, these findings demonstrate that AF-PTK@LEV@SPP1 exhibits high AF cell specificity, prolonged binding retention, and robust in vivo targeting efficiency in both cellular and tissue contexts. The results highlight its translational promise as a precision delivery vehicle for IVD–targeted therapies.

### AF-PTK@LEV@SPP1 alleviates intervertebral disc degeneration by reducing inflammation and modulating lipid metabolism

**Fig. 7:**
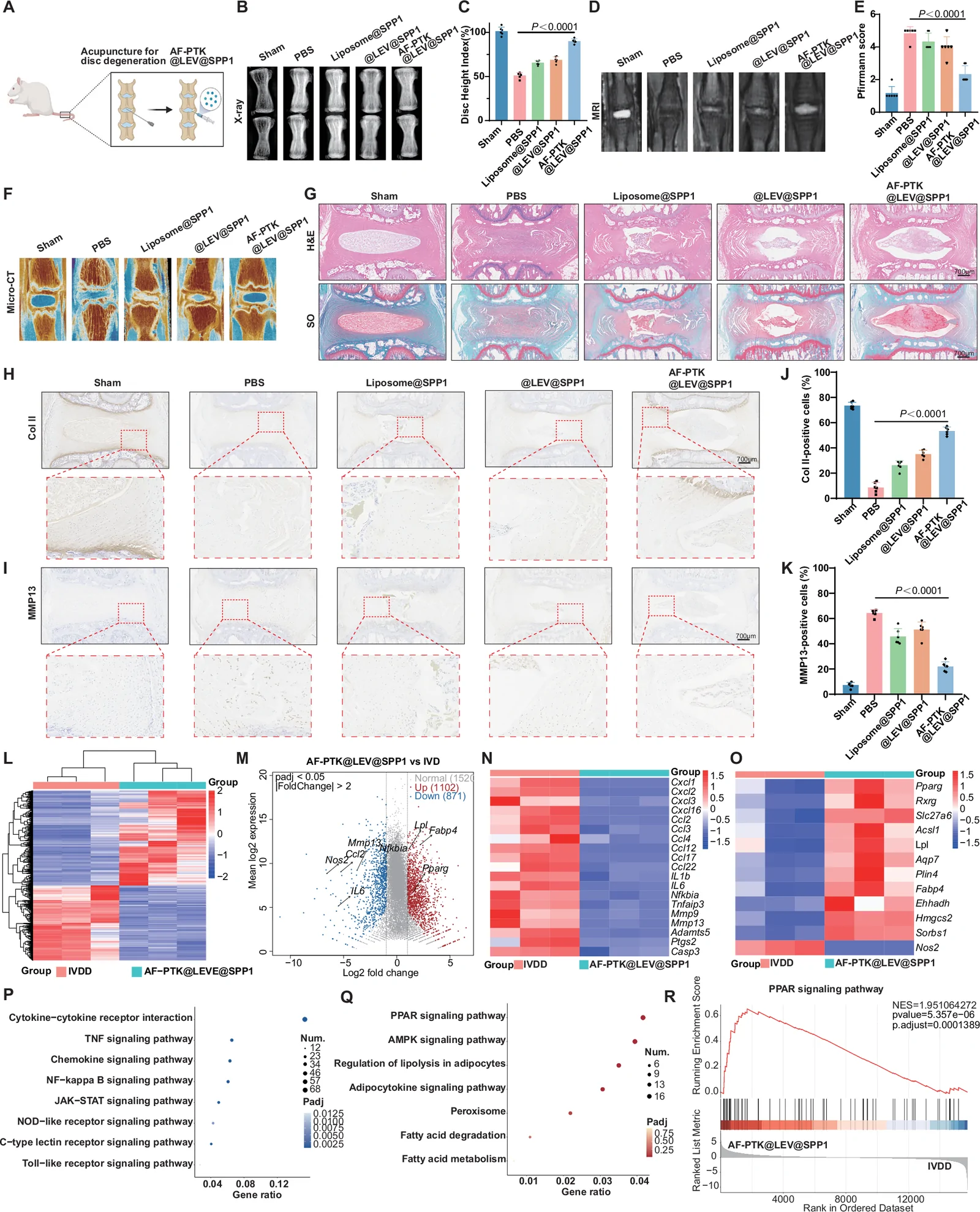
AF-PTK@LEV@SPP1 alleviates intervertebral disc degeneration by reducing inflammation and modulating lipid metabolism. **A** Schematic illustration of AF-PTK@LEV@SPP1 treatment for acupuncture-induced intervertebral disc degeneration(Created in BioRender. Chai, X. (2026) https://BioRender.com/1jbzc0w). **B** Representative X-ray images of intervertebral discs across different treatment groups after 8 weeks (Sham, PBS, Liposome@SPP1, @LEV@SPP1, AF-PTK@LEV@SPP1). **C** Quantification of disc height index from X-ray data in different treatment groups. **D** Representative MRI images of intervertebral discs in different treatment groups. **E** Quantification of Pfirrmann scores from different treatment groups indicating degeneration severity. **F** Micro-CT images showing bone architecture changes in intervertebral discs across treatment groups. **G** H&E and Safranin O/Fast Green staining of intervertebral discs at 8 weeks in different treatment groups. **H**, **I** Representative immunohistochemical staining of Col II and MMP13 in intervertebral discs from different treatment groups. **J**, **K** Quantification of Col II-positive and MMP13-positive cells in intervertebral discs. **L** Heatmap showing global mRNA expression profiles of rat intervertebral discs following acupuncture-induced degeneration and subsequent treatment with PBS (IVDD) or AF-PTK@LEV@SPP1. (*n* = 3). **M** Volcano plot illustrating significantly upregulated (red) and downregulated (blue) genes in AF-PTK@LEV@SPP1-treated discs compared with the IVDD group (|log₂FC | > 2, *p* < 0.05). Key genes involved in inflammation (Nos2, Ccl2, Il6, Mmp13) and lipid metabolism (Pparg, Fabp4, Lpl, Nfkbia) are highlighted. **N** Heatmap of inflammation- and extracellular matrix (ECM)-related genes. **O** Heatmap of lipid metabolism and PPAR signaling associated genes, indicating a shift toward anti-inflammatory and metabolic homeostasis. KEGG enrichment analysis of downregulated (**P**) and upregulated (**Q**) genes, indicating suppression of inflammatory pathways and activation of PPAR/AMPK signaling, adipocytokine signaling, and lipid metabolism. **R** GSEA confirming positive enrichment of the PPAR signaling pathway (NES = 1.95, *p* = 5.36 × 10^−6^, *q* = 1.4 × 10^−3^) in AF-PTK@LEV@SPP1-treated discs. Representative images are shown. Quantitative analyses were performed using six independent animals per group (*n* = 6). Data are presented as mean ± SD. Statistical significance was determined using one-way ANOVA followed by appropriate post hoc tests. Bulk RNA-seq analysis was performed using intervertebral disc tissues from three independent animals per group. Source data are provided as a Source Data file.

To evaluate the therapeutic efficacy of AF-PTK@LEV@SPP1 in mitigating IVD degeneration, we established a rat model of acupuncture-induced disc injury and administered nanoparticle treatment over an 8-week period (Fig. 7A). Six independent rats were included in each experimental group to ensure adequate biological replication.

Radiographic analysis revealed pronounced disc collapse and narrowing in PBS-treated animals compared with the sham group, confirming successful induction of disc degeneration. In contrast, rats treated with AF-PTK@LEV@SPP1 exhibited marked preservation of disc height and disc morphology (Fig. 7B). Quantitative analysis of the disc height index (DHI) showed a significant reduction in the PBS group (DHI ~ 50) compared with sham controls (DHI ~ 100). In contrast, AF-PTK@LEV@SPP1 treatment significantly restored the disc height (DHI ~ 90), demonstrating its therapeutic efficacy in preventing disc degeneration (Fig. 7C). Although Liposome@SPP1 and @LEV@SPP1 showed modest improvements, the AF-PTK@LEV@SPP1 group displayed the most pronounced recovery of disc height among all treatment groups.

MRI imaging further corroborated these findings. Degenerated discs in PBS-treated rats exhibited markedly reduced NP signal intensity, indicating severe dehydration and structural deterioration. By contrast, discs treated with AF-PTK@LEV@SPP1 retained higher MRI signal intensity, suggesting improved hydration and tissue integrity (Fig. 7D). Consistent with the radiographic and MRI data, Pfirrmann grading scores were significantly higher in PBS-treated discs (grade 5), indicating severe degeneration. In contrast, AF-PTK@LEV@SPP1 treatment resulted in significantly lower degeneration scores (grade 2–3), demonstrating effective protection of disc structure (Fig. 7E). Liposome@SPP1 and @LEV@SPP1 treatments produced partial improvements but remained substantially inferior to AF-PTK@LEV@SPP1.High-resolution micro-CT imaging further revealed severe osteophyte formation and endplate remodeling in PBS-treated discs. In contrast, AF-PTK@LEV@SPP1 treatment preserved vertebral endplate architecture and markedly reduced osteophyte formation (Fig. 7F), indicating improved structural stability of the IVD.

Histological examination provided additional evidence of therapeutic efficacy. H&E and Safranin O/Fast Green staining demonstrated severe disruption of the AF–NP interface and loss of proteoglycan-rich ECM in PBS-treated discs. In contrast, discs treated with AF-PTK@LEV@SPP1 retained a well-organized AF–NP structure and abundant proteoglycan deposition, closely resembling the morphology observed in the sham group (Fig. 7G). Immunohistochemical analysis further confirmed the restoration of ECM homeostasis. The percentage of COL II–positive cells, a marker of matrix synthesis, was markedly reduced in PBS-treated discs but significantly increased following AF-PTK@LEV@SPP1 treatment. Conversely, the catabolic marker MMP13 was highly expressed in degenerative discs but substantially decreased in the AF-PTK@LEV@SPP1 group. Compared with Liposome@SPP1 and @LEV@SPP1 treatments, AF-PTK@LEV@SPP1 achieved the most pronounced restoration of anabolic–catabolic balance within the disc matrix(Fig. 7H–K). Together, these in vivo imaging, histological, and molecular analyses demonstrate that AF-PTK@LEV@SPP1 effectively attenuates IVDD, preserves disc structure, and promotes ECM regeneration.

To elucidate the molecular mechanisms underlying the protective effects of AF-PTK@LEV@SPP1, we performed bulk RNA-seq on IVD tissues collected from rats treated with AF-PTK@LEV@SPP1 or PBS (IVDD group). Bulk RNA-seq was performed using disc tissues from three independent animals per group (*n* = 3) from the primary in vivo cohort.

Hierarchical clustering and global heatmap analysis revealed distinct transcriptomic profiles between groups, with extensive gene expression reprogramming in AF-PTK@LEV@SPP1-treated discs compared with the degenerative expression pattern of PBS controls (Fig. 7L). Volcano plot analysis identified 1102 genes significantly upregulated and 871 genes downregulated after AF-PTK@LEV@SPP1 treatment (Fig. 7M). Pro-inflammatory mediators such as *Nos2*, *Ccl2*, *Il6*, and *Mmp13* were markedly decreased, while metabolic and reparative regulators including *Pparg*, *Fabp4*, *Lpl*, and *Nfkbia* were upregulated, indicating a coordinated anti-inflammatory and metabolic adaptive response.

Targeted heatmaps further demonstrated two opposite gene-expression programs (Fig. 7N-O).Inflammation-related genes involved in the TNF, NF-κB, and IL-17 pathways—including *Cxcl1*, *Cxcl2*, *Cxcl3*, *Ccl2*, *Ccl3*, *Ccl4*, *Il1b*, *Il6*, *Mmp9*, *Mmp13*, *Ptgs2*, *Nfkbia*, and *Tnfaip3*—were strongly downregulated in the AF-PTK@LEV@SPP1 group (Fig. 7N).Conversely, genes associated with lipid metabolism and PPAR activation, such as *Pparg*, *Fabp4*, *Lpl*, *Acsl1*, *Aqp7*, *Hmgcs2*, *Sorbs1*, *Rxrg*, *Slc27a6*, and *Ehhadh* were significantly increased, whereas the M1-polarization marker *Nos2* was reduced (Fig. 7O). These results suggest that AF-PTK@LEV@SPP1 promotes lipid metabolic homeostasis and M2-like macrophage polarization while suppressing pro-inflammatory cascades.

KEGG pathway enrichment analysis confirmed that downregulated genes were primarily enriched in Cytokine-cytokine receptor interaction, NF-κB, TNF, Chemokine, JAK-STAT, Toll-like receptor, NOD-like receptor, and C-type lectin receptor signaling pathways (Fig. 7P).In contrast, upregulated genes were enriched in PPAR signaling, AMPK signaling, adipocytokine signaling, regulation of lipolysis in adipocytes, fatty-acid metabolism and degradation, and peroxisome pathways (Fig. 7Q).

Consistently, gene set enrichment analysis (GSEA) revealed positive enrichment of the PPAR signaling pathway (NES = 1.95, *p* = 5.36 × 10^−6^, *q* = 1.4 × 10^−3^) in the AF-PTK@LEV@SPP1 group (Fig. 7R), while Cytokine-cytokine receptor, NF-κB, and Chemokine pathways were negatively enriched (Supplementary Fig. 9A–C), confirming global suppression of inflammatory gene programs. Finally, immunohistochemical staining of ARG1 (M2 marker) and iNOS (M1 marker) further validated the in vivo macrophage polarization shift induced by AF-PTK@LEV@SPP1: ARG1 expression was elevated, whereas iNOS expression was reduced in the AF-PTK@LEV@SPP1 group compared with PBS or liposome controls (Supplementary Fig. 9D). Together, these transcriptomic and histological data demonstrate that AF-PTK@LEV@SPP1 mitigates IVDD through dual modulation of immune-inflammatory suppression and PPAR-mediated lipid metabolic activation, restoring immune-metabolic homeostasis in degenerative discs.

To further validate the safety and translational feasibility of AF-PTK@LEV@SPP1 and the targeting peptide P1, we conducted both in vivo and in vitro biosafety assessments (Supplementary Fig. 10). Histological examination of major organs-including the lung, heart, spleen, liver, and kidney-was conducted 7 days after systemic administration of AF-PTK@LEV@SPP1, @LEV@SPP1, Liposome@SPP1, PBS, or Sham (Supplementary Fig. 10A). H&E staining revealed no observable tissue abnormalities, inflammatory infiltrates, or signs of structural damage in any treatment group. The pulmonary alveolar architecture, myocardial fibers, hepatic lobular organization, renal tubules and glomeruli, and splenic parenchyma all appeared intact and comparable to controls. These findings indicate excellent systemic biocompatibility and absence of acute toxicity following administration of the nanotherapeutics.

In parallel, we evaluated the cytocompatibility of the AF-targeting peptide P1 in AF cells using a CCK-8 assay. AF cells were incubated with increasing concentrations of P1 peptide (0–20 μM) for 24 h. As shown in Supplementary Fig. 10B, no significant reduction in cell viability was observed across all tested concentrations, with viability consistently above 90%. These data demonstrate that the P1 peptide is non-cytotoxic to AF cells even at high dosages. Together, these in vivo and in vitro results confirm that both AF-PTK@LEV@SPP1 and the AF-targeting P1 peptide exhibit favorable biosafety profiles, supporting their potential for translational application in IVD regenerative therapy.

## Discussion

**Fig. 8:**
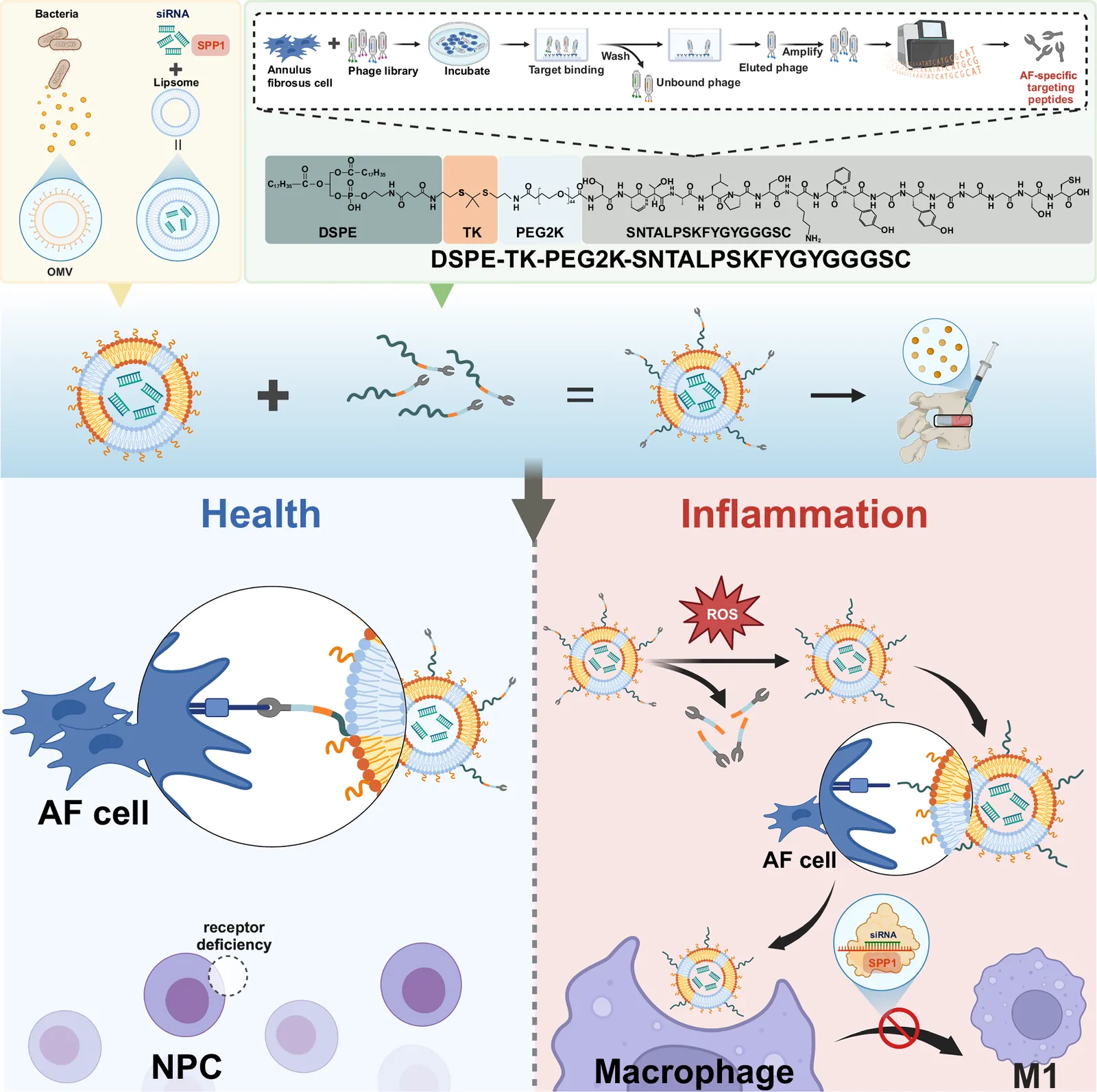
Schematic diagram of the synthesis and action of AF-PTK@LEV@SPP1. A hierarchical nanovector system anchors to annulus fibrosus cells and responds to inflammation-triggered reactive oxygen species to release therapeutic nanovesicles. These vesicles deliver small interfering ribonucleic acid to osteopontin-expressing macrophages, reduce pro-inflammatory polarization, and alleviate disc degeneration(Created in BioRender. Chai, X. (2026) https://BioRender.com/1jbzc0w).

IVDD arises from the convergence of inflammatory dysregulation, ECM breakdown, and impaired cellular resilience within an anatomically constrained, avascular microenvironment. Among disc compartments, the AF is uniquely vulnerable: its dense collagen lamellae limit molecular diffusion and immune cell egress, while mechanical overload and low regenerative capacity amplify injury cascades. Here we identify *SPP1*^*+*^ pro-inflammatory macrophages as key effectors of AF degeneration and demonstrate that *SPP1* promotes M1 polarization, NF-κB activation, and MMP-dependent catabolism, thereby coupling immune activation to matrix destruction(Fig. 8). These findings move beyond descriptive inflammation in IVDD to define an actionable macrophage pathway that can be precisely modulated in situ.

Translating this mechanism into therapy is impeded by two entrenched barriers: delivery (poor tissue penetration and retention in the AF) and specificity (dynamic, stress-triggered macrophage infiltration that renders static drug depots suboptimal). We address both with AF-PTK@LEV@SPP1, a modular nanoplatform that integrates (i) an AF peptide (P1) to achieve tissue anchoring and long-term retention, (ii) an OMV-Liposome hybrid (LEV) that confers intrinsic macrophage tropism and efficient siRNA loading, and (iii) a thioketal (PTK) linker that releases cargo under reactive oxygen species (ROS), a defining cue of degenerative niches. This spatiotemporally programmed design yields sequential behavior-initial AF localization followed by ROS-triggered liberation and macrophage uptake-enabling on-demand suppression of *SPP1* signaling where and when inflammatory stress is greatest, while minimizing off-target exposure.

Functionally, AF-PTK@LEV@SPP1 reduced M1 polarization, dampened catabolic mediators, and restored anabolic ECM markers in vitro. In co-culture, macrophage reprogramming translated into protection of AF cells, with recovery of aggrecan and collagen II and preservation of stemness-associated factors under inflammatory challenge. In vivo, the platform stabilized AF structure, preserved disc height, and improved histopathological features in a rat model of disc injury, consistent with reduced matrix catabolism and enhanced proteoglycan content. At the transcriptomic level, treatment suppressed inflammatory and chemokine axes while upregulating lipid-metabolic and PPAR-linked programs, alongside shifts in macrophage polarization markers. Together, these data support a dual mechanism of action-immune attenuation and metabolic rebalancing-that converges on restoration of disc homeostasis.

Several aspects underscore the broader significance of this work. We delineate *SPP1*-dependent macrophage pathology as a critical, targetable driver of the AF degenerative cascade, linking immune activation to ECM breakdown and cell dysfunction. We then establish a potentially generalizable design principle for avascular, fibrous tissues: combine tissue anchoring with pathology-cue responsiveness to achieve confined and timed immunomodulation. Finally, the modular structure of AF-PTK@LEV@SPP1 allows the targeting ligand, linker chemistry, and nucleic acid payload to be readily adapted for other inflammatory mediators or pathological contexts, including fibrosis and mechanically stressed tissues beyond the spine.

Safety is an essential consideration for therapeutic development in a load-bearing and anatomically sensitive organ. The AF-homing peptide demonstrated minimal cytotoxicity, and the use of detoxified OMVs (ΔmsbB) preserved macrophage specificity while reducing LPS-related immunogenicity. Systemic administration did not cause detectable histopathological abnormalities in major organs, and the ROS-responsive release mechanism inherently limits systemic exposure. These properties distinguish the present nanoplatform from conventional liposomes or drug depots that often suffer from rapid clearance or nonspecific release.

The work also raises hypotheses that merit deeper exploration. The transcriptomic signature suggests that *SPP1* silencing intersects macrophage metabolism, with PPAR-linked lipid pathways emerging as candidate co-regulators of polarization and tissue repair. Pharmacological or genetic dissection of these axes—potentially in combination with *SPP1* targeting—may yield synthetic benefits for immune remodeling. Moreover, temporal control likely matters: macrophage infiltration into the AF is stress-triggered and time-dependent, implying that dosing schedules aligned to inflammatory peaks could further enhance efficacy.

Limitations should be acknowledged. Rodent injury models recapitulate major features of human AF degeneration but cannot fully model chronic biomechanics or the heterogeneity of human disease. Larger animals and human organotypic or ex vivo cultured discs will be important to validate biodistribution, pharmacokinetics/clearance, and long-term structural repair. While we confirmed ROS-responsive release and AF-specific retention, future studies should quantify intradiscal release kinetics, the lifetime of the linker under physiological ROS, and repeat-dose effects. Additional work is also needed to define the manufacturability, batch-to-batch consistency, and acceptable endotoxin levels of OMV-derived materials, which will be essential for advancing this platform toward scalable and clinically relevant production.

In summary, we establish *SPP1*-mediated macrophage activation as a mechanistic driver of AF degeneration and present AF-PTK@LEV@SPP1 as a microenvironment-activated, macrophage-targeted therapy that reshapes the inflammatory landscape of the degenerating disc and promotes matrix repair. By integrating nanotechnology, immunomodulation, and disc biology, this study provides a path toward disease-modifying treatment in IVDD and a blueprint for confined immune engineering in other anatomically constrained, inflammatory tissues.

## Methods

### Ethics statement

All animal experiments were conducted in accordance with the Guide for the Care and Use of Laboratory Animals and were approved by the Institutional Animal Care and Use Committee of the Second Affiliated Hospital of Zhejiang University School of Medicine (approval No. 2024-351). This study is reported in accordance with ARRIVE guidelines. All procedures were performed under anesthesia, and efforts were made to minimize animal suffering. Animal welfare was monitored daily. Euthanasia was performed under deep anesthesia induced by an overdose of sodium pentobarbital (150 mg/kg, intraperitoneal injection).

Human IVD samples were collected with approval from the Institutional Review Board of the Second Affiliated Hospital of Zhejiang University (approval No. 2024SQXB002446). Written informed consent was obtained from all participants prior to sample collection. All procedures involving human samples were conducted in accordance with the Declaration of Helsinki.

### Animals

C57BL/6J mice (8 weeks old), Sprague-Dawley rats (male, 8–12 weeks old), and Lyz2-Cre;DTR mice (8-10 weeks old) were used in this study. C57BL/6J mice and Sprague-Dawley rats were purchased from Shanghai SLAC Laboratory Animal Co., Ltd. (Shanghai, China). Lyz2-Cre;DTR mice were generated in-house by crossing Lyz2-Cre transgenic mice with DTR-floxed mice. All animals were housed under specific pathogen-free conditions in a controlled environment (22 ± 1 °C, 50 ± 10% humidity, 12 h light/dark cycle) with ad libitum access to standard laboratory chow and autoclaved water. The number of animals used in each experiment is indicated in the corresponding figure legends. Both male and female mice were included, whereas all rats were male. Sex was not considered a biological variable, and no analyses were performed to assess sex differences. The number of animals used in each experiment is indicated in the corresponding figure legends.

### Materials

Hydrogenated soybean phosphatidylcholine (HSPC), DSPE-MPEG2000, and cholesterol (CHO-HP) were purchased from Bio-Tech (Shanghai, China) and used as received without further purification. Analytical-grade organic solvents, including chloroform and methanol, were obtained from Shanghai Lingfeng Chemical Reagent Co., Ltd. The siRNA targeting mouse SPP1 (siRNA-SPP1) was synthesized by Shanghai Jiman Technology Co., Ltd. All aqueous solutions were prepared using ultrapure water (Milli-Q system, Millipore, resistivity ≥18.2 MΩ cm). The Enhanced BCA Protein Assay Kit (P0010S), 4′,6-diamidino-2-phenylindole (DAPI, C1006), DiR (C1991), DiO(C1992), and Cell Counting Kit-8 (C0037) were purchased from Beyotime Biotechnology (Shanghai, China). All other chemical reagents were sourced from the Zhejiang University Reagent Platform.

### Preparation of siRNA-loaded liposomes

siRNA-loaded liposomes were prepared using a thin-film hydration method followed by probe sonication to achieve size reduction and efficient siRNA encapsulation. Briefly, a lipid mixture consisting of DSPC, cholesterol, and DSPE-PEG2000 in a molar ratio of 4:4:1 was dissolved in chloroform. The solvent was evaporated under reduced pressure at 40 °C using a rotary evaporator to form a uniform thin lipid film, which was further dried under vacuum for 2 h to remove residual solvent completely. The dried lipid film was hydrated with siRNA-SPP1 solution (10 mM citrate buffer, pH 4.0) at 37 °C with gentle vortexing for 5 min to form multilamellar vesicles. The suspension was then probe-sonicated (Branson Ultrasonics, 3 mm probe) in an ice bath for 10 min (40% amplitude, pulse mode: 5 s on/5 s off) to reduce particle size and promote siRNA encapsulation. Unencapsulated siRNA was removed by centrifugation at 10,000 × *g* for 10 min at 4 °C and further purified through a Sephadex G-50 gel filtration column pre-equilibrated with PBS (pH 7.4). The purified siRNA-loaded liposomes were concentrated using an Amicon Ultra-15 centrifugal filter device (10 kDa cutoff, Millipore) and stored at 4 °C until use.

### Bacterial culture and collection of extracellular vesicles (EVs)

Extracellular vesicles (EVs), primarily OMVs, were isolated from *Escherichia coli* using differential centrifugation under sterile conditions. The E. coli BL21(DE3)-msbB knockout strain (catalog number: EC1001) was obtained from the Zhejiang University Chemical Reagent Platform. Bacteria were streaked onto solid Luria–Bertani (LB) agar plates and cultured overnight at 37 °C. A single colony was then inoculated into liquid LB medium and incubated at 37 °C with shaking (180 rpm) for 6 h. The culture was then diluted 1:100 into fresh LB medium and grown until the optical density at 600 nm (OD600) reached approximately 1.0, corresponding to the mid-logarithmic growth phase.

For EV collection, 500 mL of bacterial culture was centrifuged at 5000 × *g* for 30 min at 4 °C to pellet the cells. The supernatant was filtered through a 0.45 μm polyethersulfone membrane (Millipore, catalog number: SHLP033RB) to remove residual bacteria. The filtrate was concentrated using a 100 kDa molecular weight cutoff centrifugal filter unit (Millipore, catalog number: UFC910096). The concentrated medium was then ultracentrifuged at 200,000 × *g* for 2 h at 4 °C (Beckman Coulter XPN-100). The resulting pellet was resuspended in sterile PBS and ultracentrifuged again under the same conditions to further purify the EVs. The final pellet was resuspended in sterile PBS to obtain the EV stock solution. To confirm sterility, an aliquot of the EV preparation was plated on LB agar plates. The protein concentration of the EV preparation, used to quantify EV yield, was measured using an Enhanced BCA Protein Assay Kit.

### Fusion of siRNA-loaded liposomes and EVs

To prepare membrane-fusogenic hybrid nanoparticles, siRNA-loaded liposomes and bacterial EVs were mixed at a mass ratio of 3:1 (liposomes: EVs) under sterile conditions. The mixture was subjected to probe sonication on ice for 10 min (30% amplitude, pulse mode: 5 s on/5 s off) to promote efficient membrane fusion. After sonication, the hybrid vesicle suspension was centrifuged at 10,000 × *g* for 10 min at 4 °C to remove aggregates and debris. The resulting supernatant was then subjected to ultracentrifugation at 100,000 × *g* for 1 h at 4 °C, which served as the final purification step for all experimental applications. The resulting pellet was resuspended in sterile PBS and stored at 4 °C for immediate use.

### Characterization of AF-PTK@LEV@SPP1

AF-PTK@LEV@SPP1 and siRNA-loaded liposomes were characterized for particle size, PDI, and zeta potential using DLS (Zetasizer Nano ZS, Malvern Instruments). Morphological evaluation was performed by transmission electron microscopy (TEM) after negative staining with 2% phosphotungstic acid.

### Encapsulation efficiency (EE) and morphology

The encapsulation efficiency was determined by measuring the unencapsulated siRNA at 260 nm using a NanoDrop spectrophotometer. The calculation was performed as follows:

$${{{\rm{EE}}}}(\%)=(1-({{{\rm{Free\; siRNA}}}}/{{{\rm{Total\; siRNA}}}}))\times 100$$

The morphology and size distribution of the liposomes were examined using TEM after negative staining with 2% phosphotungstic acid.

### ROS-responsive siRNA release assay

AF-PTK@LEV@SPP1 nanoparticles were incubated in PBS with or without 100 μM hydrogen peroxide (H₂O₂) at 37 °C. At predetermined time points (0, 2, 4, 6, 8, 12, 24, 36, 48, 60, and 72 h), supernatants were collected after centrifugation (10,000 × *g*, 10 min), and released siRNA was quantified by measuring absorbance at 260 nm. Release efficiency was calculated as the cumulative percentage relative to the total siRNA load.

### Cell culture

The AF tissue was isolated from the IVDs of 8-week-old SD rats (*n* = 2 donors) and minced into a paste before being digested with 0.1% collagenase I (Gibco, Shanghai, China) at 37 °C for 40 min. The digestion was halted by adding Dulbecco’s modified Eagle’s medium (DMEM/F-12, Gibco). The mixture was centrifuged at 1000 × *g* for 5 min, and the resulting pellet was cultured in DMEM/F-12 in a humidified incubator at 37 °C with 5% CO2. The culture medium was replenished every other day for 7 days, after which it was changed every two days, and cells were passaged once they reached 80% confluence. The third passage of AF cells was used for subsequent experiments, where 5 nM IL-1β was added to the culture for 24 h to induce degeneration.

Primary bone marrow–derived macrophages (BMDMs) were isolated from the femurs and tibias of 8-week-old C57BL/6 mice. Bone marrow cells were flushed out with sterile PBS, filtered through a 70 μm cell strainer, and cultured in RPMI-1640 medium supplemented with 10% fetal bovine serum (FBS) and 20 ng/mL recombinant murine M-CSF for 7 days to promote macrophage differentiation. Differentiated BMDMs were transfected with three siRNA constructs targeting mouse *Spp1* (siRNA-*Spp1*-si-1, si-2, si-3; synthesized by Shanghai Jiman Technology Co., Ltd.) using jetPRIME® Transfection Reagent (Polyplus-transfection, Ref Lot#0000003878) according to the manufacturer’s instructions. After 48 h of transfection, cells were harvested for qRT-PCR analysis to determine knockdown efficiency, and the most effective siRNA (si-1) was selected for subsequent experiments. For M1 polarization, BMDMs transfected with *Spp1* si-1 were stimulated with lipopolysaccharide (LPS, 100 ng/mL) for 18 h. Macrophage phenotype was assessed by flow cytometry using antibodies against CD86 and iNOS. The percentage of CD86⁺iNOS⁺ cells was used as a measure of pro-inflammatory activation. All animal procedures for BMDM isolation were approved by the Institutional Animal Care and Use Committee of the Second Affiliated Hospital of Zhejiang University.

### Flow cytometry for macrophage phenotyping

Differentiated BMDMs were harvested and washed with cold PBS. To block Fc receptors, cells were pre-incubated with anti-mouse CD16/32 (TruStain FcX™, BioLegend, CAT: #101319) for 10 min at 4 °C. Cells were then stained with Zombie Aqua Fixable Viability Kit (BioLegend) to exclude dead cells, following the manufacturer’s protocol. For surface marker staining, cells were incubated with fluorophore-conjugated anti-CD86 antibody (BioLegend) for 30 min at 4 °C in the dark. For intracellular staining of iNOS, cells were fixed and permeabilized using the True-Nuclear™ Transcription Factor Buffer Set (BioLegend, CAT: #424401), then incubated with anti-iNOS antibody (BioLegend) for 20 min at room temperature in the dark. After final washes in staining buffer (PBS with 2% FBS), samples were analyzed on a Beckman Coulter CytoFLEX LX flow cytometer using CytExpert v2.4 software. Data were processed and gated using FlowJo v10 to quantify CD86⁺iNOS⁺ macrophage populations.

### Phage display

A linear 12-amino acid peptide library (Ph.D.−12 Phage Display Peptide Library, New England Biolabs, Inc.) was utilized for bio-panning to identify peptides with specific binding affinity. Initially, 1 × 10¹² transduction units of the phage library were incubated with mouse primary fibroblasts for negative selection. The supernatant containing unbound phages was then collected and added to AF cells for positive selection. After a 1-h incubation at 37 °C, the cells were thoroughly washed five times with washing buffer (composition and pH of buffer can be specified here) to remove any non-specifically bound phages. Phages bound to the cells were subsequently eluted using an elution buffer (specify composition, pH, or concentration of buffer if necessary). The eluted phages were then amplified by infection of the Escherichia coli host strain ER2738, following the manufacturer’s protocol. The resulting phage population was used for the subsequent round of selection. This process of negative and positive selection was repeated for a total of four rounds to enrich for phages displaying peptides with higher affinity and specificity for the target cells. For each round, the phage titers and the number of plaques were monitored, and a portion of the phages from each round was used for further characterization and sequencing.

### Peptide synthesis and dye labeling

A peptide sequence consisting of three glycine (G), one serine (S), and one cysteine (C) (GGGSC) was added to the C-terminus of the peptide as a linker region. This peptide was synthesized by KS-V Peptide Biotechnology Co., Ltd. (Hefei, China). The Cy5.5 dye labeling was performed based on our previous work. Briefly, 1 × 10⁻³ M Cy5.5-maleimide was mixed with 1 × 10⁻³ M peptide in saline at a 1:4 ratio, and the mixture was stirred at room temperature for 3 h. Unconjugated peptide was removed using a Slide-A-Lyzer dialysis device (1000 MWCO, Thermo Fisher Scientific, Waltham, MA, USA). The palmitoylated peptide was synthesized by KS-V Peptide Biotechnology Co., Ltd. (Hefei, China) using the Fmoc/tBu solid-phase peptide synthesis strategy. Palmitic acid was conjugated to the ε-amino group of the first lysine residue at the C-terminus.

### Lyz2-Cre;DTR mice

Animal experiments were conducted using Lyz2-Cre;DTR mice (*n* = 3 per group), which were generated by breeding Lyz2-Cre transgenic mice with DTR (diphtheria toxin receptor) floxed mice. The Lyz2-Cre;DTR strain allows for specific deletion or ablation of macrophages or other myeloid cells through Cre-LoxP-mediated recombination. To induce cell ablation in Lyz2-Cre;DTR mice, DT was administered at a dose of 25 μg/kg by intraperitoneal injection on Day 4 and repeated at regular intervals (if applicable). Control mice (DTR floxed without Cre expression) received the same DT injection to rule out off-target effects. All mice were bred and maintained under specific pathogen-free (SPF) conditions in an environment with constant temperature (23–25 °C), controlled humidity (50%), and a 12-h light/12-h dark circadian cycle. Animals had free access to standard chow and water. All experimental protocols were approved by the Institutional Animal Care and Use Committee (IACUC) at Second Affiliated Hospital of Zhejiang University and complied with ethical guidelines for the care and use of laboratory animals.

### Mouse genotyping and transgene validation

#### Genomic DNA extraction and genotyping

Genomic DNA was extracted from mouse tail, toe, or ear tissue using a one-step genomic DNA extraction kit (Cat# K9088, Magen Biotech, China) according to the manufacturer’s protocol. Briefly, 10–50 mg of tissue was incubated with 150 μL Buffer L88-Plus and 5 μL Proteinase K at 60 °C until complete lysis (typically ≥4 h or overnight). After digestion, 300 μL of Buffer N2 (pre-mixed with Buffer N1) was added and mixed thoroughly to neutralize the reaction. The lysate was centrifuged briefly, and 3 μL of the supernatant was used for downstream PCR analysis. For samples requiring high-purity DNA (e.g., qPCR or difficult-to-amplify genotypes), DNA was extracted using a column-based purification kit (Cat# K9053, Magen Biotech). Tissues were digested in Buffer L and Proteinase K, followed by binding to a silica column, ethanol-based washing (Buffer W2), and elution with pre-warmed Buffer E at 65 °C. DNA quality was assessed via 1% agarose gel electrophoresis.

#### PCR reaction setup

Standard PCR was performed in 25 μL total volume containing 12.5 μL 2× Taq Master Mix (Cat# C7014, Magen Biotech), 0.5 μL of each primer (10 μM), DNA template (100 ng–1 μg), and nuclease-free water. Thermal cycling conditions were as follows: 95 °C for 5 min; 35–40 cycles of 95 °C for 30 s, 58 °C for 30 s, and 72 °C for 60 s per kb; with a final extension at 72 °C for 7 min. qPCR genotyping was carried out using 2× Probe qPCR Mix (Cat# C7018, Magen Biotech) in a 20 μL reaction system, including 0.8 μL primers (10 μM), 0.3 μL probes (10 μM), template DNA, and nuclease-free water. Fluorescence was detected during the annealing-extension phase (60 °C for 30 s) over 35–40 cycles, following denaturation at 95 °C for 10 s.

#### PCR product detection

Amplicons were resolved by 1–2% agarose gel electrophoresis at 120 V for 20–30 min and visualized under UV illumination using a nucleic acid dye. Genotypes were interpreted by comparing band sizes to molecular weight markers (DL2000, D50, or Marker III, Magen Biotech). For ambiguous cases or SNP variants, Sanger sequencing was performed for confirmation.

### Single-cell sequencing methodology

To investigate the cellular composition and signaling pathways involved in degenerated IVD, clinical samples from patients with severe age-related disc degeneration were collected. The IVD tissue samples were dissociated into a single-cell suspension using a mechanical dissociation method followed by enzymatic digestion with 0.1% collagenase I (Gibco, USA) at 37 °C for 30 min. The resulting cell suspension was filtered through a 40 µm cell strainer (Thermo Fisher Scientific) to remove debris and obtain a single-cell population. scRNA-seq was performed using the 10x Genomics Chromium Single-Cell 3’ Reagent Kit (10x Genomics, USA) following the manufacturer’s protocol. Briefly, cells were loaded onto the Chromium Controller (10x Genomics) to generate single-cell Gel Beads-in-Emulsion (GEMs). cDNA synthesis was performed in the GEMs, followed by amplification of the cDNA and purification. Libraries for sequencing were constructed using the Nextera XT DNA Library Preparation Kit (Illumina, USA), with final library quality assessed using the Agilent 2100 Bioanalyzer (Agilent Technologies, USA). Sequencing was conducted on the Illumina NovaSeq 6000 platform (Illumina, USA) to generate 150 bp paired-end reads. The raw sequencing data were processed using Cell Ranger (10x Genomics) software for read alignment, barcode identification, and unique molecular identifier (UMI) counting. The gene expression matrices were further analyzed using Seurat (v4.0, R) to identify distinct cell clusters, perform differential gene expression analysis, and explore cellular infiltration patterns within the degenerated IVD tissue by LC-Bio Technology, Ltd, Hangzhou. For the investigation of SPP1-related signaling pathways, pathway analysis was conducted using GSEA and Ingenuity Pathway Analysis to identify enriched pathways and biological processes associated with SPP1 expression. The analysis provided insight into the molecular mechanisms underlying IVDD, with a focus on cellular infiltration and the role of SPP1 in the degenerative process.

### Establishment of mice intervertebral disc degeneration model

Two-month-old C57/BL6J mice were used in this study to establish an IVD degeneration model by simulating the changes in stress caused by disc compression using the “tail-looping” procedure. All surgical instruments were sterilized by autoclaving. Mice were anesthetized by intraperitoneal injection of 1% sodium pentobarbital (40–50 mg/kg). Following anesthesia and disinfection, a hemostatic tourniquet was applied to the proximal end of the tail to ensure proper exposure of the surgical site. To induce IVDD, the “tail-looping” method was employed, which is a widely used model for IVD degeneration, as shown in the basic schematic in Supplementary Fig. 2F. The tail was looped around the fixed position between the L5 and L13 vertebrae, and a 0.8-mm stainless steel wire was used to secure the loop in place. The distal portion of the tail was then excised to simulate disc compression. This method provides relatively consistent compression across all mice, with minimal surgical variation, ensuring reproducible IVD degeneration. The severity of degeneration could be adjusted by altering the compression cycle or adding additional NP aspiration. Post-surgery, mice were kept warm and closely monitored during the recovery period to ensure smooth anesthesia recovery.

### Establishment of intervertebral disc injury model in rats

Twelve-week-old male Sprague–Dawley rats were used to establish an IVDD model. Under general anesthesia with pentobarbital sodium (50 mg/kg, i.p.), a needle puncture-induced degeneration procedure was performed at the coccygeal Co6–Co7 IVD. A sterile 18 G needle was percutaneously inserted into the disc, rotated 360°, and held in place for 30 s to induce mechanical injury. This procedure has been widely validated for creating consistent and reproducible disc degeneration in rodent models. All surgical instruments were autoclaved, and the procedures were conducted under sterile conditions.

### RNA sequencing

For bulk RNA sequencing, IVD tissues were collected from three independent animals per group. Total RNA was extracted from samples using the conventional TRIzol method, purified by column, and reverse transcribed to construct the cDNA library of mRNA according to the RNA molecular weight. Then, transcriptome sequencing was performed using ILlumina HiSeq 2500 technology. The data were compared with the reference genome and annotated with genes to obtain the RNA expression matrix for differential expression analysis, gene interaction network analysis, transcription factor binding site analysis, WGCNA\GSEA analysis, and Reactome protein function enrichment analysis.

### Compound administration protocol

To evaluate the therapeutic effects of AF-PTK@LEV@SPP1, a targeting peptide-loaded nanoparticle formulation, in vivo, rats were anesthetized with pentobarbital sodium (50 mg/kg, i.p.) and placed in a prone position. A 33 G Hamilton microsyringe was used to deliver the nanoparticle suspension via percutaneous intradiscal injection into the Co6–Co7 IVD under fluoroscopic guidance. Each rat received a 10 μL injection of the nanoparticle suspension (equivalent to 1 mg/kg siRNA-*Spp1*). The injection was delivered slowly over 30 s to ensure even distribution and minimize leakage. Injections were initiated 24 h post-surgery. Control groups received 10 μL of PBS or empty liposome formulation under identical conditions. All procedures were performed under sterile conditions and approved by the Institutional Animal Care and Use Committee of the Second Affiliated Hospital of Zhejiang University.

### Imaging assessment of intervertebral disc height

X-ray images of the IVDs at 8 weeks of age were obtained using a molybdenum target X-ray machine (MCR-6000, China). The IVD height was measured using ImageJ software (National Institutes of Health, USA). The therapeutic effects of AF-PTK@LEV@SPP1 on IVDD were evaluated using micro-CT and MRI at 8 weeks post-surgery. Briefly, rats were euthanized using an overdose of sodium pentobarbital, and their coccygeal vertebrae specimens were collected. The IVDs at Co6/Co7 were dissected and fixed in 4% paraformaldehyde. Microstructural evaluation of the vertebrae and cartilaginous endplates was performed using micro-computed tomography (μCT, Bruker, USA) with a resolution of 12 μm, 50 kVp, and 145 μA. Three-dimensional image reconstruction and data analysis were conducted using the instrument’s built-in software. MRI was performed with the BioSpec 7 T system (Bruker, USA), capturing T2-weighted images in the coronal plane. MRI grading for each group was assessed based on the Pfirrmann grading system.

### Histological staining and immunohistochemistry

Tissues were collected 8 weeks post-surgery from the tail vertebrae, fixed in 4% paraformaldehyde for 24 h, and decalcified in 10% EDTA for 4 weeks (Biosharp, China). After decalcification, tissues were dehydrated and embedded in paraffin. Sections were cut to 4 μm thickness and stained with H&E and Safranin O-Fast Green (SO) for histological analysis. SO staining was used to assess the severity of IVDD. The groups were assigned as follows: Sham (high positive), PBS (low positive), Liposome@SPP1 (low positive), @LEV@SPP1 (low positive), and AF-PTK@LEV@SPP1 (positive). For immunohistochemistry, Col II and MMP13 were used as markers of IVDD. Samples were treated with 3% H2O2 for 10 min, blocked with 5% BSA for 30 min, and incubated overnight at 4 °C with Col II and MMP13 antibodies. After washing with PBS, samples were incubated with a biotin-labeled secondary antibody at 37 °C for 30 min. Staining was visualized using the SABC method. Images were captured under a microscope, and the percentage of positive-staining cells was calculated using Image Pro Plus 6.0 software.

### Histological grading of disc degeneration

Histological evaluation was conducted based on a modified Masuda scoring system. Three independent regions per section were assessed by two blinded investigators, scoring AF structure, NP matrix homogeneity, and cellularity. Each component was scored from 0 (normal) to 3 (severe degeneration), and the total score was used for comparison. Mean scores were calculated for each sample and analyzed using one-way ANOVA with Tukey’s post-hoc test.

### Western blotting

Proteins were extracted from cell samples using moderate-strength RIPA buffer (BOSTER, China) supplemented with a proteasome inhibitor. Proteins were then separated by 10% SDS-PAGE and transferred onto a polyvinylidene fluoride membrane (Millipore, Shanghai, China). The membrane was blocked for 1 h with 10% non-fat milk, followed by three washes with Tris-buffered saline with 0.1% Tween-20 (TBST). The membrane was incubated overnight at 4 °C with primary antibodies. β-actin was used as an internal control. After washing off excess primary antibodies with TBST, the membrane was incubated with a horseradish peroxidase-conjugated secondary antibody (Beyotime, A0225, 1:2000) at room temperature for 1 h. Immunoreactive bands were visualized using a ChemiDoc Touch Imaging System (Bio-Rad), and signal intensity was measured.

### PCR

Total RNA was extracted from cells and tissue samples using the RNeasy Mini kit (Qiagen) following the manufacturer’s instructions. The quality and concentration of the RNA were assessed using a NanoDrop spectrophotometer (Thermo Fisher Scientific). Reverse transcription was performed using the High-Capacity cDNA Reverse Transcription Kit (Applied Biosystems) according to the manufacturer’s protocol. Quantitative PCR (qPCR) was conducted using SYBR Green real-time PCR (Applied Biosystems) to analyze gene expression. Each PCR reaction was performed in a 20 µL volume containing 10 µL of SYBR Green PCR Master Mix, 1 µL of cDNA template (diluted to 100 ng/µL), 0.4 µL of each forward and reverse primer (final concentration 200 nM), and 8.2 µL of nuclease-free water. The primers used for specific gene amplification were designed using Primer3 software, and their sequences are provided in the supplementary materials. The qPCR was performed in a 96-well plate using the QuantStudio 3 Real-Time PCR System (Applied Biosystems). The thermal cycling conditions included an initial denaturation step at 95 °C for 10 min, followed by 40 cycles of denaturation at 95 °C for 15 s, annealing at the primer-specific temperature (usually 60 °C) for 30 s, and extension at 72 °C for 30 s. A melting curve analysis was performed to confirm the specificity of the amplification products. The relative gene expression was calculated using the ΔΔCt method, with β-actin as the reference gene. All samples were analyzed in triplicate.

### Enzyme-linked immunosorbent assay (ELISA)

Cytokine levels in the culture supernatants of bone marrow–derived macrophages were determined using commercial ELISA kits for TNF-α, IL-6, and IL-10 (Jianglai Bio, China) following the manufacturer’s protocol. After treatment with LPS + IFN-γ (M1 induction) or IL-4 + IL-13 (M2 induction), with or without recombinant SPP1 or AF-PTK@LEV@SPP1, culture media were collected, centrifuged to remove debris, and stored at −80 °C until use. Samples and standards were added to 96-well plates in duplicate, incubated with biotin-conjugated detection antibody and streptavidin-HRP, and developed with TMB substrate. Absorbance was measured at 450 nm using a microplate reader, and cytokine concentrations were calculated from standard curves fitted by a four-parameter logistic model. Data are presented as mean ± SD (*n* = 3 biological replicates) and analyzed by two-tailed Student’s *t* test, with *p* < 0.05 considered statistically significant.

### Antibody

#### Western blot

Aggrecan(ABclonal,A8536,1:500),MMP13(abcam,ab39012,1:2000),ADAMTS5(ABclonal,A23125,1:2000),Collagen II(Proteintech,28459-1-AP,1:500),SOX2(ABclonal,A0561,1:500),OCT4(ABclonal,A25315,1:1000),c-MYC(ABclonal,A19032,1:1000),STAT1(Affinity,AF6300,1:500),STAT6(Affinity,AF6302,1:500),Phospho-STAT1(Affinity,AF3300,1:500),Phospho-STAT6(Affinity,AF3301,1:500),ARG1(Affinity,DF6657,1:500),iNOS(Affinity,AF0199,1:500),CD206(Affinity,DF4149,1:500),CD86(Affinity,DF6332,1:500),p65(Affinity,AF5006,1:500),Phospho-p65 (Affinity,AF3389,1:500),β-actin (Abcam,ab8227,1:1000)

#### Immunohistochemistry

Col II (Abcam,ab307674,1:100), MMP13 (Affinity,AF5355,1:100),ARG1(Affinity,DF6657,1:100),iNOS(Affinity,AF0199,1:100)

### Statistical analysis

The letter “*n*” denotes the number of biologically independent samples. Sample sizes, statistical tests, and main effects are provided in the figure legends. Data are presented as mean ± standard deviation (Mean ± SD). Comparisons between two groups were performed using an independent samples t-test or Wilcoxon rank-sum test, depending on data distribution. For multiple-group comparisons, one-way ANOVA with post hoc Tukey or Dunnett’s tests was applied. Analyses were conducted using GraphPad Prism v.10.0, and exact *P* values are indicated in the figures. Statistical significance was set at *P* < 0.05.

### Reporting summary

Further information on research design is available in the Nature Portfolio Reporting Summary linked to this article.

## Supplementary information

Supplementary information

Peer Review file

Reporting summary

## Source data

Source Data

## Data Availability

The original contributions presented in this study are included in the article and its Supplementary Material. Further inquiries can be directed to the corresponding authors. The raw single-cell RNA sequencing data generated in this study have been deposited in the Genome Sequence Archive for Human (GSA-Human) under accession codes HRA010009 and HRA018950. The processed single-cell RNA-seq expression matrix files have been deposited in OMIX under accession code OMIX017533. The bulk RNA sequencing data generated in this study have been deposited in the NCBI Gene Expression Omnibus (GEO) under accession code GSE289741. These datasets are publicly available. Source data are provided with this paper.
