# Supplementary material for: An annulus fibrosus anchored nanovector reprograms osteopontin driven macrophages in preclinical disc degeneration models
Source: Nat Commun. 2026 Jul 21;17:8887. doi: 10.1038/s41467-026-75720-3 (PMC13500848; doi:10.1038/s41467-026-75720-3)
Supplement: Supplementary file 1 [file 41467_2026_75720_MOESM1_ESM.pdf]

Supplementary Information for "An annulus fibrosus anchored nanovector reprograms osteopontin driven macrophages in preclinical disc degeneration models"

Zilong Li[1,2,3,4,5#], Yucheng Xue[1,2,3,4,5#], Zhenyu Li[6#], Miaojie Fang[7], Yiwen Xu[1,2,3,4,5], Kelei Wang[1,2,3,4,5], Hong Liu[2,3,4,5,8], Zhenzhong Chen[2,3,4,5,9], Jianbin Xu[1,2,3,4,5*], Ning Zhang[1,2,3,4,5*]

1.Department of Orthopedic Surgery, The Second Affiliated Hospital, Zhejiang University School of Medicine, Hangzhou 310009, China

2.Orthopedics Research Institute of Zhejiang University, Hangzhou 310009, China

3.Key Laboratory of Motor System Disease Research and Precision Therapy of Zhejiang Province, Hangzhou 310009, China

4.Zhejiang Provincial Clinical Medical Research Center for Motor System Diseases, Hangzhou 310009, China

5.International Chinese Musculoskeletal Research Society, Hangzhou 310009, China

6.University of California, Santa Barbara, California 93106, United States

7.Department of Orthopedics,The Fourth Affiliated Hospital, Zhejiang University School of Medicine, Yiwu 322000, China

8.Department of Orthopedics, Jiangnan Hospital, Zhejiang Chinese Medical University 310053, Hangzhou, China

9.Department of Orthopedics, Lishui Municipal Central Hospital, Lishui,323000, China

*Corresponding author.Email:Ning Zhang,zhangning@zju.edu.cn;Jianbin Xu,xu9709426@zju.edu.cn

#These authors contributed equally

* These authors jointly supervised this work

Supplementary Fig. 1. MRI imaging and clinical metadata of lumbar disc degeneration patients used for single-cell RNA sequencing.

A

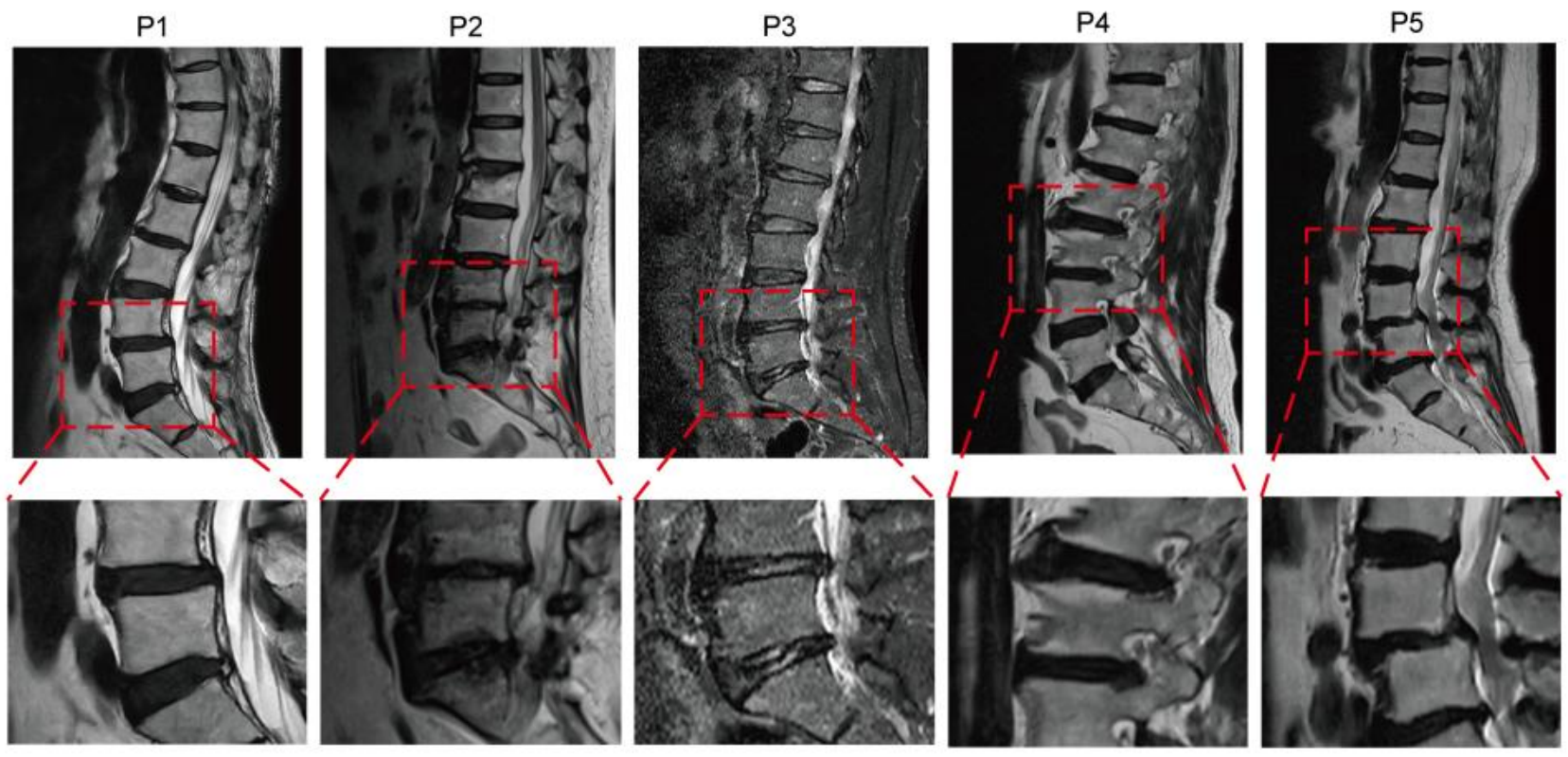

B

Patient	Sex	Age	Diagnosis	Clinical Treatment (Surgery Type)	Tissue Site	Pfirrmann grade
P1	Male	70	Lumbar spondylolisthesis, fracture	Posterior lumbar interbody fusion (L4/5), pedicle screw fixation	L4/5 IVD	IV
P2	Female	78	Lumbar stenosis & herniation	Lateral lumbar fusion and discectomy (L4/5, L5/S1)	L4/5, L5/S1 IVD	IV
P3	Male	63	Lumbar stenosis	Posterior lumbar fusion(L4/5,L5/S1)	L4/5 ,L5/S1 IVD	III
P4	Male	76	Lumbar spinal stenosis	Lateral lumbar interbody fusion (L3/4, L4/5), pedicle screw fixation	L3/4, L4/5 IVD	IV
P5	Female	67	Multilevel disc herniation and stenosis	Posterior fusion and decompression (L4/5)	L4/5 IVD	IV

(A) Sagittal T2-weighted MRI scans of five patients (P1-P5) with lumbar disc degeneration. Red dashed boxes highlight intervertebral disc regions (L3/4 to L5/S1) from which surgical tissue samples were obtained for downstream single-cell RNA sequencing (scRNA-seq).Enlarged views beneath each scan highlight structural changes in the discs associated with degeneration.

(B) Clinical profiles of the corresponding patients, including sex, age, diagnosis, surgical procedure, tissue collection site, and Pfirrmann grade based on MRI analysis. The Pfirrmann grading system classifies the degree of intervertebral disc degeneration from grade I (healthy) to grade V (severely degenerated).

Supplementary Fig. 2.Genotyping of transgenic mice and establishment of tail-loop intervertebral disc degeneration model.

A

Primer Name	Sequence(5'- 3')	PCR size	Primer illustration
olMR8052	GCGAAGAGTTTGTCCTCAACC	Mutant = 300bp<br>Wild type = 603bp	DTR
olMR8545	AAAGTCGCTCTGAGTTGTTAT		
olMR8546	GGAGCGGGAGAAATGGATATG		
Lyz2-iCre-tF1	AGTGCTGAAGTCCATAGATCGG	KI：543bp	Lyz2-iCre
Lyz2-iCre-tR1	CTGATTCTCCTCATCACCAGG		
Lyz2-iCre-tF1	AGTGCTGAAGTCCATAGATCGG	KI：1413bp<br>Wt：357bp	
Lyz2-iCre-tR2	GTCACTCACTGCTCCCCTGT		

B

Primer Name	Sequence(5'- 3')	PCR size	Primer illustration
H11-tF2	ATGCCCACCAAAGTCATCAGTGTAG	KI:1465bp	mG/mT
H11-CAG-5tR2	AGGCGGGCCATTTACCGTAAGTTA		
H11-tF3	GGGCAGTCTGGTACTTCCAAGCT	WT:285bp	
H11-			

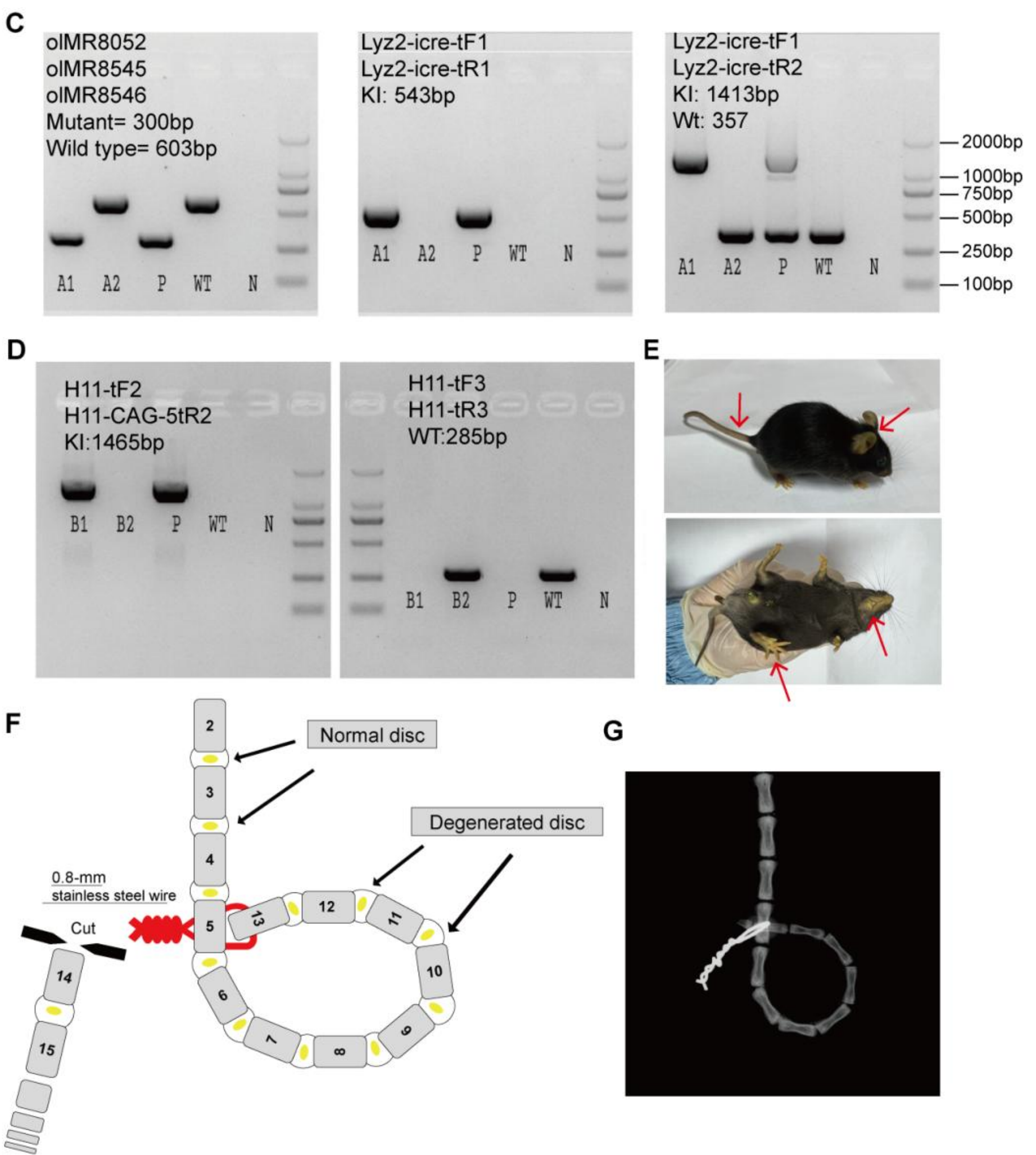

(A-B) Primer sequences used for genotyping DTR (A) and mG/mT (B) mouse lines crossed with Lyz2-iCre. PCR product sizes and genotyping strategy are indicated.
(C) PCR results for Lyz2-iCre and DTR transgenic alleles. Lane A1: Lyz2-iCre;DTR double transgenic mouse (KI/mutant),Lane A2: wild-type (WT) control,Lane P: positive control,Lane WT: WT DNA control,Lane N:No-Template Control.Expected bands: 300 bp for mutant DTR, 603 bp for WT; 543 bp and 1413 bp for KI Lyz2-iCre, 357 bp for WT.
(D) PCR genotyping of Lyz2-iCre crossed with mG/mT mice.Lane B1: Lyz2-iCre;mG/mT double transgenic mouse (KI),Lane B2: wild-type (WT) control,Lane P: positive control,Lane WT: WT DNA control,Lane N: No-Template Control.Expected bands: 1465 bp for mG/mT KI, 285 bp for WT.
(E) Gross appearance of a Lyz2-Cre$^{mG/mT}$ double transgenic mouse,as indicated by the red arrows. Strong GFP fluorescence is visible in the ears, paws, tail, and skin, consistent with Lyz2 promoter-driven Cre activity and membrane-localized GFP reporter expression.
(F) Schematic illustration of the tail-loop disc degeneration model. A stainless steel wire (0.8 mm diameter) is used to ligate vertebrae 5–13, creating mechanical stress on enclosed intervertebral discs (segments 6–12), which develop degeneration over time. Discs outside the loop serve as non-degenerate controls.
(G) X-ray radiograph confirming the closed-loop configuration of the tail vertebrae following wire ligation.

Supplementary Fig. 3.Quantitative assessment of histological degeneration, matrix remodeling, and macrophage involvement in Figure 1.

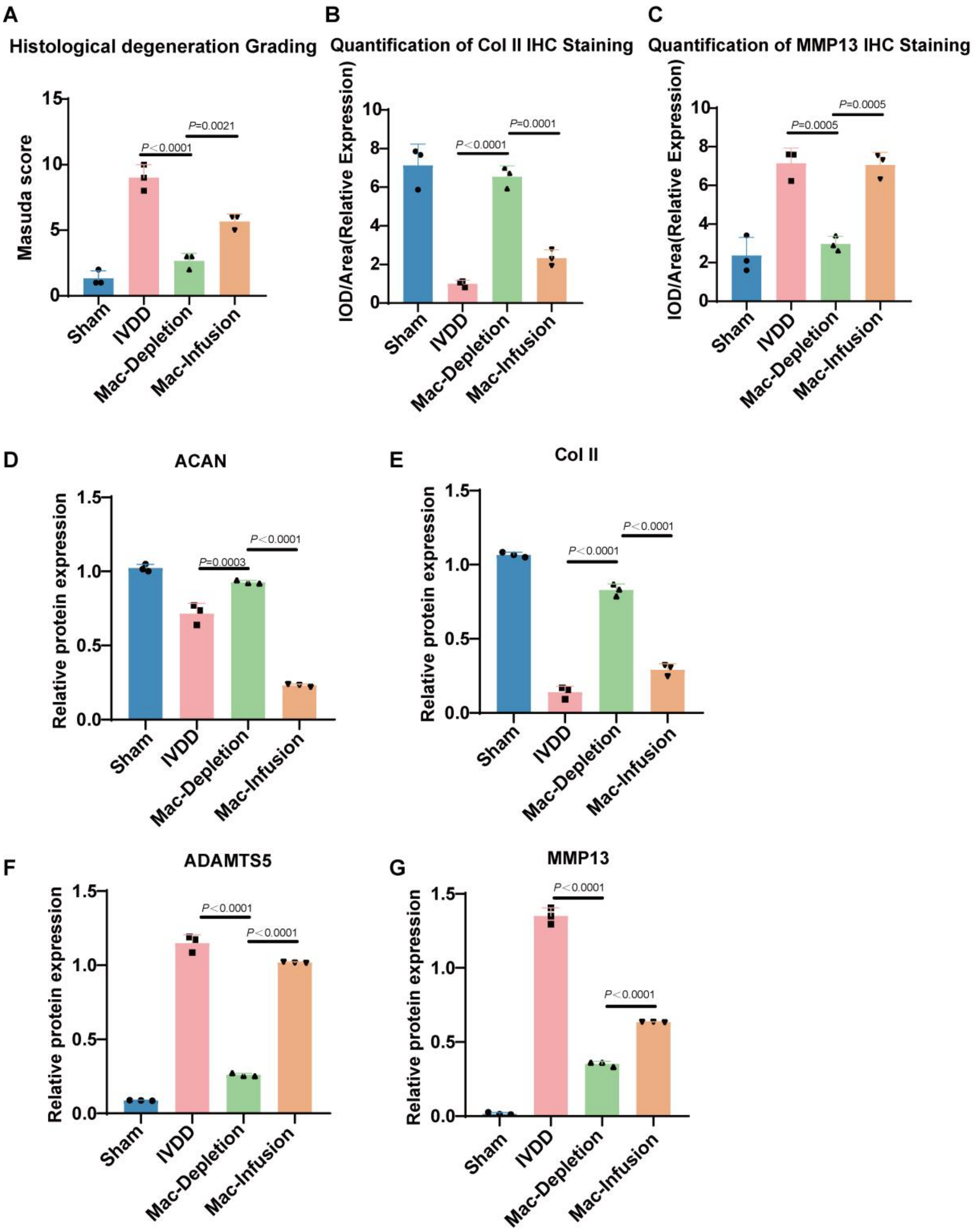

(A) Histological grading of intervertebral disc degeneration using the Masuda scoring system in Figure 1 H. Macrophage depletion significantly reduced degeneration scores compared to the IVDD group, while macrophage re-infusion partially increased degeneration levels.

(B-C) Immunohistochemical quantification of Figure 1 I: (B) Type II Collagen (Col II) and (C) MMP13 and Col II expression decreased in the IVDD group and was rescued by macrophage depletion. Conversely, MMP13 expression increased with degeneration and was partially normalized after macrophage depletion.

(D-G) Protein (Western Blot) quantification of disc matrix-related gene expression in

Figure 1 J, showing relative levels of (D) Aggrecan (ACAN), (E) Col II, (F) ADAMTS5, and (G) MMP13. Macrophage depletion restored anabolic gene expression (ACAN, Col II) and suppressed catabolic enzymes (ADAMTS5, MMP13), while macrophage infusion reversed these effects.
Each dot represents an individual biological replicate (n = 3 per group). Data are presented as mean ± SD.*P* value was determined by one-way ANOVA followed by Tukey's post-hoc test.

Supplementary Fig. 4.Analysis of osteoclast activity and vertebral bone integrity in a mouse model of intervertebral disc degeneration (IVDD) with macrophage depletion(lyz2-Cre iDTR) and infusion.

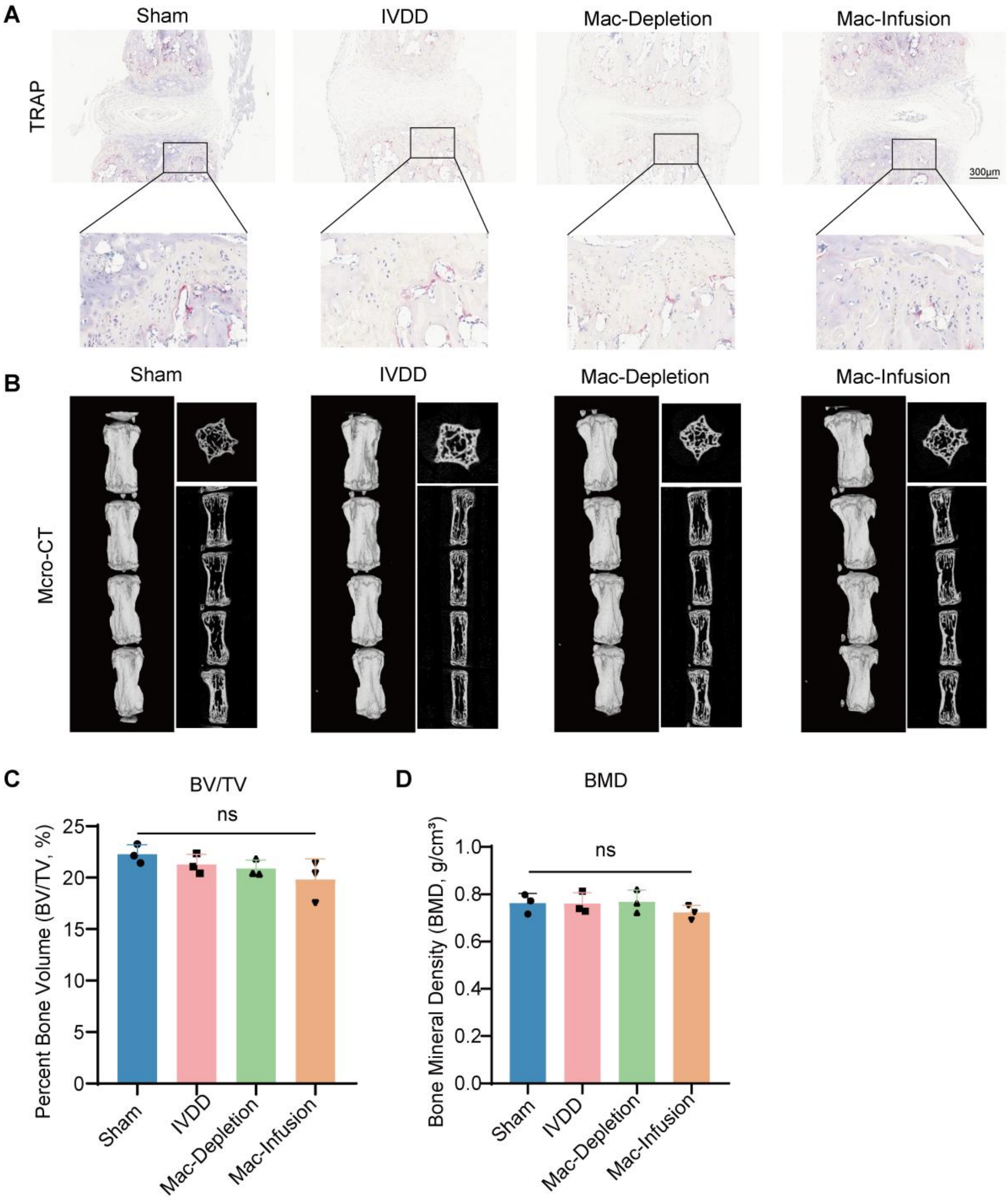

(A) Representative TRAP staining images of vertebrae adjacent to the degenerated intervertebral discs in Sham(WT mice without tail-loop surgery), IVDD(WT mice with tail-loop surgery), Mac-depletion(lyz2-Cre;iDTR mice with tail-loop surgery), and Mac-infusion groups(Lyz2-Cre;iDTR mice receiving macrophage infusion after tail-loop surgery.). TRAP-positive multinucleated osteoclasts (indicated in red) are scarce across all groups, suggesting minimal osteoclast activation. Scale bar=300 μm.

(B) Representative micro-computed tomography (micro-CT) images of lumbar vertebrae adjacent to the degenerated discs. Both transverse and sagittal views are shown. No gross structural defects or trabecular rarefaction were observed among

groups, indicating preserved vertebral bone architecture.

(C) Percent bone volume (BV/TV, %) of vertebral trabecular bone determined by micro-CT analysis. Vertebral bone mass was maintained in all groups with no significant difference ($n = 3$ per group; mean ± SEM; one-way ANOVA).

(D) Bone mineral density (BMD) measurements from micro-CT scans. No significant differences in BMD were observed across the groups ($n = 3$ per group; mean ± SD; one-way ANOVA)."ns" indicates no statistically significant difference.

Supplementary Fig. 5.In vivo experiments show that conventional liposomes cannot have a better therapeutic effect on intervertebral discs.

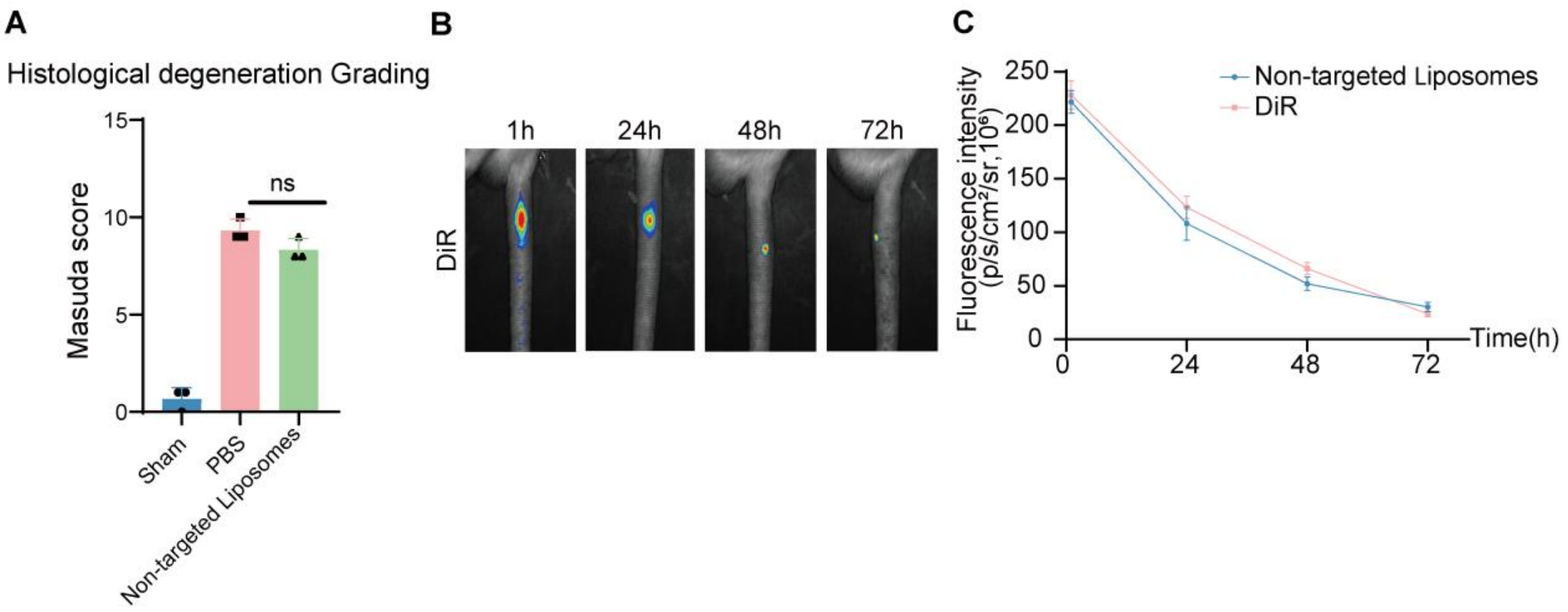

(A) Histological grading of intervertebral disc degeneration using the Masuda scoring system in Figure 3 B.

(B) Time-course in vivo imaging of DiR in rat intervertebral discs over 72h.

(C) Time-dependent decay of fluorescence signal from in vivo imaging of Non-targeted Liposomes(Figure 3C) and DiR.Each dot represents an individual biological replicate (n = 3 per group;one-way ANOVA). Data are presented as mean ± SD."ns" indicates no statistically significant difference.

Supplementary Fig. 6.In vitro and in vivo evaluation of the binding affinity and specificity of P1 and scrambled P1.

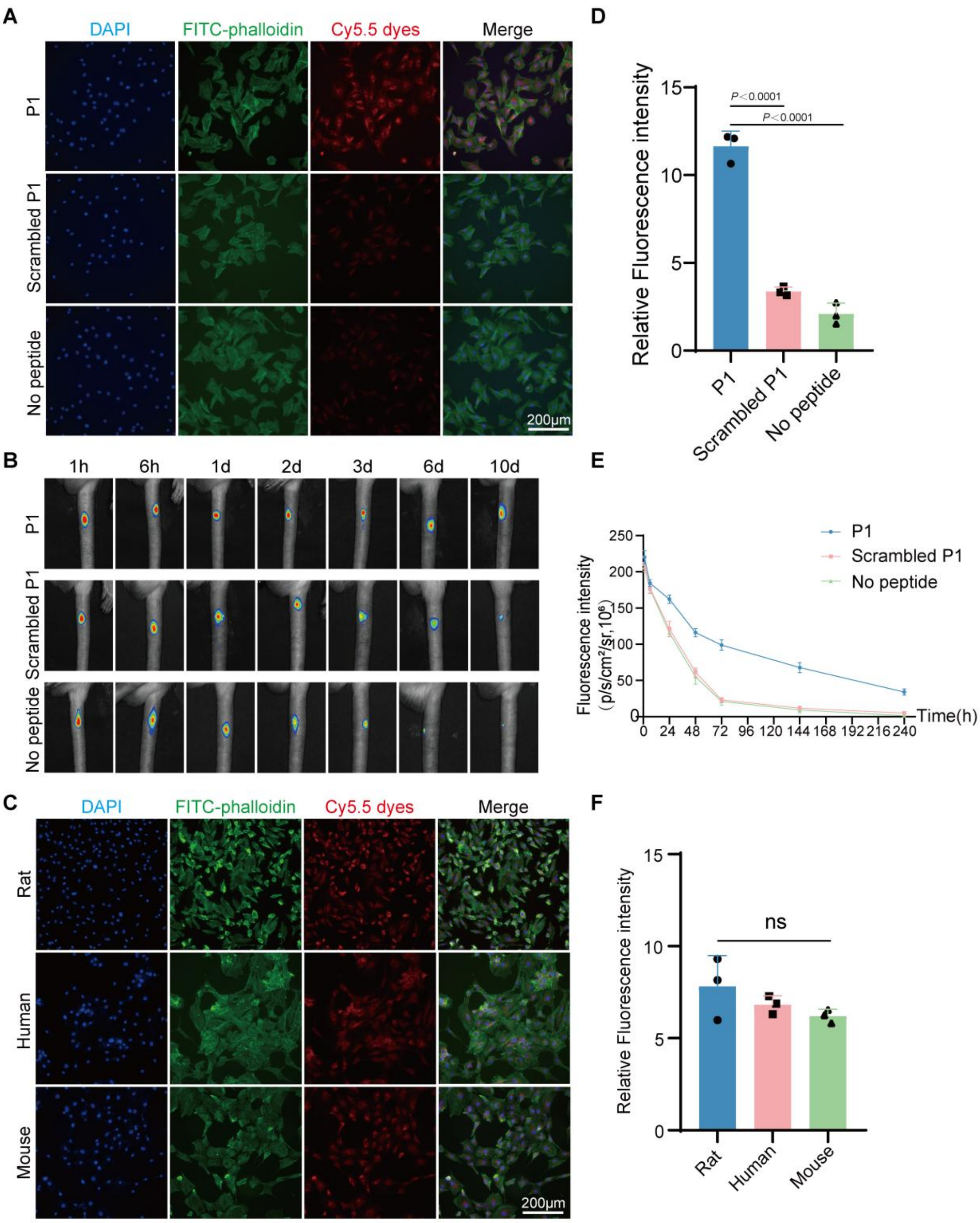

(A) Confocal microscopy images showing the binding of P1， scrambled P1 and No peptide (Cy5.5-labeled) with FITC-labeled AF cells after incubation at 10 μM for 30 minutes. DAPI was used for nuclear staining.

(B) In vivo fluorescence imaging of the accumulation of P1， scrambled P1 and No peptide (Cy5.5-labeled) in the rat intervertebral discs at different time points (1h, 6h, 1d, 2d, 3d, 6d, 10d) post-injection.

(C) Confocal microscopy demonstrating the specificity of P1 (Cy5.5-labeled) with different cell types, including Rat AF cells, Human AF cells, and Mouse AF cells after

incubation at 10 μM for 30 minutes.
(D) Quantification of relative fluorescence intensity in AF cells treated with P1, scrambled P1, or no peptide.
(E) Fluorescence intensity of P1， scrambled P1 and no peptide in rat intervertebral discs over time (1h to 240h) post-injection.
(F) Quantification of relative fluorescence intensity of P1 in Rat, Human, and Mouse AF cells. Each dot represents an individual biological replicate (n = 3 per group). Data are presented as mean ± SD.*P* value was determined by one-way ANOVA followed by Tukey’s post-hoc test."ns" indicates no statistically significant difference.

Supplementary Fig. 7.Physicochemical characterization and cellular uptake of AF-PTK@LEV@SPP1.

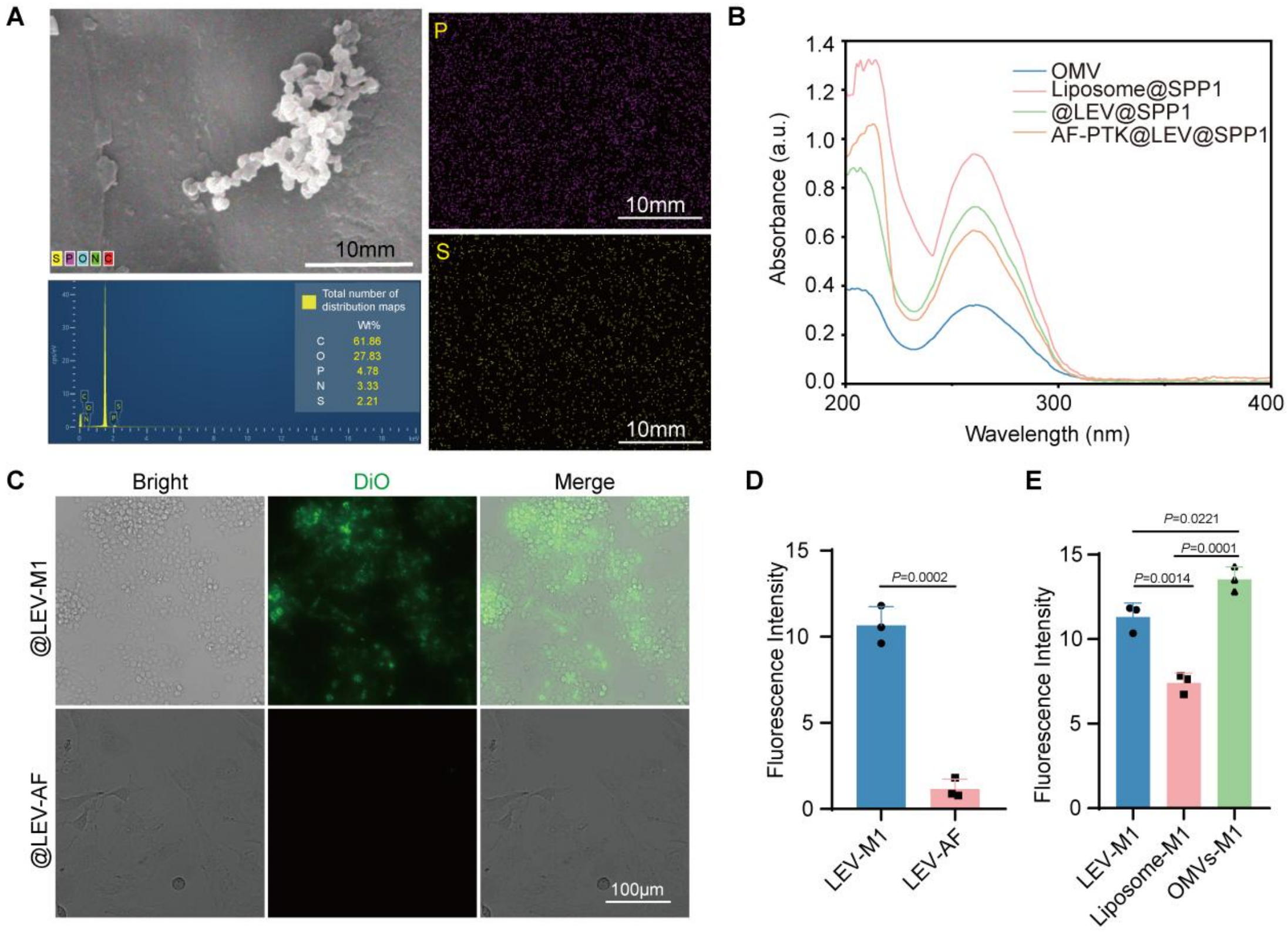

(A) SEM image, energy-dispersive X-ray spectroscopy (EDS) spectrum, and elemental distribution maps showing the composition of AF-PTK@LEV@SPP1, with elemental mapping for phosphorus (P) and sulfur (S). Scale bar for elemental maps, 10 mm.

(B) UV–visible absorption spectra of OMVs, Liposome@SPP1, @LEV@SPP1, and AF-PTK@LEV@SPP1.

(C) Fluorescence microscopy images of M1 macrophages and AF cells incubated with DiO-labeled LEV at 10 μM for 30 minutes. Scale bar = 100 μm.

(D) Quantification of relative fluorescence intensity in M1 macrophages and AF cells following nanoparticle incubation.

(E) Quantification of DiO fluorescence intensity in M1 macrophages after incubation with DiO-labeled @LEV, liposome, or OMVs formulations in Figure 4(G).

Each dot represents an individual biological replicate (n = 3 per group). Data are presented as mean ± SD.*P* value was determined by one-way ANOVA followed by Unpaired t test(D) and Tukey's post-hoc test(E).

Supplementary Fig. 8.NF-κB signaling and paracrine crosstalk mediate the AF-PTK@LEV@SPP1-induced restoration of annulus fibrosus cell homeostasis.

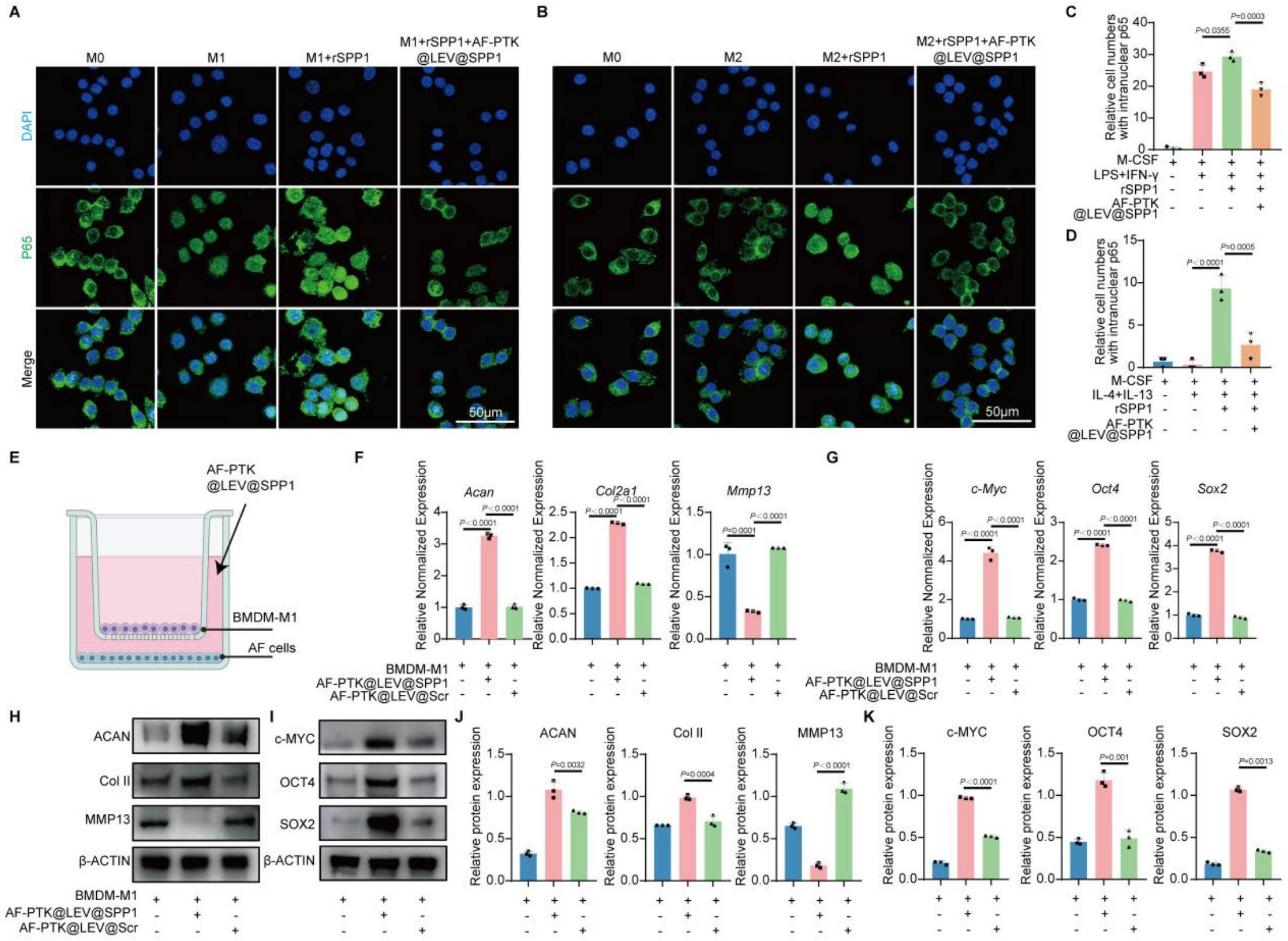

(A, B) Immunofluorescence images showing NF-κB p65 nuclear translocation in each treatment group.

(C, D) The number of cells with intranuclear p65.The results were normalized and analyzed using Student's t-test (n = 3,mean±SD).

(E) Schematic diagram of the Transwell co-culture system with BMDM-M1 and AF cells exposed to different treatments.

(F, G) Quantitative real-time PCR analysis of AF cell gene expression, including aggrecan (*Acan*), collagen type II (*Col2a1*), matrix metalloproteinase 13 (*Mmp13*), and stemness-associated genes (*Oct4*, *Sox2*, *c-Myc*), following indirect exposure to treated BMDMs.(n = 3,mean±SD)

(H, I) Western blot analysis of the indicated proteins in AF cells co-cultured with BMDMs pretreated as shown.

(J, K) Protein (Western Blot) quantification of in Supplementary Fig. 8. H-I.(n = 3,mean±SD).

Representative images are shown. Data are presented as mean ± SD from at least three independent experiments. *P* value was determined from one-way ANOVA followed by Tukey post-hoc test. Source data are provided as a Source Data file.

Supplementary Fig. 9. AF-PTK@LEV@SPP1 alleviates intervertebral disc degeneration by suppressing inflammatory signaling and reprogramming macrophage polarization.

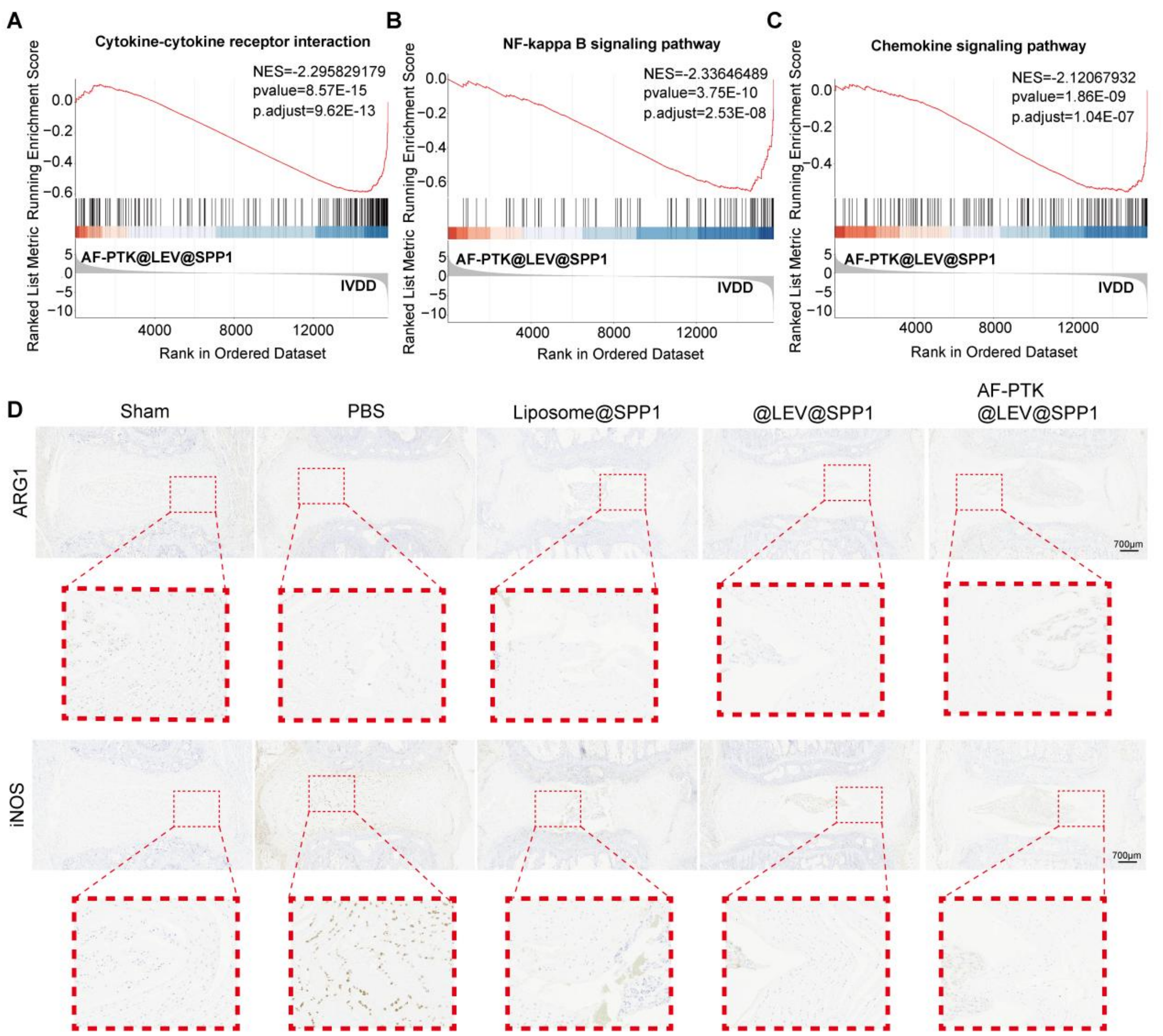

(A-C) GSEA plots showing negative enrichment (NES < 0) of Cytokine–cytokine receptor, NF-κB, and Chemokine signaling pathways in AF-PTK@LEV@SPP1-treated discs, consistent with global suppression of inflammatory transcriptional programs.

(D) Immunohistochemical staining of ARG1 (M2 macrophage marker) and iNOS (M1 marker) in intervertebral discs from different treatment groups (Sham, PBS, Liposome@SPP1, @LEV@SPP1, AF-PTK@LEV@SPP1). Enhanced ARG1 and reduced iNOS expression in AF-PTK@LEV@SPP1-treated tissues confirm macrophage polarization toward an anti-inflammatory M2 phenotype(n=6).

Supplementary Fig. 10. Biosafety evaluation of AF-PTK@LEV@SPP1 and P1 peptide in vivo and in vitro.

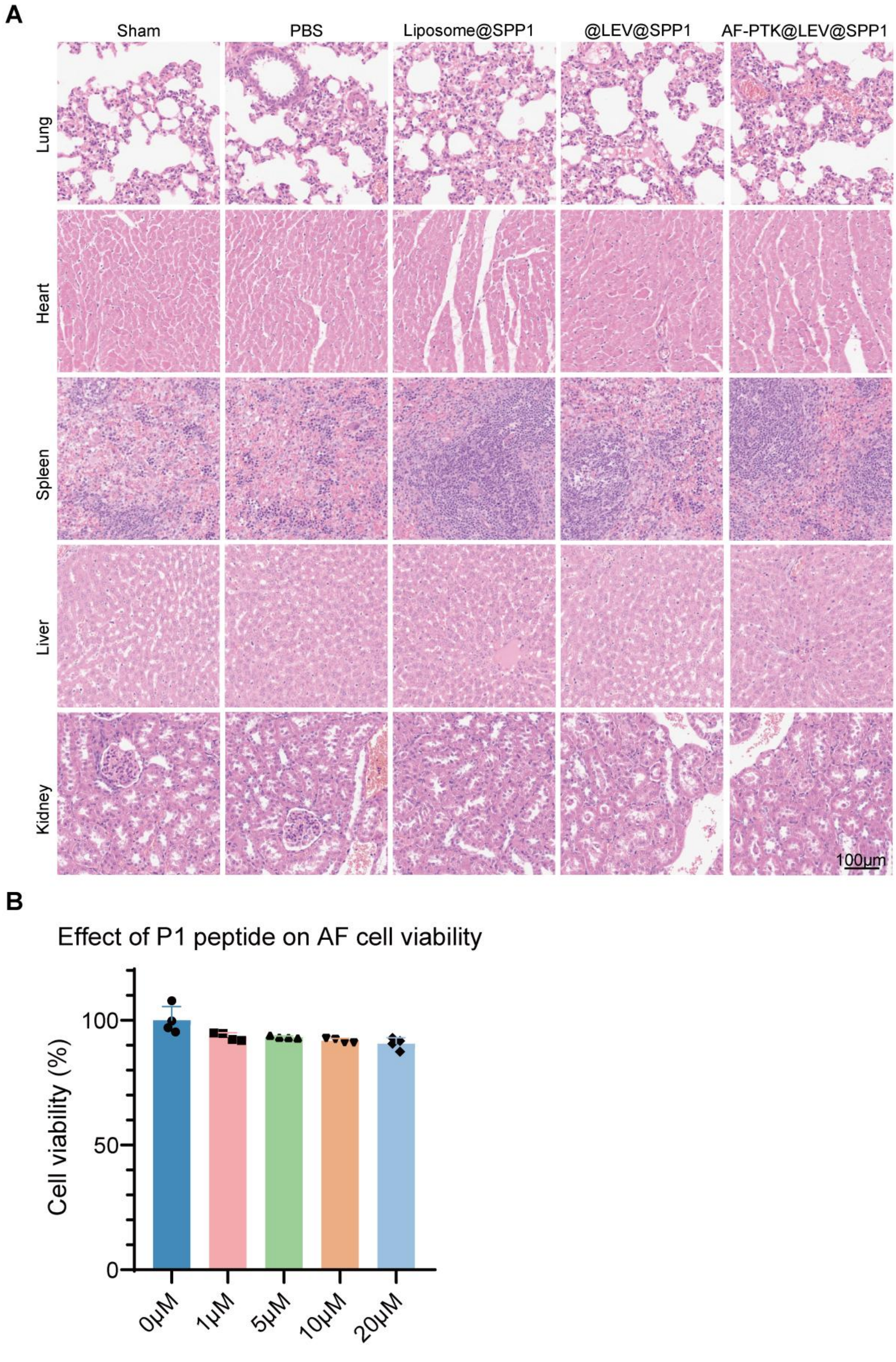

(A) Representative hematoxylin and eosin (H&E) staining of major organs (lung, heart, spleen, liver, and kidney) collected from rats 7 days after intravenous

administration of Sham,PBS, Liposome@SPP1, @LEV@SPP1, or AF-PTK@LEV@SPP1. No evident histopathological abnormalities were observed in any group, indicating good systemic biocompatibility. Scale bar: 100 μm.

(B) Cell viability of annulus fibrosus (AF) cells after incubation with increasing concentrations (0–20 μM) of P1 peptide for 24 hours, assessed by CCK-8 assay. Data are presented as mean ± SD (n = 4).

Supplementary Fig. 11.Representative gating strategy for flow cytometry experiments.

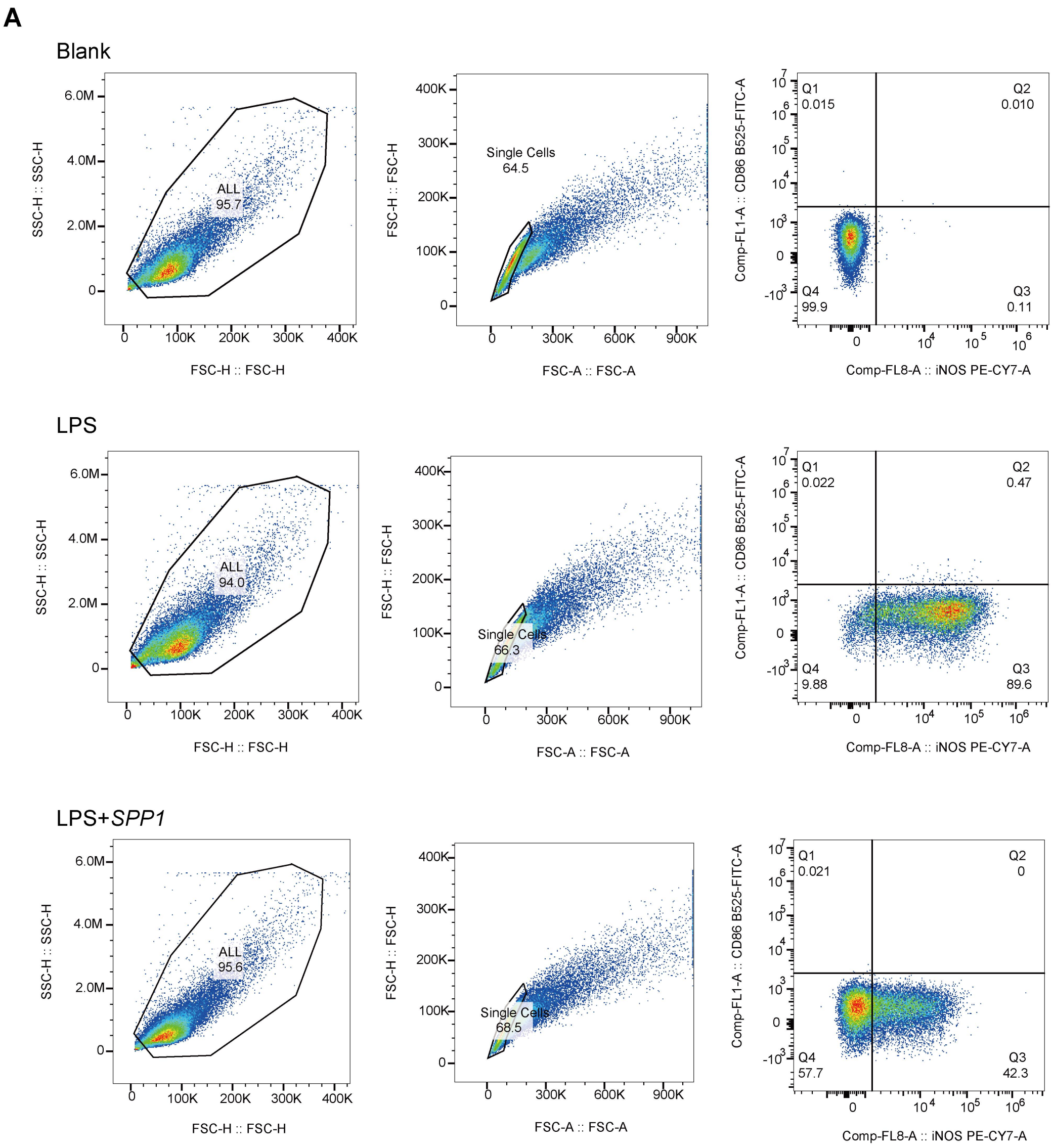

(A)Blank control, LPS,LPS+*SPP1* and gating strategy for flow cytometry.The same gating strategy was applied to all samples in Figures 2H and 6C.

Supplementary Fig. 12.Agarose gel electrophoresis assessing siRNA stability in different formulations over time under enzymatic degradation conditions with molecular weight markers.

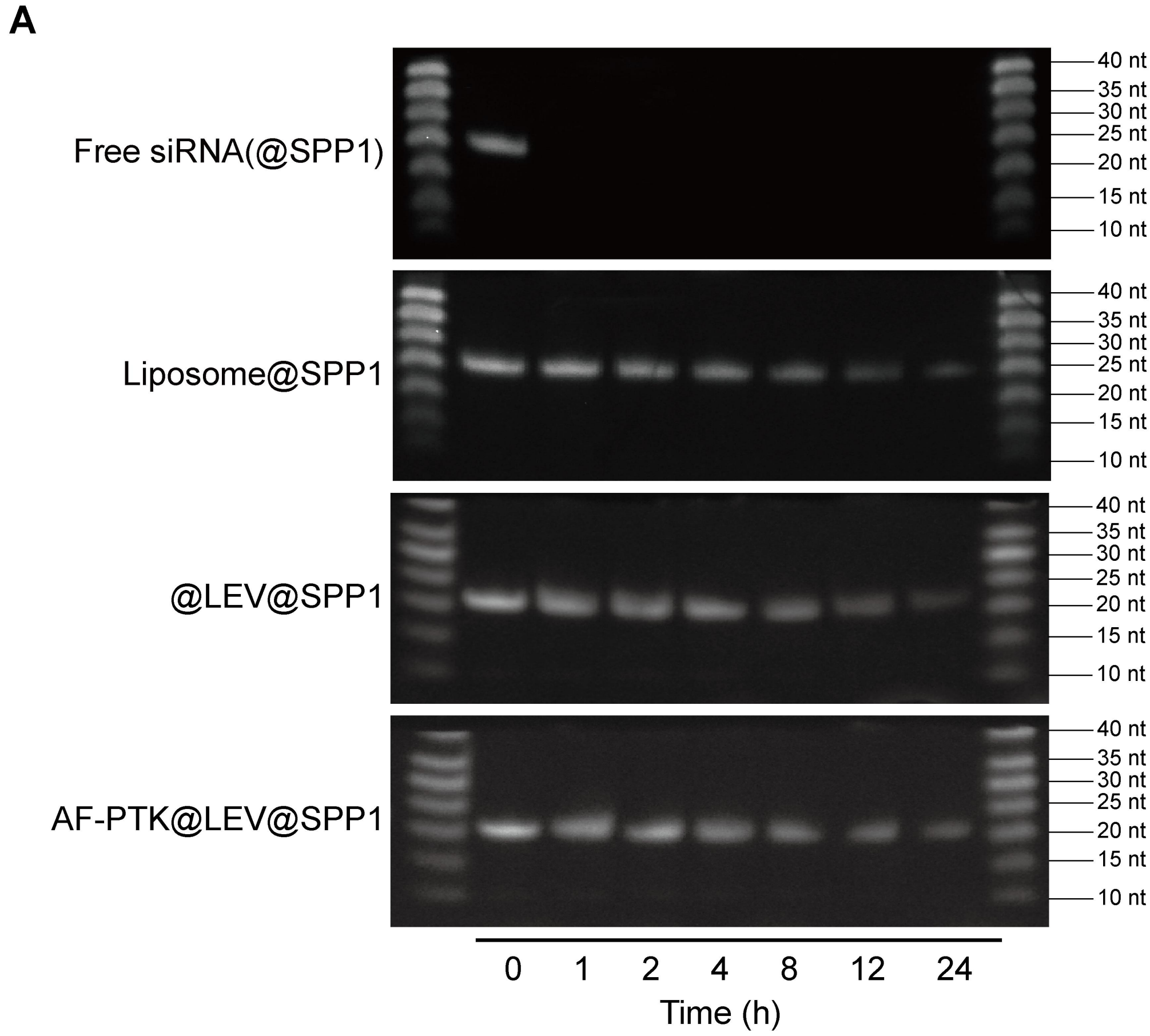

(A)Agarose gel electrophoresis assessing siRNA stability in different formulations over time under enzymatic degradation conditions with molecular weight markers. Four groups were tested: free siRNA, Liposome@SPP1, @LEV@SPP1, and AF-PTK@LEV@SPP1.The expected RNA duplex region is 19 nt, with an overall oligonucleotide length of approximately 21 nt including the 3′ dTdT overhangs.

Supplementary Table1. The used primer sequences.

Gene	Strand	Primer Sequences
Col2a1(Col II)	forward	5’-GAGCAAGAGCAAGGACAAGGA-3’
	reverse	5’-GGTGGACAGTAGATGAAGCGG-3’
MMP13	forward	5’-TGGAGTCTGGAACTCATGGC-3’
	reverse	5’-GCCATCCAGTTCAGGGAACA-3’
Acan	forward	5’-AGACGCTGCAGGACATCAAC-3’
	reverse	5’-GGTGGTGTCATCGTCATAGC-3’
Adamts5	forward	5’-GCCATCAACGACATCAAGGA-3’
	reverse	5’-GGTCCAGCTCGATGATGTCC-3’
Spp1	forward	5’-CACAAGCAGACACTTTCACTCCA-3’
	reverse	5’-CCATAAGCCAAGCTATCACCTCG-3’
c-Myc	forward	5’-CTGCATGAGGAGACACCGCC-3’
	reverse	5’-AGCCCGACTCCGACCTCTTG-3’
Oct4	forward	5’-GAGGGAACCTCCTCTGAGCC-3’
	reverse	5’-GCAGGGCTTTCATGTCCTGG -3’
Sox2	forward	5’-ATGAACGGCTCGCCCACCTA-3’
	reverse	5’-CTGGAGCTGGCCTCGGACTT-3’
