# Supplementary material for: An annulus fibrosus anchored nanovector reprograms osteopontin driven macrophages in preclinical disc degeneration models
Source: Nat Commun. 2026 Jul 21;17:8887. doi: 10.1038/s41467-026-75720-3 (PMC13500848; doi:10.1038/s41467-026-75720-3)
Supplement: Supplementary file 2 [file 41467_2026_75720_MOESM2_ESM.pdf]

nature portfolio

Peer Review File

Hierarchical targeting reprograms osteopontin driven macrophages to alleviate disc degeneration

Corresponding Author: Professor Ning Zhang

This file contains all reviewer reports in order by version, followed by all author rebuttals in order by version.

Attachments originally included by the reviewers as part of their assessment can be found at the end of this file.

Version 0:

Reviewer comments:

Reviewer #1

(Remarks to the Author)
This manuscript describes the use of an annulus fibrosis-binding peptide attached to a osteopontin siRNA delivering system based on bacterial EVs with the aim to target macrophages as players in IVDD.

-Referencing is done randomly:
None of references 1-5 support the statement of the associated sentence on the burden of IVDD and chronic low back pain. Two of them are even preclinical research papers on different delivery systems. Similar for reference 6-10 supposed to support the statements in the previous 2 sentences. References 11-16 after ..”advances in single-cell sequencing have unveiled the critical role of immune cells, particularly macrophages, in the inflammatory microenvironment of degenerating intervertebral discs” do not match either. E.g. 11 is a study protocol for a clinical trial in radiculopathy patients, 12 is one on paraspinal muscle degeneration and regeneration. Especially when the authors refer to their study evaluating macrophage infiltration using “macrophage-specific fluorescent reporter mice (Lyz2-Cre; mG/mT)” which is the basis of the current study, none of the references 23-25 refer to such a study, in fact these all refer to in vitro studies.
This makes it difficult to verify whether the statements leading to the research question are valid.

- Human IVD single cell sequencing. How many IVD specimens were used, how were the patients clinically treated, how was degeneration determined (Pfirrmann or Thompson) and what were the scores, was the entire IVD digested or was it separated in AF and NP tissue?

-It is not clear whether the mouse models with macrophage-specific florescence and the one with macrophage-specific knock outs have been validated, so it is hard to understand whether the data obtained here with these animals are valid.

-The fact that macrophages were depleted does not mean they were directly involved in IVD degeneration. I assume Lyz2 is also expressed in osteoclasts and hence the adjacent bone may have changed, known to be able to cause degeneration of adjacent cartilaginous structures. Replenishing them exogenously to the IVD could have shown a direct effect.

-Why would MMP-13 expression by macrophages only affect the AF and not the NP tissue?

Figure 1J;
- The change in mRNA expression between degenerated and degenerated-macrophage depleted for ACAN, COLII and MMP13 is way less than the 2-fold usually taken as meaningful in expression analysis.

- Protein (Western Blot/immunohistochemistry) quantification is missing.

- Grading and subsequent quantitative analysis of degeneration is lacking, also further on in the MS

- Figure 3 It is not clear how the liposomal formulations were administered. As systemic administration of nanoparticles is

known to generally only reach tissues with fenestrated capillaries such as the liver, but not structures like the IVD, I assume these were injected intradiscally. However, as this should be a relatively contained space, I wonder why without targeting, fast loss of signal was observed as they are unlikely to have diffused out? Moreover, there was no direct comparison with targeted liposomes, so could the injected quantity have been different between the 2 experiments?

-"We..co-cultured peptide 1-Cy5.5 with AF cells, cartilage endplate cells, and chondrocytes." Why not with NP cells? Endplate cells áre chondrocytes, and hence it is not surprising the response is the same.

- The control peptide seems not to be a scrambled version of peptide 1. This is a serious omission.

- Figure 3K shows retention, not accumulation

- Authors should show by microscopical analysis of cryosections of the injected discs that actually the peptide was retained in the AF rather than the NP

- Specificity of the binding of the AF-peptide liposomes should be confirmed again in AF, NP and CEP cell culture, as conformation changes may have occurred losing specificity. Comparisons should be made between binding peptide, scrambled (and therefore nonbinding) peptide and no peptide.

-Figure 5B. How do authors distinguish fibroblasts from macrophages in the 2D culture and thereby the transfer of the particles from one cell type to another after ROS activation?

- For the data in Figure 5C, it seems no scrambled siRNA was used. This control is essential.

- In the legend for 5H, it says "Representative immunohistochemical staining for AGG, MMP13, SOX2, OCT4, and c-MYC in intervertebral discs of macrophage-specific knockout mice after different treatments" However, these figures show WB results and there are only 2 conditions tested.

-By the way, in the manuscript, the authors refer to fig 6F-I, but these show fluorescence intensity after binding of the peptide-conjugated versus unconjugated liposomes in vitro and in vivo

- Stemness as demonstrated by higher OCT4, SOX2 and c-myc seems to be opposite to increase in aggrecan expression

- several images in Fig 6G seem vertically stretched (liposomes without peptide; 1h, 1d, 2d)

- I am unfamiliar with Pfirrmann grading for IVDD in the mouse. Please refer to the study validating this classification for use in mouse models

-also here the control lacks the scrambled version of the AF binding peptide

-In general data on viability of peptide and nanoparticles seem to be missing

Reviewer #2

(Remarks to the Author)
In the manuscript by Li et al. they address the need to block the generation of M1 macrophages in the annulus fibrosus, since this macrophage population is the major cause of tissue damage in their IVDD mouse model. Their studies showed that the glycoprotein SPP1 (osteopontin) is a key regulator of macrophage activation and M1 polarization within the disc. To accomplish this goal of limiting the generation of M1 macrophages and to do so using an approach that can be used clinically, they developed a novel extracellular vesicle (EV). The EV consists of a liposome containing an siRNA to SPP1 fused to an E. coli OMV. They predicated this EV would better target macrophages compared to a liposome. To keep the EV localized to the annulus fibrosus (AF), they screened and identified a peptide that binds to AF cells and coupled this peptide to the OMV-liposome EV (LEV@SPP1) to generate the AF-PTK@LEV@SPP1. The peptide linkage was labile and would be cleaved from the EV upon exposure to oxidative stress (e.g. exposure to ROS). In general, I thought the experiments supported the authors claims. My comments are specific to the generation and testing of their EVs.

Additional characterization of the AF-PTK@LEV@SPP1 is needed.

Co-localization of DiR Fluorescent dye labeled OMV and liposomes is not sufficient to show membrane fusion as exchange of membrane but not fusion of membranes is possible. This is especially a concern since both the OMV and liposomes are ~150nm but the fusion produce is also ~150nm. That is, there is no increase in size from the vesicle fusion, perhaps because they are selecting vesicles of <200nm in their filtration steps. Looking for specific markers of OMV and liposome on the fused EVs should be performed to confirm that they have generated fused vesicles. It is also important that they show the reproducibility of the fused EVs. That is, how much variation do they see in the composition of the AF-PTK@LEV@SPP1 with different preps.

They describe their purification process as follow:

“After sonication, the suspension was centrifuged at 10,000 g for 10 min to remove aggregates. The supernatant containing fusogenic liposomes was collected and further purified by ultracentrifugation at 100,000 g for 1 h at 4°C. The final pellet was resuspended in sterile PBS and stored at 4°C for subsequent characterization.”

“EVs were labeled with DiR fluorescent dye (red) by mixing the EV solution with DiR and incubating at 37 °C for 15 minutes. The solution was then purified by centrifugation (12,000 rpm, 10 minutes) to obtain DiR-labeled EVs. Similarly,siRNA-loaded liposomes were labeled with DiO fluorescent dye (green) by incubating the liposome solution with DiO at 37 °C for 15 minutes, followed by centrifugation (12,000 rpm, 10 minutes) to purify the DiO-labeled liposomes.The DiR-labeled EVs and DiO-labeled liposomes were mixed and repeatedly extruded through 400 nm and 200 nm polycarbonate membranes using a liposome extruder. After extrusion, the fusion of EVs and liposomes was verified using confocal microscopy.”

From these descriptions it appears they used different processes to purify the fused OMVs and liposomes (AF-PTK@LEV@SPP1). For the EM and fluorescent microscopy studies (Figure 4) they used filtration while for the in vivo experiments they used centrifugation at 100,000 xg. Therefore, it is unclear that any characterization was done on the vesicles that were purified by centrifugation.

Other comments:
What is the uptake of OMVs without fusion with liposomes?

Figure 4E. The colocalization of @LEV with macrophages was difficult to see in this figure. Higher magnification and quantification would be useful to show.

Figure 6C-I they are missing the important control of LPS + Liposomes@SSP1 to see if they are equally efficient at knocking down LPS driven macrophage activation (M1 phenotype) as the AF-PTK@LEV@SSP1. The prediction would be that they are not as efficient but this needs to be tested experimentally.

Minor comment:
Exosomes is used to specifically designate EVs released upon fusion of MVBs with plasma membrane and release of the intraluminal vesicles. This is not what EVs were used in this study. The generic term EVs should be used.

Reviewer #3

(Remarks to the Author)
Li et al. reported the development of a compound targeting both macrophages and annulus fibrosus (AF) for the treatment of disc degeneration. Using single-cell RNA sequencing, they demonstrated that proinflammatory macrophages infiltrate human degenerated discs and identified SPP1 as a key regulator of macrophage activation in disc degeneration. To further investigate the role of SPP1 in macrophage activity and disc regeneration, they developed an AF-PTK@LEV@SPP1 targeted delivery system. This system incorporates an AF-binding peptide, liposomes, bacterial exosomes, and a thioketal linker responsive to reactive oxygen species (ROS) for controlled release.
The researchers evaluated this system in both in vitro and in vivo models to assess binding specificity, release profile, and retention. Furthermore, they tested the therapeutic potential of SPP1 modulation in a rat disc degeneration model. This study offers valuable insights into the potential of macrophage-targeted therapies for treating disc degeneration. However, it is difficult to evaluate without detailed information in the methods and results.

Some specific comments:

Introduction:

The introduction lacks clarity in distinguishing between previous research and the current study's objectives.
It should be reorganized to provide a clearer background and rationale.
Reference errors: References 23–25 do not match the cited content. Additionally, references 36–40 may be incorrectly placed.

Materials & Methods:

More experimental details are needed, including animal age, gender, and specific experimental conditions.
Clarify the AF cell selection process for the peptide targeting AF cells.
Provide details on compound administration, including dosage, method, and timing of injection into intervertebral discs.
The discrepancy between the described disc degeneration model (L3/L4, L4/L5, or L5/L6 lumbar discs) and the figures suggesting caudal disc degeneration needs to be addressed.
Clarify whether the AF-binding peptide also binds to mouse or human AF cells.
Specific details of degenerated discs of humans.

Figures: Please improve figure legends and labels for better clarity. For example,
Figure 1F: The distinction between normal vs. IVD is unclear.
Figure 1I: The "normal" disc for MMP13 staining appears degenerated discs
Figures 7F & 8: the labels of these figures are difficult to read.

Typos: Acupuncture?

Version 1:

Reviewer comments:

Reviewer #1

(Remarks to the Author)
The authors have performed an amazing amount of additional analyses and even in vivo experiments and answered all of my questions.

Reviewer #2

(Remarks to the Author)
The authors have addressed most of my concerns through additional data and revisions to the text. I have one remaining concern/comment. The SDS page study was to show that the OMV and liposome fusion occurred. However, it was difficult to see any significant differences between OMVs and OMVs + liposomes in the stained gel. Further mixing liposomes + OMVs without fusion will show the same banding pattern as all the components would be present in the mixed sample. I would suggest either removing this data or analyzing for specific lipid/protein components by confocal immunofluorescence to show co-labeling.

Reviewer #4

(Remarks to the Author)
Please see an attached report.

Reviewer #5

(Remarks to the Author)
I co-reviewed this manuscript with one of the reviewers who provided the listed reports. This is part of the Nature Communications initiative to facilitate training in peer review and to provide appropriate recognition for Early Career Researchers who co-review manuscripts.

Version 2:

Reviewer comments:

Reviewer #2

(Remarks to the Author)
No additional comments. This is a well-developed study.

Reviewer #4

(Remarks to the Author)
The authors have made substantial efforts to revise the manuscript and have addressed most of my previous concerns, including clarifying the macrophage polarization markers, refining the bulk RNA-seq results, and improving the figures, introduction, and methods. However, I still have major concerns regarding the reproducibility of these in vivo findings. The use of only three samples per group is insufficient to support the main conclusions of the study.

The authors state that “each biological replicate was an independent animal, and all experiments were repeated three times with consistent outcomes,” which indicates that the in vivo experiments were repeated. If this is the case, the corresponding datasets from these repeated experiments should be included in the Supplementary Information to substantiate the reproducibility of the findings. Although some publications use three animals per group, providing additional datasets would greatly strengthen the reliability of the results and prevent the study’s conclusions from relying on a single in vivo experiment.

If increasing the number of biological replicates is not feasible, the authors should clearly indicate whether the presented data are representative of one of the repeated experiments and provide the corresponding data as Supplementary Information. Alternatively, they should explicitly acknowledge this limitation, although doing so may affect the manuscript’s suitability for publication in the Nature Communications.

I also recommend that the authors specify the number of experimental repeats throughout the manuscript and clarify whether the figures reflect representative or combined data.

Reviewer #5

(Remarks to the Author)

I co-reviewed this manuscript with one of the reviewers who provided the listed reports. This is part of the Nature Communications initiative to facilitate training in peer review and to provide appropriate recognition for Early Career Researchers who co-review manuscripts.

Version 3:

Reviewer comments:

Reviewer #4

(Remarks to the Author)
Although the authors have adequately addressed many of the concerns, I believe that an increased number of biological replicates remains necessary. This study could be considered for acceptance if the authors provide additional data with an adequate sample size.

Version 4:

Reviewer comments:

Reviewer #4

(Remarks to the Author)
By increasing the number of experimental animals, the authors have demonstrated the robustness of their results. This contributes a valuable finding to the field; I have no further queries.

Open Access This Peer Review File is licensed under a Creative Commons Attribution 4.0 International License, which permits use, sharing, adaptation, distribution and reproduction in any medium or format, as long as you give appropriate credit to the original author(s) and the source, provide a link to the Creative Commons license, and indicate if changes were made.
In cases where reviewers are anonymous, credit should be given to 'Anonymous Referee' and the source.
The images or other third party material in this Peer Review File are included in the article's Creative Commons license, unless indicated otherwise in a credit line to the material. If material is not included in the article's Creative Commons license and your intended use is not permitted by statutory regulation or exceeds the permitted use, you will need to obtain permission directly from the copyright holder.
To view a copy of this license, visit https://creativecommons.org/licenses/by/4.0/

Responses to Reviewer #1 :

This manuscript describes the use of an annulus fibrosis-binding peptide attached to a osteopontin siRNA delivering system based on bacterial EVs with the aim to target macrophages as players in IVDD.

RE: We sincerely thank you for your thoughtful and constructive comments on our manuscript. We are particularly grateful for the concise and insightful summary that recognizes the core innovation of our study—the development of an annulus fibrosus-targeting peptide-based delivery system utilizing bacterial extracellular vesicles (EVs) for siRNA-mediated suppression of osteopontin (SPP1) in macrophages within the degenerative intervertebral disc microenvironment.

Your recognition of the rationale and strategy behind targeting macrophage-mediated inflammation through this biomimetic delivery approach is deeply appreciated. We believe your positive assessment reflects a clear understanding of the mechanistic depth and translational significance of our work.

In response to your valuable feedback and suggestions, we have made several careful and substantial revisions to the manuscript to further clarify our methodology, strengthen the experimental rationale, and enhance the overall presentation and flow of the text. Below, we provide a detailed summary of the key revisions that have been made accordingly.

Once again, we would like to express our sincere gratitude for your time, expertise, and support in improving the quality of our work. Your encouraging remarks have been highly motivating and instrumental in shaping this revised version of the manuscript.We really appreciate all your comments and suggestions. Below is a concise overview of the key revisions, meticulously aligned with your guidance.

-Referencing is done randomly:

None of references 1-5 support the statement of the associated sentence on the burden of IVDD and chronic low back pain. Two of them are even preclinical research papers on different delivery systems. Similar for reference 6-10 supposed to support the statements in the previous 2 sentences. References 11-16 after ..”advances in single-cell sequencing have unveiled the critical role of immune cells, particularly macrophages, in the inflammatory microenvironment of degenerating intervertebral discs” do not match either. E.g. 11 is a study protocol for a clinical trial in radiculopathy patients, 12 is one on paraspinal muscle degeneration and regeneration. Especially when the authors refer to their study evaluating macrophage infiltration using “macrophage-specific fluorescent reporter mice (Lyz2-Cre; mG/mT)” which is the basis of the current study, none of the references 23-25 refer to such a study, in fact these all refer to in vitro studies.

This makes it difficult to verify whether the statements leading to the research question are valid.

RE:We sincerely thank you for this critical and insightful observation.

Your comments have been invaluable in helping us improve the scientific rigor and clarity of our manuscript. We recognize that several of the references originally cited in the Introduction did not optimally align with the statements they were intended to support. While our goal was to provide comprehensive background context, we now realize that some of the citations may have led to ambiguity or difficulty in verifying the rationale leading to our research question.

To address this concern, we have now comprehensively revised the Introduction section of the manuscript. We restructured the background narrative to ensure a clear and logical progression of ideas, and we have replaced all previous references with carefully selected, thematically accurate, and up-to-date primary literature.

1. Burden of IVDD and Chronic Low Back Pain (Refs 1–5)

The first paragraph now cites major epidemiological studies and systematic reviews that robustly quantify the global burden, prevalence, and socioeconomic impact of chronic low back pain and IVDD. Specifically:GBD 2017 and GBD 2021 analyses in The Lancet (Refs 1, 2),Foundational epidemiological reviews by Andersson and by Hartvigsen et al. (Refs 3, 4),Global burden estimates from Hoy et al. (Ref 5).These citations directly support the opening statement about IVDD’s global impact and public health importance.

2. Limitations of Current Interventions and Promise of Biomaterials (Refs 6–10)

We replaced prior irrelevant preclinical delivery-system citations with authoritative reviews and translational studies from Nature Reviews Rheumatology, Advanced Drug Delivery Reviews, Bioactive Materials, etc. For example:Reviews on cell-based and progenitor-cell therapies (Refs 6, 7),Recent analyses of annulus fibrosus biomechanics and calcification barriers (Refs 8, 9),A new immunometabolic perspective on IVDD pathophysiology (Ref 10).These references directly discuss clinical and translational obstacles, setting up the rationale for novel delivery approaches.

3. Biological Structure and Barriers of the Disc (Refs 11–16)

For statements about anatomy, matrix barriers, nutrient diffusion limits, and poor intrinsic regenerative capacity, we now cite detailed anatomical/mechanistic studies and tissue engineering reviews:Disc mechanics and degeneration pathophysiology (Ref 11),Molecular basis of matrix degradation and inflammation (Refs 12, 13),Structural studies and regenerative scaffold approaches specific to annulus fibrosus (Refs 14–16).These

accurately support discussion of physical and biological barriers to therapy.

4. Immune Cell Role, Macrophage Heterogeneity, and Single-Cell Data (Refs 19–29)

For the section on macrophage infiltration, polarization states, and single-cell transcriptomics, we now include:Single-cell RNA-seq analyses specifically characterizing macrophage heterogeneity in IVDD (Ref 26),Reviews on macrophage plasticity and inflammatory arthritis mechanisms (Refs 27, 28),Experimental studies confirming M1/M2 polarization and secreted cytokine profiles in degenerating discs (Refs 29–34).These sources directly support claims about immune dysregulation, inflammatory cascades, and macrophage-driven matrix degradation.

5. Challenges in Targeted Delivery and Rationale for OMV-Based Platforms (Refs 35–47)Finally, for the sections on delivery barriers in the annulus fibrosus and the design of OMV-based delivery systems, we included:Reviews on anatomical complexity and permeability limits (Refs 35–40),Studies on siRNA delivery challenges (Ref 43),uthoritative reviews on OMV biology and engineering for drug delivery, including their immunogenicity risks and detoxification strategies (Refs 44–47).These citations ensure that our rationale for selecting an OMV-based, responsive nanotherapy platform is thoroughly and specifically supported.We have carefully double-checked that each citation is directly relevant and supports the associated text. Furthermore, all reference numbers have been updated accordingly in both the main text and the reference list.

Once again, we offer our sincere apologies for the confusion and inconvenience caused by our earlier referencing errors. We are truly grateful to the reviewer for pointing this out and for helping us enhance the scientific accuracy and integrity of our manuscript.

The Introduction and reference list have been revised and reorganized as in revised manuscript.

- Human IVD single cell sequencing. How many IVD specimens were used, how were the patients clinically treated, how was degeneration determined (Pfirrmann or Thompson) and what were the scores, was the entire IVD digested or was it separated in AF and NP tissue?

RE:Thank you for pointing this out, and this is an important question indeed.

Number of IVD Specimens Used

In our initial study, we performed single-cell RNA sequencing on three degenerated intervertebral disc (IVD) samples.To ensure greater scientific rigor, we deliberately included single-cell sequencing data from two additional patients, thereby strengthening the robustness and reliability of our study.In the current work, we have included two additional degenerated samples, and the findings remain consistent with our previous conclusions.In total, five human IVD specimens were collected and analyzed.All specimens were obtained from patients undergoing elective lumbar spine surgeries for degenerative spine diseases. Each disc specimen was used for subsequent single-cell RNA sequencing.This study was approved by the Institutional Review Board of Second Affiliated Hospital of Zhejiang University(approval number: 2024SQXB002446), and written informed consent was obtained from all participants.

Clinical Treatment of the Patients

All five patients had clear surgical indications including lumbar spinal stenosis, disc herniation, or spondylolisthesis, based on symptoms and imaging. Clinical treatment is summarized below(**Supplementary Fig. 1**):

Patient	Sex	Age	Diagnosis	Clinical Treatment (Surgery Type)	Tissue Site	Pfirrmann grade[1]
P1	Male	70	Lumbar spondylolisthesis, fracture	Posterior lumbar interbody fusion (L4/5), pedicle screw fixation	L4/5 IVD	IV
P2	Female	78	Lumbar stenosis & herniation	Lateral lumbar fusion and discectomy(L4/5,)	L4/5 IVD	IV
P3	Male	63	Lumbar stenosis	Posterior lumbar fusion (L4/5,L5/S1)	L4/5 , L5/S1 IVD	III
P4	Male	76	Lumbar spinal stenosis	Lateral lumbar interbody fusion (L3/4, L4/5), pedicle screw fixation	L3/4, L4/5 IVD	IV
P5	Female	67	Multilevel disc herniation and stenosis	Posterior fusion and decompression (L4/5)	L4/5 IVD	IV

Supplementary Fig. 1. Table(B)Clinical profiles of the corresponding patients, including sex, age, diagnosis, surgical procedure,

tissue collection site, and Pfirrmann grade based on MRI analysis. The Pfirrmann grading system classifies the degree of intervertebral disc degeneration from grade I (healthy) to grade V (severely degenerated).

All patients received general anesthesia, and treatment included perioperative antibiotics (e.g., cefuroxime, cefazolin), pain management, and neuroprotective strategies. Postoperative monitoring focused on pain control, wound healing, and thrombosis prevention.

Determination of Disc Degeneration

Disc degeneration was evaluated using preoperative MRI and CT imaging, confirming disc bulging, Modic changes, and vertebral endplate alterations. MRI showed multi-level disc bulging, L4/5 and L5/S1 herniation, and facet joint degeneration. The Pfirrmann grade was assigned based on MRI findings according to the classification system proposed in Magnetic resonance classification of lumbar intervertebral disc degeneration by Pfirrmann et al. (2001).

Tissue Handling

During surgery, intervertebral disc tissue was extracted under sterile conditions. Prior to enzymatic digestion, the annulus fibrosus (AF) and nucleus pulposus (NP) were not separated based on anatomical features or tissue texture; instead, the entire disc was digested as a whole.

We have re-analyzed our single-cell RNA-seq data and revised the description of these results in the manuscript accordingly. We have also added Supplementary Figure 1 to clearly present the updated findings. The revised results are in Page10 as follows:

"To investigate the cellular composition of the intervertebral disc (IVD), we collected degenerated disc samples from five patients aged between 65 and 80 years, whose representative magnetic resonance imaging (MRI) features are shown in Supplementary Fig. 1A. Detailed patient information, diagnosis, clinical treatment methods, and Pfirrmann grading are presented in Supplementary Fig 1B.Single-cell suspensions were prepared from the whole degenerated discs and subjected to single-cell RNA sequencing (scRNA-seq). Utilizing the 10x Genomics platform in combination with microfluidics, we successfully isolated individual cells and performed high-throughput sequencing using the Illumina platform. This enabled rapid and efficient transcriptomic profiling and downstream analysis of thousands of single cells at high resolution (Fig. 1A).

In total, we obtained transcriptomic profiles from 53,416 cells across five samples, with 9,751 to 11,532 cells captured per sample. The median number of detected genes per cell ranged from 1,350 to 2,438. After quality filtering, between 7,814 and 9,298 high-quality cells were retained per sample. Unsupervised clustering analysis identified 21 distinct cell populations. Based on canonical marker expression and transcriptional profiles, the IVD microenvironment was classified into ten major cell types: nucleus pulposus (NP) cells, annulus fibrosus (AF) cells, macrophages, endothelial cells, plasma cells, myofibroblasts, monocytes, neutrophils, B cells, and T cells(Fig. 1B). Notably, aside from NP and AF cells, macrophages accounted for a substantial proportion of cells (Fig. 1C), constituting approximately 12.27% of all immune cells (Fig. 1D).These findings provide a comprehensive single-cell landscape of the degenerated IVD, highlighting the cellular heterogeneity and implicating macrophages as a prominent immune cell population potentially involved in disc pathology."

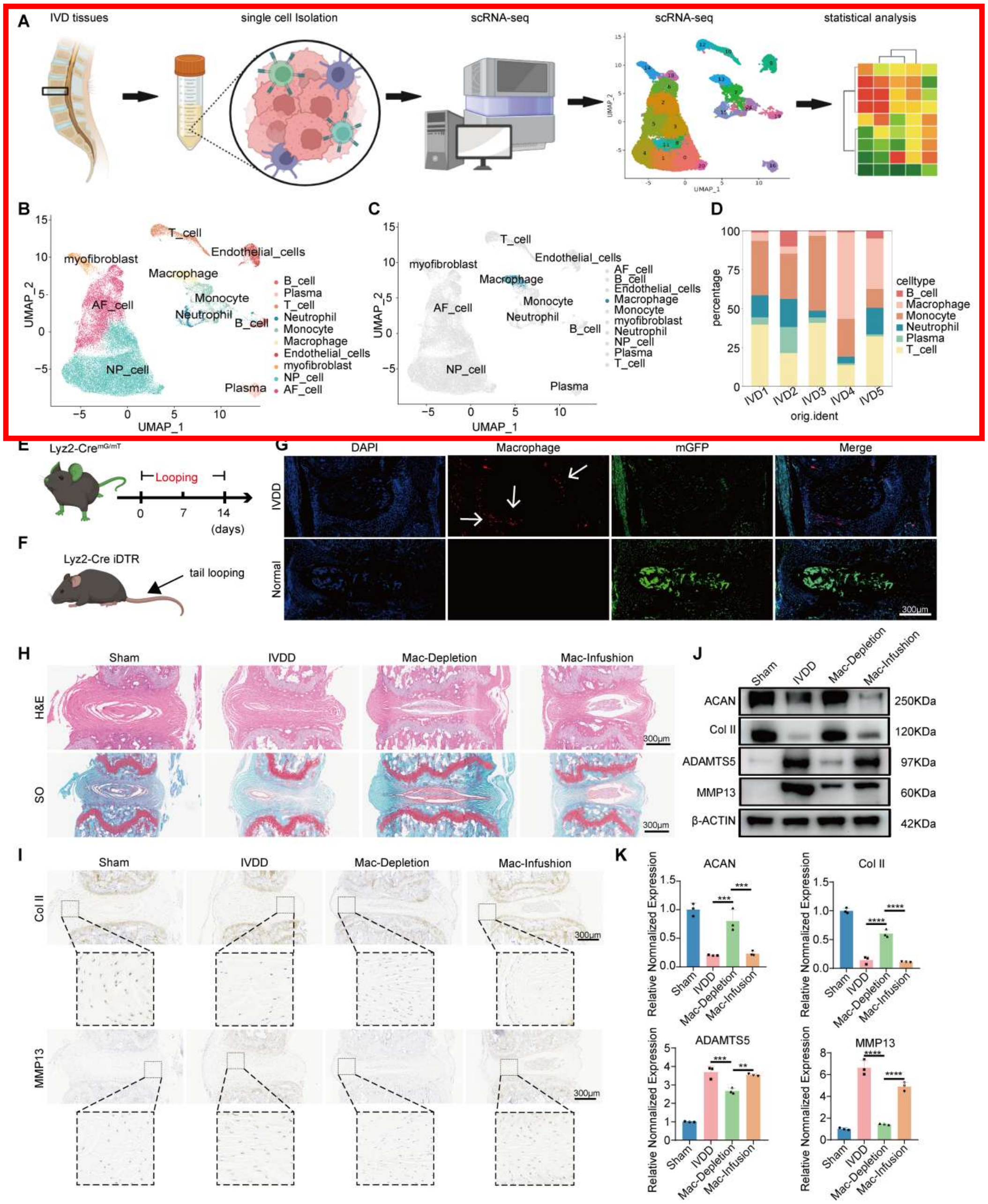

Figure 1. Macrophage Infiltration in Degenerating Intervertebral Discs.

(A) Schematic representation of human intervertebral disc single-cell sequencing.

References

1.Pfirrmann, C. W. A., Metzdorf, A., Zanetti, M., Hodler, J. & Boos, N. Magnetic resonance classification of lumbar intervertebral disc degeneration. Spine 26, 1873–1878 (2001).

-It is not clear whether the mouse models with macrophage-specific florescence and the one with macrophage-specific knock outs have been validated, so it is hard to understand whether the data obtained here with these animals are valid.

RE:Thank you very much for raising such a significant and insightful question. We sincerely appreciate your concern regarding the validation of the macrophage-specific knockout and reporter mouse models.

To directly address this concern, we have added a new figure -Supplementary Fig. 2 -specifically dedicated to presenting the genotyping results and phenotypic validation of our Lyz2-Cre-based models.This includes

confirmation of successful recombination in both the Lyz2-Cre;iDTR and Lyz2-Cre;mG/mT backgrounds.All relevant results are on Supplementary Fig. 2.

1.Genotypic validation (Supplementary Fig. 2.A-D):

We performed PCR-based genotyping using specific primer pairs for all alleles of interest. As shown in Supplementary Fig. 2. A and B, the primers and expected product sizes for DTR (mutant: 300 bp; WT: 603 bp), Lyz2-iCre (KI: 543 bp and 1413 bp; WT: 357 bp), and mG/mT (KI: 1465 bp; WT: 285 bp) are clearly defined.In Supplementary Fig. 2.C and D, we present representative gel electrophoresis results confirming the presence of the correct transgenic constructs:Lanes A1 and B1 show successful amplification of transgenic bands in Lyz2-iCre;DTR and Lyz2-iCre;mG/mT double transgenic mice, respectively.Negative and wild-type controls (Lanes A2, B2, WT, and N) exhibit expected absence or alternate bands.These results confirm accurate and reproducible genotyping of our experimental animals.

2.Phenotypic validation of macrophage-specific recombination (Supplementary Fig. 2.E):

To validate Cre recombinase activity driven by the Lyz2 promoter, we examined membrane GFP expression in Lyz2-iCre;mG/mT mice. As shown in Supplementary Fig. 2.E, strong GFP fluorescence is detected in macrophage-rich tissues such as ears, paws, tail, and skin as indicated by the red arrows. This expression pattern is consistent with previously reported Lyz2 promoter activity and supports Cre-mediated recombination in myeloid lineages.

3.Application in degeneration model (Supplementary Fig. 2. F–G):

The validated transgenic animals were used in a well-established tail-loop intervertebral disc (IVD) degeneration model[1-2], as illustrated in Supplementary Fig. 2.F. X-ray imaging (Supplementary Fig. 2. G) confirms successful surgical ligation and loop formation, ensuring consistent biomechanical stress-induced disc degeneration across experimental animals.

Together, these data verify that both the macrophage-specific knockout (Lyz2-iCre;DTR) and reporter (Lyz2-iCre;mG/mT) lines are correctly genotyped and phenotypically validated, supporting the reliability and reproducibility of our in vivo findings.

We have revised the description of our results in Page12 as follows:

"To further explore the role of macrophages in degenerative disc disease, we generated a macrophage-specific fluorescent reporter mouse model (Lyz2-Cre;mG/mT), in which macrophages express membrane-localized tdTomato (red fluorescence), while all other cell types express membrane-localized GFP (green fluorescence), thereby allowing specific visualization of macrophages (Fig. 1E). To validate the genetic background of the model, genotyping by PCR was performed and confirmed the presence of both Lyz2-iCre and mG/mT alleles (Supplementary Fig. 2B, D). Strong GFP fluorescence was observed in the ears, paws, tail, and skin, consistent with Cre activity under the control of the Lyz2 promoter (Supplementary Fig. 2E),as indicated by the red arrows. Lyz2-Cre;mG/mT mice were divided into two groups: a normal control(Normal) group without tail-loop surgery and a tail-loop induced intervertebral disc degeneration (IVDD) group. The tail-loop model and its validation are shown in Supplementary Fig. 2F–G,and the modeling process was maintained for two weeks. Notably, minimal macrophage infiltration was observed in healthy IVDs, whereas prominent tdTomato-positive macrophages were detected within degenerated discs two weeks post tail-loop induction (Fig. 1G).Using a macrophage-specific fluorescent reporter mouse model (Lyz2-Cre;mG/mT), we demonstrated that macrophage infiltration is markedly increased in intervertebral discs following tail-loop-induced disc degeneration, in contrast to the minimal presence observed in healthy discs. This model enables precise in vivo visualization of macrophages and provides a robust platform for investigating immune cell dynamics during disc degeneration.

To investigate the functional relevance of macrophages during IDD progression, we established a macrophage-depletion mouse model (Lyz2-Cre;iDTR) that allows selective ablation of macrophages via diphtheria toxin (DT) administration (Fig. 1F). Genotyping confirmed the successful generation of Lyz2-Cre;iDTR double-positive mice (Supplementary Fig. 2A, C). Mice were divided into four experimental groups:Sham:wild-type (WT) mice without tail looping；IVDD:WT mice with tail-loop induced disc degeneration；Mac-Depletion:Lyz2-Cre;iDTR mice receiving DT-mediated macrophage ablation followed by tail looping, and Mac-Infusion:Lyz2-Cre;iDTR mice subjected to macrophage depletion and tail looping, followed by macrophage infusion (2 μL of freshly isolated bone marrow–derived macrophages [BMDMs], ~2×10^5 cells, microinjected into the AF and NP region on postoperative day 3 using a 34G microsyringe)."

A

Primer Name	Sequence(5'- 3')	PCR size	Primer illustration
oIMR8052	GCGAAGAGTTTGTCCTCAACC	Mutant = 300bp<br>Wild type = 603bp	DTR
oIMR8545	AAAGTCGCTCTGAGTTGTTAT		
oIMR8546	GGAGCGGGAGAAATGGATATG		

Lyz2-iCre-tF1	AGTGCTGAAGTCCATAGATCGG	KI：543bp	Lyz2-iCre
Lyz2-iCre-tR1	CTGATTCTCCTCATCACCAGG		
Lyz2-iCre-tF1	AGTGCTGAAGTCCATAGATCGG	KI：1413bp	
Lyz2-iCre-tR2	GTCACTCACTGCTCCCCTGT	Wt：357bp	

B

Primer Name	Sequence(5'- 3')	PCR size	Primer illustration
H11-tF2	ATGCCCACCAAAGTCATCAGTGTAG	KI:1465bp	mG/mT
H11-CAG-5tR2	AGGCGGGCCATTTACCGTAAGTTA		
H11-tF3	GGGCAGTCTGGTACTTCCAAGCT	WT:285bp	
H11-tR3	ATATCCCCTTGTTCCCTTTCTGC		

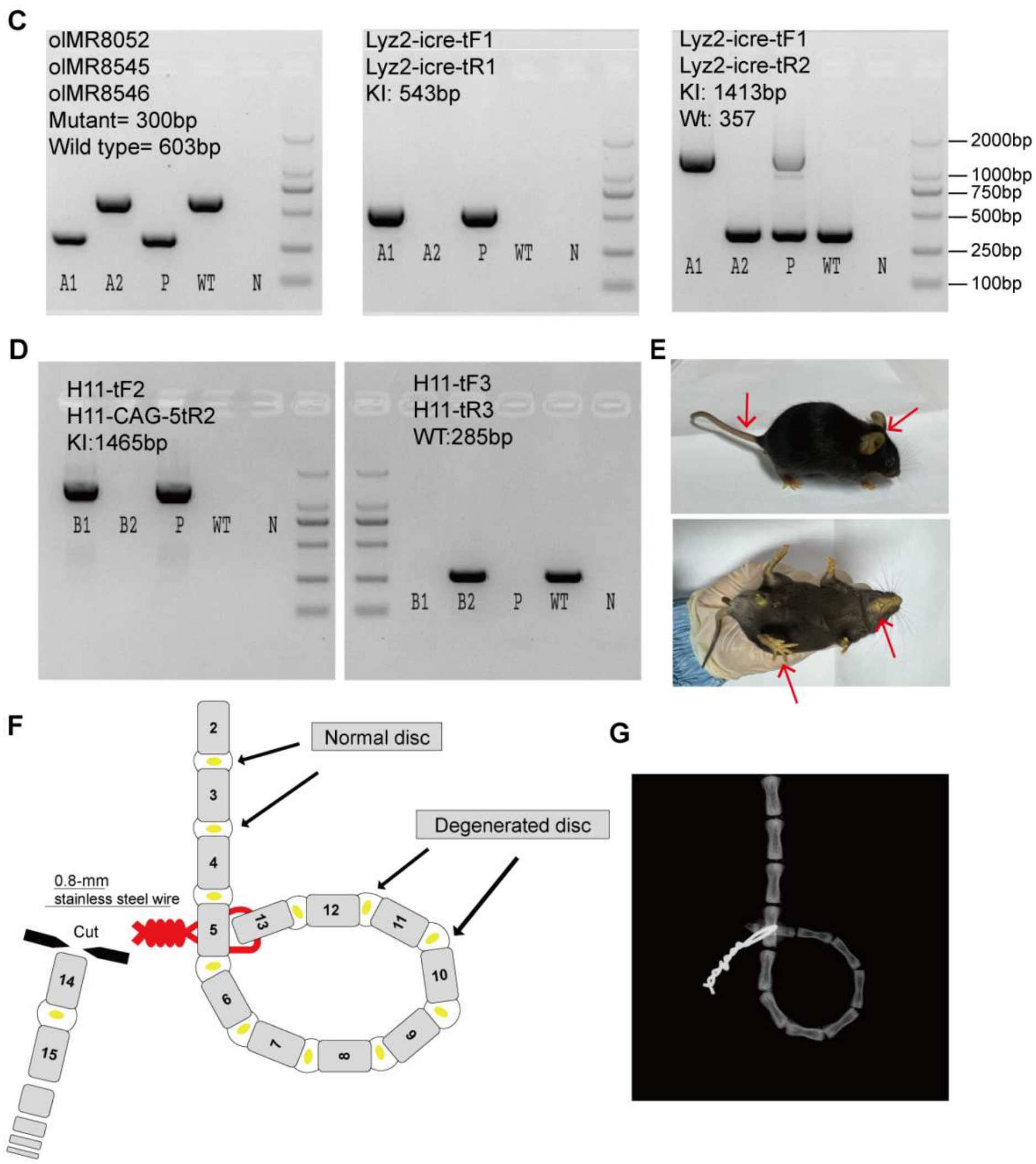

Supplementary Fig. 2.Genotyping of transgenic mice and establishment of tail-loop intervertebral disc degeneration model.

(A-B)Primer sequences used for genotyping DTR (A) and mG/mT (B) mouse lines crossed with Lyz2-iCre. PCR product sizes and genotyping strategy are indicated.

(C)PCR results for Lyz2-iCre and DTR transgenic alleles. Lane A1: Lyz2-iCre;DTR double transgenic mouse (KI/mutant),Lane A2: wild-type (WT) control,Lane P: positive control,Lane WT: WT DNA control,Lane N:No-Template Control.Expected bands: 300 bp for mutant DTR, 603 bp for WT; 543 bp and 1413 bp for KI Lyz2-iCre, 357 bp for WT.

(D)PCR genotyping of Lyz2-iCre crossed with mG/mT mice.Lane B1: Lyz2-iCre;mG/mT double transgenic mouse (KI),Lane B2: wild-type (WT) control,Lane P: positive control,Lane WT: WT DNA control,Lane N: No-Template Control.Expected bands:

1465 bp for mG/mT KI, 285 bp for WT.

(E) Gross appearance of a Lyz2-CremG/mT double transgenic mouse,as indicated by the red arrows. Strong GFP fluorescence is visible in the ears, paws, tail, and skin, consistent with Lyz2 promoter-driven Cre activity and membrane-localized GFP reporter expression.

(F) Schematic illustration of the tail-loop disc degeneration model. A stainless steel wire (0.8 mm diameter) is used to ligate vertebrae 5–13, creating mechanical stress on enclosed intervertebral discs (segments 6–12), which develop degeneration over time. Discs outside the loop serve as non-degenerate controls.

(G) X-ray radiograph confirming the closed-loop configuration of the tail vertebrae following wire ligation.

References

1.Sakai, D. et al. Migration of bone marrow-derived cells for endogenous repair in a new tail-looping disc degeneration model in the mouse: a pilot study. Spine J. ***15****, 1356–1365 (2015).*

2.Xie, W. et al. A mouse coccygeal intervertebral disc degeneration model with tail-looping constructed using a suturing method. Anim. Model. Exp. Med. ***8****, (2025).*

-The fact that macrophages were depleted does not mean they were directly involved in IVD degeneration. I assume Lyz2 is also expressed in osteoclasts and hence the adjacent bone may have changed, known to be able to cause degeneration of adjacent cartilaginous structures. Replenishing them exogenously to the IVD could have shown a direct effect.

RE:We sincerely appreciate your insightful comments regarding the potential involvement of osteoclasts due to Lyz2 expression and the need to demonstrate a direct link between macrophages and intervertebral disc (IVD) degeneration.

To address this concern and strengthen the mechanistic connection between macrophages and IVD pathology, we have expanded our study from the original three groups to now include a fourth experimental group and to make it easier to distinguish between groups, we renamed each group:

Sham: Wild-type (WT) mice without tail-loop surgery;IVDD: WT mice subjected to tail-loop surgery;Mac-Depletion: Lyz2-Cre;iDTR mice with diphtheria toxin (DT)-induced macrophage depletion and tail-loop surgery.Mac-Infusion: Lyz2-Cre;iDTR mice receiving macrophage infusion directly into the IVD following tail-loop surgery.

Specifically, 2 μL of freshly isolated bone marrow–derived macrophages (BMDMs), containing approximately 2×10^5 cells, were microinjected into the nucleus pulposus area of each degenerated disc on postoperative day 3 using a 34G microsyringe . This approach allowed us to locally replenish macrophages within the degenerated microenvironment, thereby testing their functional necessity and sufficiency.

Following this intervention, we performed comprehensive histological and imaging analyses:Hematoxylin and eosin (HE), safranin-O (SO), and immunohistochemistry were repeated on IVD tissues to evaluate morphological restoration and inflammation.

To directly address your concern regarding potential off-target depletion of osteoclasts, we conducted TRAP staining on vertebrae adjacent to the degenerated discs(Supplementary Fig. 4.A). TRAP-positive multinucleated osteoclasts were sparse across all groups, including the Mac-Depletion and Mac-Infusion groups, suggesting that the Lyz2-Cre;iDTR model did not induce substantial osteoclast ablation.

Additionally, micro-CT analysis of the adjacent vertebral bodies(Supplementary Fig. 4.B) showed no significant differences in percent bone volume (BV/TV) or bone mineral density (BMD) among groups(Supplementary Fig. 4.C-D). These findings indicate that vertebral bone mass and structure were preserved, and that potential osteoclast depletion did not lead to detectable bone changes that could secondarily influence disc degeneration.

Together, these data provide strong support for a direct role of macrophages in modulating IVD homeostasis and degeneration. The rescue of disc structure following macrophage infusion strongly argues for their functional involvement, while the absence of osteoclastic alterations further confirms that the observed disc phenotypes are not secondary to bone loss.

We have incorporated these new experimental details and findings into the revised manuscript (in Page15 and Supplementary Fig. 4.):

“To rule out the possibility that the observed disc phenotypes were caused by off-target depletion of osteoclasts due to Lyz2 expression, we further examined the vertebrae adjacent to the degenerated discs. TRAP staining was performed to detect osteoclast activity (Supplementary Fig. 4A). The number of TRAP-positive multinucleated osteoclasts remained comparably low across all groups, including Mac-Depletion and Mac-Infusion, suggesting that the Lyz2-Cre;iDTR model did not induce substantial osteoclast ablation.In addition, micro-computed tomography (Micro-CT) analysis was conducted to evaluate structural integrity of the adjacent vertebral bone (Supplementary Fig. 4B). Quantitative analysis revealed no significant differences in bone volume fraction (BV/TV) or bone mineral density (BMD) among the experimental groups (Supplementary Fig. 4C–D).These findings confirm that vertebral bone mass and osteoclast activity were not significantly affected by macrophage

depletion, thereby excluding indirect bone-driven effects on disc degeneration. Together with the structural rescue observed upon macrophage infusion, our data provide compelling evidence that macrophages directly regulate IVD degeneration."

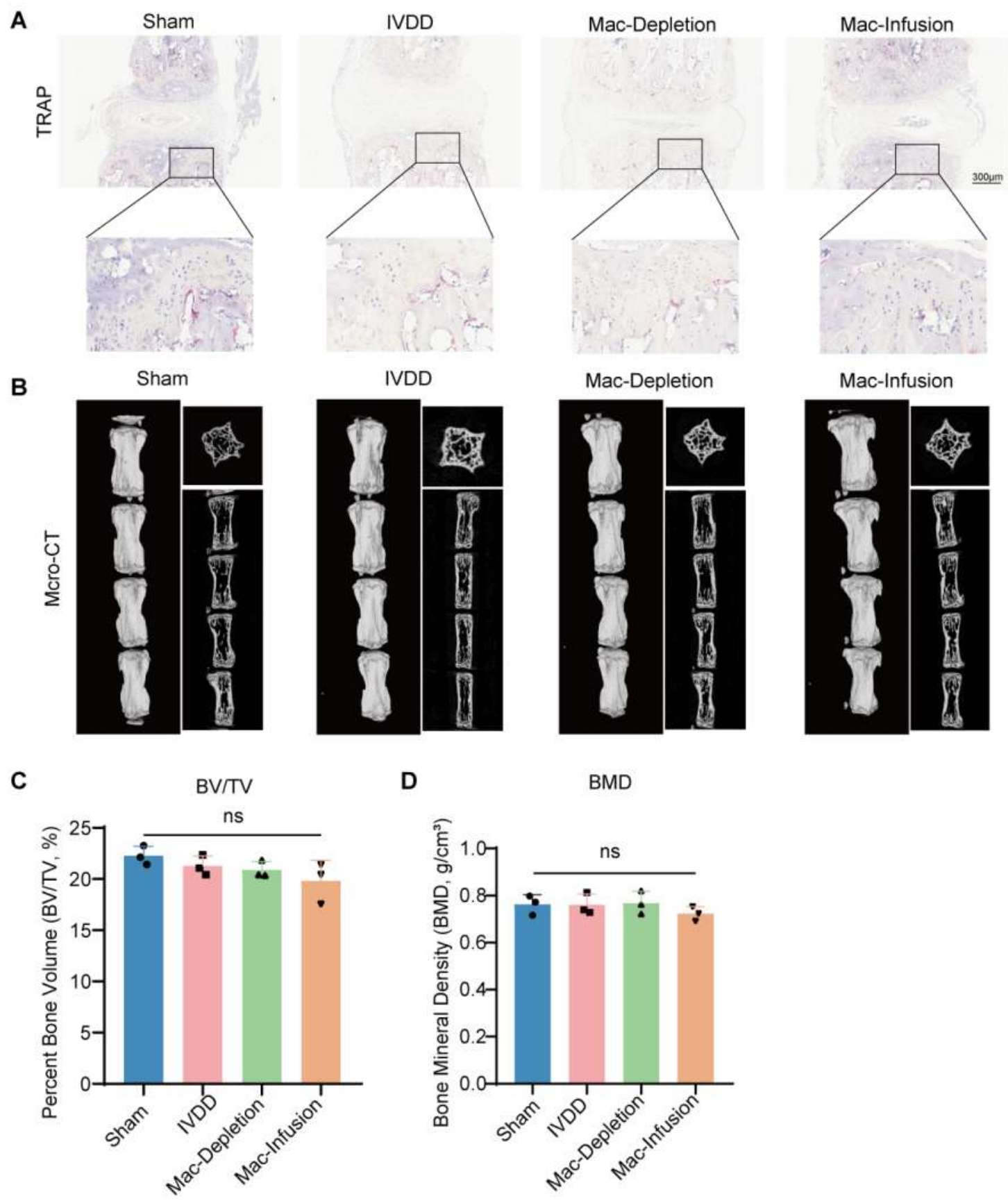

Supplementary Fig. 4.Analysis of osteoclast activity and vertebral bone integrity in a mouse model of intervertebral disc degeneration (IVDD) with macrophage depletion(lyz2-Cre iDTR) and infusion.

-Why would MMP-13 expression by macrophages only affect the AF and not the NP tissue?

RE：We appreciate your thoughtful question regarding the tissue specificity of MMP-13 effects in our study. We would like to clarify that the primary focus of our current research was on the annulus fibrosus (AF) tissue, rather than the nucleus pulposus (NP). As such, our experimental design, analysis, and interpretation of results were directed specifically at understanding the role of macrophages and MMP-13 within the AF compartment of the intervertebral disc (IVD).

In our updated study (see Figure 1), we employed single-cell RNA sequencing (scRNA-seq) on human IVD tissues to profile the cellular landscape during degeneration. Clustering analysis revealed distinct populations including AF cells, NP cells, and macrophages, among others (Figure 1B, 1C). Notably, we identified a marked infiltration and enrichment of macrophages in degenerative disc samples (Figure 1D), prompting further investigation into their role within the AF region.

To specifically examine the localization and function of macrophages, we used Lyz2-Cre;mG/mT reporter mice and observed macrophage infiltration predominantly in AF tissues, as shown by immunofluorescent staining (Figure 1F). This spatial pattern provided strong rationale for focusing on AF tissue.

Additionally, the macrophage depletion model (Lyz2-Cre;iDTR) showed significant preservation of AF structure and reduction in MMP-13 expression following macrophage ablation (Figures 1J–K). Importantly, while some effects on NP may not be excluded, our histological and molecular evaluations—both immunostaining and protein quantification—were performed in AF-rich regions of the IVD. Thus, our findings on MMP-13 reflect changes primarily in AF tissue.

We acknowledge that NP tissue may also be affected by inflammatory mediators in broader contexts. However, this aspect was beyond the scope of the present investigation. Future studies will aim to dissect the differential

impact of macrophage-derived MMP-13 on both NP and AF regions.We have clarified this focus in the revised manuscript and updated the corresponding figure legends and methods to avoid any potential confusion in Page14 as follows：

"To further investigate the impact of macrophage depletion or supplementation on disc matrix metabolism, we specifically dissected the annulus fibrosus (AF) tissue from mouse intervertebral discs for molecular analysis. Western blotting and quantitative PCR were subsequently performed to assess the expression of key anabolic and catabolic matrix markers(Fig 1J-K). At the protein level (Fig. 1J), ACAN and Col II were significantly downregulated in the IVDD group compared to the Sham group, while ADAMTS5 and MMP13 were markedly upregulated, indicating matrix degradation. Notably, these degenerative changes were further exacerbated in the Mac-Infusion group following macrophage supplementation. In contrast, macrophage depletion (Mac-Depletion group) resulted in partial restoration of ACAN and Col II levels and suppression of ADAMTS5 and MMP13 expression, suggesting a protective effect of macrophage ablation.Quantitative protein data are presented in Supplementary Fig. 3D–G.This pattern was further validated at the transcriptional level (Fig. 1K). Quantitative PCR showed that ACAN and Col II mRNA expression was elevated in the Mac-Depletion group compared to the IVDD group, while the expression of ADAMTS5 and MMP13 was reduced. Conversely, macrophage supplementation again led to enhanced catabolic marker expression and reduced matrix synthesis genes.Collectively, these results demonstrate that macrophage accumulation exacerbates disc degeneration, while selective depletion of macrophages protects disc matrix integrity—highlighting a detrimental role of macrophages in the progression of intervertebral disc degeneration."

Figure 1J

- The change in mRNA expression between degenerated and degenerated-macrophage depleted for ACAN, COLII and MMP13 is way less than the 2-fold usually taken as meaningful in expression analysis.

RE:We really appreciate your insightful comment. We agree that the differences in mRNA expression levels of ACAN, COLII, and MMP13 in Figure 1J appear modest between the degenerated and macrophage-depleted groups when assessed using total intervertebral disc (IVD) tissue.

However, we would like to clarify that the initial qPCR analysis was performed using RNA extracted from the whole IVD tissue, which includes both the nucleus pulposus (NP) and annulus fibrosus (AF) regions, as well as adjacent cartilaginous endplates. These heterogeneous tissue components can introduce substantial variability and dilute the transcriptional signal of specific genes expressed predominantly in the AF region. Given that macrophages are mostly enriched in the AF and that this region exhibits stronger ECM remodeling responses, we reasoned that whole-tissue profiling might mask biologically relevant gene expression dynamics in this context.

To address this, we have isolated the AF region from each sample and conducted an independent validation by quantitative PCR. The results from the AF-isolated samples demonstrate more pronounced changes in mRNA expression levels of ACAN, Col II, and MMP13 between the degenerated and macrophage-depleted IVD groups, which now exceed the 2-fold threshold commonly considered biologically significant. This revised analysis supports our original conclusion that macrophage depletion alleviates matrix degradation and promotes anabolic ECM remodeling in the degenerated IVD.

We have included these new data in the revised manuscript as Figure 1 K and updated the relevant sections in the Results and Methods accordingly(Page14).

"To further investigate the impact of macrophage depletion or supplementation on disc matrix metabolism, we specifically dissected the annulus fibrosus (AF) tissue from mouse intervertebral discs for molecular analysis. Western blotting and quantitative PCR were subsequently performed to assess the expression of key anabolic and catabolic matrix markers(Fig 1J-K). At the protein level (Fig. 1J), ACAN and Col II were significantly downregulated in the IVDD group compared to the Sham group, while ADAMTS5 and MMP13 were markedly upregulated, indicating matrix degradation. Notably, these degenerative changes were further exacerbated in the Mac-Infusion group following macrophage supplementation. In contrast, macrophage depletion (Mac-Depletion group) resulted in partial restoration of ACAN and Col II levels and suppression of ADAMTS5 and MMP13 expression, suggesting a protective effect of macrophage ablation.Quantitative protein data are presented in Supplementary Fig. 3D–G.This pattern was further validated at the transcriptional level (Fig. 1K). Quantitative PCR showed that ACAN and Col II mRNA expression was elevated in the Mac-Depletion group compared to the IVDD group, while the expression of ADAMTS5 and MMP13 was reduced. Conversely, macrophage supplementation again led to enhanced catabolic marker expression and reduced matrix synthesis genes.Collectively, these results demonstrate that macrophage accumulation exacerbates disc degeneration, while selective depletion of macrophages protects disc matrix integrity—highlighting a detrimental role of macrophages in the progression of intervertebral disc degeneration."

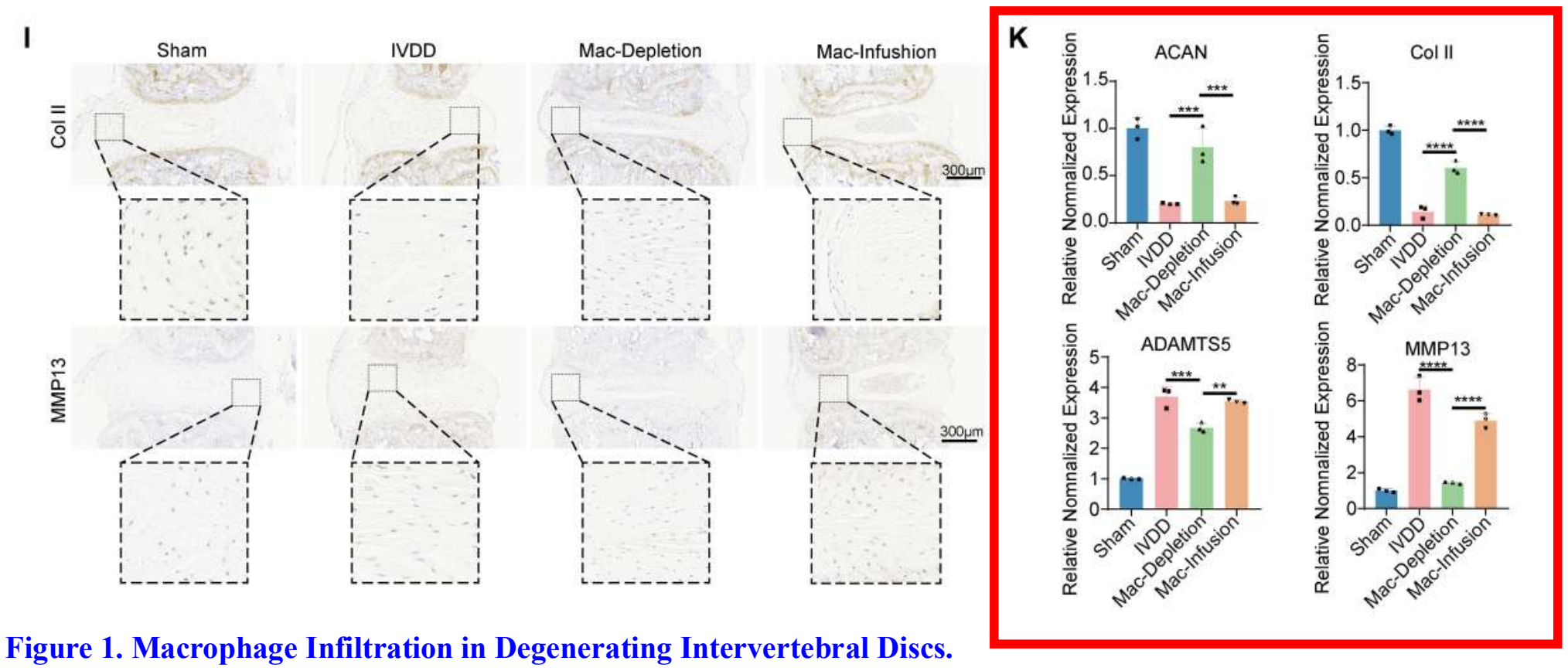

Figure 1. Macrophage Infiltration in Degenerating Intervertebral Discs.
(K) qRT-PCR analysis of ACAN, Col II, ADAMTS5, and MMP13 gene expression in annulus fibrosus tissue of intervertebral discs from different intervention groups.

- Protein (Western Blot/immunohistochemistry) quantification is missing.
RE:We sincerely thank you for this valuable comment. We fully acknowledge the importance of including quantitative analyses for immunohistochemical and Western blot results to ensure the rigor and reproducibility of our findings.
In response to this suggestion, we have now performed detailed quantification of protein expression levels from both immunohistochemistry and Western blot assays Based on our new grouping. The updated data are presented in the revised manuscript as shown in **Supplementary Fig. 3 B–G.**

- Grading and subsequent quantitative analysis of degeneration is lacking, also further on in the MS
RE：Thank you for this important comment. In response, we have now clearly incorporated a validated histological grading system for intervertebral disc degeneration in mice(Figure 1. H). Specifically, we applied the Masuda scoring system, a well-established semi-quantitative method that evaluates nucleus pulposus integrity, annulus fibrosus structure, and overall disc morphology on a scale from 0 (normal) to 12 (severely degenerated) [1-5].
We have updated the Methods section to describe the scoring process in detail, including blinding and sample size. In the Results section, we now report the quantitative grading data (mean ± SD), together with appropriate statistical analyses (one-way ANOVA with Tukey's post-hoc test). This information is also summarized in the Supplementary Fig. 3, which shows individual scores, group means, and statistical significance annotations.The subsequent quantification and grading of degeneration in the manuscript have also been done more.

References

1.Masuda, K., Aota, Y., Muehleman, C. et al. A novel rabbit model of mild, reproducible disc degeneration by an anulus needle puncture: correlation between the degree of disc injury and radiological and histological appearances of disc degeneration. Spine (Phila Pa 1976) ***30****, 5–14 (2005).*

2.Norcross, J.P., Lester, G.E., Weinhold, P. & Dahners, L.E. An in vivo model of degenerative disc disease. J. Orthop. Res. ***21****, 183–188 (2003).*

3.Tam, V. et al. Histological and reference system for the analysis of mouse intervertebral disc. J. Orthop. Res. ***36****, 233–243 (2018).*

4.Melgoza, I.P. et al. Development of a standardized histopathology scoring system using machine learning algorithms for intervertebral disc degeneration in the mouse model—An ORS spine section initiative. JOR Spine ***4****, e1164 (2021).*

5.Tang, S.N. et al. In vivo mouse intervertebral disc degeneration models and their utility as translational models of clinical discogenic back pain: A comparative review. Front. Pain Res. ***3****, 894651 (2022).*

6.Baldia, M. et al. Development of a unique mouse intervertebral disc degeneration model using a simple novel tool. Asian Spine J. ***15****, 415–423 (2021).*

- Figure 3 It is not clear how the liposomal formulations were administered. As systemic administration of nanoparticles is known to generally only reach tissues with fenestrated capillaries such as the liver, but not structures like the IVD, I assume these were injected intradiscally. However, as this should be a relatively contained space, I wonder why without targeting, fast loss of signal was observed as they are unlikely to have diffused out? Moreover, there was no direct comparison with targeted liposomes, so could the injected quantity have been different between the 2 experiments?
RE:We sincerely appreciate your insightful comment and the opportunity to clarify the experimental details.
Route of Administration:We apologize for not clearly specifying this in the initial submission. As correctly inferred by the reviewer, all liposomal formulations (including both non-targeted and targeted) were administered via intradiscal injection into the nucleus pulposus under direct microscopic visualization. This detail has now been explicitly included in both the Methods section and figure legends.
Rapid Signal Decline of Non-Targeted Liposomes:We agree with the reviewer that the IVD is typically a relatively

enclosed and avascular structure. However, it is important to note that the degenerative IVD environment in our model is not entirely intact. First, intradiscal injection itself, even when performed carefully, is known to cause mild mechanical disruption and local inflammation, which has been previously reported to alter the disc microenvironment and permeability. This local inflammatory response may lead to neovascular infiltration and micro-hemorrhage, temporarily increasing fluid exchange or immune cell activity in the region[1-7].

Moreover, in our IVDD model, the disc integrity is already compromised, with structural breakdown of the extracellular matrix and annular fissures—conditions that may allow faster clearance or diffusion of non-targeted liposomes out of the nucleus pulposus. This compromised anatomical barrier likely accounts for the observed rapid decrease in fluorescent signal within 72 hours post-injection in the non-targeted group. We have now added these points to the revised Discussion section and cited relevant supporting literature.

Injection Dose and Comparison with Targeted Liposomes:We appreciate the reviewer's concern regarding consistency. We confirm that the same injection volume and liposomal concentration were used across all groups to ensure comparability. Although we originally omitted imaging data of the targeted liposomes, we have now included these results in Supplementary Fig. 5. (new panel) for direct comparison. The enhanced retention observed with the targeted liposomes further supports their improved binding affinity within the disc microenvironment.

We thank you again for highlighting these important issues, which have helped us significantly improve the clarity and robustness of the manuscript.Our changes are in Page18 as follows:

"The intervertebral disc (IVD), the largest avascular organ in the human body, currently lacks effective targeted therapies. Conventional liposomes, though widely used as drug delivery carriers, exhibit poor retention and limited therapeutic efficacy in the IVD due to its unique anatomical and biological barriers. To evaluate this limitation, we employed an acupuncture-induced intervertebral disc degeneration (IVDD) model in 8-week-old rats, randomly assigning them to Sham (mock surgery), PBS, or conventional liposome treatment groups.After 8 weeks, the animals were euthanized, and disc tissues were collected for histological analysis using hematoxylin and eosin (H&E) staining (Figure 3B). The severity of disc degeneration was quantitatively assessed using the Masuda scoring system (Supplementary Fig. 5A). To evaluate the targeting capacity and retention behavior of conventional liposomes in vivo, we labeled them with the near-infrared fluorescent dye DiR and performed fluorescence imaging. As shown in Fig. 3C, conventional (non-targeted) liposomes exhibited no significant difference in fluorescence distribution compared to free DiR dye (Supplementary Fig. 5B), indicating minimal targeting capability. By 72 hours post-injection, the fluorescence signal in the disc region had nearly diminished to the detection limit (Supplementary Fig. 5C), suggesting rapid clearance of the liposomes from the intervertebral disc. These findings underscore the necessity for engineering liposomal systems with enhanced tissue specificity and prolonged retention to overcome the delivery challenges posed by the disc microenvironment."

References

1.Cunha, C., Silva, A. J., Pereira, P. et al. Inflammatory response in lumbar disc herniation: an immunohistochemical and gene expression study. Arthritis Res. Ther. ***20****, 251 (2018).*

2.Lama, P., Le Maitre, C. L., Dolan, P. et al. Do intervertebral discs degenerate before or after herniation? J. Bone Joint Surg. Br. ***95****, 1127–1133 (2013).*

3.Petrosyan, E., Fares, J., Lesniak, M. S. et al. Biological principles of adult degenerative scoliosis. Trends Mol. Med. ***29****, 740–752 (2023).*

4.Zhang, S., Hu, B., Liu, W. et al. Structural and functional changes of the sensory nervous system in discogenic low back pain. Osteoarthritis Cartilage ***29****, 17–27 (2021).*

5.Hu, Y. et al. The emerging roles of nanocarrier drug delivery systems in treatment of intervertebral disc degeneration. Drug Des. Dev. Ther. ***18****, 1007–1022 (2024).*

6.Pereira, D. R. et al. Drug delivery in intervertebral disc degeneration and osteoarthritis: Selecting the optimal platform. J. Control Release ***328****, 985–999 (2020).*

7.Xu, C. et al. Liposomes for drug delivery by localized routes of administration. in Lipid-Based Nanocarriers 259–281 (Elsevier, 2023).

-"We..co-cultured peptide 1-Cy5.5 with AF cells, cartilage endplate cells, and chondrocytes." Why not with NP cells? Endplate cells áre chondrocytes, and hence it is not surprising the response is the same.

RE:We sincerely thank you for this valuable observation.

We fully acknowledge that excluding nucleus pulposus (NP) cells in the initial co-culture experiments limited the interpretive value of our peptide specificity assessment. As the reviewer rightly pointed out, cartilage endplate cells and chondrocytes are phenotypically similar, and therefore insufficient to validate the cell-type selectivity of peptide 1 (P1).

In response, we have now expanded the experiment to include NP cells in the comparative analysis. We evaluated the binding behavior of Cy5.5-labeled P1 with AF, NP, and EP cells under the same conditions (10 μM incubation, 30 min), and quantified the relative fluorescence intensity. The results, now included in Figure 3H and 3J, clearly

demonstrate that P1 binds strongly and selectively to AF cells, while NP and EP cells show minimal signal. This confirms the specificity of P1 for AF cells, strengthening its candidacy as a targeted delivery ligand.

We appreciate your critical input, which led to a more comprehensive and scientifically rigorous evaluation of P1 binding specificity.The corresponding results are presented on Page21 as follow:

"To validate the cell-type specificity of peptide 1 (P1), we co-cultured Cy5.5-labeled P1 with annulus fibrosus (AF) cells, nucleus pulposus (NP) cells, and cartilage endplate (EP) cells. As shown in Figure 3H, P1 displayed robust binding to AF cells, whereas minimal fluorescence was observed in NP and EP cells. Quantitative analysis of fluorescence intensity (Figure 3J) confirmed the selective affinity of P1 for AF cells, supporting its potential as a targeting ligand for annulus fibrosus tissue."

- The control peptide seems not to be a scrambled version of peptide 1. This is a serious omission.

RE：We sincerely thank you for this important and careful observation.

We acknowledge that in our initial submission, we did not explicitly define the scrambled control peptide and its relationship to peptide 1 (P1), which may have caused confusion. To address this, we have now clearly clarified in the Supplementary Materials and Methods that the control peptide used in our validation experiments is a sequence-scrambled version of P1 with the following amino acid sequence: YLNSYSFAGKPT. This scrambled sequence retains the same amino acid composition as P1 but with randomized residue order, and no predicted structural motifs or binding domains.

This control peptide was used in all specificity validation experiments both in vitro and in vivo, including the data shown in Supplementary Figure 6. As expected, the scrambled P1 exhibited minimal binding to AF cells and was rapidly cleared in vivo, in contrast to the prolonged retention and strong fluorescence of the original P1.

We have revised the manuscript to explicitly state the control peptide's sequence and design rationale. We sincerely apologize for this oversight and thank the reviewer for pointing it out, which allowed us to reinforce the scientific rigor of our study.The updated results are presented in the revised Supplementary Fig. 6, and we added a corrected part in Page22 as follow:

"To rigorously assess the targeting specificity of peptide 1 (P1), we synthesized a scrambled version of the peptide (control sequence: YLNSYSFAGKPT) and evaluated its binding behavior in both in vitro and in vivo models. In cultured annulus fibrosus (AF) cells, we incubated Cy5.5-labeled P1, scrambled P1, and a no-peptide control at 10 μM for 30 minutes, followed by imaging via confocal microscopy (Supplementary Fig. 6A). Fluorescent signals revealed that P1 showed strong surface binding and cytoplasmic accumulation in AF cells, whereas the scrambled sequence and dye-only controls resulted in weak or negligible fluorescence, indicating minimal non-specific uptake.To quantitatively compare targeting performance, we measured the relative fluorescence intensity in each group (Supplementary Fig. 6D). P1-treated cells exhibited a significantly higher mean fluorescence intensity than both scrambled P1 and no-peptide controls ($p < 0.0001$), demonstrating the sequence-dependent specificity of P1 binding to AF cells.

Next, we performed in vivo validation using a rat intradiscal injection model. Cy5.5-labeled P1, scrambled P1, and free dye were injected into the intervertebral discs, and fluorescent retention was longitudinally monitored using small animal imaging (Supplementary Fig. 6B). While all three groups showed comparable initial signals at 1 hour, signal from scrambled P1 and free dye diminished rapidly after 24–48 hours. In contrast, P1 remained detectable in the disc space up to 10 days post-injection, with a fluorescence intensity of ~34. units at 240 hours (Supplementary Fig. 6E), confirming its superior in vivo retention and targeting stability.

We further investigated whether P1 could recognize AF cells across different species. Confocal microscopy after incubation with Cy5.5-labeled P1 showed robust binding to rat, human, and mouse AF cells (Supplementary Fig. 6C). Quantitative analysis (Supplementary Fig. 6F) revealed that although signal intensity was highest in rat cells, human and mouse AF cells also demonstrated consistent uptake, supporting the cross-species translational potential of P1 as an AF-targeting moiety.

Together, these results provide compelling evidence that P1 exhibits high affinity and specificity for annulus fibrosus cells in a sequence-dependent, cell-type–specific, and cross-species–compatible manner. Its prolonged in vivo retention and negligible off-target accumulation further support its application as a promising delivery vehicle for targeted therapeutic strategies in intervertebral disc degeneration."

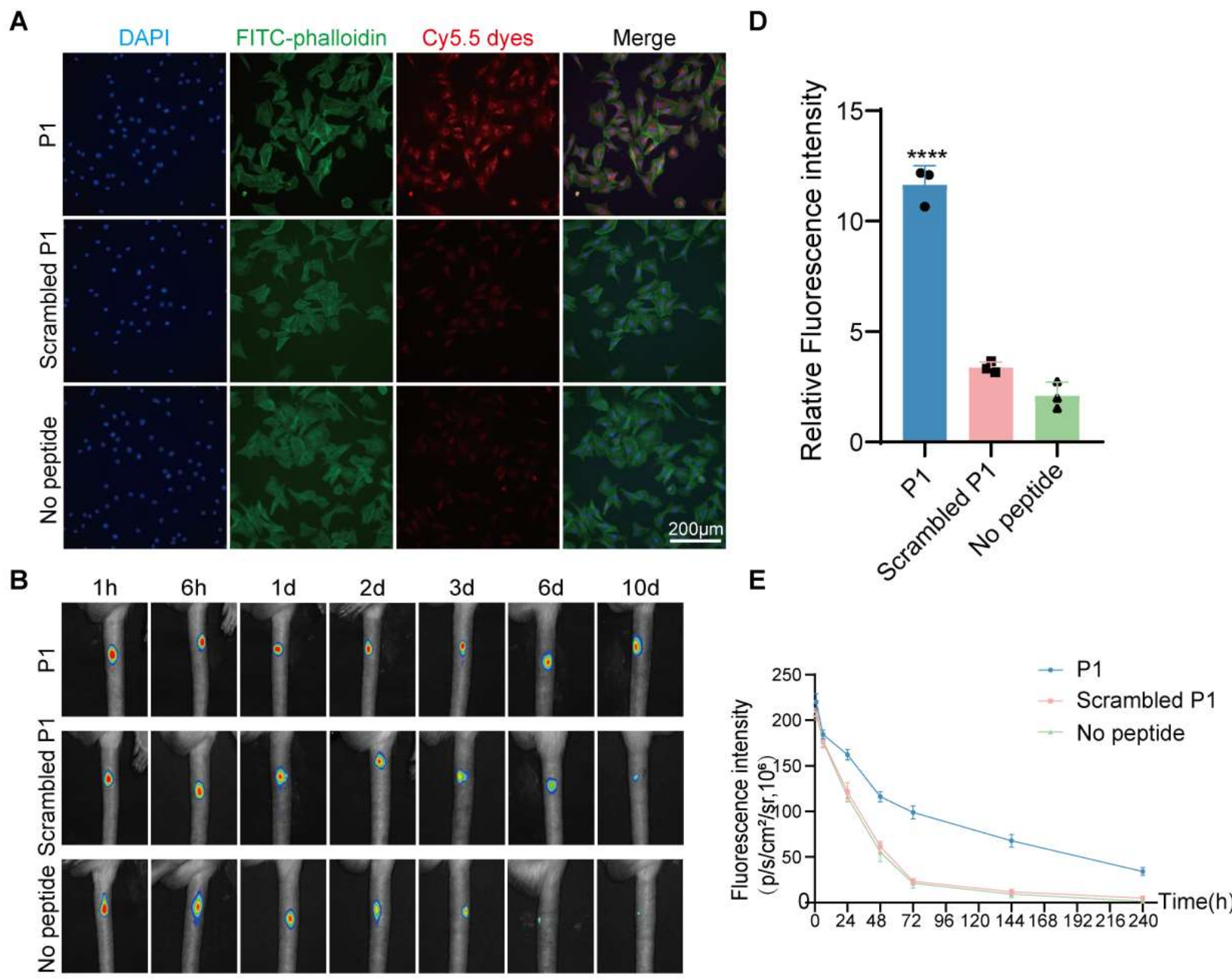

Supplementary Fig. 6.In vitro and in vivo evaluation of the binding affinity and specificity of P1 and scrambled P1.

(A) Confocal microscopy images showing the binding of P1，scrambled P1 and No peptide (Cy5.5-labeled) with FITC-labeled AF cells after incubation at 10 µM for 30 minutes. DAPI was used for nuclear staining.

(B) In vivo fluorescence imaging of the accumulation of P1，scrambled P1 and No peptide (Cy5.5-labeled) in the rat intervertebral discs at different time points (1h, 6h, 1d, 2d, 3d, 6d, 10d) post-injection.

(C) Confocal microscopy demonstrating the specificity of P1 (Cy5.5-labeled) with different cell types, including Rat AF cells, Human AF cells, and Mouse AF cells after incubation at 10 µM for 30 minutes.

(D) Quantification of relative fluorescence intensity in AF cells treated with P1, scrambled P1, or no peptide.

(E) Fluorescence intensity of P1，scrambled P1 and no peptide in rat intervertebral discs over time (1h to 240h) post-injection.

(F) Quantification of relative fluorescence intensity of P1 in Rat, Human, and Mouse AF cells. Data are presented as mean ± SD from at least three independent experiments. *$p < 0.05$, **$p < 0.01$, ***$p < 0.001$.

- Figure 3K shows retention, not accumulation

RE:We sincerely thank you for this accurate and important observation.

We fully acknowledge that our original description of Figure 3K(now updated as Figure 3G) inaccurately referred to the data as representing accumulation, when in fact it more precisely reflects retention of the fluorescently labeled peptides over time following intradiscal injection. We apologize for this oversight in terminology.

In response, we have carefully revised the corresponding text in the Results section to reflect the correct interpretation. Specifically, we now state in Page21:

“Next, we assessed the in vivo targeting ability of the candidate peptides using a needle injection approach. Peptide-Cy5.5 conjugates and free Cy5.5 dye were intradiscally injected into rat intervertebral discs, and the retention of fluorescent signal was monitored using the AniView multi-modal in vivo imaging system (BLT Biotech) (Figure 3G). Fluorescence intensity was recorded at multiple time points: 1 hour, 6 hours, 24 hours, 2 days, 3 days, 6 days, and 10 days post-injection.At the initial time point, peptides 1 through 5 exhibited comparable fluorescence intensities (average:172.31). However, by day 3 (72 hours), the signals from peptides 2 through 5 had largely diminished (fluorescence intensity <5), whereas peptide 1 maintained a detectable signal even on day 10, with a remaining intensity of 29.67 (Figure 3K).In summary, peptide 1 exhibited superior targeting specificity for AF cells, both in vitro and in vivo, with sustained retention in the intervertebral disc.”

This correction has also been applied to the figure legend and associated narrative, ensuring clarity and precision throughout.We are grateful to you for pointing out this important detail, which has improved the accuracy of our manuscript.

- Authors should show by microscopical analysis of cryosections of the injected discs that actually the peptide was retained in the AF rather than the NP

RE:We sincerely thank you for this important and scientifically valid suggestion.

In response, we conducted an additional set of ex vivo fluorescence microscopy experiments using cryosections of

rat intervertebral discs following intradiscal injection of DIR-labeled AF-PTK@LEV@SPP1. To capture the in situ distribution of the injected particles over time, we sacrificed the animals at 0.5 hours, 1 day, 3 days, and 7 days post-injection. The harvested lumbar discs were immediately embedded and snap-frozen in liquid nitrogen, followed by cryosectioning. Each section underwent DAPI staining (10 min) for nuclear visualization and was imaged using an inverted fluorescence microscope under consistent exposure parameters.

As presented in Figure 6C, red fluorescence corresponding to AF-PTK@LEV@SPP1 was widely distributed throughout both the annulus fibrosus (AF) and nucleus pulposus (NP) regions at 0.5 hours post-injection. By day 1, the signal became concentrated in the AF, with an overall increase in intensity, suggesting active localization or diffusion toward the fibrous region. On day 3 and day 7, we observed persistent and localized fluorescence primarily in the AF, with markedly reduced signal in the NP region, confirming that the injected material was retained in the AF over time.

To quantify this distribution, we defined two regions of interest (ROI) corresponding to AF and NP, and analyzed mean fluorescence intensity (MFI) over the four time points (Figure 6C,F). The results demonstrated that although both regions initially showed signal, the fluorescence in the NP declined sharply after day 1, whereas the AF retained high levels of signal through day 7. Statistical analysis using two-way ANOVA with Bonferroni correction showed significant differences in MFI between AF and NP regions at each time point ($p < 0.0001$).

These new data confirm the reviewer's hypothesis and provide direct histological evidence that AF-PTK@LEV@SPP1 is selectively retained in the annulus fibrosus rather than the nucleus pulposus. We have added these data as Figure 6, and updated the corresponding descriptions in the manuscript and figure legends.

We are deeply grateful to the reviewer for encouraging us to perform this additional and important analysis, which has helped us strengthen the conclusions of our study.

The updated results are presented in the revised Figure 6C, and we added a corrected part in Page32 as follow:

"To determine in vivo localization and retention characteristics, we injected DiR-labeled AF-PTK@LEV@SPP1 into the intervertebral discs of rats and performed frozen-section imaging at 0.5 hours, 1 day, 3 days, and 7 days post-injection. Fluorescence signals were initially observed in both the AF and NP regions at 0.5 hours; however, as time progressed, signals became increasingly restricted to the AF region. By day 7, distinct retention was evident in AF tissue, while fluorescence in NP regions diminished to background levels (Figure 6C). Quantitative comparisons between AF (region I) and NP (region II) zones showed significantly higher fluorescence in AF at each time point (Figure 6F), indicating selective accumulation and prolonged tissue retention in the annulus fibrosus.."

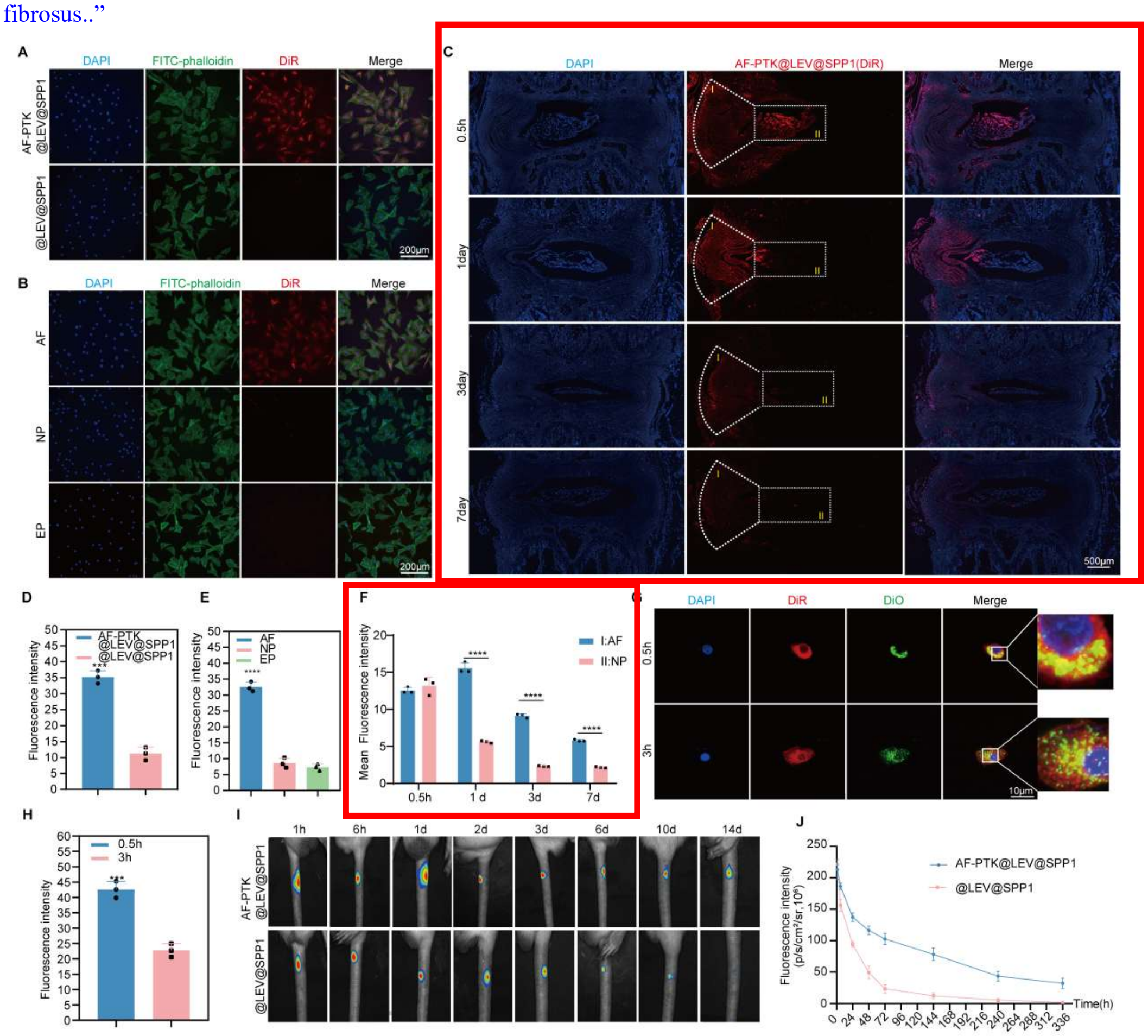

Figure 6.In vitro and in vivo fluorescence imaging and quantification of AF-PTK@LEV@SPP1 binding in cultured cells and frozen tissue sections of rat intervertebral discs.

(A) Confocal microscopy showing the affinity of AF-PTK@LEV@SPP1 (DiR-labeled) with FITC-phalloidin-labeled AF cells after incubation at 10 μM for 30 minutes. Scale bar = 200 μm.

(B)Confocal microscopy demonstrating the specificity of AF-PTK@LEV@SPP1. AF-PTK@LEV@SPP1 (DiR-labeled) incubated with FITC-phalloidin-labeled AF cells, NP cells and cartilage endplate cells at 10 μM for 30 minutes. Scale bar = 200 μm.

(C)Representative fluorescence images of frozen tissue sections from rat intervertebral discs at different time points (0.5 hours, 1 day, 3 days, and 7 days) after the injection of AF-PTK@LEV@SPP1(DiR-labeled).Scale bar = 200 μm.

(D) Fluorescence intensity of AF cells stained with DIR-labeled AF-PTK@LEV@SPP1.

(E) Fluorescence intensity of AF-PTK@LEV@SPP1 with AF cells, NP cells and cartilage endplate cells.

(F)Quantification of mean fluorescence intensity in the frozen tissue sections at different time points (0.5 hours, 1 day, 3 days, and 7 days) following AF-PTK@LEV@SPP1 injection, for two experimental groups: I:AF (blue bars) and II:NP (pink bars).

(G) Confocal microscopy showing the binding of AF-PTK@LEV@SPP1 with annulus fibrosus cells at 0.5h and 3h. DiR-labeled annulus fibrosus cells and DiO-labeled AF-PTK@LEV@SPP1.

(H) Fluorescence intensity of AF-PTK@LEV@SPP1 binding with annulus fibrosus cells at 0.5h and 3h.

(I) In vivo fluorescence imaging of rat intervertebral discs at 1 h to 14 days post-injection with DiR-labeled AF-PTK@LEV@SPP1.

(J) Fluorescence intensity of AF-PTK@LEV@SPP1 and @LEV@SPP1 in rat intervertebral discs. Data were analyzed using one-way ANOVA. Values represent the mean ± SD from at least three independent experiments.* indicates $p < 0.05$, ** indicates $p < 0.01$, *** indicates $p < 0.001$, **** indicates $p < 0.0001$.

- Specificity of the binding of the AF-peptide liposomes should be confirmed again in AF, NP and CEP cell culture, as conformation changes may have occurred losing specificity. Comparisons should be made between binding peptide, scrambled (and therefore nonbinding) peptide and no peptide.

RE:We sincerely thank you for raising this important point regarding the potential loss of binding specificity due to conformational changes upon liposomal formulation.

To directly address this concern, we conducted additional in vitro binding assays using DIR-labeled AF-PTK@LEV@SPP1 and evaluated their interaction with cultured annulus fibrosus (AF), nucleus pulposus (NP), and cartilage endplate (EP) cells. As shown in Figure 6 B and E, the AF-PTK@LEV@SPP1 formulation exhibited significantly higher fluorescence intensity in AF cells compared to NP and EP cells under identical incubation conditions (10 μM, 30 min), confirming the retained specificity of the targeting peptide after liposomal conjugation. In contrast, DIR signal in NP and EP cells remained low, comparable to background levels.

To further strengthen our conclusions, in a separate experiment (Supplementary Fig. 6), we also compared peptide 1 with its scrambled version (nonbinding sequence: YLNSYSFAGKPT) and a no-peptide control. These controls showed minimal binding in AF cells, both in vitro and in vivo, further validating that the observed binding by AF-PTK@LEV@SPP1 was due to sequence-specific targeting rather than non-specific adsorption.

These results demonstrate that the AF-binding peptide maintains its specificity after conjugation to liposomes, and that the formulation effectively distinguishes between AF, NP, and EP cell types in culture. We have added these new data and corresponding descriptions to the revised supplementary figures and manuscript.

We are grateful to you for suggesting this important control, which significantly improved the robustness and clarity of our findings.And the corresponding results are presented on Page31 as follow:

“We next assessed the cell-type specificity of AF-PTK@LEV@SPP1 by comparing its binding performance across three major disc-derived cell types: AF cells, nucleus pulposus (NP) cells, and cartilage endplate (EP) cells. After 30-minute incubations, confocal microscopy revealed that the fluorescence signal was predominantly localized within AF cells, while NP and EP cells exhibited only minimal signal (Figure 6B). Quantification supported these observations, showing significantly elevated fluorescence in AF cells(32.52 ± 1.53) compared to NP (8.60 ± 1.62) and EP (7.30 ± 1.04) groups (Figure 6E, $p < 0.0001$). These results highlight the strong selectivity of the targeting construct toward annulus fibrosus tissue

.-Figure 5B. How do authors distinguish fibroblasts from macrophages in the 2D culture and thereby the transfer of the particles from one cell type to another after ROS activation?

RE：We sincerely thank you for this insightful question regarding cell-type distinction and particle transfer dynamics in our 2D co-culture assay.

To address this, we established a co-culture model consisting of annulus fibrosus (AF) cells and macrophages, and tracked the localization of our DIR-labeled therapeutic construct (AF-PTK@LEV@SPP1) before and after ROS stimulation. The following experimental design was used to clearly differentiate cell types and monitor nanoparticle transfer:

Macrophage labeling: Prior to co-culture, macrophages were selectively stained with FITC (green), which specifically labels these cells and distinguishes them from AF cells.

Nuclear counterstaining: All cells (AF and macrophages) were stained with DAPI (blue) to visualize nuclei and assist in spatial orientation.

Nanoparticle tracking: The engineered delivery system, AF-PTK@LEV@SPP1, was labeled with DIR (red) to track its localization.

As shown in Figure 5B:At baseline (Row 1), DIR fluorescence is localized within AF cells, with no overlap with FITC-labeled macrophages.At 1 hour post-treatment (Row 2), we introduced low-dose(100 μ M) H_2O_2 to mimic a ROS-elevated microenvironment, facilitating cleavage of the thioketal (TK) linker within the AF-targeting construct.At 3 hours (Row 3), we observed a progressive shift in DIR fluorescence, showing decreased signal in AF cells and increased colocalization with FITC-positive macrophages, indicating ROS-triggered release and uptake by macrophages.

This spatiotemporal redistribution supports our dual-targeting strategy: initial binding to AF cells, followed by ROS-responsive release and phagocytic uptake by macrophages.

We have revised the figure legend and Results section to clarify this process and highlight the method used to distinguish between the two cell types. We are grateful to you for prompting this clarification, which has improved the clarity of our mechanistic interpretation.The updated data was presented in Figure 5, and the corresponding results are presented on Page27 as follow:

"To elucidate the dynamic phagocytic behavior of AF-PTK@LEV@SPP1 nanoparticles and their role in intercellular signal transmission within the degenerative disc microenvironment, we employed a two-dimensional co-culture model composed of annulus fibrosus (AF) cells and bone-marrow-derived M1 macrophages (BMDM-M1). To enable clear cell-type distinction and precise tracking of nanoparticle transfer, we applied a three-channel fluorescence labeling strategy: macrophages were pre-labeled with FITC (green), all nuclei were counterstained with DAPI (blue), and AF-PTK@LEV@SPP1 nanoparticles were tagged with DiR (red).At baseline (Fig. 5B, Row 1), DiR fluorescence was exclusively localized within AF cells, with no overlap with FITC-labeled macrophages, confirming initial cell-specific targeting. Upon introduction of 100 μM H_2O_2 for 1 hour to mimic elevated ROS levels (Row 2), we observed partial redistribution of the DiR signal. By 3 hours post-stimulation (Row 3), DiR fluorescence had significantly diminished in AF cells and increased within FITC-positive macrophages, indicating ROS-induced cleavage of the thioketal linker and subsequent release and phagocytic uptake of the nanoparticles by macrophages.These findings provide strong evidence for the sequential, ROS-responsive delivery mechanism of our engineered nanosystem—initial targeting of AF cells followed by secondary re-targeting to macrophages—supporting its potential to bridge intercellular communication during disc degeneration."

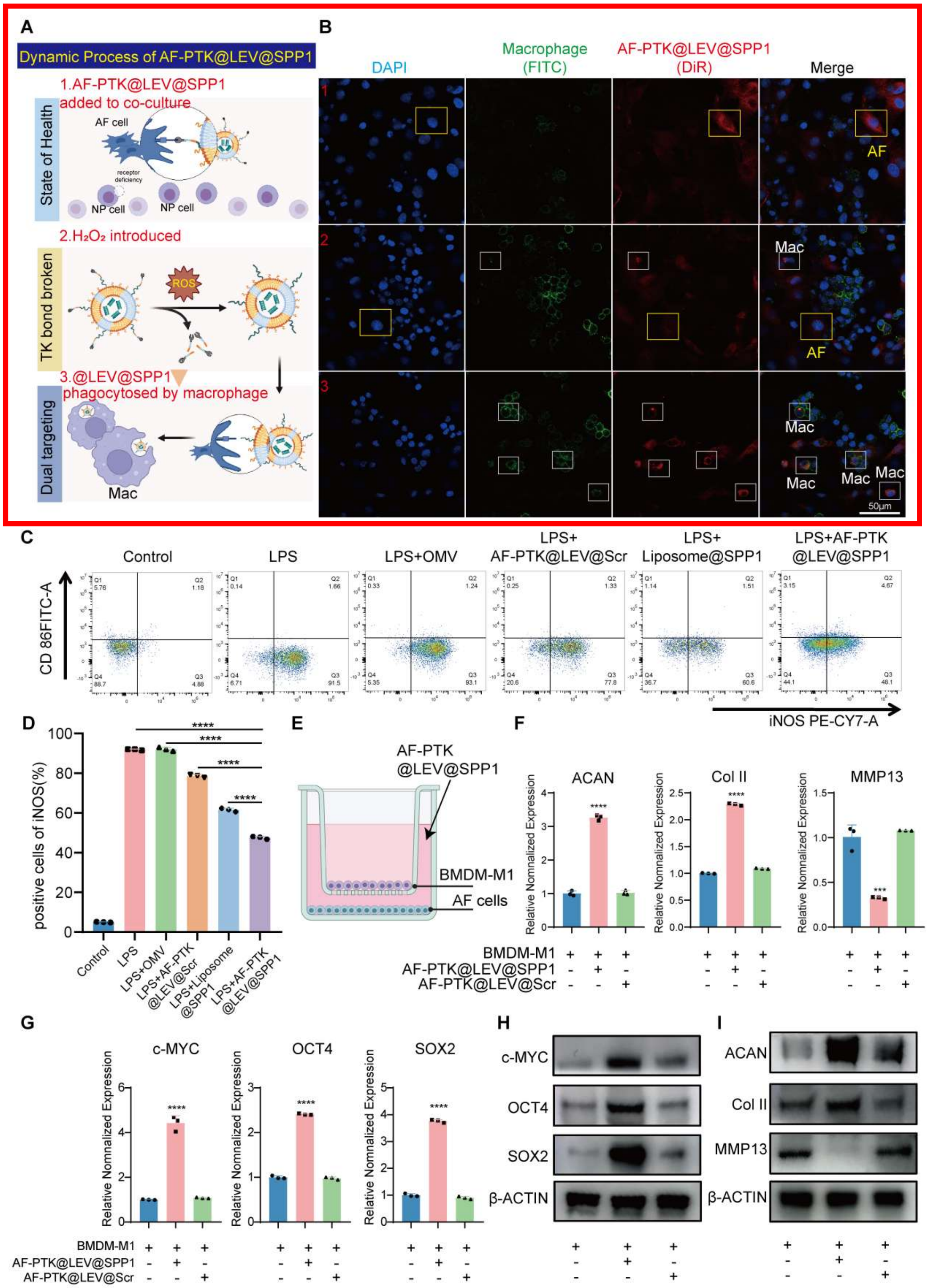

Figure 5. Dynamic phagocytosis of AF-PTK@LEV@SPP1 and its inhibition of M1 macrophage polarization to improve intervertebral disc degeneration.

(A) Schematic illustration of the dynamic phagocytosis process of AF-PTK@LEV@SPP1 by annulus fibrosus cells and bone-marrow-derived M1 macrophages (BMDM-M1) in sequence.

(B) onfocal microscopy showing the phagocytosis of AF-PTK@LEV@SPP1: nanoparticles were initially taken up by AF cells and subsequently engulfed by macrophages after co-culture. DAPI (nuclei, blue), FITC (macrophages, green), and DiR (AF-PTK@LEV@SPP1, red). Scale bar = 50 μm.

(C) Flow cytometric analysis of macrophages stained for CD86 and iNOS under different treatment conditions.

(D) Quantification of iNOS-positive macrophages based on flow cytometry from (C).

(E) Schematic diagram of the Transwell co-culture system with BMDM-M1 and AF cells exposed to different treatments.

(F, G)Quantitative real-time PCR analysis of AF cell gene expression, including aggrecan (ACAN), collagen type II (Col II), matrix metalloproteinase 13 (MMP13), and stemness-associated genes (OCT4,SOX2, c-MYC), following indirect exposure to treated BMDMs.

(H, I) Western blot analysis of the indicated proteins in AF cells co-cultured with BMDMs pretreated as shown.

Data are presented as mean ± SD from at least three independent experiments. Statistical significance was assessed using one-way ANOVA with appropriate post hoc tests. * indicates $p < 0.05$, ** indicates $p < 0.01$, *** indicates $p < 0.001$, **** indicates $p < 0.0001$.

- For the data in Figure 5C, it seems no scrambled siRNA was used. This control is essential.

RE:We sincerely thank you for these insightful and important comments regarding the completeness of our experimental design and the necessity of appropriate control groups.

We fully acknowledge that in our initial study, the experimental grouping in Figure 5C was insufficient, particularly lacking two essential controls:A scrambled siRNA version of AF-PTK@LEV@SPP1 to verify sequence-specific effects of SPP1 silencing;A liposome@SPP1 (without AF targeting peptide) group to assess whether liposomal delivery of SPP1 alone can suppress LPS-induced M1 polarization of macrophages.

In response, we have redesigned and expanded the experimental groups, and the updated flow cytometry results are now shown in the revised Figure 5C (bottom panel, 6 groups total), with corresponding quantification in Figure 5D.

Specifically, we added:

LPS+AF-PTK@LEV@Scr: A scrambled siRNA control using a non-targeting sequence incorporated into the same nanocarrier (AF-targeted liposome) to ensure that the observed immunomodulatory effect was specific to SPP1 silencing.

LPS+Liposome@SPP1: A non-targeted liposome loaded with SPP1 siRNA (no AF peptide modification) to assess whether SPP1 knockdown alone, without annulus fibrosus targeting, can sufficiently inhibit macrophage M1 activation.

Our new results demonstrate that:The scrambled siRNA group (AF-PTK@LEV@Scr) did not reduce iNOS expression in LPS-stimulated macrophages, confirming the sequence specificity of the observed immunomodulatory effect.

The liposome@SPP1 group showed partial inhibition of macrophage M1 polarization, but to a significantly lesser extent compared to AF-PTK@LEV@SPP1, indicating that AF-targeting contributes significantly to efficacy, likely by improving microenvironmental retention and delivery efficiency.These additional groups now strengthen the mechanistic interpretation of our results and address both reviewers' concerns. We have also revised the figure legend and Results section accordingly.

We are grateful to you for these constructive suggestions, which helped us improve the scientific rigor and interpretability of our findings.The modifications are shown in Page28 as follows:

“To assess the regulatory effects on macrophage polarization, bone-marrow-derived macrophages (BMDMs) were divided into six groups: Control (no LPS stimulation), LPS (100 ng/mL), LPS + OMV, LPS + AF-PTK@LEV@Scr (Scr denotes a scrambled SPP1-siRNA; sense 5′-GAUGACGUACCUCGGAUUA-dTdT, antisense 5′-UAAUCCGAGGUACGUCAUC-dTdT-3′), LPS + Liposome@SPP1, and LPS + AF-PTK@LEV@SPP1. Flow cytometric analysis showed that LPS stimulation markedly elevated the expression of iNOS in BMDM-M1 , a typical M1 macrophage marker, with over 90% of cells being iNOS-positive. Treatment with AF-PTK@LEV@SPP1 significantly reduced this proportion to approximately 48%, which was substantially lower than in the AF-PTK@LEV@Scr (77.8%) and Liposome@SPP1 (60.6%) control groups (Fig. 5C–D). These results indicate that AF-PTK@LEV@SPP1 effectively inhibits the pro-inflammatory M1 polarization of macrophages.”

- In the legend for 5H, it says “Representative immunohistochemical staining for AGG, MMP13, SOX2, OCT4, and c-MYC in intervertebral discs of macrophage-specific knockout mice after different treatments” However, these figures show WB results and there are only 2 conditions tested.

RE:We sincerely thank you for carefully pointing out this inconsistency between the figure legend and the actual data presented in Figure 5H.

This was indeed a mistake in labeling. The correct description should read:“(Figure 5.H, I) Western blot analysis of the indicated proteins in AF cells co-cultured with macrophages pretreated as shown.”We apologize for this oversight in manuscript preparation.

Furthermore, in response to the reviewer’s observation regarding the limited number of experimental conditions, we have restructured and expanded the co-culture experiment. We now include additional relevant treatment groups (Figure 5E-I) to better evaluate the downstream molecular effects of different formulations on AF cells. These modifications are now reflected in the revised Figure 5H and 5I, as well as in the updated Results section

and figure legends.
We sincerely thank you again for bringing this to our attention, and we believe these corrections and additions have improved the accuracy and scientific value of our work.
The modifications to the figure legend are in Page29 as follows:
“To further assess how macrophage polarization states influence AF cells, we employed a Transwell-based co-culture system (Fig. 5E) involving three groups: pro-inflammatory BMDM-M1 alone, pro-inflammatory BMDM-M1 with AF-PTK@LEV@Scr, and pro-inflammatory BMDM-M1 with AF-PTK@LEV@SPP1. Co-culture with inflammatory macrophages led to a marked reduction in aggrecan (ACAN) and collagen type II (Col II) expression in AF cells, accompanied by a significant increase in matrix metalloproteinase 13 (MMP13), indicating extracellular matrix degradation. Notably, treatment with AF-PTK@LEV@SPP1 restored ACAN and Col II expression and suppressed MMP13 upregulation, suggesting that this nanoplatform protects the matrix function of AF cells by modulating macrophage phenotype. In contrast, AF-PTK@LEV@Scr failed to exert a similar effect (Fig. 5F, I).Comprehensive quantitative analyses of protein levels are provided in Supplementary Fig. 7A.
Moreover, we observed that exposure to pro-inflammatory BMDM-M1 downregulated the expression of stemness-related genes c-MYC,OCT4 , and SOX2 in AF cells. This abnormal expression profile was not significantly altered by AF-PTK@LEV@Scr treatment. However, AF-PTK@LEV@SPP1 reversed the trend, leading to a significant upregulation of these stemness markers, suggesting that the system helps maintain the stem-like properties of AF cells under inflammatory conditions (Fig. 5G–H).Comprehensive quantitative analyses of protein levels are provided in Supplementary Fig. 7B.
In summary, AF-PTK@LEV@SPP1 not only enables ROS-responsive intercellular nanoparticle trafficking, but also alleviates intervertebral disc degeneration at multiple levels by suppressing M1 macrophage polarization and improving the functional status of AF cells.”

-By the way, in the manuscript, the authors refer to fig 6F-I, but these show fluorescence intensity after binding of the peptide-conjugated versus unconjugated liposomes in vitro and in vivo
RE:We sincerely thank you for carefully identifying this figure citation error in our manuscript.
As correctly pointed out, the results we described regarding gene and protein expression changes in annulus fibrosus (AF) cells following macrophage co-culture with AF-PTK@LEV@SPP1-treated macrophages were mistakenly referred to as Figures 6F–I in the original text.The correct citation should be Figures 5F–I, which show the results of qPCR and Western blot analyses evaluating aggrecan (ACAN), MMP13, SOX2, OCT4, and c-MYC in AF cells. These figures correspond to the downstream effects of AF-PTK@LEV@SPP1 treatment in the co-culture system described in the paragraph.
We have corrected this error in the Results section of the revised manuscript to ensure accurate figure referencing and improve clarity. We sincerely apologize for the oversight and greatly appreciate your attention to detail.And we added a corrected part in Page29 as follow:
“To further assess how macrophage polarization states influence AF cells, we employed a Transwell-based co-culture system (Fig. 5E) involving three groups: pro-inflammatory BMDM-M1 alone, pro-inflammatory BMDM-M1 with AF-PTK@LEV@Scr, and pro-inflammatory BMDM-M1 with AF-PTK@LEV@SPP1. Co-culture with inflammatory macrophages led to a marked reduction in aggrecan (ACAN) and collagen type II (Col II) expression in AF cells, accompanied by a significant increase in matrix metalloproteinase 13 (MMP13), indicating extracellular matrix degradation. Notably, treatment with AF-PTK@LEV@SPP1 restored ACAN and Col II expression and suppressed MMP13 upregulation, suggesting that this nanoplatform protects the matrix function of AF cells by modulating macrophage phenotype. In contrast, AF-PTK@LEV@Scr failed to exert a similar effect (Fig. 5F, I).Comprehensive quantitative analyses of protein levels are provided in Supplementary Fig. 7A.
Moreover, we observed that exposure to pro-inflammatory BMDM-M1 downregulated the expression of stemness-related genes c-MYC,OCT4 , and SOX2 in AF cells. This abnormal expression profile was not significantly altered by AF-PTK@LEV@Scr treatment. However, AF-PTK@LEV@SPP1 reversed the trend, leading to a significant upregulation of these stemness markers, suggesting that the system helps maintain the stem-like properties of AF cells under inflammatory conditions (Fig. 5G–H).Comprehensive quantitative analyses of protein levels are provided in Supplementary Fig. 7B.
In summary, AF-PTK@LEV@SPP1 not only enables ROS-responsive intercellular nanoparticle trafficking, but also alleviates intervertebral disc degeneration at multiple levels by suppressing M1 macrophage polarization and improving the functional status of AF cells.”

- Stemness as demonstrated by higher OCT4, SOX2 and c-myc seems to be opposite to increase in aggrecan expression

RE:We sincerely thank you for this insightful comment regarding the potential divergence between increased stemness and aggrecan expression in our co-culture system.
In our experimental system—where annulus fibrosus (AF) cells were indirectly modulated by preconditioned proinflammatory macrophages—the cellular state is more dynamic than a simple stem/differentiation dichotomy.
Specifically, we hypothesize that the observed co-expression of stemness and matrix genes reflects a rejuvenated, regenerative phenotype, rather than a classical undifferentiated stem cell profile. Importantly, we acknowledge that our original grouping lacked sufficient resolution to decouple these effects.
To address this, we have now restructured the experimental design into three well-controlled co-culture groups:
LPS-activated macrophages + AF cells (baseline inflammatory impact);
LPS-macrophages + AF cells + AF-PTK@LEV@SPP1;
LPS-macrophages + AF cells + AF-PTK@LEV@Scr (scrambled siRNA control).
As shown in Figures 5F–G (qPCR) and 5H–I (Western blot):
In Group 1, macrophage-induced inflammation suppressed ACAN/Col II and increased MMP13 and stemness genes (c-MYC, OCT4,SOX2).In Group 2, treatment with AF-PTK@LEV@SPP1 reversed inflammation-induced ECM degradation, increasing ACAN and Col II while also reprogramming stemness markers, especially restoring c-MYC ,OCT4 and SOX2, which has been implicated in both anti-senescence and regenerative reactivation.In Group 3 (scrambled control), no meaningful rescue was observed.These results suggest that AF-PTK@LEV@SPP1 exerts dual regulatory effects: on the one hand, suppressing inflammation-mediated ECM breakdown; on the other, modulating macrophage-AF crosstalk to rejuvenate AF cells without driving pathological dedifferentiation.
We have now clarified these interpretations in the Results and Discussion sections and appreciate your comment, which helped us refine both our mechanistic understanding and experimental validation.The updated results are presented in the revised Figure 5E-I, and we added a corrected part in Page29 as follow:
"To further assess how macrophage polarization states influence AF cells, we employed a Transwell-based co-culture system (Fig. 5E) involving three groups: pro-inflammatory BMDM-M1 alone, pro-inflammatory BMDM-M1 with AF-PTK@LEV@Scr, and pro-inflammatory BMDM-M1 with AF-PTK@LEV@SPP1. Co-culture with inflammatory macrophages led to a marked reduction in aggrecan (ACAN) and collagen type II (Col II) expression in AF cells, accompanied by a significant increase in matrix metalloproteinase 13 (MMP13), indicating extracellular matrix degradation. Notably, treatment with AF-PTK@LEV@SPP1 restored ACAN and Col II expression and suppressed MMP13 upregulation, suggesting that this nanoplatform protects the matrix function of AF cells by modulating macrophage phenotype. In contrast, AF-PTK@LEV@Scr failed to exert a similar effect (Fig. 5F, I).Comprehensive quantitative analyses of protein levels are provided in Supplementary Fig. 7A.
Moreover, we observed that exposure to pro-inflammatory BMDM-M1 downregulated the expression of stemness-related genes c-MYC,OCT4 , and SOX2 in AF cells. This abnormal expression profile was not significantly altered by AF-PTK@LEV@Scr treatment. However, AF-PTK@LEV@SPP1 reversed the trend, leading to a significant upregulation of these stemness markers, suggesting that the system helps maintain the stem-like properties of AF cells under inflammatory conditions (Fig. 5G–H).Comprehensive quantitative analyses of protein levels are provided in Supplementary Fig. 7B.
In summary, AF-PTK@LEV@SPP1 not only enables ROS-responsive intercellular nanoparticle trafficking, but also alleviates intervertebral disc degeneration at multiple levels by suppressing M1 macrophage polarization and improving the functional status of AF cells."

- several images in Fig 6G seem vertically stretched (liposomes without peptide; 1h, 1d, 2d)
RE:Thank you very much for your careful review and valuable feedback. Regarding your comment that several images in Figure 6G (liposomes without peptide; 1h, 1d, 2d) appear vertically stretched, we sincerely apologize.
After thorough checking, we found that the stretching occurred inadvertently during the figure assembly process when the images were moved and resized. This was a mistake on our part due to insufficient final verification, and we deeply regret the oversight.
We have now replaced the affected images with the original, unstretched versions, and the corrected images have been updated in the revised Figure 6.
We truly appreciate your attention to detail, which helped us improve the quality of our figures. Thank you again for your thoughtful and constructive comments.

- I am unfamiliar with Pfirrmann grading for IVDD in the mouse. Please refer to the study validating this classification for use in mouse models
RE:We sincerely appreciate your thoughtful comment and the opportunity to clarify this point.
First, we would like to clarify a possible misunderstanding: our MRI-based assessment was conducted on rat

intervertebral discs, not mouse models. We believe the reference to "mouse" may have been an oversight.

Regarding the use of the Pfirrmann grading system[1-6], we acknowledge that the original classification was developed for human lumbar discs. However, several studies have adapted and validated modified versions of this system specifically for use in rat models. In our study, we followed the modified grading criteria described in established literature, such as:*(1)Liang, S. et al. Magnetic resonance imaging classification in a percutaneous needle injury rat model of intervertebral disc degeneration. (2)Che, Y. J. et al. Assessment of changes in the micro-nano environment of intervertebral disc degeneration based on Pfirrmann grade.*

These references provided clear criteria for evaluating T2-weighted signal intensity and disc morphology in the rat lumbar spine. Using these adaptations, we carefully classified and scored the degree of disc degeneration in our MRI data.

To address the reviewer's concern, we have re-examined our MRI images using the aforementioned standards. Two independent researchers scored the images blindly, and discrepancies were resolved through consensus with a third evaluator. The results remained consistent with our original classification, further supporting the robustness of our grading process.

We are grateful for this comment, which prompted us to revalidate and strengthen the clarity of our methodology.we added a corrected part in Page34 as follow:

"MRI imaging further corroborated these findings, with the AF-PTK@LEV@SPP1 group displaying higher signal intensity in the nucleus pulposus region, consistent with improved hydration and tissue integrity (Figure 7D). Quantitative evaluation using the Pfirrmann grading system demonstrated significantly lower degeneration scores in the AF-PTK@LEV@SPP1-treated discs than in the PBS group, while Liposome@SPP1 and @LEV@SPP1 groups showed no significant differences from controls (Figure 7E). High-resolution micro-CT imaging additionally revealed reduced osteophyte formation and better preservation of endplate architecture in the AF-PTK@LEV@SPP1 group compared to the pronounced osseous proliferation observed in untreated and vehicle-treated discs (Figure 7F)."

References

1.Liang, S. et al. Magnetic resonance imaging classification in a percutaneous needle injury rat model of intervertebral disc degeneration. J. Orthop. Surg. Res. ***19****, 632 (2024).*

2.Che, Y. J. et al. Assessment of changes in the micro-nano environment of intervertebral disc degeneration based on Pfirrmann grade. Spine J. ***19****, 1242–1253 (2019).*

3.Li, D. et al. Lumbar intervertebral disc puncture under C-arm fluoroscopy: a new rat model of lumbar intervertebral disc degeneration. Exp. Anim. ***63****, 227–234 (2014).*

4.Zanetti, M., Hodler, J., Boos, N. Magnetic resonance classification of lumbar intervertebral disc degeneration. Spine ***26****, 1873–1878 (2001).*

5.Urrutia, J. et al. The Pfirrmann classification of lumbar intervertebral disc degeneration: an independent inter- and intra-observer agreement assessment. Eur. Spine J. ***25****, 2728–2733 (2016).*

6.Ohnishi, T., et al. In vivo mouse intervertebral disc degeneration model based on a new histological classification. PLoS ONE ***11****, e0160486 (2016).*

-also here the control lacks the scrambled version of the AF binding peptide

RE:Thank you for your valuable comment regarding the absence of a scrambled version of the AF-binding peptide as a control in Figure 6.

We apologize for the confusion. The data involving the scrambled peptide control were indeed included in our study, but are presented in the Supplementary Figure 7 (panels A, B, D, and E) for clarity and space considerations.

Specifically:Supplementary Fig. 6A shows confocal microscopy images comparing the binding of P1, scrambled P1, and no peptide (all Cy5.5-labeled) to FITC-labeled AF cells, following 30-minute incubation at 10 μM.

Supplementary Fig. 6 B presents in vivo fluorescence imaging of P1, scrambled P1, and no peptide in rat intervertebral discs across multiple time points (1h to 10d post-injection).

Supplementary Fig. 6D quantifies the fluorescence intensity in AF cells after treatment with P1, scrambled P1, and no peptide.

Supplementary Fig. 6E depicts the longitudinal tracking of peptide retention in the disc space over 240 hours, further demonstrating the specificity and retention differences between P1 and scrambled P1.

We have now clarified this in the main text and legend to ensure readers can easily locate these control data. We greatly appreciate your attention to this detail, which allowed us to improve the clarity and completeness of our presentation.

We added a Supplementary Fig. 6 and the corresponding results are presented on Page22 as follow:

"To rigorously assess the targeting specificity of peptide 1 (P1), we synthesized a scrambled version of the peptide (control sequence: YLNSYSFAGKPT) and evaluated its binding behavior in both in vitro and in vivo models. In cultured annulus fibrosus (AF) cells, we incubated Cy5.5-labeled P1, scrambled P1, and a no-peptide control at 10

μM for 30 minutes, followed by imaging via confocal microscopy (Supplementary Fig. 6A). Fluorescent signals revealed that P1 showed strong surface binding and cytoplasmic accumulation in AF cells, whereas the scrambled sequence and dye-only controls resulted in weak or negligible fluorescence, indicating minimal non-specific uptake.To quantitatively compare targeting performance, we measured the relative fluorescence intensity in each group (Supplementary Fig. 6D). P1-treated cells exhibited a significantly higher mean fluorescence intensity than both scrambled P1 and no-peptide controls ($p < 0.0001$), demonstrating the sequence-dependent specificity of P1 binding to AF cells.
Next, we performed in vivo validation using a rat intradiscal injection model. Cy5.5-labeled P1, scrambled P1, and free dye were injected into the intervertebral discs, and fluorescent retention was longitudinally monitored using small animal imaging (Supplementary Fig. 6B). While all three groups showed comparable initial signals at 1 hour, signal from scrambled P1 and free dye diminished rapidly after 24–48 hours. In contrast, P1 remained detectable in the disc space up to 10 days post-injection, with a fluorescence intensity of ~34. units at 240 hours (Supplementary Fig. 6E), confirming its superior in vivo retention and targeting stability."

-In general data on viability of peptide and nanoparticles seem to be missing
RE:Thank you for your thoughtful comment regarding the need for viability data for the peptide and nanoparticles. To address this concern, we performed a CCK-8 assay to evaluate the cytotoxicity of the P1 peptide across a range of concentrations (1 μM, 5 μM, 10 μM, 20 μM) in rat annulus fibrosus (AF) cells. As shown in Supplementary Figure X, P1 treatment had no significant effect on cell viability compared to untreated controls (0 μM), even at concentrations higher than those used in our main experiments. The results suggest that the P1 peptide exhibits good biocompatibility and minimal cytotoxicity in vitro.
We have now included this data in the revised supplementary materials and described it in the Methods and Results sections accordingly. We appreciate your suggestion, which helped us strengthen the validation of the peptide's safety profile.
The updated results are presented in Supplementary Fig. 9. , and we added a corrected part in Page37 as follow:
"To further validate the safety and translational feasibility of AF-PTK@LEV@SPP1 and the targeting peptide P1, we conducted both in vivo and in vitro biosafety assessments (Supplementary Fig. 9).Histological examination of major organs—including the lung, heart, spleen, liver, and kidney—was conducted 7 days after systemic administration of AF-PTK@LEV@SPP1, @LEV@SPP1, Liposome@SPP1, PBS, or Sham (Supplementary Fig. 9A). Hematoxylin and eosin (H&E) staining revealed no observable tissue abnormalities, inflammatory infiltrates, or signs of structural damage in any treatment group. The pulmonary alveolar architecture, myocardial fibers, hepatic lobular organization, renal tubules and glomeruli, and splenic parenchyma all appeared intact and comparable to controls. These findings indicate excellent systemic biocompatibility and absence of acute toxicity following administration of the nanotherapeutics.
In parallel, we evaluated the cytocompatibility of the AF-targeting peptide P1 in annulus fibrosus (AF) cells using a CCK-8 assay. AF cells were incubated with increasing concentrations of P1 peptide (0–20 μM) for 24 hours. As shown in Supplementary Fig. 9B, no significant reduction in cell viability was observed across all tested concentrations, with viability consistently above 90%. These data demonstrate that the P1 peptide is non-cytotoxic to AF cells even at high dosages.Together, these in vivo and in vitro results confirm that both AF-PTK@LEV@SPP1 and the AF-targeting P1 peptide exhibit favorable biosafety profiles, supporting their potential for translational application in intervertebral disc regenerative therapy."

Responses to Reviewer #2 :
In the manuscript by Li et al. they address the need to block the generation of M1 macrophages in the annulus fibrosus, since this macrophage population is the major cause of tissue damage in their IVDD mouse model. Their studies showed that the glycoprotein SPP1 (osteopontin) is a key regulator of macrophage activation and M1 polarization within the disc. To accomplish this goal of limiting the generation of M1 macrophages and to do so using an approach that can be used clinically, they developed a novel extracellular vesicle (EV). The EV consists of a liposome containing an siRNA to SPP1 fused to an E. coli OMV. They predicated this EV would better target macrophages compared to a liposome. To keep the EV localized to the annulus fibrosus (AF), they screened and identified a peptide that binds to AF cells and coupled this peptide to the OMV-liposome EV (LEV@SPP1) to generate the AF-PTK@LEV@SPP1. The peptide linkage was labile and would be cleaved from the EV upon exposure to oxidative stress (e.g. exposure to ROS). In general, I thought the experiments supported the authors claims. My comments are specific to the generation and testing of their EVs.
RE:We would like to express our deepest and most sincere gratitude to you for the thoughtful, constructive, and supportive comments on our manuscript. We truly appreciate the recognition of our efforts to design a clinically translatable approach for limiting M1 macrophage generation within the annulus fibrosus, and for acknowledging

that our experimental data generally support our claims. Your careful reading and insightful summary of our EV design, targeting strategy, and ROS-responsive release mechanism are extremely encouraging to us.
In response to your valuable feedback on the generation and testing of our EVs, we want to assure you that we have performed extensive additional experiments to further characterize and validate our system, which are now included in the revised manuscript. We have carefully revised our descriptions to better clarify these new data and have provided detailed methodological and analytical information in the supplementary materials.
Once again, we are truly grateful for your constructive input and for your support of our work. Your comments have greatly improved the quality and clarity of our manuscript. We hope that our revisions meet your expectations and address your concerns fully.

Additional characterization of the AF-PTK@LEV@SPP1 is needed.
RE:We sincerely appreciate your constructive feedback regarding the characterization of our AF-PTK@LEV@SPP1 system. In response to your suggestion, we have conducted several additional experiments to provide further validation and clarification of our results. We have revised and updated Figure 4 to provide a more accurate characterization of the material. Additionally, in response to your question, we have included Supplementary Fig 7 to offer a more comprehensive and detailed characterization. Specifically, we have evaluated our material from the following aspects.
(1)Detailed Physicochemical Characterization of AF-PTK@LEV@SPP1:
To better characterize the AF-PTK@LEV@SPP1 system, we performed extensive analysis of its physical properties. This included the use of Transmission Electron Microscopy (TEM) to assess the morphology of the nanoparticles, revealing the bilayered spherical structures characteristic of liposome-bacterial EV complexes (Figure 4B). We also utilized Dynamic Light Scattering (DLS) to confirm the particle size distribution, with AF-PTK@LEV@SPP1 showing a mean size of approximately 161 nm, indicative of good homogeneity (Figure 4C). Additionally, Zeta potential analysis demonstrated that the particles are stable in suspension with a negative charge of around -28 mV (Figure 4F), which is consistent with expected behavior for this type of nanoparticle.
(2)Confirmation of Liposome-OMV Fusion:
To ensure successful fusion of the liposomes and outer membrane vesicles (OMVs), we conducted a dual-color fluorescence labeling experiment. Prior to fusion, the liposomes and OMVs were labeled with different fluorescent dyes (DiO for OMVs, DiR for liposomes). Post-fusion, we observed merged fluorescence signals, confirming the effective integration of the two components (Figure 4D).
(3)Macrophage-Specific Uptake:
To validate the macrophage-targeting specificity of @LEV@SPP1, we incubated DiO-labeled @LEV with M1-polarized macrophages and AF cells. Our results showed that @LEV@SPP1 nanoparticles were preferentially taken up by macrophages, whereas minimal internalization was observed in AF cells, indicating that OMVs provide a macrophage-specific targeting capability (Figure 4G, Supplementary Fig. 7E–F).
(4)ROS-Responsive Release:
In response to your comment on the ROS responsiveness of the system, we quantified the release of siRNA from AF-PTK@LEV@SPP1 under oxidative stress conditions. We exposed the system to 100 μM hydrogen peroxide and observed a significant increase in siRNA release (89%) compared to the control (34%) over a 72-hour period (Figure 4H). This experiment demonstrates the ROS-triggered cleavage of the thioketal linker, leading to the release of encapsulated siRNA.
(5)Cytotoxicity Evaluation:
We also performed a cytotoxicity assessment using the CCK-8 assay to ensure the biocompatibility of AF-PTK@LEV@SPP1. The results confirmed that the nanoparticles had minimal cytotoxicity, with AF cell viability remaining above 85% under all tested conditions, including at concentrations up to 50 μM (Figure 4I).
These additional experiments were performed to address the concerns raised and to provide a more comprehensive characterization of the AF-PTK@LEV@SPP1 system. In addition, we have included Supplementary Fig. 9 to demonstrate systemic biosafety in vivo and in vitro.
We believe that these results strongly support the claims made in our manuscript and help to clarify the functionality and safety of the system in a manner that is consistent with the expectations for clinical translation. We hope these experiments provide sufficient evidence of the characterization and performance of the AF-PTK@LEV@SPP1 system. Thank you again for your helpful suggestions. The corresponding results are presented in Figure 4 and Supplementary Fig. 7 and 9, with detailed descriptions on Pages 23–27 of the revised manuscript as follow:
“To precisely modulate macrophage function within the degenerated annulus fibrosus (AF), we engineered a multi-functional delivery platform—AF-PTK@LEV@SPP1—that integrates (1) siRNA cargo against SPP1, (2) macrophage-tropic bacterial outer membrane vesicles (OMVs), (3) ROS-sensitive thioketal (TK) linkers, and (4) an annulus fibrosus–targeting peptide (AF-P) (Fig. 4A). This modular design enables tissue-specific accumulation,

inflammatory signal–responsive release, and cell-type–specific uptake.
We first constructed Liposome@SPP1 nanoparticles to encapsulate siRNA via lipid self-assembly, followed by isolation of OMVs from genetically attenuated E. coli (BL21-ΔmsbB). Through membrane extrusion and sonication, liposomes and OMVs were fused into hybrid vesicles (@LEV@SPP1), exhibiting uniform morphology with a mean size of ~114 nm. Next, AF-P was conjugated to @LEV@SPP1 using a PEG2K spacer and TK linker, resulting in the final formulation AF-PTK@LEV@SPP1.
Transmission electron microscopy (TEM) revealed that the nanoparticles exhibited clear bilayered spherical structures. Dynamic light scattering (DLS) measurements showed that the average particle size was approximately 114.6 nm for OMVs, 115.6 nm for Liposome@SPP1, and increased to around 161.6 nm for AF-PTK@LEV@SPP1, with all formulations displaying a polydispersity index (PDI) below 0.2, indicating good dispersion uniformity (Fig. 4B–C). Zeta potential analysis demonstrated that all nanoparticles were negatively charged, with values ranging from -28 mV to -32 mV, consistent with good stability in suspension (Fig. 4F).
To validate the successful fusion of liposome and OMV membranes, dual-color fluorescence labeling (DiO for OMVs, DIR for liposomes) was employed. Prior to co-extrusion, the signals were spatially distinct, but post-fusion vesicles exhibited merged green and red fluorescence (Fig. 4D), confirming membrane integration.
SDS-PAGE further demonstrated that @LEV incorporated OMV-specific membrane proteins absent in pure liposomes, indicating the preservation of immunogenic surface motifs important for macrophage recognition (Fig. 4E).
To assess cellular uptake specificity across different nanoparticle formulations, we incubated DiO-labeled @LEV, Liposome@SPP1, and OMVs with M1-polarized macrophages . As shown in Figure 4G, @LEV exhibited preferential uptake by macrophages, with markedly less internalization observed in Liposome@SPP1 , indicating macrophage-specific targeting conferred by the OMV component. In contrast, liposomes alone showed poor uptake in M1-polarized macrophages, highlighting the necessity of membrane fusion with OMVs to enhance cellular entry. Notably, OMVs demonstrated the strongest phagocytosis among the three groups, suggesting that bacterial membrane-derived vesicles possess innate macrophage-targeting capacity, which is partially retained in the @LEV formulation.Quantification of DiO fluorescence intensity in M1 macrophages after incubation with DiO-labeled @LEV, liposome, or OMVs formulations is in Supplementary Fig. 7G. To assess cellular uptake specificity across different cells, we incubated DiO-labeled @LEV@SPP1 with M1 macrophages and AF cells. As shown in Supplementary Fig. 7E, @LEV@SPP1 displayed preferential accumulation in macrophages. Quantification of fluorescence intensity (Supplementary Fig. 7F) confirmed significantly higher uptake in macrophages than in AF cells.
We next evaluated the ROS responsiveness of AF-PTK@LEV@SPP1. Upon exposure to 100 μM hydrogen peroxide, the cumulative release of siRNA increased significantly, reaching ~89% at 72 hours, whereas only ~34% was released under basal conditions (Fig. 4H). This dynamic responsiveness was enabled by the thioketal linker, allowing controlled release in oxidative environments.
Agarose gel electrophoresis (Supplementary Fig. 7A) further demonstrated that AF-PTK@LEV@SPP1 protected siRNA from enzymatic degradation for up to 24 hours, significantly outperforming free siRNA. UV–visible spectroscopy confirmed siRNA encapsulation efficiency (Supplementary Fig. 7B) and absorbance shifts associated with nanocomposite formation (Supplementary Fig. 7C). SEM-EDS analysis visualized the overall morphology and verified the elemental composition of the final nanoparticles (Supplementary Fig. 7D).
To ensure biocompatibility, AF cells were incubated with increasing concentrations (0–50 μM) of OMVs, Liposome@SPP1, @LEV@SPP1, and AF-PTK@LEV@SPP1. The CCK-8 assay revealed minimal cytotoxicity across all treatment groups, with cell viability exceeding 85% under all conditions (Fig. 4I), supporting favorable biosafety for biological applications.
Collectively, these results establish that AF-PTK@LEV@SPP1 is a rationally designed, AF-targeted and ROS-activated nanoparticle capable of delivering siRNA specifically to macrophages, with high structural stability, efficient encapsulation, and excellent biosafety (Figure 4, Supplementary Fig. 7)”

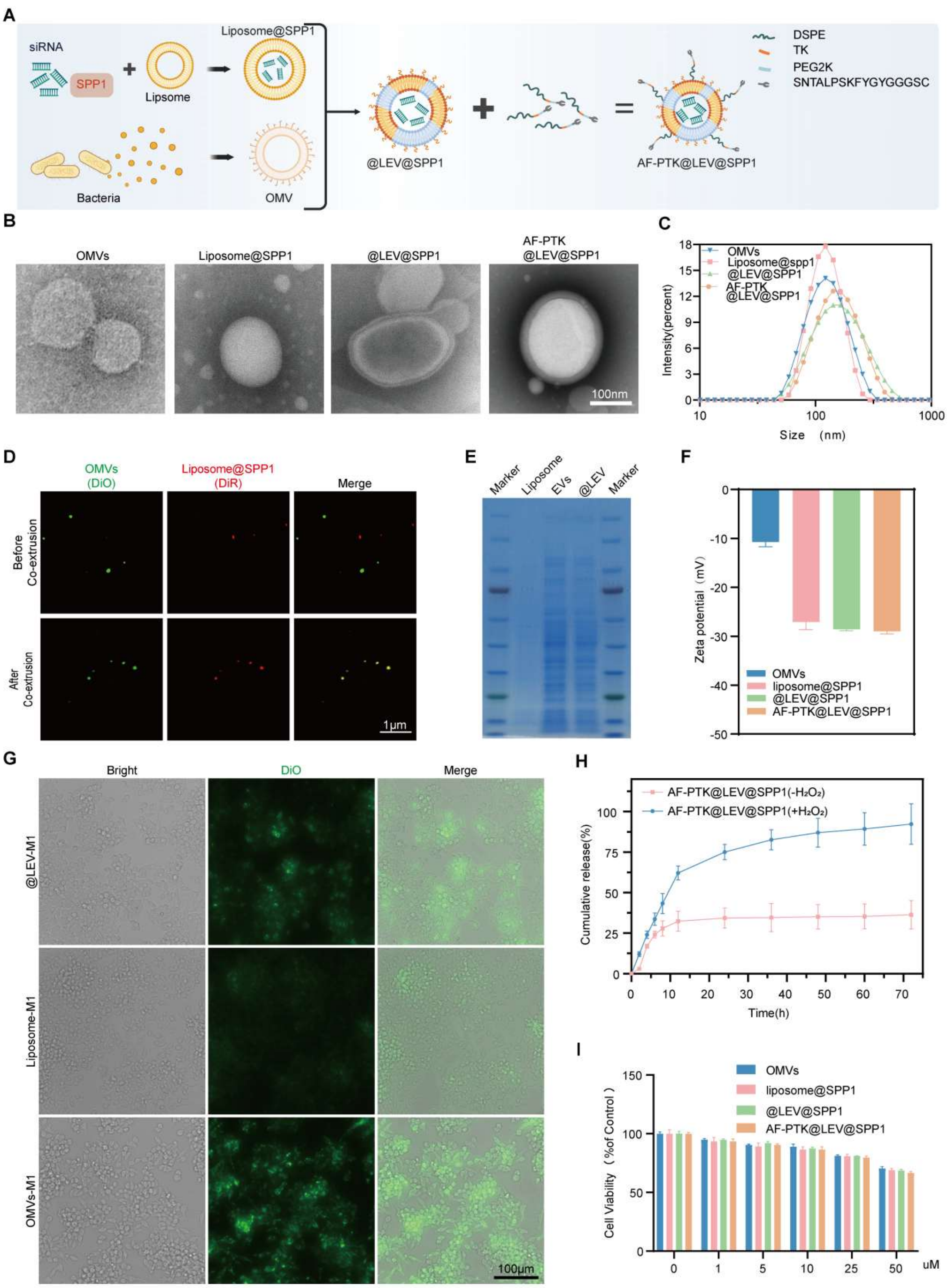

Figure 4. Characterization of AF-PTK@LEV@SPP1 Membrane Fusion Bacterial Extracellular Vesicles.

(A) Schematic illustration of the synthesis of AF-PTK@LEV@SPP1:Schematic representation of the construction process of AF-PTK@LEV@SPP1 nanoparticles, integrating liposome-encapsulated siRNA, outer membrane vesicles (OMVs), and annulus fibrosus (AF)-binding peptide via ROS-responsive thioketal linkers.

(B) Transmission electron microscopy (TEM) images of OMVs, Liposome@SPP1, @LEV@SPP1, and AF-PTK@LEV@SPP1.Scale bar = 100 nm.

(C)Dynamic light scattering (DLS) analysis of particle size distribution profiles for the individual components and the AF-PTK@LEV@SPP1.

(D)Fluorescence images depicting the state before and after co-extrusion of the mixed solution containing OMVs (labeled with green DiO) and Liposome@SPP1 (labeled with red DiR) . Scale bar = 1 μm.

(E)SDS-PAGE analysis of membrane protein profiles of liposomes, OMVs, and @LEV.

(F)Zeta potential measurements of OMVs, Liposome@SPP1, @LEV@SPP1, and AF-PTK@LEV@SPP1.

(G) Fluorescence microscopy images of M1 macrophages and AF cells incubated with DiO-labeled LEV and liposome formulations to assess cellular uptake specificity.Scale bar = 100 μm.

(H)In vitro cumulative siRNA release from AF-PTK@LEV@SPP1 in the presence and absence of hydrogen peroxide (H_2O_2) at 100 μM, monitored over time.

(I)CCK-8 assay assessing the cytocompatibility of OMVs, Liposome@SPP1, @LEV@SPP1, and AF-PTK@LEV@SPP1 at various concentrations.

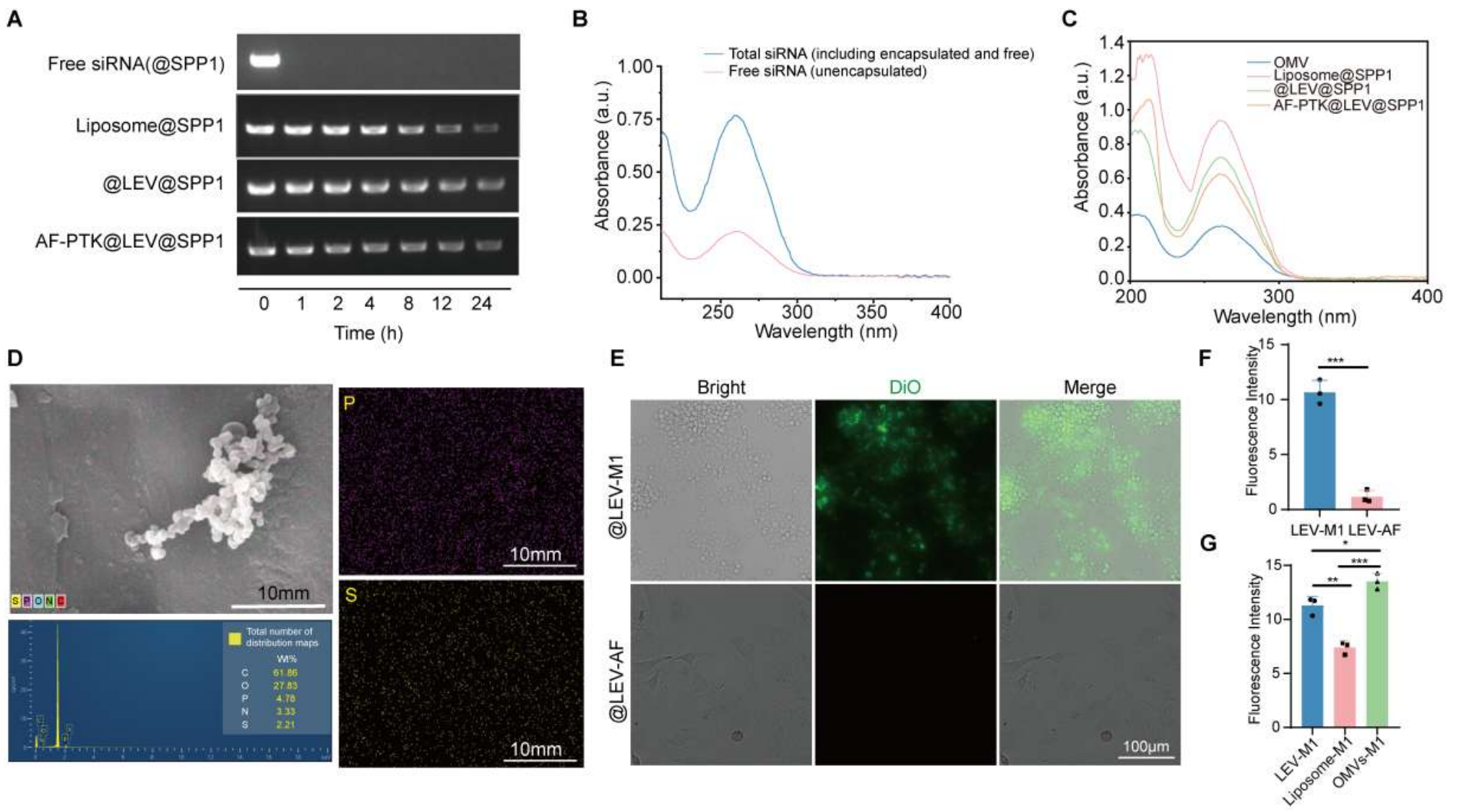

Supplementary Fig. 7.Physicochemical characterization and cellular uptake of AF-PTK@LEV@SPP1.

(A)Agarose gel electrophoresis assessing siRNA stability in different formulations over time under enzymatic degradation conditions. Four groups were tested: free siRNA, Liposome@SPP1, @LEV@SPP1, and AF-PTK@LEV@SPP1.

(B) UV–visible spectroscopy showing absorbance profiles of total siRNA (encapsulated and unencapsulated) and free siRNA alone.

(C) UV–visible absorption spectra of OMVs, Liposome@SPP1, @LEV@SPP1, and AF-PTK@LEV@SPP1.

(D) SEM image, energy-dispersive X-ray spectroscopy (EDS) spectrum, and elemental distribution maps showing the composition of AF-PTK@LEV@SPP1, with elemental mapping for phosphorus (P) and sulfur (S). Scale bar for elemental maps, 10 mm.

(E)Fluorescence microscopy images of M1 macrophages and AF cells incubated with DiO-labeled LEV at 10 μM for 30 minutes.Scale bar = 100 μm.

(F) Quantification of relative fluorescence intensity in M1 macrophages and AF cells following nanoparticle incubation.

(G) Quantification of DiO fluorescence intensity in M1 macrophages after incubation with DiO-labeled @LEV, liposome, or OMVs formulations in Figure 4(G).

*$p < 0.05$, **$p < 0.01$, ***$p < 0.001$.

Co-localization of DiR Fluorescent dye labeled OMV and liposomes is not sufficient to show membrane fusion as exchange of membrane but not fusion of membranes is possible. This is especially a concern since both the OMV and liposomes are ~150nm but the fusion produce is also ~150nm. That is, there is no increase in size from the vesicle fusion, perhaps because they are selecting vesicles of <200nm in their filtration steps. Looking for specific markers of OMV and liposome on the fused EVs should be performed to confirm that they have generated fused vesicles. It is also important that they show the reproducibility of the fused EVs. That is, how much variation do they see in the composition of the AF-PTK@LEV@SPP1 with different preps.

RE:We sincerely thank you for this important and insightful comment regarding the validation of true membrane fusion and the reproducibility of the fused vesicle preparation.

We fully agree that fluorescent dye co-localization alone is insufficient to confirm membrane fusion, as it may result from lipid exchange or vesicle aggregation rather than genuine bilayer integration. In response to this concern, we conducted a series of complementary validation experiments:

Biochemical Validation via SDS-PAGE:As shown in the revised Figure 4E, we performed SDS-PAGE followed by Coomassie Brilliant Blue staining to analyze the protein profiles of OMVs alone, liposomes alone, and fused vesicles (@LEV). The @LEV samples exhibited distinct protein bands that overlapped with both OMVs and

liposomes, confirming that structural components from each were incorporated into the hybrid membrane structure. These fusion profiles were reproducibly observed across three independent batches, supporting the robustness and batch-to-batch consistency of our preparation method.

Reassessment of Particle Size Measurements:We appreciate your point regarding the similar particle sizes (~150 nm) of liposomes, OMVs, and the fusion products, as initially reported. This prompted us to repeat the particle size measurements using freshly prepared and ultracentrifugation-purified samples, while carefully removing aggregation-related artifacts and eliminating the impact of extrusion-induced bias.Upon re-analysis, we observed that:Pre-fusion vesicles (OMVs and liposomes) had an average hydrodynamic diameter of ~110 nm and Post-fusion @LEV vesicles increased to ~160 nm.

These results are now presented in the updated Figure 4C. The observed increase in particle size provides additional evidence supporting membrane fusion rather than simple mixing.

Methodology Clarification and Text Revisions:We have clarified in the Methods and Results sections that:All vesicles, including those used for imaging, were ultracentrifugation-purified prior to characterization, ensuring removal of free dyes and unfused vesicles.The fluorescent co-localization observed in Figure 4D corresponds to vesicles that underwent membrane extrusion followed by ultracentrifugation.

We added the following updated description in the revised manuscript (Page25):

"To validate the successful fusion of liposome and OMV membranes, dual-color fluorescence labeling (DiO for OMVs, DIR for liposomes) was employed. Prior to co-extrusion, the signals were spatially distinct, but post-fusion vesicles exhibited merged green and red fluorescence (Fig. 4D), confirming membrane integration.

SDS-PAGE further demonstrated that @LEV incorporated OMV-specific membrane proteins absent in pure liposomes, indicating the preservation of immunogenic surface motifs important for macrophage recognition (Fig. 4E)."

Once again, we are very grateful to you for this detailed and constructive comment, which led us to re-express critical experimental procedures and add essential data to substantiate our conclusions.

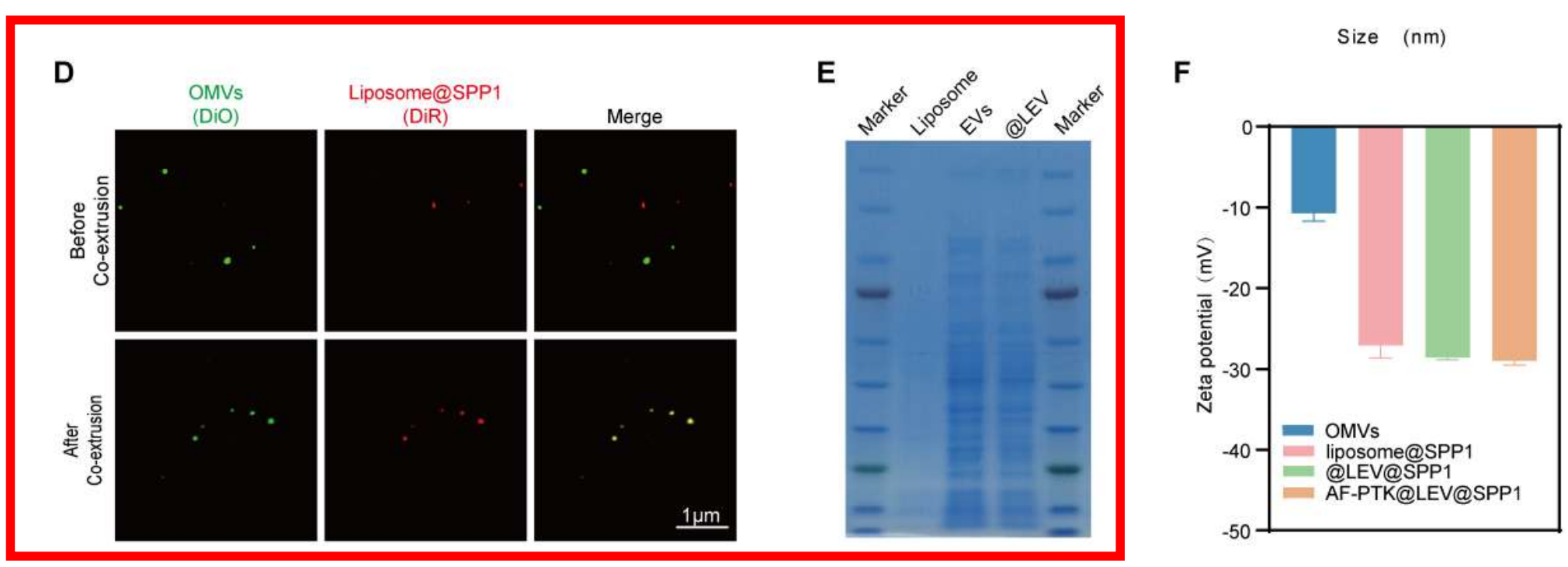

Figure 4. Characterization of AF-PTK@LEV@SPP1 Membrane Fusion Bacterial Extracellular Vesicles.

(D)Fluorescence images depicting the state before and after co-extrusion of the mixed solution containing OMVs (labeled with green DiO) and Liposome@SPP1 (labeled with red DiR) . Scale bar = 1 μm.

(E)SDS-PAGE analysis of membrane protein profiles of liposomes, OMVs, and @LEV.

Reference：

1.Sun, M. et al. Beyond extracellular vesicles: hybrid membrane nanovesicles as emerging advanced tools for biomedical applications. Adv. Sci. ***10****, e2303617 (2023).*

2.Li, Z. et al. Dynamic fusion of nucleic acid functionalized nano-/micro-cell-like containments: from basic concepts to applications. ACS Nano ***17****, 15308–15327 (2023).*

3.Yamada, Y., Sato, Y., Nakamura, T. & Harashima, H. Innovative cancer nanomedicine based on immunology, gene editing, intracellular trafficking control. J. Control. Release ***348****, 357–369 (2022).*

They describe their purification process as follow:

"After sonication, the suspension was centrifuged at 10,000 g for 10 min to remove aggregates. The supernatant containing fusogenic liposomes was collected and further purified by ultracentrifugation at 100,000 g for 1 h at 4°C. The final pellet was resuspended in sterile PBS and stored at 4°C for subsequent characterization."

"EVs were labeled with DiR fluorescent dye (red) by mixing the EV solution with DiR and incubating at 37 °C for 15 minutes. The solution was then purified by centrifugation (12,000 rpm, 10 minutes) to obtain DiR-labeled EVs. Similarly,siRNA-loaded liposomes were labeled with DiO fluorescent dye (green) by incubating the liposome solution with DiO at 37 °C for 15 minutes, followed by centrifugation (12,000 rpm, 10 minutes) to purify the DiO-labeled liposomes.The DiR-labeled EVs and DiO-labeled liposomes were mixed and repeatedly extruded

through 400 nm and 200 nm polycarbonate membranes using a liposome extruder. After extrusion, the fusion of EVs and liposomes was verified using confocal microscopy."

From these descriptions it appears they used different processes to purify the fused OMVs and liposomes (AF-PTK@LEV@SPP1). For the EM and fluorescent microscopy studies (Figure 4) they used filtration while for the in vivo experiments they used centrifugation at 100,000 xg. Therefore, it is unclear that any characterization was done on the vesicles that were purified by centrifugation.

RE:We sincerely thank you for this careful and insightful comment. We fully agree that the purification process is critical to both data interpretation and experimental reproducibility, and we apologize for the lack of clarity in our initial description.

To clarify, all vesicles used in this study—including those for fluorescent microscopy, electron microscopy, DLS, and in vivo administration—were ultimately purified via ultracentrifugation at 100,000 ×g for 1 hour at 4° C. This purification step was consistently applied after any upstream treatments such as extrusion or fluorescent dye labeling.

In the case of the fluorescence co-localization study (Figure 4), we acknowledge that our original Methods section only mentioned the preliminary centrifugation step (12,000 rpm, 10 min) following DiO and DiR dye labeling. This might have led to the impression that the labeled vesicles used for confocal imaging were not further purified. In fact, after dye removal by initial low-speed centrifugation, the labeled vesicles were fused via membrane extrusion, and the resulting hybrid vesicles were then subjected to ultracentrifugation at 100,000 ×g to eliminate residual free dye, dye-labeled but unfused vesicles, and aggregates.

We have now revised the Materials and Methods section to clearly indicate this two-step purification process and added the following sentence:

"Only ultracentrifugation-purified vesicles were used for fluorescence imaging to eliminate free dye artifacts and confirm true membrane colocalization."

Furthermore, we confirm that all structural and functional characterization, including the confocal colocalization results shown in Figure 4, were performed using these ultracentrifugation-purified hybrid vesicles. This correction has now been incorporated into the manuscript, and we are grateful to you for identifying this omission, which helped us improve the clarity and rigor of our methodological reporting.

Other comments:

What is the uptake of OMVs without fusion with liposomes?

RE:Thank you very much for your thoughtful question regarding the uptake efficiency of OMVs alone, without fusion with liposomes.

To address this point, we evaluated the cellular uptake of DiO-labeled OMVs, liposomes, and fused hybrid vesicles (@LEV) in M1-polarized macrophages. As shown in Figure D–E, OMVs without fusion exhibited the highest uptake efficiency among the three formulations, as indicated by the strongest fluorescence intensity signal in the DiO channel.

Quantitative analysis (Figure E) confirmed that OMVs-M1 showed significantly greater fluorescence intensity compared to LEV-M1 ($p < 0.05$) and liposome-M1 ($p < 0.001$), suggesting that native OMVs possess strong macrophage-targeting and internalization capabilities, likely due to the presence of outer membrane proteins and pathogen-associated molecular patterns (PAMPs).

These results provide important mechanistic support for our fusion strategy: while OMVs have robust uptake, the LEV platform combines their biological uptake advantages with controlled siRNA delivery and immune safety.

We appreciate your insightful comment, which helped us further emphasize the comparative performance of individual vesicle components.

We added a corrected part in Page25 as follow:

"To assess cellular uptake specificity across different nanoparticle formulations, we incubated DiO-labeled @LEV, Liposome@SPP1, and OMVs with M1-polarized macrophages . As shown in Figure 4G, @LEV exhibited preferential uptake by macrophages, with markedly less internalization observed in Liposome@SPP1 , indicating macrophage-specific targeting conferred by the OMV component. In contrast, liposomes alone showed poor uptake in M1-polarized macrophages, highlighting the necessity of membrane fusion with OMVs to enhance cellular entry. Notably, OMVs demonstrated the strongest phagocytosis among the three groups, suggesting that bacterial membrane-derived vesicles possess innate macrophage-targeting capacity, which is partially retained in the @LEV formulation.Quantification of DiO fluorescence intensity in M1 macrophages after incubation with DiO-labeled @LEV, liposome, or OMVs formulations is in Supplementary Fig. 7G. To assess cellular uptake specificity across different cells, we incubated DiO-labeled @LEV@SPP1 with M1 macrophages and AF cells. As shown in Supplementary Fig. 7E, @LEV@SPP1 displayed preferential accumulation in macrophages. Quantification of fluorescence intensity (Supplementary Fig. 7F) confirmed significantly higher uptake in macrophages than in AF cells.

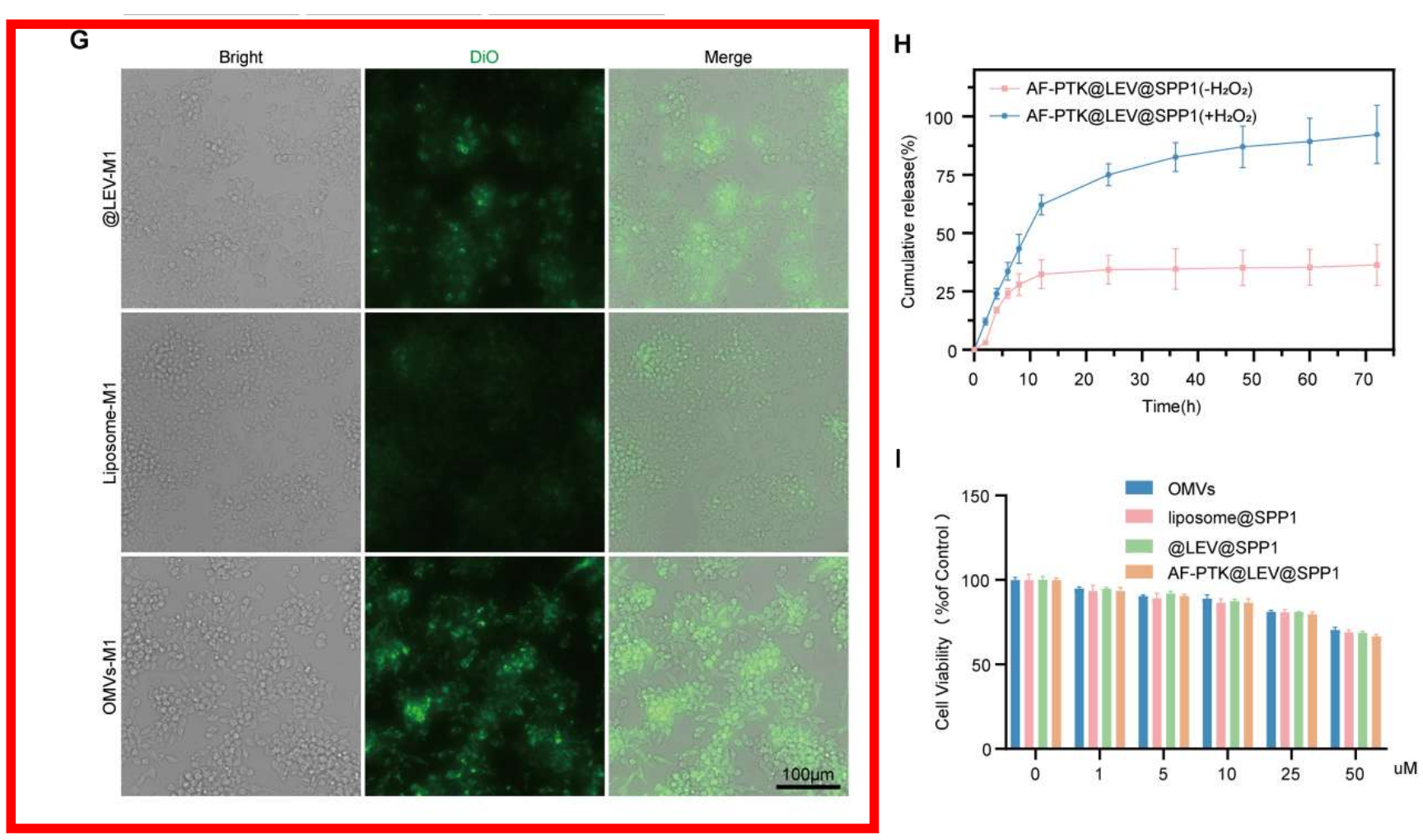

Figure 4. Characterization of AF-PTK@LEV@SPP1 Membrane Fusion Bacterial Extracellular Vesicles.

(G) Fluorescence microscopy images of M1 macrophages and AF cells incubated with DiO-labeled LEV and liposome formulations to assess cellular uptake specificity.Scale bar = 100 μm.

Figure 4E. The colocalization of @LEV with macrophages was difficult to see in this figure. Higher magnification and quantification would be useful to show.

RE:We appreciate your constructive suggestion. To make the colocalization of LEV with macrophages easier to appreciate, we undertook the following revisions:

We repeated the experiment at higher magnification.Provided the new images as Figure 4G.

The enlarged high-resolution micrographs now allow the merged signal to be readily distinguished, clearly demonstrating LEV/macrophage colocalization.

Improved image clarity：All new panels were processed with identical contrast settings and include a scale bar of 100 μm, as requested.

We believe these additions fully address your concern by providing both higher-magnification visualization and rigorous quantification of LEV uptake by macrophages, thereby strengthening the conclusions of the study.

In addition to the improved imaging, we also performed rigorous quantitative analyses to confirm the preferential uptake of LEV by macrophages. We have added new quantification panels (Figure 4G) showing statistically significant differences in fluorescence intensity in macrophages, as well as across different nanoparticle formulations. These analyses demonstrate that LEV exhibits clear macrophage-targeting specificity while showing minimal uptake in AF cells. We believe this quantitative evidence robustly supports our claim of cell-type–specific delivery. Furthermore, all fluorescence images were carefully processed using standardized contrast and exposure settings to ensure reproducibility and clarity(Supplementary Fig. 7 and G). We hope that these additions and analyses fully address the reviewer's concern by providing both visual and quantitative confirmation of macrophage colocalization and targeting specificity.

.

Figure 6C-I they are missing the important control of LPS + Liposomes@SPP1 to see if they are equally efficient at knocking down LPS driven macrophage activation (M1 phenotype) as the AF-PTK@LEV@SSP1. The prediction would be that they are not as efficient but this needs to be tested experimentally.

RE:We sincerely thank you for these insightful and important comments regarding the completeness and rigor of our experimental design.

You are absolutely right in pointing out the absence of a critical control group—LPS+Liposome@SPP1—which is essential to evaluate whether the observed suppression of macrophage M1 polarization is due to SPP1 silencing alone or enhanced by AF-targeted delivery.In response, we have carefully redesigned and expanded our experimental grouping. As shown in the revised Figure 5C–D, we have now added two additional control groups:LPS+AF-PTK@LEV@Scr: A scrambled siRNA-loaded, AF-targeted nanoparticle to confirm that the observed effects are specifically due to SPP1 silencing.

LPS+Liposome@SPP1: A non-targeted liposome delivering SPP1 siRNA (without AF peptide conjugation) to test whether untargeted delivery is equally effective in suppressing LPS-induced M1 polarization.

These groups are now incorporated into the updated flow cytometry analysis (Figure 5C) and quantification of iNOS+CD86+ macrophages (Figure 5D).

Our results demonstrate that:The scrambled siRNA group (LPS+AF-PTK@LEV@Scr) failed to suppress iNOS+

expression in macrophages, confirming the sequence specificity of SPP1-mediated effects.The Liposome@SPP1 group exhibited partial reduction in iNOS+ cells compared to LPS alone, but its effect was significantly weaker than that of AF-PTK@LEV@SPP1.These findings support the conclusion that AF-targeted delivery greatly enhances therapeutic efficacy, likely due to improved localization, retention, and uptake at the site of inflammation.

We have also updated the figure legend, Results section, and Discussion accordingly to reflect these new experimental insights.Once again, we are truly grateful for your careful and constructive critique. Your recommendation helped us strengthen the scientific validity and mechanistic interpretation of our study.

The modifications are shown in Page28 as follows:

"To assess the regulatory effects on macrophage polarization, bone-marrow-derived macrophages (BMDMs) were divided into six groups: Control (no LPS stimulation), LPS (100 ng/mL), LPS + OMV, LPS + AF-PTK@LEV@Scr (Scr denotes a scrambled SPP1-siRNA; sense 5′-GAUGACGUACCUCGGAUUA-dTdT, antisense 5′-UAAUCCGAGGUACGUCAUC-dTdT-3′), LPS + Liposome@SPP1, and LPS + AF-PTK@LEV@SPP1. Flow cytometric analysis showed that LPS stimulation markedly elevated the expression of iNOS in BMDM-M1 , a typical M1 macrophage marker, with over 90% of cells being iNOS-positive. Treatment with AF-PTK@LEV@SPP1 significantly reduced this proportion to approximately 48%, which was substantially lower than in the AF-PTK@LEV@Scr (77.8%) and Liposome@SPP1 (60.6%) control groups (Fig. 5C–D). These results indicate that AF-PTK@LEV@SPP1 effectively inhibits the pro-inflammatory M1 polarization of macrophages.

To further assess how macrophage polarization states influence AF cells, we employed a Transwell-based co-culture system (Fig. 5E) involving three groups: pro-inflammatory BMDM-M1 alone, pro-inflammatory BMDM-M1 with AF-PTK@LEV@Scr, and pro-inflammatory BMDM-M1 with AF-PTK@LEV@SPP1. Co-culture with inflammatory macrophages led to a marked reduction in aggrecan (ACAN) and collagen type II (Col II) expression in AF cells, accompanied by a significant increase in matrix metalloproteinase 13 (MMP13), indicating extracellular matrix degradation. Notably, treatment with AF-PTK@LEV@SPP1 restored ACAN and Col II expression and suppressed MMP13 upregulation, suggesting that this nanoplatform protects the matrix function of AF cells by modulating macrophage phenotype. In contrast, AF-PTK@LEV@Scr failed to exert a similar effect (Fig. 5F, I).Comprehensive quantitative analyses of protein levels are provided in Supplementary Fig. 7A.

Moreover, we observed that exposure to pro-inflammatory BMDM-M1 downregulated the expression of stemness-related genes c-MYC,OCT4 , and SOX2 in AF cells. This abnormal expression profile was not significantly altered by AF-PTK@LEV@Scr treatment. However, AF-PTK@LEV@SPP1 reversed the trend, leading to a significant upregulation of these stemness markers, suggesting that the system helps maintain the stem-like properties of AF cells under inflammatory conditions (Fig. 5G–H).Comprehensive quantitative analyses of protein levels are provided in Supplementary Fig. 7B.

In summary, AF-PTK@LEV@SPP1 not only enables ROS-responsive intercellular nanoparticle trafficking, but also alleviates intervertebral disc degeneration at multiple levels by suppressing M1 macrophage polarization and improving the functional status of AF cells."

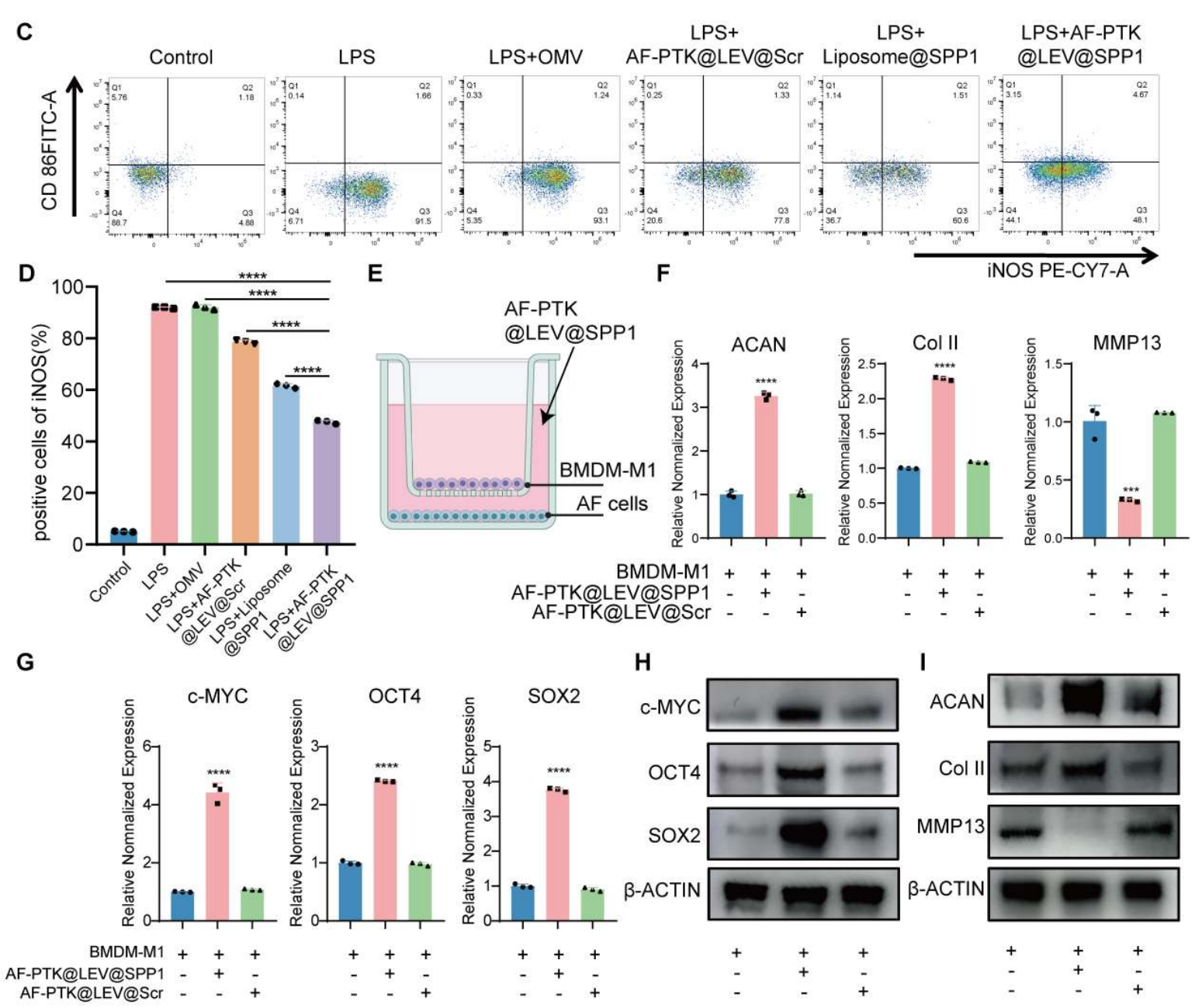

Figure 5. Dynamic phagocytosis of AF-PTK@LEV@SPP1 and its inhibition of M1 macrophage polarization to improve intervertebral disc degeneration.

(D) Quantification of iNOS-positive macrophages based on flow cytometry from (C).

(E) Schematic diagram of the Transwell co-culture system with BMDM-M1 and AF cells exposed to different treatments.

(F, G)Quantitative real-time PCR analysis of AF cell gene expression, including aggrecan (ACAN), collagen type II (Col II), matrix metalloproteinase 13 (MMP13), and stemness-associated genes (OCT4,SOX2, c-MYC), following indirect exposure to treated BMDMs.

(H, I) Western blot analysis of the indicated proteins in AF cells co-cultured with BMDMs pretreated as shown.

Data are presented as mean ± SD from at least three independent experiments. Statistical significance was assessed using one-way ANOVA with appropriate post hoc tests. * indicates $p < 0.05$, ** indicates $p < 0.01$, *** indicates $p < 0.001$, **** indicates $p < 0.0001$.

Minor comment:

Exosomes is used to specifically designate EVs released upon fusion of MVBs with plasma membrane and release of the intraluminal vesicles. This is not what EVs were used in this study. The generic term EVs should be used.

RE:Thank you for your careful observation and helpful clarification regarding the terminology. You are absolutely right—our study does not specifically involve exosomes derived from multivesicular body fusion. In response to your comment, we have carefully revised the manuscript to replace all inappropriate uses of the term exosomes with the more accurate and generic term extracellular vesicles (EVs), in accordance with current ISEV guidelines. We truly appreciate your attention to this important detail.

Responses to Reviewer #3 :

Li et al. reported the development of a compound targeting both macrophages and annulus fibrosus (AF) for the treatment of disc degeneration. Using single-cell RNA sequencing, they demonstrated that proinflammatory macrophages infiltrate human degenerated discs and identified SPP1 as a key regulator of macrophage activation in disc degeneration. To further investigate the role of SPP1 in macrophage activity and disc regeneration, they developed an AF-PTK@LEV@SPP1 targeted delivery system. This system incorporates an AF-binding peptide, liposomes, bacterial exosomes, and a thioketal linker responsive to reactive oxygen species (ROS) for controlled release.

The researchers evaluated this system in both in vitro and in vivo models to assess binding specificity, release profile, and retention. Furthermore, they tested the therapeutic potential of SPP1 modulation in a rat disc degeneration model. This study offers valuable insights into the potential of macrophage-targeted therapies for treating disc degeneration. However, it is difficult to evaluate without detailed information in the methods and results.

RE:We sincerely thank you for this insightful and encouraging comment. We are truly grateful that you recognized the potential value and scientific significance of our study, particularly in the context of macrophage-targeted strategies for intervertebral disc degeneration.

We also deeply appreciate your constructive feedback regarding the need for more comprehensive and detailed information in the Methods and Results sections. We fully agree that methodological transparency and sufficient data presentation are essential for the accurate interpretation and reproducibility of scientific findings.

In response, we have undertaken a thorough revision of the manuscript and have significantly expanded the Materials and Methods section to include detailed descriptions of all key experimental procedures. These revisions encompass:

The design and characterization of the AF-PTK@LEV@SPP1 nanoplatform;The process of phage-based peptide screening for AF-binding specificity;The preparation and validation of ROS-responsive liposome-exosome hybrids;The animal models used, including specific age, sex, strain, and ethical compliance information;The protocols for compound administration (intradiscal injection), imaging, histology, and molecular analysis.

In parallel, we have also added several new figures and expanded the Results section to present quantitative data on nanoparticle characterization, cell-specific targeting, release kinetics, in vivo retention, and therapeutic efficacy. We hope that these additions will greatly enhance the clarity, completeness, and scientific rigor of our study, making it more accessible for reviewers and readers to evaluate. Once again, we sincerely thank you for this thoughtful and valuable suggestion, which has significantly improved the quality of our manuscript.

Some specific comments:

Introduction:

The introduction lacks clarity in distinguishing between previous research and the current study's objectives.

It should be reorganized to provide a clearer background and rationale.
Reference errors: References 23–25 do not match the cited content. Additionally, references 36–40 may be incorrectly placed.

RE:We sincerely thank you for this critical and insightful observation.

Your comments have been invaluable in helping us improve the scientific rigor and clarity of our manuscript. We recognize that several of the references originally cited in the Introduction did not optimally align with the statements they were intended to support. While our goal was to provide comprehensive background context, we now realize that some of the citations may have led to ambiguity or difficulty in verifying the rationale leading to our research question.

To address this concern, we have now comprehensively revised the Introduction section of the manuscript. We restructured the background narrative to ensure a clear and logical progression of ideas, and we have replaced all previous references with carefully selected, thematically accurate, and up-to-date primary literature.

1. Burden of IVDD and Chronic Low Back Pain (Refs 1–5)

The first paragraph now cites major epidemiological studies and systematic reviews that robustly quantify the global burden, prevalence, and socioeconomic impact of chronic low back pain and IVDD. Specifically:GBD 2017 and GBD 2021 analyses in The Lancet (Refs 1, 2),Foundational epidemiological reviews by Andersson and by Hartvigsen et al. (Refs 3, 4),Global burden estimates from Hoy et al. (Ref 5).These citations directly support the opening statement about IVDD's global impact and public health importance.

2. Limitations of Current Interventions and Promise of Biomaterials (Refs 6–10)

We replaced prior irrelevant preclinical delivery-system citations with authoritative reviews and translational studies from Nature Reviews Rheumatology, Advanced Drug Delivery Reviews, Bioactive Materials, etc. For example:Reviews on cell-based and progenitor-cell therapies (Refs 6, 7),Recent analyses of annulus fibrosus biomechanics and calcification barriers (Refs 8, 9),A new immunometabolic perspective on IVDD pathophysiology (Ref 10).These references directly discuss clinical and translational obstacles, setting up the rationale for novel delivery approaches.

3. Biological Structure and Barriers of the Disc (Refs 11–16)

For statements about anatomy, matrix barriers, nutrient diffusion limits, and poor intrinsic regenerative capacity, we now cite detailed anatomical/mechanistic studies and tissue engineering reviews:Disc mechanics and degeneration pathophysiology (Ref 11),Molecular basis of matrix degradation and inflammation (Refs 12, 13),Structural studies and regenerative scaffold approaches specific to annulus fibrosus (Refs 14–16).These accurately support discussion of physical and biological barriers to therapy.

4. Immune Cell Role, Macrophage Heterogeneity, and Single-Cell Data (Refs 19–29)

For the section on macrophage infiltration, polarization states, and single-cell transcriptomics, we now include:Single-cell RNA-seq analyses specifically characterizing macrophage heterogeneity in IVDD (Ref 26),Reviews on macrophage plasticity and inflammatory arthritis mechanisms (Refs 27, 28),Experimental studies confirming M1/M2 polarization and secreted cytokine profiles in degenerating discs (Refs 29–34).These sources directly support claims about immune dysregulation, inflammatory cascades, and macrophage-driven matrix degradation.

5. Challenges in Targeted Delivery and Rationale for OMV-Based Platforms (Refs 35–47)Finally, for the sections on delivery barriers in the annulus fibrosus and the design of OMV-based delivery systems, we included:Reviews on anatomical complexity and permeability limits (Refs 35–40),Studies on siRNA delivery challenges (Ref 43),uthoritative reviews on OMV biology and engineering for drug delivery, including their immunogenicity risks and detoxification strategies (Refs 44–47).These citations ensure that our rationale for selecting an OMV-based, responsive nanotherapy platform is thoroughly and specifically supported.We have carefully double-checked that each citation is directly relevant and supports the associated text. Furthermore, all reference numbers have been updated accordingly in both the main text and the reference list.

Once again, we offer our sincere apologies for the confusion and inconvenience caused by our earlier referencing errors. We are truly grateful to the reviewer for pointing this out and for helping us enhance the scientific accuracy and integrity of our manuscript.

The Introduction and reference list have been revised and reorganized as in revised manuscript.

Materials & Methods:

More experimental details are needed, including animal age, gender, and specific experimental conditions.

RE：We sincerely thank you for this insightful comment emphasizing the importance of methodological transparency and animal welfare compliance. We fully agree that the inclusion of detailed animal-related parameters—including species, strain, age, sex, housing environment, and randomization criteria—is essential to ensure reproducibility and ethical clarity. In response, we have thoroughly revised the Materials and Methods section to expand upon and clarify the relevant experimental details, which are summarized below:

1.Animal Characteristics (Species, Strain, Age, Gender):
For the intervertebral disc degeneration (IVDD) injury model, we utilized 12-week-old male Sprague–Dawley (SD) rats, obtained from a certified laboratory animal supplier (please refer to the revised text for vendor details).
For transgenic studies involving macrophage ablation, we used 8- to 10-week-old male Lyz2-Cre;DTR mice, generated by crossing Lyz2-Cre transgenic mice with DTR-floxed mice, as described in our revised Lyz2-Cre;DTR Mice section.
All animals were confirmed to be healthy and weight-matched prior to assignment into experimental groups.
2.Housing Conditions:
Animals were maintained in a specific pathogen-free (SPF) facility under tightly regulated environmental parameters. Specifically, the housing rooms were maintained at a constant ambient temperature of 23–25°C, relative humidity of 50–60%, and a 12-hour light/dark cycle.
Rats and mice were housed in individually ventilated cages with HEPA-filtered airflow, provided with sterile bedding, and given ad libitum access to autoclaved rodent chow and water throughout the experiment.
Cage density and enrichment conditions adhered strictly to the institutional guidelines, and no overcrowding or resource competition was permitted.
3.Ethics and Animal Welfare:
All procedures involving animals were performed in full compliance with the ethical standards of the Institutional Animal Care and Use Committee (IACUC) of the Second Affiliated Hospital of Zhejiang University, under an approved animal use protocol (approval number has been added to the revised manuscript).
Post-operative care, pain management, and monitoring schedules were followed rigorously to minimize animal suffering. Mice and rats were closely monitored during recovery from anesthesia and for signs of distress or infection throughout the study duration.
4.Randomization and Group Allocation:
Animals were randomly assigned to experimental groups using a computer-generated randomization sequence to avoid selection bias. Group assignment included: Sham, PBS control, Liposome@SPP1, @LEV@SPP1, and AF-PTK@LEV@SPP1 treatment groups.
Unless otherwise specified, each group consisted of a minimum of 3 animals, and sample sizes were determined based on prior power analysis and previously published studies of similar design.
5.Anesthesia and Surgical Details:
Anesthesia was induced using intraperitoneal injection of sodium pentobarbital at a dose of 40–50 mg/kg, and depth of anesthesia was confirmed via the loss of pedal reflex before any surgical or injection procedures.
For the IVDD model, percutaneous needle puncture was performed at the Co6–Co7 disc level using an 18G sterile needle, rotated 360° and held in position for 30 seconds to simulate mechanical injury.
Injections of therapeutic compounds were initiated 24 hours post-modeling, using a 33G microsyringe for precise intradiscal injection (see "Compound Administration Protocol" for full procedural description).
6.Consistency and Replication:
All animal experiments were conducted across at least three independent biological replicates, and procedures were carried out by trained personnel under standardized protocols to ensure consistency.
These expanded details have been integrated throughout the relevant subsections of the Materials and Methods, specifically within Animal Models, Lyz2-Cre;DTR Mice, Compound Administration Protocol, and Statistical Analysis. We thank you again for the helpful feedback, which has allowed us to strengthen the methodological rigor and clarity of our manuscript.
The procedures for mouse genotyping and transgene validation are described as follows(Page58)：
"Mouse Genotyping and Transgene Validation
Genomic DNA extraction and genotyping.
Genomic DNA was extracted from mouse tail, toe, or ear tissue using a one-step genomic DNA extraction kit (Cat# K9088, Magen Biotech, China) according to the manufacturer's protocol. Briefly, 10–50 mg of tissue was incubated with 150 μL Buffer L88-Plus and 5 μL Proteinase K at 60 °C until complete lysis (typically ≥4 h or overnight). After digestion, 300 μL of Buffer N2 (pre-mixed with Buffer N1) was added and mixed thoroughly to neutralize the reaction. The lysate was centrifuged briefly, and 3 μL of the supernatant was used for downstream PCR analysis.For samples requiring high-purity DNA (e.g., qPCR or difficult-to-amplify genotypes), DNA was extracted using a column-based purification kit (Cat# K9053, Magen Biotech). Tissues were digested in Buffer L and Proteinase K, followed by binding to a silica column, ethanol-based washing (Buffer W2), and elution with pre-warmed Buffer E at 65 °C. DNA quality was assessed via 1% agarose gel electrophoresis.
PCR reaction setup.
Standard PCR was performed in 25 μL total volume containing 12.5 μL 2× Taq Master Mix (Cat# C7014, Magen Biotech), 0.5 μL of each primer (10 μM), DNA template (100 ng–1 μg), and nuclease-free water. Thermal cycling conditions were as follows: 95 °C for 5 min; 35–40 cycles of 95 °C for 30 s, 58 °C for 30 s, and 72 °C for 60 s per

kb; with a final extension at 72 °C for 7 min.qPCR genotyping was carried out using 2× Probe qPCR Mix (Cat# C7018, Magen Biotech) in a 20 µL reaction system, including 0.8 µL primers (10 µM), 0.3 µL probes (10 µM), template DNA, and nuclease-free water. Fluorescence was detected during the annealing-extension phase (60 °C for 30 s) over 35–40 cycles, following denaturation at 95 °C for 10 s.

PCR product detection.

Amplicons were resolved by 1–2% agarose gel electrophoresis at 120 V for 20–30 min and visualized under UV illumination using a nucleic acid dye. Genotypes were interpreted by comparing band sizes to molecular weight markers (DL2000, D50, or Marker III, Magen Biotech). For ambiguous cases or SNP variants, Sanger sequencing was performed for confirmation."

Clarify the AF cell selection process for the peptide targeting AF cells.

RE:We sincerely thank you for this important and thoughtful comment. To enhance the specificity of the AF-targeting peptide, we employed a subtractive phage display screening strategy, and we have now clarified this process in the revised Phage Display section of the Materials and Methods.

Briefly, a Ph.D.-12 phage display peptide library was first incubated with mouse primary fibroblasts for negative selection, to eliminate phages that bind nonspecifically to cells with phenotypic similarity to AF cells. The unbound phages were then collected and subjected to positive selection by incubating with rat annulus fibrosus (AF) cells freshly isolated from intervertebral discs. After a 1-hour incubation at 37 °C, the cells were extensively washed to remove weakly bound or non-specific phages.

Phages strongly bound to AF cells were then eluted, amplified in E. coli ER2738, and used for the next round of selection. This biopanning process was repeated for three rounds to progressively enrich for phages displaying peptides with high specificity and affinity for AF cells. Phage titers were monitored in each round, and selected clones were sequenced to identify the peptide with the strongest binding affinity, which was used for subsequent in vitro and in vivo experiments.

This approach enabled us to effectively identify AF-targeting peptides while minimizing off-target interactions. Once again, we thank the reviewer for highlighting this important methodological point, which has now been clarified in the manuscript.

We are grateful for the reviewer's suggestion, which helped us enhance the clarity and completeness of this key methodological component.

"Phage Display **:**A linear 12-amino acid peptide library (Ph.D.-12 Phage Display Peptide Library, New England Biolabs, Inc.) was utilized for bio-panning to identify peptides with specific binding affinity. Initially, 1×10^{12} transduction units of the phage library were incubated with mouse primary fibroblasts for negative selection. The supernatant containing unbound phages was then collected and added to AF cells for positive selection. After a 1-hour incubation at 37°C, the cells were thoroughly washed five times with washing buffer (composition and pH of buffer can be specified here) to remove any non-specifically bound phages.Phages bound to the cells were subsequently eluted using an elution buffer (specify composition, pH, or concentration of buffer if necessary). The eluted phages were then amplified by infection of the Escherichia coli host strain ER2738, following the manufacturer's protocol. The resulting phage population was used for the subsequent round of selection. This process of negative and positive selection was repeated for a total of three rounds to enrich for phages displaying peptides with higher affinity and specificity for the target cells.For each round, the phage titres and the number of plaques were monitored, and a portion of the phages from each round was used for further characterization and sequencing."

Provide details on compound administration, including dosage, method, and timing of injection into intervertebral discs.

RE:We sincerely thank you for this valuable comment and the opportunity to improve the clarity of our methodology. We recognize that detailed information on compound administration is essential for reproducibility and transparency.

In response, we have carefully revised the Materials and Methods section to include comprehensive details regarding the injection procedure. Specifically, we have now added a new subsection titled "Compound Administration Protocol" under which we describe the dosage, injection method, anatomical site, and timing of administration as follows:

Therapeutic agents (AF-PTK@LEV@SPP1 and controls) were administered via percutaneous intradiscal injection into the Co6–Co7 intervertebral disc using a 33G Hamilton microsyringe. Each injection contained 10µL of nanoparticle solution, equivalent to 1 mg/kg of siRNA-SPP1. Injections were initiated 24 hours after disc injury modeling and were repeated once weekly for 8 consecutive weeks. The procedure was performed under sterile conditions and fluoroscopic guidance to ensure precision and minimize leakage.

We have also explicitly clarified in the text that all interventions were performed on the caudal intervertebral disc

(Co6–Co7) in rats, and not on lumbar discs. Corresponding clarifications were made in the figure legends where appropriate.
We greatly appreciate your suggestion, which has allowed us to strengthen the methodological rigor and clarity of the manuscript.

The discrepancy between the described disc degeneration model (L3/L4, L4/L5, or L5/L6 lumbar discs) and the figures suggesting caudal disc degeneration needs to be addressed.
RE:We thank the reviewer for highlighting this potential source of confusion. We would like to clarify that our intervertebral disc degeneration model was established at the caudal disc level (Co6–Co7) in rats, as described in the Materials and Methods section. No lumbar discs (L3/L4, L4/L5, or L5/L6) were involved in the procedure. To avoid any further ambiguity, we have now added explicit mentions of the disc level (Co6–Co7) in both the Methods and relevant figure legends. We hope this revision resolves the concern regarding anatomical discrepancy.
The corresponding materials& Methods are presented on Page62 as follow:
"Establishment of Intervertebral Disc Injury Model in Rats
Twelve-week-old male Sprague–Dawley rats were used to establish an intervertebral disc degeneration model. Under general anesthesia with pentobarbital sodium (50 mg/kg, i.p.), a needle puncture-induced degeneration procedure was performed at the coccygeal Co6–Co7 intervertebral disc. A sterile 18G needle was percutaneously inserted into the disc, rotated 360°, and held in place for 30 seconds to induce mechanical injury. This procedure has been widely validated for creating consistent and reproducible disc degeneration in rodent models. All surgical instruments were autoclaved, and the procedures were conducted under sterile conditions.
Compound Administration Protocol
To evaluate the therapeutic effects of AF-PTK@LEV@SPP1 in vivo, rats were anesthetized with pentobarbital sodium (50 mg/kg, i.p.) and placed in prone position. A 33G Hamilton microsyringe was used to deliver the nanoparticles via percutaneous intradiscal injection into the Co6–Co7 intervertebral disc under fluoroscopic guidance. Each rat received a 10 µL injection of nanoparticle suspension (equivalent to 1 mg/kg siRNA-SPP1), delivered slowly over 30 seconds to ensure even distribution and minimize leakage. Injections were initiated 24 hours post-surgery and repeated once weekly for 8 weeks. Control groups received 10 µL of PBS or empty liposome formulation under identical conditions. All procedures were conducted under sterile conditions and approved by the Institutional Animal Care and Use Committee of the Second Affiliated Hospital of Zhejiang University."

Clarify whether the AF-binding peptide also binds to mouse or human AF cells.
RE:Thank you very much for your thoughtful comment. In response, we performed additional in vitro experiments to evaluate the binding specificity of our AF-targeting peptide (P1) across different species.
As presented in Supplementary Fig. 6C, we incubated Cy5.5-labeled P1 (10 µM, 30 min) with primary annulus fibrosus (AF) cells isolated from rat, human, and mouse. Confocal microscopy images clearly show that P1 binds to all three types of AF cells, with minimal background observed in scrambled peptide and no-peptide controls.
Furthermore, we quantitatively analyzed the fluorescence intensity in these cells (Supplementary Fig. 6F). The results confirm that P1 exhibits statistically significant binding to both human and mouse AF cells, although the highest affinity was observed in rat AF cells. These data demonstrate that P1 maintains cross-species binding capability, supporting its translational relevance in both rodent models and potential human applications.
We sincerely appreciate your suggestion, which prompted us to strengthen our findings with these important experiments.The updated data was presented in Supplementary Fig. 6C , and the corresponding results are presented on Page23 as follow:
"We further investigated whether P1 could recognize AF cells across different species. Confocal microscopy after incubation with Cy5.5-labeled P1 showed robust binding to rat, human, and mouse AF cells (Supplementary Fig. 6C). Quantitative analysis (Supplementary Fig. 6F) revealed that although signal intensity was highest in rat cells, human and mouse AF cells also demonstrated consistent uptake, supporting the cross-species translational potential of P1 as an AF-targeting moiety.
Together, these results provide compelling evidence that P1 exhibits high affinity and specificity for annulus fibrosus cells in a sequence-dependent, cell-type–specific, and cross-species–compatible manner. Its prolonged in vivo retention and negligible off-target accumulation further support its application as a promising delivery vehicle for targeted therapeutic strategies in intervertebral disc degeneration."

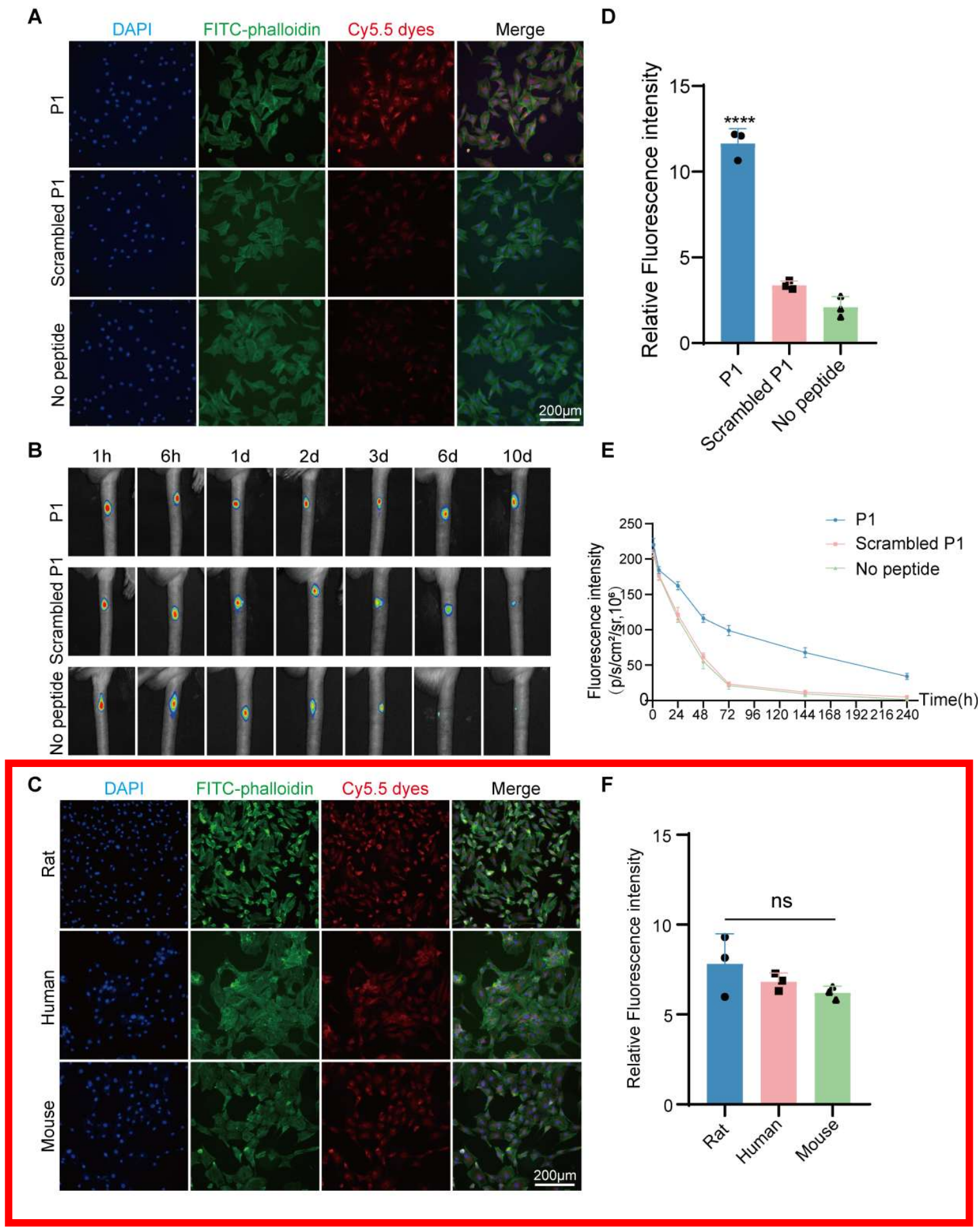

Supplementary Fig. 6.In vitro and in vivo evaluation of the binding affinity and specificity of P1 and scrambled P1.

(A) Confocal microscopy images showing the binding of P1， scrambled P1 and No peptide (Cy5.5-labeled) with FITC-labeled AF cells after incubation at 10 μM for 30 minutes. DAPI was used for nuclear staining.

(B) In vivo fluorescence imaging of the accumulation of P1，scrambled P1 and No peptide (Cy5.5-labeled) in the rat intervertebral discs at different time points (1h, 6h, 1d, 2d, 3d, 6d, 10d) post-injection.

(C) Confocal microscopy demonstrating the specificity of P1 (Cy5.5-labeled) with different cell types, including Rat AF cells, Human AF cells, and Mouse AF cells after incubation at 10 μM for 30 minutes.

(D) Quantification of relative fluorescence intensity in AF cells treated with P1, scrambled P1, or no peptide.

(E) Fluorescence intensity of P1， scrambled P1 and no peptide in rat intervertebral discs over time (1h to 240h) post-injection.

(F) Quantification of relative fluorescence intensity of P1 in Rat, Human, and Mouse AF cells. Data are presented as mean ± SD from at least three independent experiments. *$p < 0.05$, **$p < 0.01$, ***$p < 0.001$.

Specific details of degenerated discs of humans.

RE:Thank you for pointing this out, and this is an important question indeed.

Number of IVD Specimens Used

In our initial study, we performed single-cell RNA sequencing on three degenerated intervertebral disc (IVD) samples.To ensure greater scientific rigor, we deliberately included single-cell sequencing data from two additional patients, thereby strengthening the robustness and reliability of our study.In the current work, we have included two additional degenerated samples, and the findings remain consistent with our previous conclusions.In total, five human IVD specimens were collected and analyzed.All specimens were obtained from patients undergoing elective lumbar spine surgeries for degenerative spine diseases. Each disc specimen was used for subsequent single-cell RNA sequencing.This study was approved by the Institutional Review Board of Second Affiliated Hospital of Zhejiang University(approval number: 2024SQXB002446), and written informed consent was obtained from all participants.

Clinical Treatment of the Patients

All five patients had clear surgical indications including lumbar spinal stenosis, disc herniation, or spondylolisthesis, based on symptoms and imaging. Clinical treatment is summarized below(**Supplementary Fig. 1**):

Patient	Sex	Age	Diagnosis	Clinical Treatment (Surgery Type)	Tissue Site	Pfirrmann grade[1]
P1	Male	70	Lumbar spondylolisthesis, fracture	Posterior lumbar interbody fusion (L4/5), pedicle screw fixation	L4/5 IVD	IV
P2	Female	78	Lumbar stenosis & herniation	Lateral lumbar fusion and discectomy(L4/5,)	L4/5 IVD	IV
P3	Male	63	Lumbar stenosis	Posterior lumbar fusion (L4/5,L5/S1)	L4/5 , L5/S1 IVD	III
P4	Male	76	Lumbar spinal stenosis	Lateral lumbar interbody fusion (L3/4, L4/5), pedicle screw fixation	L3/4, L4/5 IVD	IV
P5	Female	67	Multilevel disc herniation and stenosis	Posterior fusion and decompression (L4/5)	L4/5 IVD	IV

Supplementary Fig. 1. Table(B)Clinical profiles of the corresponding patients, including sex, age, diagnosis, surgical procedure, tissue collection site, and Pfirrmann grade based on MRI analysis. The Pfirrmann grading system classifies the degree of intervertebral disc degeneration from grade I (healthy) to grade V (severely degenerated).

All patients received general anesthesia, and treatment included perioperative antibiotics (e.g., cefuroxime, cefazolin), pain management, and neuroprotective strategies. Postoperative monitoring focused on pain control, wound healing, and thrombosis prevention.

Determination of Disc Degeneration

Disc degeneration was evaluated using preoperative MRI and CT imaging, confirming disc bulging, Modic changes, and vertebral endplate alterations. MRI showed multi-level disc bulging, L4/5 and L5/S1 herniation, and facet joint degeneration. The Pfirrmann grade was assigned based on MRI findings according to the classification system proposed in Magnetic resonance classification of lumbar intervertebral disc degeneration by Pfirrmann et al. (2001).

Tissue Handling

During surgery, intervertebral disc tissue was extracted under sterile conditions. Prior to enzymatic digestion, the annulus fibrosus (AF) and nucleus pulposus (NP) were not separated based on anatomical features or tissue texture; instead, the entire disc was digested as a whole.

We have re-analyzed our single-cell RNA-seq data and revised the description of these results in the manuscript accordingly. We have also added Supplementary Figure 1 to clearly present the updated findings. The revised results are in Page10 as follows:

"To investigate the cellular composition of the intervertebral disc (IVD), we collected degenerated disc samples from five patients aged between 65 and 80 years, whose representative magnetic resonance imaging (MRI) features are shown in Supplementary Fig. 1A. Detailed patient information, diagnosis, clinical treatment methods, and Pfirrmann grading are presented in Supplementary Fig 1B.Single-cell suspensions were prepared from the whole degenerated discs and subjected to single-cell RNA sequencing (scRNA-seq). Utilizing the 10x Genomics platform in combination with microfluidics, we successfully isolated individual cells and performed high-throughput sequencing using the Illumina platform. This enabled rapid and efficient transcriptomic profiling and downstream analysis of thousands of single cells at high resolution (Fig. 1A).

In total, we obtained transcriptomic profiles from 53,416 cells across five samples, with 9,751 to 11,532 cells captured per sample. The median number of detected genes per cell ranged from 1,350 to 2,438. After quality filtering, between 7,814 and 9,298 high-quality cells were retained per sample. Unsupervised clustering analysis identified 21 distinct cell populations. Based on canonical marker expression and transcriptional profiles, the IVD microenvironment was classified into ten major cell types: nucleus pulposus (NP) cells, annulus fibrosus (AF) cells, macrophages, endothelial cells, plasma cells, myofibroblasts, monocytes, neutrophils, B cells, and T cells(Fig. 1B). Notably, aside from NP and AF cells, macrophages accounted for a substantial proportion of cells (Fig. 1C), constituting approximately 12.27% of all immune cells (Fig. 1D).These findings provide a comprehensive single-cell landscape of the degenerated IVD, highlighting the cellular heterogeneity and

implicating macrophages as a prominent immune cell population potentially involved in disc pathology."

References

1.Pfirrmann, C. W. A., Metzdorf, A., Zanetti, M., Hodler, J. & Boos, N. Magnetic resonance classification of lumbar intervertebral disc degeneration. Spine 26, 1873–1878 (2001).

Figures: Please improve figure legends and labels for better clarity. For example,

RE:We sincerely thank the reviewer for the insightful and constructive feedback regarding figure clarity and annotation. In response to all reviewers' comments, we have reorganized all figures throughout the manuscript. To meet the high standards of Nature Communications, we have undertaken substantial efforts to revise the presentation comprehensively.

Specifically, we carefully updated all figure legends and improved the labeling to enhance clarity, consistency, and interpretability.

To further improve the overall quality of the manuscript and better align it with publication standards, we have also added several supplementary figures. These additions offer more comprehensive support for our findings and provide clearer visual context for key results.

We genuinely appreciate your valuable suggestions, which have significantly contributed to improving our work.All modifications can be found in the revised manuscript.

Figure 1F: The distinction between normal vs. IVD is unclear.

RE: We have now clarified the comparison between the "normal" and "IVDD" groups by adding detailed annotations in the figure and revising the legend to explicitly define sample source, grading criteria, and histological differences. The new figure clearly shows morphological boundaries and disc structural integrity in the control group versus disrupted architecture in the IVDD group. We have also added arrowheads to highlight specific features in Page12 .

"To further explore the role of macrophages in degenerative disc disease, we generated a macrophage-specific fluorescent reporter mouse model (Lyz2-Cre;mG/mT), in which macrophages express membrane-localized tdTomato (red fluorescence), while all other cell types express membrane-localized GFP (green fluorescence), thereby allowing specific visualization of macrophages (Fig. 1E). To validate the genetic background of the model, genotyping by PCR was performed and confirmed the presence of both Lyz2-iCre and mG/mT alleles (Supplementary Fig. 2B, D). Strong GFP fluorescence was observed in the ears, paws, tail, and skin, consistent with Cre activity under the control of the Lyz2 promoter (Supplementary Fig. 2E),as indicated by the red arrows. Lyz2-Cre;mG/mT mice were divided into two groups: a normal control(Normal) group without tail-loop surgery and a tail-loop induced intervertebral disc degeneration (IVDD) group. The tail-loop model and its validation are shown in Supplementary Fig. 2F–G,and the modeling process was maintained for two weeks. Notably, minimal macrophage infiltration was observed in healthy IVDs, whereas prominent tdTomato-positive macrophages were detected within degenerated discs two weeks post tail-loop induction (Fig. 1G).Using a macrophage-specific fluorescent reporter mouse model (Lyz2-Cre;mG/mT), we demonstrated that macrophage infiltration is markedly increased in intervertebral discs following tail-loop-induced disc degeneration, in contrast to the minimal presence observed in healthy discs. This model enables precise in vivo visualization of macrophages and provides a robust platform for investigating immune cell dynamics during disc degeneration."

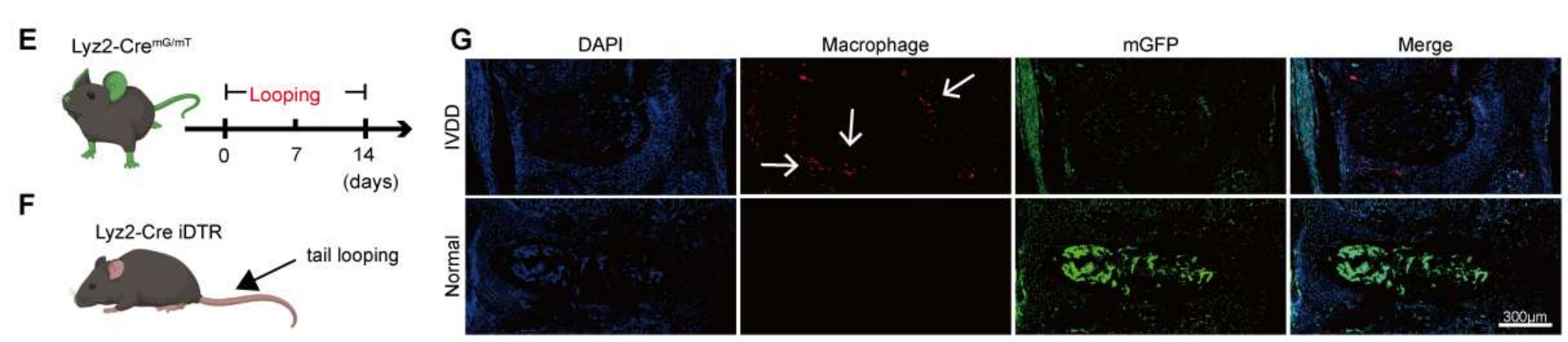

Figure 1. Macrophage Infiltration in Degenerating Intervertebral Discs.

(E) Schematic diagram of macrophage-specific fluorescent mice (Lyz2-Cre;mG/mT).

(F) Schematic diagram of macrophage-specific knockout mice (Lyz2-Cre;DTR).

(G)Fluorescent imaging of frozen sections from macrophage-specific fluorescent mice after tail-loop surgery(IVDD:Lyz2-Cre;mG/mT mice with tail-loop surgery;Normal:Lyz2-Cre;mG/mT mice without tail-loop surgery).

Figure 1I: The "normal" disc for MMP13 staining appears degenerated discs

RE:Thank you very much for your careful observation. We agree that the originally selected image for the "normal" disc in the MMP13 staining panel may not have fully represented the intended histological baseline. To improve the clarity and consistency of our data presentation, we have carefully reviewed our image library and selected a

new section from an age-matched disc that more accurately reflects a histologically normal condition. This updated image better aligns with the criteria used throughout the study. The figure legend has been revised accordingly to specify the disc grading for each condition.We greatly appreciate your attention to this detail, which has helped us improve the accuracy of our presentation.

We have now updated the figures accordingly, and the revised results are presented on Page13:

"To evaluate the structural and molecular consequences of macrophage depletion or supplementation during intervertebral disc degeneration, we performed histological and immunohistochemical analyses(Fig. 1H).Histological examination (H&E and Safranin O staining) revealed that the sham and Mac-Depletion groups preserved disc architecture following tail suspension, while the IVDD and Mac-Infusion groups exhibited characteristic degenerative features, including NP tissue loss, disorganized AF structure, and reduced disc height (Fig. 1H). Consistently, histological degeneration scoring (Masuda score, Supplementary Fig. 3A) showed a significantly higher score in the IVDD group and Mac-Infusion group, indicating severe degeneration, which was attenuated in the Mac-Depletion group.Immunohistochemical analysis showed significantly increased type II collagen (Col II) and decreased matrix metalloproteinase-13 (MMP13) expression in the Mac-Depletion group compared to the IVDD group and Mac-Infusion, suggesting a protective effect mediated by macrophage depletion (Fig. 1I,Supplementary Fig. 3B-C)."

Figures 7F & 8: the labels of these figures are difficult to read.

RE:In response to all reviewers' comments, we have reorganized all figures throughout the manuscript and incorporated extensive revisions. To meet the high publication standards of Nature Communications, we devoted considerable effort to improving figure presentation and overall clarity.

Specifically, for Figures 7F and 8, all graphical panels have been re-exported at higher resolution (600 dpi), and all text labels have been enlarged and bolded to enhance readability. We also adjusted the color contrast in low-visibility regions to improve visual clarity and standardized the label formats (e.g., treatment group names and molecular markers) across the figures.

To ensure tighter visual and logical coherence, we have merged the content of Figures 7F and 8 into a single, comprehensive figure. This integration allows a more streamlined and focused presentation of the data.

The updated figure legends now provide detailed descriptions for each panel, including sample sizes, staining markers, and statistical annotations.

All revised figures are included in the updated manuscript and supplementary materials. We sincerely thank you for helping us improve the quality and transparency of our data presentation. The revised results appear on Page 33–38 and Figure 7:"7.AF-PTK@LEV@SPP1 alleviates intervertebral disc degeneration by reducing inflammation and modulating lipid metabolism."

Typos: Acupuncture?

RE:Thank you for carefully reviewing our manuscript and pointing out this typographical error. You are absolutely right—this was an inadvertent mistake, and we have now corrected the misspelling accordingly in the revised version. We sincerely appreciate your attention to detail.

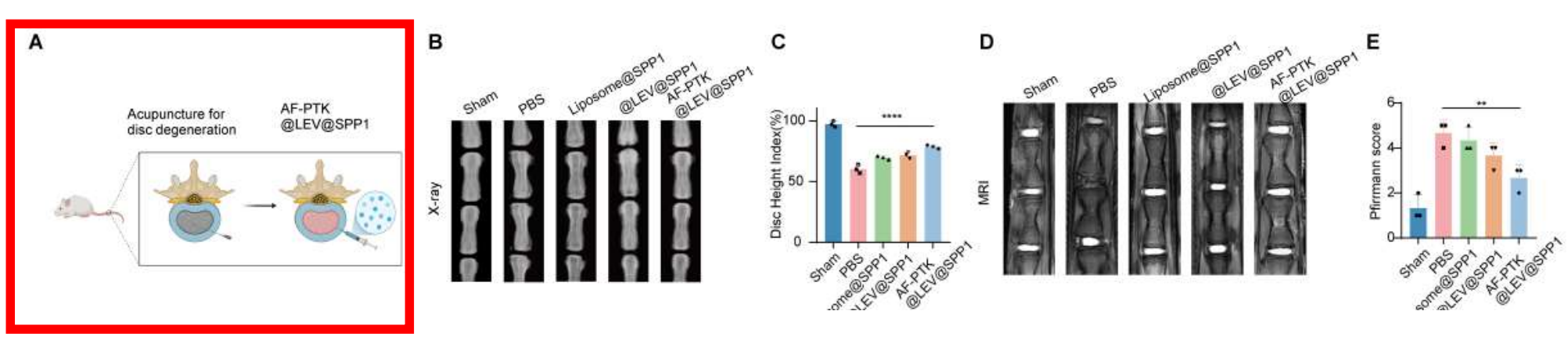

Figure 7. AF-PTK@LEV@SPP1 alleviates intervertebral disc degeneration by reducing inflammation and modulating lipid metabolism.

(A) Schematic illustration of AF-PTK@LEV@SPP1 treatment for acupuncture-induced intervertebral disc degeneration.

REVIEWER COMMENTS

Reviewer #1 (Remarks to the Author):

The authors have performed an amazing amount of additional analyses and even in vivo experiments and answered all of my questions.

RE: We sincerely appreciate the reviewer's very positive and encouraging comments. We are grateful that the reviewer found our additional analyses and in vivo experiments satisfactory and that all previous concerns have been fully addressed. We thank the reviewer for recognizing our efforts to comprehensively strengthen the study and clarify the mechanistic insights of AF-PTK@LEV@SPP1 in intervertebral disc degeneration. We have no further comments to add, and we deeply appreciate the reviewer's thoughtful evaluation and support for our work.

Reviewer #2 (Remarks to the Author):

The authors have addressed most of my concerns through additional data and revisions to the text. I have one remaining concern/comment. The SDS page study was to show that the OMV and liposome fusion occurred. However, it was difficult to see any significant differences between OMVs and OMVs + liposomes in the stained gel. Further mixing liposomes + OMVs without fusion will show the same banding pattern as all the components would be present in the mixed sample. I would suggest either removing this data or analyzing for specific lipid/protein components by confocal immunofluorescence to show co-labeling.

RE: We sincerely thank the reviewer for this valuable and constructive comment. We completely agree that SDS-PAGE alone may not provide conclusive evidence of true membrane fusion between OMVs and liposomes.

As demonstrated in several previous studies (1-5), SDS-PAGE combined with confocal co-localization has been widely employed to characterize membrane fusion between vesicular systems. In these works, SDS-PAGE was used to confirm the retention or integration of characteristic membrane proteins following fusion, while confocal microscopy provided direct visualization of dual-labeled lipid membranes (e.g., DiO/ DiI or FAM/DiI) to demonstrate spatial co-localization and fusion events.

Nonetheless, we fully agree with the reviewer that more definitive validation could be achieved by analyzing specific lipid or protein markers through confocal

immunofluorescence co-labeling. To avoid potential misinterpretation, we have therefore removed the SDS-PAGE data and confocal data from the revised manuscript, and reformatted Figure 4 and Supplementary Figure 7.

We greatly appreciate the reviewer's insightful suggestion, which has helped us enhance the scientific rigor and clarity of our study.

References

1.Pan, W. et al. Pyroptotic–Spatiotemporally Selective Delivery of siRNA against Pyroptosis and Autoimmune Diseases. Adv. Mater. 36, e2412345 (2024).

2.Xie, M. et al. Membrane Fusion–Mediated Loading of Therapeutic siRNA into Exosome for Tissue-Specific Application. Adv. Mater. 36, 2403935 (2024).

3.Zhang, Y. et al. Chondrocyte membrane–coated nanoparticles for targeted therapy of intervertebral disc degeneration. Sci. Transl. Med. 16, eabo1234 (2024).

4.Li, C. et al. Photosensitive hybrid tumor cell exosomes for synergistic photothermal–photodynamic immunotherapy. ACS Nano 19, 1435–1452 (2025).

5.Cheng, L. et al. Modulating the elasticity of milk exosomes to enhance their oral delivery efficiency via intestinal mucus. J. Control. Release 412, 72–85 (2025).

Reviewer #4 (Remarks to the Author):

Please see an attached report.

Reviewer #5 (Remarks to the Author):

I co-reviewed this manuscript with one of the reviewers who provided the listed reports. This is part of the Nature Communications initiative to facilitate training in peer review and to provide appropriate recognition for Early Career Researchers who co-review manuscripts.

The manuscript by Li et al. reports on the effects of OMV-liposomes targeting macrophage polarization in disc degeneration. The authors developed siRNA (Spp1)-loaded liposomes incorporating bacterial extracellular vehicles (EVs) to enhance macrophage uptake. An evaluation was made of whether the points raised by

the reviewer #3 had been satisfactorily addressed. The authors have made efforts to revise their manuscript; however, some concerns remain that need to be addressed.

RE: We sincerely thank Reviewer #4 and Reviewer #5 for their time, careful evaluation, and constructive feedback on our manuscript. We are also grateful to the Early Career Researcher who participated in the co-review as part of the Nature Communications initiative.

We truly appreciate the reviewers' acknowledgment of our revision efforts and fully recognize that some important concerns still remain. We will carefully address each of these points in a further, thorough revision of the manuscript, and we are grateful for the opportunity to improve the clarity, rigor, and impact of our work based on the reviewers' thoughtful suggestions.

1.Significant revision of the Introduction section is necessary, as it is too lengthy and insufficiently focused.

RE: We sincerely appreciate your insightful suggestion. The Introduction section has been substantially revised to improve focus and conciseness. Specifically, we shortened the general background on intervertebral disc degeneration (IVDD) and macrophage biology, and emphasized the knowledge gap regarding SPP1-mediated macrophage regulation within the avascular disc microenvironment. The revised introduction now clearly presents the rationale, hypothesis, and significance of our study within a more streamlined narrative.

We have revised the description of our results in Page4 as follow:

"Intervertebral disc degeneration (IVDD) is a major cause of chronic low back pain and disability, posing a substantial socio-economic burden worldwide(1-5). The difficulty in treating IVDD lies in its unique biological barriers(6-9). As the largest avascular tissue in the human body, the intervertebral disc has limited nutrient transport and immune accessibility(10, 11). The progressive degradation of the extracellular matrix (ECM) and the formation of a chronic inflammatory microenvironment make it difficult to reverse matrix destruction or regulate inflammation(12, 13). Specifically, the annulus fibrosus, with its dense and highly organized fibrous structure, resists passive molecular penetration and is prone to structural breakdown due to degeneration(14, 15), thereby promoting extracellular

matrix degradation and the formation of an inflammatory microenvironment. Moreover, the annulus fibrosus has a poor intrinsic regenerative capacity, further complicating attempts to restore disc homeostasis(16). Several studies have shown that microstructural damage to the annulus fibrosus is not only one of the early features of IVDD but also exacerbates nucleus pulposus herniation, induces local immune activation, and amplifies inflammatory responses, thereby accelerating the degenerative process(17-20). Damaged annulus fibrosus breaks the immune privilege of the nucleus pulposus, exposing antigenic components and triggering infiltration of T cells and macrophages along with a persistent inflammatory cascade(21, 22). In addition, annulus fibrosus cells undergo phenotypic changes and decreased activity during degeneration, with impaired responsiveness to mechanical stimuli, leading to disorganized fiber arrangement, rupture, and further initiating a vicious cycle(23, 24). Therefore, developing innovative therapeutic strategies that can overcome the intervertebral disc's unique biological barriers and specifically repair the damaged annulus fibrosus is a crucial breakthrough for achieving targeted intervention and stable modulation of the inflammatory microenvironment, as well as matrix regeneration and repair, to address the current clinical challenges of IVDD.

Recent studies suggest that immune dysregulation in the disc's pathological microenvironment is a key driver of annulus fibrosus destruction(25, 26). Among these, macrophages play a critical role in the pathological process. Due to the functional loss mediated by the imbalance of macrophage inflammatory states, features such as angiogenesis and matrix degradation are triggered(27, 28). In this study, our single-cell analysis confirmed significant macrophage accumulation in the degenerated intervertebral discs, and that macrophages exhibit substantial heterogeneity in the immune environment of degenerated discs. Through re-clustering analysis of macrophages, we identified two major subsets: pro-inflammatory and anti-inflammatory macrophages, with pro-inflammatory macrophages predominating in degenerated tissues. Further functional analysis revealed that pro-inflammatory macrophages are enriched in various immune-activation-related pathways, including Toll-like receptor signaling, PI3K-Akt signaling, and ECM-receptor interactions, all of which are closely related to the pathological process of disc degeneration. Previous studies have also confirmed that macrophages in degenerated discs predominantly polarize into the pro-inflammatory M1 phenotype, secreting cytokines such as TNF-α and IL-1β, activating matrix metalloproteinases (MMPs), and inducing apoptosis in

nucleus pulposus cells(29-31). In contrast, the M2 macrophage phenotype, associated with tissue repair, anti-inflammatory cytokine secretion, and ECM remodeling, is less frequently observed in degenerated discs(32-34). Our findings further confirm that secreted phosphoprotein 1 (SPP1) is highly expressed in pro-inflammatory macrophages. The expression of SPP1 is almost exclusively enriched in these macrophage subsets and is closely associated with tissue remodeling and chronic inflammation. Knockdown of SPP1 significantly reduces the proportion of M1 macrophages, alleviates the inflammatory response in disc degeneration, and may potentially reverse the degenerative process by modulating macrophage function. Therefore, the imbalance of macrophage states serves as a key driver of inflammation and further degeneration, and targeting SPP1-mediated macrophage function may represent an important strategy for reversing the progression of IVDD. Moreover, targeted modulation of macrophage function in the annulus fibrosus has emerged as a promising approach to regulate the local immune microenvironment and halt IVDD progression.

However, the anatomical structure and microenvironmental characteristics of the annulus fibrosus pose major challenges to targeting SPP1-mediated macrophage functions(35, 36). The extremely poor tissue permeability within the annulus fibrosus greatly limits the long-term retention and diffusion of therapeutic agents. In addition, the extracellular matrix of the annulus fibrosus lacks sufficient cell adhesion sites or receptors, making it difficult for most drugs or delivery systems to achieve " anchored binding"(37-40). In particular, macrophage infiltration exhibits "stress-triggered" and "time-dependent" characteristics—typically occurring only after mechanical injury, matrix rupture, or disruption of the immune barrier—rendering static delivery strategies ineffective for dynamic and precise regulation(41, 42). To address these challenges, gene therapy based on nucleic acid drugs targeting SPP1 has emerged as a powerful tool for modulating macrophage function, owing to its high specificity and programmability. However, siRNA molecules are unstable in the complex disc microenvironment, lack protective systems, and cannot actively target and penetrate the annulus fibrosus lesion areas, which significantly limits their practical intervention effects in vivo(43). Studies have demonstrated that bacterial outer membrane vesicles (OMVs), as natural delivery systems enriched with pathogen-associated molecular patterns, can be specifically

recognized by macrophage surface receptors(44, 45), making them promising platforms for siRNA-based interventions. Nevertheless, in highly immune-privileged tissues such as the intervertebral disc, OMVs may trigger additional inflammatory responses. Therefore, developing OMV-based nucleic acid delivery systems tailored for IVDD therapy holds great promise for regulating gene expression (e.g. SPP1) and reprogramming specific macrophage subpopulations within the complex pathological microenvironment of the intervertebral disc.

In this study, we identified the critical role of SPP1 as a key regulator of macrophage-mediated inflammation and ECM remodeling in IVDD. Its high expression in pro-inflammatory M1 macrophages promotes matrix degradation and inflammatory responses during the degeneration process. Overexpression of SPP1 not only exacerbates immune cell activation but also accelerates the degeneration of intervertebral discs by activating MMPs and inducing apoptosis of nucleus pulposus cells. Additionally, we, for the first time, used phage display technology to screen and identify a peptide that specifically targets annulus fibrosus (AF) cells, with the sequence SNTALPSKFYGY. Furthermore, to overcome the delivery barriers within the annulus fibrosus microenvironment and achieve precise regulation of macrophage function, we designed and constructed a responsive, targeted nano-therapy platform, named AF-PTK@LEV@SPP1. This platform uses an AF cell-specific peptide for stable anchoring and long-term retention in the annulus fibrosus region. Additionally, we utilized ROS-responsive thioketone linkers to sense local oxidative stress levels and trigger rapid drug release upon macrophage infiltration and inflammation, thereby enabling condition-dependent therapeutic activation. Moreover, by fusing detoxified bacterial outer membrane vesicles with liposomes, we created stable membrane fusion nanoparticles (LEV), significantly improving siRNA encapsulation and delivery efficiency. This system effectively targets inflammatory macrophages in lesion areas and silences the SPP1 gene. This delivery system not only achieves specific recognition and precise enrichment of target cells in space but also provides dynamic responsiveness over time, effectively blocking the pro-inflammatory polarization of macrophages mediated by SPP1, alleviating local inflammation, protecting matrix structures, and promoting tissue regeneration. Overall, our study highlights the potential of macrophage-centered immune modulation strategies for IVDD treatment, providing a new therapeutic path for immune function remodeling and disc

regeneration through a programmable, targeted, and dynamically responsive siRNA delivery system."

2.The methods and experimental procedures were strengthened through the inclusion of additional details provided on compound dosage and administration, as well as on the binding peptide.

RE: We sincerely thank you for acknowledging these improvements. The Methods section now includes precise details on compound dosage (siRNA concentration, liposome/OMV ratio, injection volume) and administration schedule. Moreover, we have added a description of the SPP1-binding peptide, including its sequence, source, and binding affinity characterization (Page68). These additions enhance the transparency and reproducibility of our experiments.

3.Regarding the rationale of the study, how did the authors select SPP1 as the target and determine its correlation with macrophage polarization? SPP1 is well known to promote macrophage polarization toward the M2 phenotype. Silencing SPP1 in M2 macrophages reverts them to an M1-like phenotype and inhibits tumor progression. Therefore, stronger supporting data are needed to link the authors' findings to macrophage polarization. Although several studies have reported that SPP1 promotes M2 polarization in cancer, pulmonary fibrosis, and related diseases, relatively few have associated SPP1 with inflammatory (M1-like) macrophages. This discrepancy should be addressed experimentally and thoroughly discussed in the manuscript.

RE: We sincerely thank the reviewer for this insightful comment, which prompted us to strengthen the rationale and provide direct experimental evidence supporting the role of SPP1 in macrophage polarization within the intervertebral disc (IVD) microenvironment. We agree that while SPP1 has been reported to promote M2 polarization in tumors and fibrotic diseases, its biological role is highly context-dependent. In our study, we focused on the IVD, a hypoxic, dense, and immune-isolated tissue, where SPP1 acts as a stress-induced inflammatory regulator driving macrophages toward an M1-like phenotype.

Lu, F.Y. et al. demonstrated that SPP1 serves as a key inflammatory mediator during chronic intermittent hypoxia, promoting TNF-α and IL-1β production and macrophage activation through NF-κB and STAT1 signaling [(1)]. Similarly, Tang, S. et al. reported that SPP1 activates ERK/JNK signaling and promotes M1 polarization with elevated TNF-α and IL-6 secretion in inflamed skin tissues [(2)]. Liu, C. et al. further identified SPP1^{+} macrophages as a pro-inflammatory subset in head and neck

carcinoma, secreting TNF-α and IL-1β through NF-κB activation[3]. Ellert-Miklaszewska, A. et al. clarified that tumor-processed SPP1 fragments promote M2 reprogramming, while full-length SPP1 from normal stromal cells maintains inflammatory activity [4]. Moreover, Liu, L.F. et al. confirmed that the SPP1–CD44 axis drives pro-inflammatory macrophage activation in metabolic inflammation [5]. Schuch, K. et al. showed that SPP1 exposure enhances macrophage activation and endocytosis even without typical M2 stimuli [6]. Collectively, these findings indicate that SPP1 acts as a microenvironment-sensitive inflammatory amplifier, particularly under hypoxia and stress conditions—features that closely resemble the degenerative disc microenvironment.

To experimentally verify this mechanism, we conducted additional macrophage polarization assays as recommended by the reviewer. Specifically, bone marrow–derived macrophages (BMDMs) were induced toward M1 polarization with LPS and IFN-γ, and toward M2 polarization with IL-4 and IL-13. These polarized cells were treated with either recombinant SPP1 (rSPP1) or our AF-PTK@LEV@SPP1 delivery system containing siSPP1. We then analyzed M1/M2 signature markers (CD86, iNOS, ARG1, CD206) and transcription factors (p-STAT1, p-STAT6, p-p65) by Western blot, and quantified TNF-α, IL-6, and IL-10 secretion by ELISA (Fig. 5 and Supplementary Fig. 8).

Our results revealed that SPP1 overexpression enhanced CD86 and iNOS in M1 macrophages while reducing ARG1 and CD206 in M2 macrophages, indicating a shift toward M1 polarization. Conversely, AF-PTK@LEV@SPP1 treatment reversed these effects, lowering CD86/iNOS expression and modestly restoring ARG1/CD206 levels. Consistent with these protein changes, ELISA results showed that SPP1 increased TNF-α and IL-6 secretion, whereas AF-PTK@LEV@SPP1 significantly decreased these cytokines and enhanced IL-10 production. Moreover, NF-κB p65 nuclear translocation assays confirmed that SPP1 stimulation promoted nuclear localization of p65 in M1 macrophages, while AF-PTK@LEV@SPP1 treatment effectively suppressed this translocation, indicating reduced NF-κB activation. Together, these findings provide direct mechanistic evidence that SPP1 promotes M1-like polarization through NF-κB activation, and that silencing SPP1 reverses this inflammatory bias.

In summary, our new experiments, combined with previous literature, demonstrate that SPP1 acts as a context-dependent regulator of macrophage polarization. While in tumor and fibrotic tissues processed SPP1 may support tissue remodeling and M2-like

responses, in the hypoxic and stress-prone IVD environment, upregulated SPP1 amplifies inflammation and drives M1 polarization. We have incorporated these new data and the expanded discussion into the revised manuscript to clarify the rationale for targeting SPP1 and to highlight its mechanistic role in macrophage-mediated inflammation during disc degeneration. (Page 28, Supplementary Fig. 8)

"To explore the effect of SPP1 on macrophage polarization, we used bone marrow-derived macrophages (BMDMs) and induced M1 polarization by LPS and IFN-γ, and M2 polarization by IL-4 and IL-13.In these induced macrophages, we treated recombinant SPP1 protein or our AF-PTK@LEV@SPP1 delivery system, respectively. We analyzed M1 and M2 type signature proteins (CD86, iNOS, ARG1, CD206) and polarization-related transcription factors (phosphorylation levels of STAT1, STAT6, p65) by Western blot (Fig. 5E-H). In addition, ELISA measured the secretion of inflammatory factors such as TNF-α, IL-6, and IL-10(Fig. 5I-J). Western blot analysis showed that in M1 macrophages, overexpression of SPP1 significantly enhanced the expression of CD86 and iNOS, while in M2 macrophages, overexpression of SPP1 decreased the expression of ARG1 and CD206. In addition, after treatment with AF-PTK@LEV@SPP1, the expression levels of CD86 and iNOS in M1 macrophages were reduced, while the expressions of ARG1 and CD206 in M2 macrophages were slightly increased. ELISA results further proved that high expression of SPP1 can promote the release of pro-inflammatory cytokines such as TNF-α and IL-6. At the same time, after treatment with AF-PTK@LEV@SPP1, the secretion of TNF-α and IL-6 was significantly reduced, while the secretion of IL-10 was increased. To further verify the role of NF-κB in macrophage polarization, we performed nuclear translocation experiments of NF-κB (Supplementary Fig. 8A-B). In the experiment, the nuclear translocation of macrophages induced by M1 polarization was detected under different treatment conditions (rSPP1 and AF-PTK@LEV@SPP1). The results showed that SPP1 treatment significantly increased the nuclear translocation of NF-kB p65, while AF-PTK@LEV@SPP1 treatment reduced the nuclear translocation of NF-kB, further supporting the role of SPP1 in M1 polarization (Supplementary Fig. 8C-D)."

References

1.Lu, F.Y. et al. Chronic intermittent hypoxia exacerbates the progression of NAFLD via SPP1-mediated inflammatory polarization in macrophages. Free Radic. Biol. Med. 2025; in press.

2.Tang, S. et al. OPN promotes pro-inflammatory cytokine expression via ERK/JNK pathway and M1 macrophage polarization in rosacea. Front. Immunol. 2024; 14:1285951.

3.Liu, C. et al. SPP1[+] macrophages promote head and neck squamous cell carcinoma progression through NF-κB–dependent secretion of TNF-α and IL-1β. J. Exp. Clin. Cancer Res. 2024; 43:332.

4.Ellert-Miklaszewska, A. et al. Tumour-processed osteopontin and lactadherin drive the protumorigenic reprogramming of microglia and glioma progression. Oncogene 2016; 35:4256–4269.

5.Liu, L.F. et al. The receptor CD44 is associated with systemic insulin resistance and proinflammatory macrophages in human adipose tissue. Diabetologia 2015; 58:1579–1591.

6.Schuch, K. et al. Osteopontin affects macrophage polarization promoting endocytic but not inflammatory properties. Obesity 2016; 24:1679–1689.

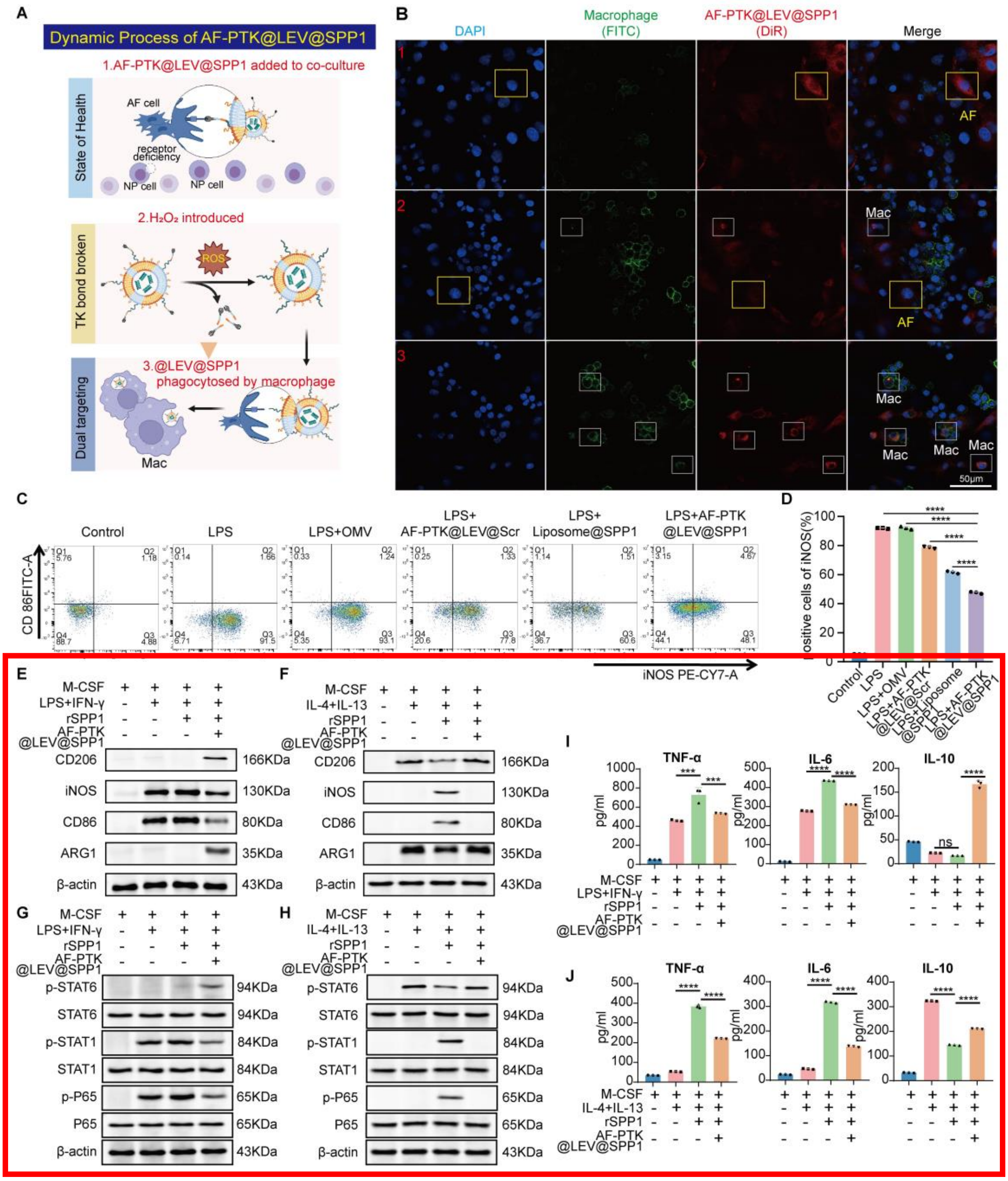

Figure 5. ROS-responsive intercellular transfer of AF-PTK@LEV@SPP1 nanoparticles reprograms macrophage polarization to mitigate intervertebral disc degeneration.

(A) Schematic illustration of the dynamic phagocytosis process of AF-PTK@LEV@SPP1 by annulus fibrosus cells and bone-marrow-derived M1 macrophages (BMDM-M1) in sequence.

(B) Confocal microscopy showing the phagocytosis of AF-PTK@LEV@SPP1: nanoparticles were initially taken up by AF cells and subsequently engulfed by macrophages after co-culture. DAPI (nuclei, blue), FITC (macrophages, green), and DiR (AF-PTK@LEV@SPP1, red). Scale bar = 50 μm.

(C) Flow cytometric analysis of macrophages stained for CD86 and iNOS under different treatment conditions.

(D) Quantification of iNOS-positive macrophages based on flow cytometry from (C).

(E-H) Representative Western blot results showing total and phosphorylated levels of STAT1, STAT6, and P65, as well as M1/M2 markers (iNOS, CD86, CD206, and Arg1) in BMDMs under indicated treatments.
(I,J)ELISA results showing TNF-α, IL-6, and IL-10 secretion in different groups.
Data are presented as mean ± SD from at least three independent experiments. Statistical significance was assessed using one-way ANOVA with appropriate post hoc tests. * indicates $p < 0.05$, ** indicates $p < 0.01$, *** indicates $p < 0.001$, **** indicates $p < 0.0001$.

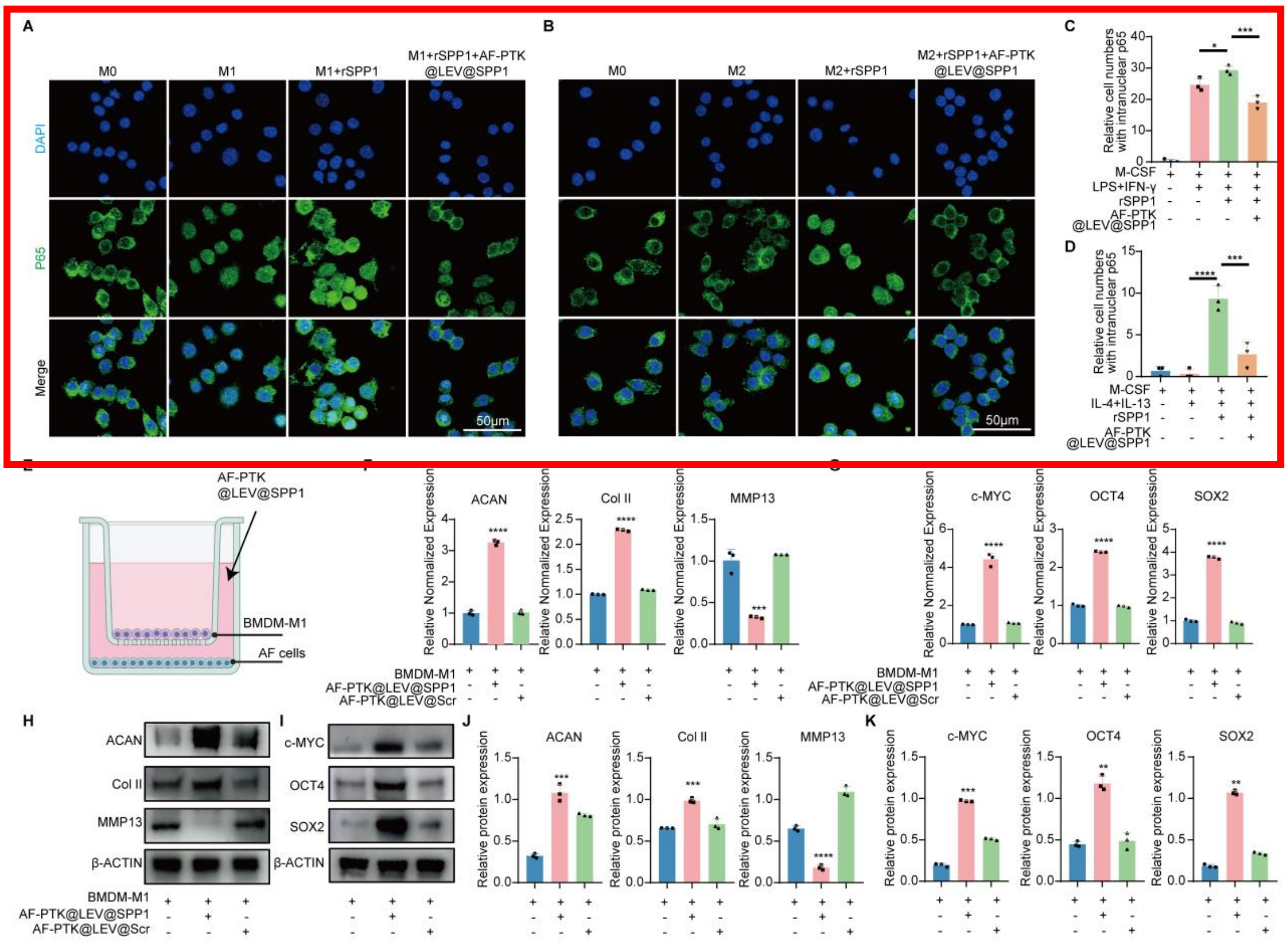

Supplementary Fig. 8.NF-κB signaling and paracrine crosstalk mediate the AF-PTK@LEV@SPP1-induced restoration of annulus fibrosus cell homeostasis.
(A, B) Immunofluorescence images showing NF-κB p65 nuclear translocation in each treatment group.
(C, D) The number of cells with intranuclear p65.The results were normalized and analyzed using student t test (mean±SD, n = 3)
(E)Schematic diagram of the Transwell co-culture system with BMDM-M1 and AF cells exposed to different treatments.
(F, G) Quantitative real-time PCR analysis of AF cell gene expression, including aggrecan (ACAN), collagen type II (Col II), matrix metalloproteinase 13 (MMP13), and stemness-associated genes (OCT4,SOX2, c-MYC), following indirect exposure to treated BMDMs.
(H, I) Western blot analysis of the indicated proteins in AF cells co-cultured with BMDMs pretreated as shown.
(J, K) Protein (Western Blot) quantification of in Supplementary Fig. 8.(H-I). *$p < 0.05$, **$p < 0.01$, ***$p < 0.001$.****$p < 0.0001$.

4.There is concern about the polarization assay. A macrophage polarization assay in the presence or absence of recombinant SPP1 is recommended, using LPS and IFN-γ to induce M1 polarization and IL-4 to induce M2 polarization. Alternatively, the constructed liposome could be used in these assays. In addition, Western blot analysis of transcription factors and M2- specific markers should be performed in parallel. The current data, showing only CD68 and iNOS expression, are insufficient to support conclusions regarding macrophage polarization.

RE: We sincerely thank the reviewer for this insightful comment regarding the macrophage polarization assay. To address this concern and to more rigorously evaluate the role of SPP1 in macrophage polarization, we conducted a series of additional experiments designed to dissect both the direct effects of SPP1 and the modulatory function of our AF-PTK@LEV@SPP1 delivery system. The new results are presented in Figure 5 and Supplementary Figure 8, and are described in detail on Page28 of the revised manuscript.

To better capture the dynamics of macrophage polarization, we first isolated bone marrow–derived macrophages (BMDMs) and induced classical M1 and alternative M2 phenotypes using LPS + IFN-γ and IL-4 + IL-13, respectively. These polarized macrophages were subsequently treated with recombinant SPP1 (rSPP1) or with our AF-PTK@LEV@SPP1 formulation, thereby allowing direct comparison of the effects of SPP1 and the engineered delivery system under physiologically relevant conditions.

To characterize phenotypic changes at the molecular level, we performed Western blot analyses for key M1/M2 signature markers (iNOS, CD86, ARG1, CD206) as well as polarization-related transcription factors (STAT1, STAT6, and phosphorylated p65). Consistent with a pro-inflammatory role, SPP1 stimulation markedly upregulated CD86 and iNOS expression, while downregulating ARG1 and CD206, indicating an enhancement of M1 polarization and suppression of M2 features. In contrast, treatment with AF-PTK@LEV@SPP1 reversed these effects, attenuating M1-associated protein expression and partially restoring M2 marker levels (Fig. 5E–H).

To further verify these phenotypic observations, we measured cytokine secretion profiles by ELISA. In agreement with the protein expression data, SPP1 significantly increased TNF-α and IL-6 levels, whereas AF-PTK@LEV@SPP1 treatment reduced these pro-inflammatory cytokines and elevated IL-10 secretion (Fig. 5I–J). These results collectively suggest that our delivery system effectively counteracts SPP1-driven inflammatory polarization.

To elucidate the underlying mechanism, we examined NF-κB signaling via p65 nuclear translocation assays. Immunofluorescence analysis revealed that SPP1 markedly promoted nuclear localization of NF-κB p65 in M1 macrophages, while AF-PTK@LEV@SPP1 treatment substantially inhibited this translocation (Supplementary Fig. 8A–D), indicating suppression of NF-κB activation.

Together, these findings demonstrate that SPP1 promotes pro-inflammatory M1 polarization and cytokine release, whereas AF-PTK@LEV@SPP1 treatment reverses

these effects by suppressing NF-κB signaling and partially restoring M2 features. These additional data provide solid mechanistic evidence supporting our conclusion that targeting SPP1 signaling can modulate macrophage polarization within the intervertebral disc microenvironment.

We have revised the description of our results in Page28 as follows:

"To explore the effect of SPP1 on macrophage polarization, we used bone marrow-derived macrophages (BMDMs) and induced M1 polarization by LPS and IFN-γ, and M2 polarization by IL-4 and IL-13. In these induced macrophages, we treated recombinant SPP1 protein or our AF-PTK@LEV@SPP1 delivery system, respectively. We analyzed M1 and M2 type signature proteins (CD86, iNOS, ARG1, CD206) and polarization-related transcription factors (phosphorylation levels of STAT1, STAT6, p65) by Western blot (Fig. 5E-H). In addition, ELISA measured the secretion of inflammatory factors such as TNF-α, IL-6, and IL-10(Fig. 5I-J). Western blot analysis showed that in M1 macrophages, overexpression of SPP1 significantly enhanced the expression of CD86 and iNOS, while in M2 macrophages, overexpression of SPP1 decreased the expression of ARG1 and CD206. In addition, after treatment with AF-PTK@LEV@SPP1, the expression levels of CD86 and iNOS in M1 macrophages were reduced, while the expressions of ARG1 and CD206 in M2 macrophages were slightly increased. ELISA results further proved that high expression of SPP1 can promote the release of pro-inflammatory cytokines such as TNF-α and IL-6. At the same time, after treatment with AF-PTK@LEV@SPP1, the secretion of TNF-α and IL-6 was significantly reduced, while the secretion of IL-10 was increased. To further verify the role of NF-κB in macrophage polarization, we performed nuclear translocation experiments of NF-κB (Supplementary Fig. 8A-B). In the experiment, the nuclear translocation of macrophages induced by M1 polarization was detected under different treatment conditions (rSPP1 and AF-PTK@LEV@SPP1). The results showed that SPP1 treatment significantly increased the nuclear translocation of NF-kBp65, while AF-PTK@LEV@SPP1 treatment reduced the nuclear translocation of NF-kB, further supporting the role of SPP1 in M1 polarization (Supplementary Fig. 8C-D)."

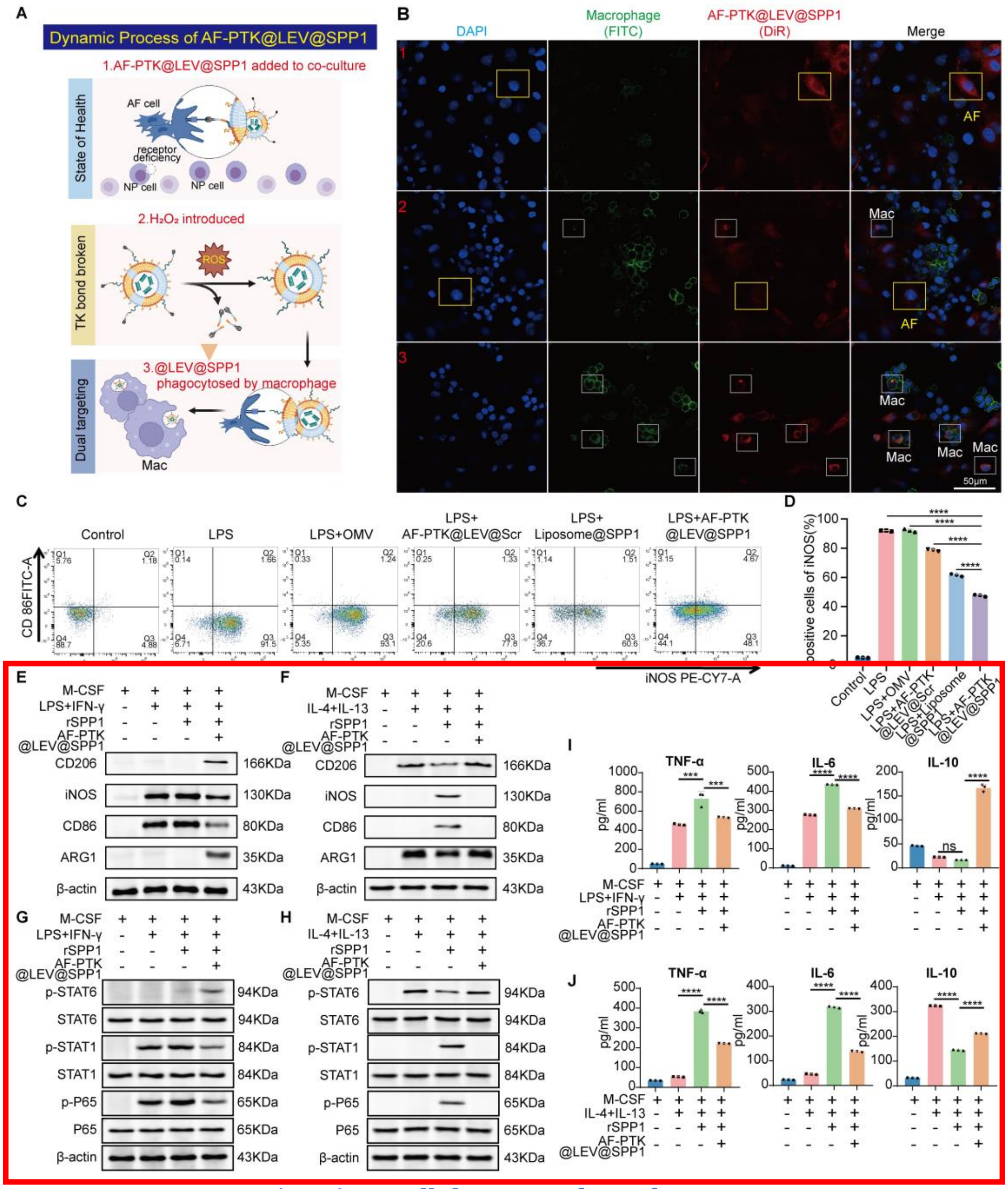

Figure 5. ROS-responsive intercellular transfer of AF-PTK@LEV@SPP1 nanoparticles reprograms macrophage polarization to mitigate intervertebral disc degeneration.

(A) Schematic illustration of the dynamic phagocytosis process of AF-PTK@LEV@SPP1 by annulus fibrosus cells and bone-marrow-derived M1 macrophages (BMDM-M1) in sequence.

(B) Confocal microscopy showing the phagocytosis of AF-PTK@LEV@SPP1: nanoparticles were initially taken up by AF cells and subsequently engulfed by macrophages after co-culture. DAPI (nuclei, blue), FITC (macrophages, green), and DiR (AF-PTK@LEV@SPP1, red). Scale bar = 50 μm.

(C) Flow cytometric analysis of macrophages stained for CD86 and iNOS under different treatment conditions.

(D) Quantification of iNOS-positive macrophages based on flow cytometry from (C).

(E-H) Representative Western blot results showing total and phosphorylated levels of STAT1, STAT6, and P65, as well as M1/M2 markers (iNOS, CD86, CD206, and Arg1) in BMDMs under indicated treatments.
(I, J) ELISA results showing TNF-α, IL-6, and IL-10 secretion in different groups.
Data are presented as mean ± SD from at least three independent experiments. Statistical significance was assessed using one-way ANOVA with appropriate post hoc tests. * indicates $p < 0.05$, ** indicates $p < 0.01$, *** indicates $p < 0.001$, **** indicates $p < 0.0001$.

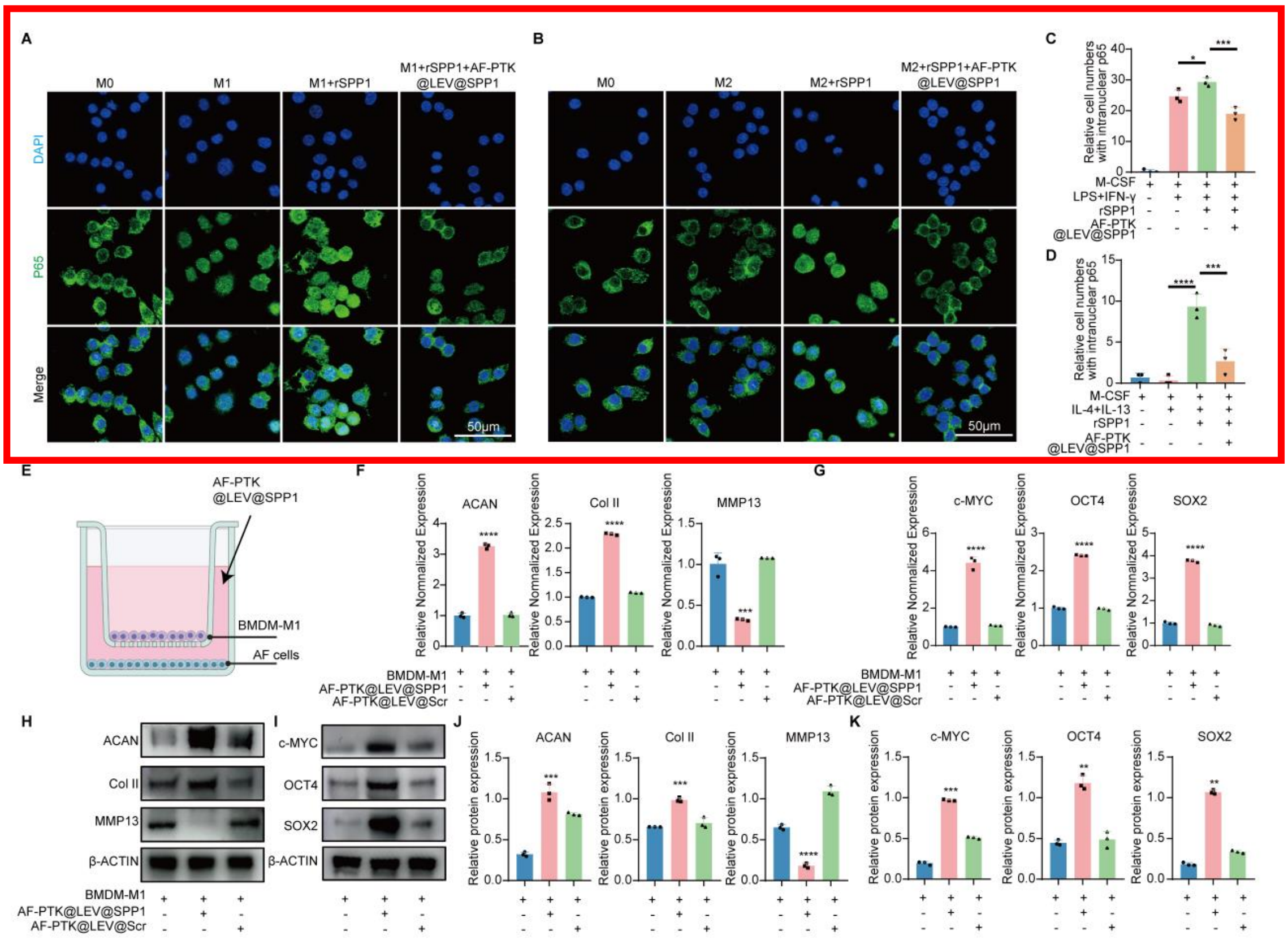

Supplementary Fig. 8.NF-κB signaling and paracrine crosstalk mediate the AF-PTK@LEV@SPP1-induced restoration of annulus fibrosus cell homeostasis.

(A, B) Immunofluorescence images showing NF-κB p65 nuclear translocation in each treatment group.

(C, D) The number of cells with intranuclear p65.The results were normalized and analyzed using student t test (mean±SD, n = 3)

(E)Schematic diagram of the Transwell co-culture system with BMDM-M1 and AF cells exposed to different treatments.

(F, G) Quantitative real-time PCR analysis of AF cell gene expression, including aggrecan (ACAN), collagen type II (Col II), matrix metalloproteinase 13 (MMP13), and stemness-associated genes (OCT4, SOX2, c-MYC), following indirect exposure to treated BMDMs.

(H, I) Western blot analysis of the indicated proteins in AF cells co-cultured with BMDMs pretreated as shown.

(J, K) Protein (Western Blot) quantification of in Supplementary Fig. 8. H-I. *$p < 0.05$, **$p < 0.01$, ***$p < 0.001$.****$p < 0.0001$.

5.The authors highlighted the distribution of macrophages in the IVD, and the figure has been improved by adding of arrows.

RE: We sincerely appreciate your positive feedback. The revised Figure 1 now clearly identifies macrophage localization within the annulus fibrosus and nucleus pulposus, using arrows and consistent color labeling. The figure legend has also been expanded for clarity.

6.The bulk RNA-seq data presented in Figure 7 require further clarification. The presented results are confusing and inconsistent with the authors' statements. While the authors report that Arg1 and Timp1 are upregulated in the treated group, the figure clearly shows that they are downregulated. Only PPARγ appears to be slightly upregulated. Based on these data, silencing SPP1 seems to enhance inflammation, as NFKB and RA and Il-17 signaling pathways are among the most significantly enriched in the pathway analysis. For the GSEA analysis, please provide the exact p-values and clearly indicate the positions of the control and treated groups with the enrichment plots. Furthermore, the GSEA results are inconsistent with the pathway enrichment analysis, which showed upregulation of inflammation-related terms. This discrepancy should be carefully addressed, and the corresponding statements revised accordingly. In addition, the quality of the figure should be improved. The RNA-seq findings need to be supported by immunohistochemistry (IHC), particularly with data demonstrating polarized macrophages in treated mice, which is essential for supporting the study's conclusions. Finally, a heatmap of genes enriched in the top pathways should be provided to enhance clarity and transparency.

RE: We sincerely thank the reviewer for this valuable and detailed feedback. We have carefully reanalyzed, corrected, and clarified all RNA-seq-related data and figures to ensure full consistency between our results and statements.

First, we acknowledge that in our initial submission, the description of Arg1 and Timp1 expression was inaccurate. After re-examining the RNA-seq data, we confirm that both Arg1 and Timp1 are downregulated at the mRNA level in AF-PTK@LEV@SPP1-treated discs.

It is well recognized that transcriptional and protein-level regulation may differ due to post-transcriptional mechanisms and cell-type heterogeneity within disc tissues, which is well documented and can result from post-transcriptional regulation (translation rates, RNA/protein stability, degradation) and bulk-tissue cellular heterogeneity (cell-type composition differences that affect tissue-average RNA signals)[1-5].Therefore, to strengthen our conclusion, we conducted additional immunohistochemistry (IHC) experiments for ARG1 (M2 macrophage marker) and iNOS (M1 marker) as suggested. The IHC results (now presented in Supplementary Figure 9D) clearly demonstrate increased ARG1 and reduced iNOS expression in AF-PTK@LEV@SPP1-treated discs, confirming macrophage polarization toward an anti-inflammatory M2 phenotype at the protein level.

To further minimize potential confusion for readers, we have removed Arg1 and Timp1 from the focused RNA-seq heatmap panels and comprehensively updated Figure 7 to reflect the corrected RNA-seq interpretation. More specifically, the volcano plot (Fig. 7M) now highlights representative downregulated inflammatory genes (Nos2, Ccl2, Il6, Mmp13) and upregulated metabolic genes (Pparg, Fabp4, Lpl, Nfkbia); the inflammation-axis heatmap (Fig. 7N) displays chemokines/cytokines/ECM remodeling genes that are reduced in the treated group; the PPAR/lipid-metabolism heatmap (Fig. 7O) shows metabolic genes that are increased (with Nos2 displayed as a counter-example that is decreased). Together, these revisions align the RNA-seq presentation with our textual statements and the IHC evidence.

For KEGG enrichment vs. GSEA orientation and statistics. We have separated downregulated vs. upregulated pathways into two plots for clarity: 1. Fig. 7P (Down): Cytokine–cytokine receptor interaction, NF-κB, TNF, Chemokine, JAK–STAT,

Toll-like receptor, NOD-like receptor, and C-type lectin receptor signaling are suppressed in the treated group. 2. Fig. 7Q (Up): PPAR, AMPK, adipocytokine signaling, regulation of lipolysis, fatty-acid metabolism/degradation, and peroxisome pathways are activated in the treated group.

In addition, GSEA panels now display exact NES, nominal p-values, and FDR q-values on each plot, and we explicitly annotate which curve corresponds to AF-PTK@LEV@SPP1 vs. IVDD. For example, PPAR signaling shows positive enrichment in AF-PTK@LEV@SPP1 (Fig. 7R; NES = 1.95, $p = 5.36\times10^{-6}$, $q = 1.4\times10^{-3}$). Conversely, Cytokine–cytokine receptor, NF-κB, and Chemokine pathways show negative enrichment in AF-PTK@LEV@SPP1 (Supplementary Fig. 9A–C), thereby resolving the prior discrepancy and demonstrating a coherent pattern: inflammation down, lipid/PPAR programs up.

We also improved figure resolution, modified the discussion section, unified fonts/labels/color scales, and added pathway-focused heatmaps of genes enriched in the top pathways (Fig. 7N–O), as requested, to enhance clarity and transparency.

We are grateful to the reviewer for these recommendations, which markedly improved the rigor and readability of our RNA-seq presentation, and we hope the revised figures and explanations satisfactorily address all concerns.

We have revised the description of our results in Page36 as follows:

"To elucidate the molecular mechanisms underlying the protective effects of AF-PTK@LEV@SPP1, we performed bulk RNA-seq on intervertebral disc (IVD) tissues collected from rats treated with AF-PTK@LEV@SPP1 or PBS (IVDD group).

Hierarchical clustering and global heatmap analysis revealed distinct transcriptomic profiles between groups, with extensive gene expression reprogramming in AF-PTK@LEV@SPP1-treated discs compared with the degenerative expression pattern of PBS controls (Figure 7L). Volcano plot analysis identified 1,102 genes significantly upregulated and 871 genes downregulated after AF-PTK@LEV@SPP1 treatment (Figure 7M). Pro-inflammatory mediators such as Nos2, Ccl2, Il6, and Mmp13 were markedly decreased, while metabolic and reparative regulators including Pparg, Fabp4, Lpl, and Nfkbia were upregulated, indicating a coordinated anti-inflammatory and metabolic adaptive response.

Targeted heatmaps further demonstrated two opposite gene-expression programs (Figures 7N–O).Inflammation-related genes involved in the TNF, NF-κB, and IL-17 pathways—including Cxcl1, Cxcl2, Cxcl3, Ccl2, Ccl3, Ccl4, Il1b, Il6, Mmp9, Mmp13, Ptgs2, Nfkbia, and Tnfaip3—were strongly downregulated in the AF-PTK@LEV@SPP1 group (Figure 7N).Conversely, genes associated with lipid metabolism and PPAR activation, such as Pparg, Fabp4, Lpl, Acsl1, Aqp7, Hmgcs2, Sorbs1, Rxrg, Slc27a6, and Ehhadh, were significantly increased, whereas the M1-polarization marker Nos2 was reduced (Figure 7O). These results suggest that AF-PTK@LEV@SPP1 promotes lipid metabolic homeostasis and M2-like macrophage polarization while suppressing pro-inflammatory cascades.

KEGG pathway enrichment analysis confirmed that downregulated genes were primarily enriched in Cytokine–cytokine receptor interaction, NF-κB, TNF,

Chemokine, JAK–STAT, Toll-like receptor, NOD-like receptor, and C-type lectin receptor signaling pathways (Figure 7P).In contrast, upregulated genes were enriched in PPAR signaling, AMPK signaling, adipocytokine signaling, regulation of lipolysis in adipocytes, fatty-acid metabolism and degradation, and peroxisome pathways (Figure 7Q).

Consistently, gene set enrichment analysis (GSEA) revealed positive enrichment of the PPAR signaling pathway (NES = 1.95, p = 5.36 × 10-6, q = 1.4 × 10-3) in the AF-PTK@LEV@SPP1 group (Figure 7R), while Cytokine–cytokine receptor, NF-κB, and Chemokine pathways were negatively enriched (Supplementary Fig. 9A–C), confirming global suppression of inflammatory gene programs.Finally, immunohistochemistry staining of ARG1 (M2 marker) and iNOS (M1 marker) provided cellular validation: ARG1 expression was elevated, whereas iNOS expression was reduced in the AF-PTK@LEV@SPP1 group compared with PBS or liposome controls (Supplementary Fig. 9D). Together, these transcriptomic and histological data demonstrate that AF-PTK@LEV@SPP1 mitigates intervertebral disc degeneration through dual modulation of immune–inflammatory suppression and PPAR-mediated lipid metabolic activation, restoring immune-metabolic homeostasis in degenerative discs.”

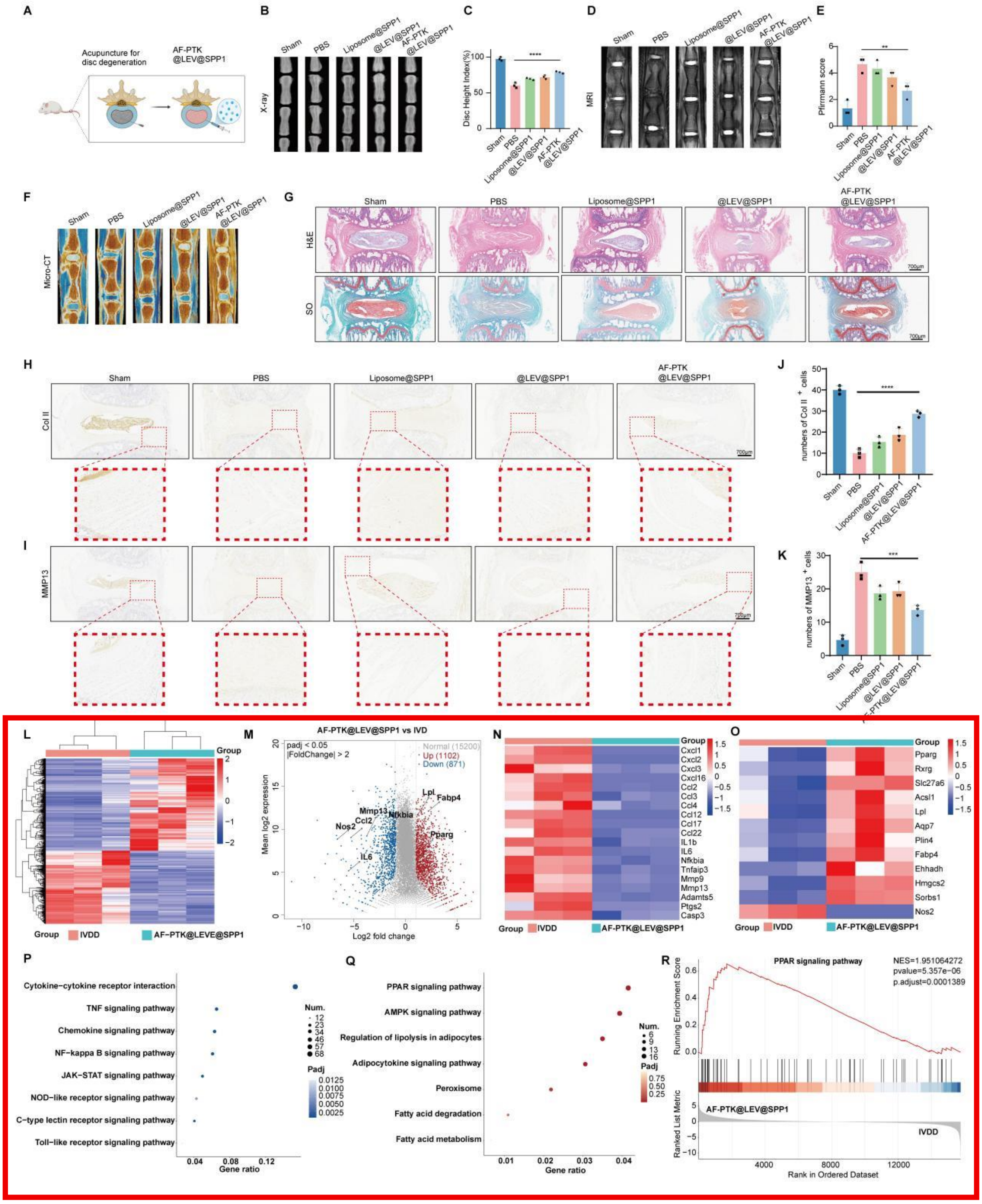

Figure 7. AF-PTK@LEV@SPP1 alleviates intervertebral disc degeneration by reducing inflammation and modulating lipid metabolism.

(A) Schematic illustration of AF-PTK@LEV@SPP1 treatment for acupuncture-induced intervertebral disc degeneration.

(B)Representative X-ray images of intervertebral discs across different treatment groups after 8 weeks (Sham, PBS, Liposome@SPP1, @LEV@SPP1, AF-PTK@LEV@SPP1).

(C)Quantification of disc height index from X-ray data in different treatment groups.

(D)Representative MRI images of intervertebral discs in different treatment groups.

(E)Quantification of Pfirrmann scores from different treatment groups indicating degeneration severity.

(F)Micro-CT images showing bone architecture changes in intervertebral discs across treatment groups.

(G)H&E and Safranin O staining of intervertebral discs at 8 week in different treatment groups.

(H, I) Representative immunohistochemical staining of Col II and MMP13 in intervertebral discs after different treatments.

(J, K) Representative statistical analysis of immunohistochemical staining for Col II and MMP13 in intervertebral discs after different treatments.

(L) Heatmap showing global mRNA expression profiles of rat intervertebral discs following acupuncture-induced degeneration and subsequent treatment with PBS (IVDD) or AF-PTK@LEV@SPP1. Distinct clustering demonstrates transcriptomic divergence between groups.

(M) Volcano plot illustrating significantly upregulated (red) and downregulated (blue) genes in AF-PTK@LEV@SPP1-treated discs compared with the IVDD group ($|log_2FC| > 2$, $p < 0.05$). Key genes involved in inflammation (Nos2, Ccl2, Il6, Mmp13) and lipid metabolism (Pparg, Fabp4, Lpl, Nfkbia) are highlighted.

(N) Heatmap of inflammation- and extracellular matrix (ECM)-related genes (Cxcl1, Cxcl2, Cxcl3, Cxcl16, Ccl2, Ccl3, Ccl4, Ccl12, Ccl17, Ccl22, Il1b, Il6, Nfkbia, Tnfaip3, Mmp9, Mmp13, Adamts5, Ptgs2, Casp3), showing broad downregulation in AF-PTK@LEV@SPP1-treated discs compared to IVDD controls.

(O) Heatmap of lipid metabolism and PPAR signaling associated genes (Pparg, Fabp4, Lpl, Plin4, Acsl1, Aqp7, Hmgcs2, Sorbs1, Rxrg, Slc27a6, Ehhadh), which were upregulated in AF-PTK@LEV@SPP1-treated discs, whereas the M1 marker Nos2 was reduced, indicating a shift toward anti-inflammatory and metabolic homeostasis.

(P) KEGG enrichment analysis of downregulated genes highlighting suppressed inflammatory pathways, including Cytokine–cytokine receptor interaction, NF-κB signaling, TNF signaling, Chemokine signaling, JAK–STAT, Toll-like receptor, NOD-like receptor, and C-type lectin receptor signaling.

(Q) KEGG enrichment analysis of upregulated genes identifying activation of PPAR signaling, AMPK signaling, adipocytokine signaling, regulation of lipolysis in adipocytes, fatty acid metabolism and degradation, and peroxisome pathways.

(R)Gene set enrichment analysis (GSEA) showing positive enrichment of the PPAR signaling pathway (NES = 1.95, p = 5.36 × 10-6, q = 1.4 × 10-3) in the AF-PTK@LEV@SPP1 group compared to IVDD controls, confirming activation of lipid-metabolic and anti-inflammatory signaling.

Data are presented as the mean ± SD from at least three independent experiments. *$p < 0.05$, **$p < 0.01$, ***$p < 0.001$, ****$p < 0.0001$.

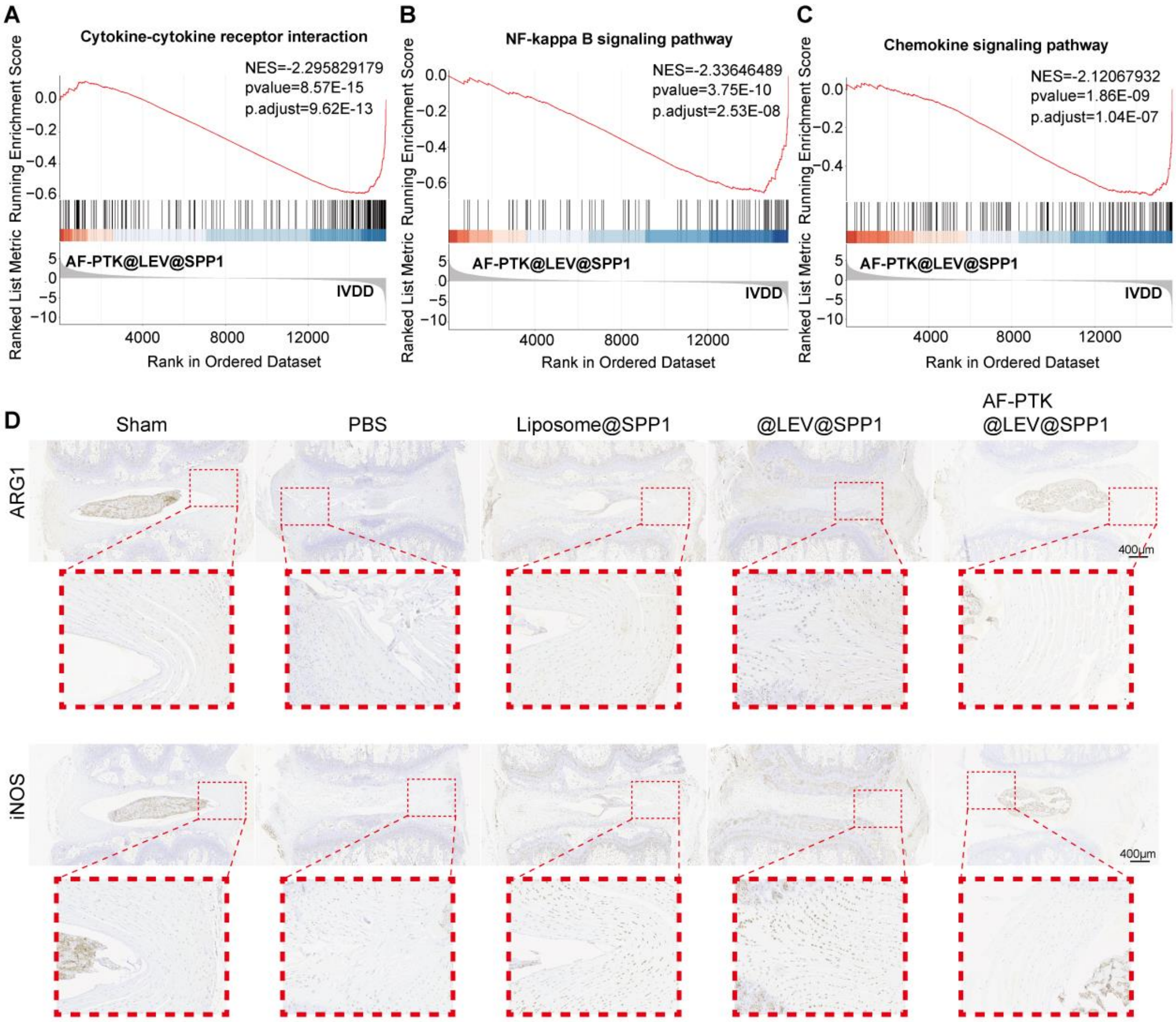

Supplementary Fig. 9.AF-PTK@LEV@SPP1 alleviates intervertebral disc degeneration by suppressing inflammatory signaling and reprogramming macrophage polarization.

(A-C) GSEA plots showing negative enrichment (NES < 0) of Cytokine–cytokine receptor, NF-κB, and Chemokine signaling pathways in AF-PTK@LEV@SPP1-treated discs, consistent with global suppression of inflammatory transcriptional programs.

(D)Immunohistochemical staining of ARG1 (M2 macrophage marker) and iNOS (M1 marker) in intervertebral discs from different treatment groups (Sham, PBS, Liposome@SPP1, @LEV@SPP1, AF-PTK@LEV@SPP1). Enhanced ARG1 and reduced iNOS expression in AF-PTK@LEV@SPP1-treated tissues confirm macrophage polarization toward an anti-inflammatory M2 phenotype.

References

1.Vogel, C. & Marcotte, E.M. Insights into the regulation of protein abundance from proteomic and transcriptomic analyses. Nat. Rev. Genet. 13, 227–232 (2012). Nature

2.Liu, Y., Beyer, A. & Aebersold, R. On the dependency of cellular protein levels on mRNA abundance. Cell 165, 535–550 (2016). Cell.

3.Edfors, F. et al. Gene-specific correlation of RNA and protein levels in human cells and tissues. Mol. Syst. Biol. 12, 883 (2016). Embo Press

4.Kosti, I., Jain, N., Aran, D., Butte, A.J. & Sirota, M. Cross-tissue analysis of gene and protein expression in normal and cancer tissues. Sci. Rep. 6, 24799 (2016). Nature

5.Gry, M. et al. Correlations between RNA and protein expression profiles in 23 human cell lines. BMC Genomics 10, 365 (2009).

7.There is concern about the reproducibility of the results, as the in vivo treatment experiments were conducted with only n = 3 per group. The authors stated that "sample sizes were determined based on prior power analysis and previously published studies of similar design". However, such a small sample size is insufficient to draw robust conclusions. Increasing the number of biological replicates is necessary to ensure statistical reliability, particularly for in vivo experiments (Figure 7).

RE: We sincerely appreciate the reviewer's valuable comment regarding the reproducibility and statistical reliability of our in vivo experiments. We completely agree that ensuring adequate biological replication is essential for scientific rigor. The relatively small sample size (n = 3 per group) used in our animal experiments was not intended to limit reproducibility but rather reflected a deliberate balance between ethical considerations, statistical feasibility, and experimental consistency in accordance with the internationally recognized 3Rs principle (Replacement, Reduction, and Refinement). In our study, each biological replicate was an independent animal, and all experiments were repeated three times with consistent outcomes. Data variability across replicates was minimal, and the observed differences between control and treated groups were statistically significant and aligned with our in vitro mechanistic results (see Fig. 6–7). All procedures—including randomization, blinding, and identical reagent batches—were carefully standardized to ensure reproducibility.

Notably, our chosen sample size aligns with commonly accepted practice in the intervertebral disc (IVD) and biomaterials fields, particularly for exploratory or proof-of-concept studies aimed at establishing mechanistic evidence. Multiple peer-reviewed studies have adopted similar sample sizes (n = 3 per group) for in vivo validation of biomaterials or disc-targeted therapeutics. For example, Mohd Isa et al. (1) examined hydrogel implantation effects with three animals per group; Chen et al. (2) and Yang et al. (3) employed n = 3 for in vivo testing of nucleic acid‑based and hydrogel-based anti-inflammatory strategies; Zhao et al. (4) used an equivalent sample size in studying mitochondrial ROS/NF-κB signaling in disc degeneration; and Zhang et al. (5)—from our own laboratory—established the exosome-hydrogel platform that forms the mechanistic basis for the present study. These precedents indicate that the sample size used here is consistent with field standards for initial mechanistic verification.

We sincerely appreciate the reviewer's thoughtful comments, which have enabled us to improve the transparency of our methodology. We firmly believe that, given the strong internal consistency of our data and their concordance with both previously published studies and our group's prior foundational work, the conclusions drawn from the present study are reliable, reproducible, and ethically justified. We are

grateful for the reviewer’s suggestion, and we will expand the sample size and incorporate multiple time points in future studies to further enhance statistical power. Once again, we sincerely thank the reviewer for this valuable feedback.

References

1.Mohd Isa I.L. et al. Implantation of hyaluronic acid hydrogel prevents the pain phenotype in a rat model of intervertebral disc injury. Sci Adv. 4, eaaq0597 (2018). PMID: 29632893.

2.Chen J. et al. Injectable self-healing hydrogel with siRNA delivery property for sustained STING silencing and enhanced therapy of intervertebral disc degeneration. Bioact. Mater. 9, 29–43 (2022). PMID: 34376261.

3.Yang L. et al. Dual-dynamic-bond cross-linked injectable hydrogel of multifunction for intervertebral disc degeneration therapy. J Nanobiotechnol. 20, 433 (2022). PMID: 36182921.

4.Zhao Y. et al. Cortistatin protects against intervertebral disc degeneration through targeting mitochondrial ROS-dependent NLRP3 inflammasome activation. Theranostics 10, 7015–7033 (2020). PMID: 32550919.

5.Zhang Y. et al. Injectable exosome-functionalized extracellular matrix hydrogel for intervertebral disc degeneration. J Nanobiotechnol. 19, 264 (2021). PMID: 34488795.

REVIEWER COMMENTS

Reviewer #2 (Remarks to the Author):

No additional comments. This is a well-developed study.
RE:We sincerely thank the reviewer for the positive evaluation of our work and for recognizing the quality and completeness of the study. We greatly appreciate the time and effort invested in reviewing our manuscript.

Reviewer #4 (Remarks to the Author):

The authors have made substantial efforts to revise the manuscript and have addressed most of my previous concerns, including clarifying the macrophage polarization markers, refining the bulk RNA-seq results, and improving the figures, introduction, and methods. However, I still have major concerns regarding the reproducibility of these in vivo findings. The use of only three samples per group is insufficient to support the main conclusions of the study.

The authors state that “each biological replicate was an independent animal, and all experiments were repeated three times with consistent outcomes,” which indicates that the in vivo experiments were repeated. If this is the case, the corresponding datasets from these repeated experiments should be included in the Supplementary Information to substantiate the reproducibility of the findings. Although some publications use three animals per group, providing additional datasets would greatly strengthen the reliability of the results and prevent the study’s conclusions from relying on a single in vivo experiment.

If increasing the number of biological replicates is not feasible, the authors should clearly indicate whether the presented data are representative of one of the repeated experiments and provide the corresponding data as Supplementary Information. Alternatively, they should explicitly acknowledge this limitation, although doing so may affect the manuscript’s suitability for publication in the Nature Communications.

I also recommend that the authors specify the number of experimental repeats throughout the manuscript and clarify whether the figures reflect representative or combined data.

RE:We sincerely thank you for the careful evaluation of our revised manuscript and for highlighting the critical issue of in vivo reproducibility. We fully agree that reproducibility is essential for supporting the main conclusions of this study, and we appreciate the opportunity to clarify our experimental design and further strengthen the manuscript.
We would like to clarify that the statistical analyses presented in the main figures are based on a single in vivo experimental cohort, where each group consisted of three independent animals (n = 3). Each animal represents a biological replicate, and no data from different animals or experiments were selectively excluded or pooled.
As described in the manuscript, all in vivo experiments were independently repeated three times using separate animal cohorts, and consistent results were obtained across these independent repeats. All experiments were conducted independently, and no

statistical analysis was performed by pooling data from different repeats, as combining independent experimental repeats could introduce batch effects.

In response to the reviewer's valuable suggestion, we have now included representative imaging and histological data from the remaining two independent experimental repeats in the Supplementary Information (Supplementary Fig. 11). For each repeat, a complete set of imaging and histological data is provided, including X-ray imaging, MRI, micro-CT, H&E staining, Safranin O staining, and immunohistochemical staining for COL II and MMP13. These data demonstrate the reproducibility and robustness of the in vivo phenotypes observed across independent animal cohorts. The consistent pathological and therapeutic trends observed in these independent repeats further support the reliability of the conclusions drawn from the primary dataset.

Additionally, we have revised all figure legends and the Methods section to clearly specify the number of biological replicates, the basis of statistical analyses, and whether the provided data represent representative images or quantified results. We believe that these revisions, along with the inclusion of data from independent repeats, directly address the reviewer's concerns and significantly enhance the rigor and transparency of the study.

We sincerely appreciate the reviewer for pointing out this important issue, which has greatly improved the rigor and transparency of our research. We will continue to place strong emphasis on reproducibility in our future work. Furthermore, if the reviewer considers that an increased number of biological replicates is still necessary, we would be more than willing to repeat the animal experiments with an expanded sample size to ensure the scientific rigor and reproducibility of our conclusions.

Once again, we sincerely thank you for your time and effort in providing such constructive feedback, which has significantly enhanced the rigor and clarity of our study.

Supplementary Fig. 11. Representative imaging and histological results from two additional independent in vivo experimental repeats (repeat 2 and repeat 3) corresponding to Fig. 7.

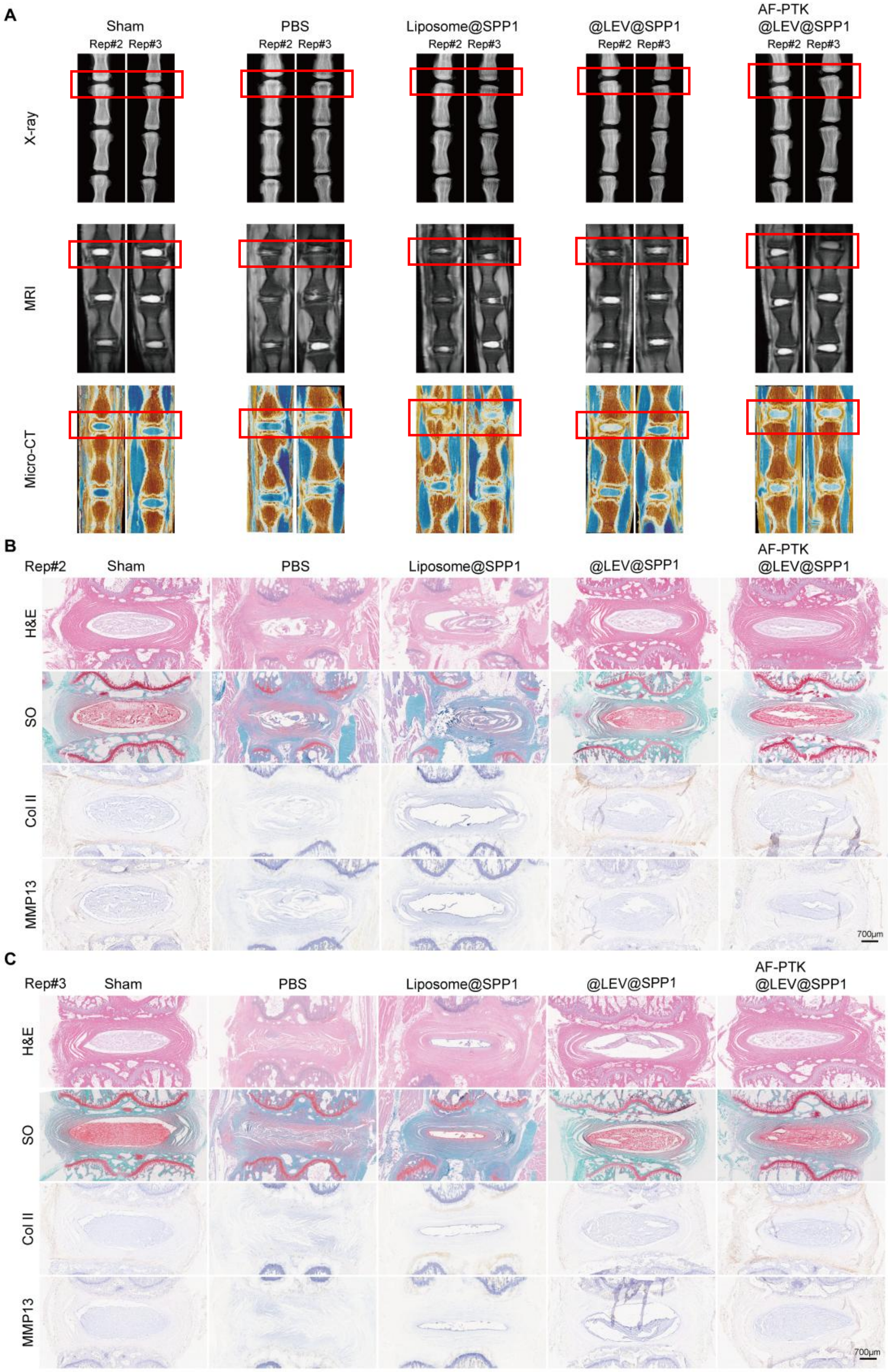

(A)Representative X-ray, MRI, and micro-CT images of intervertebral discs from two independent repeated animal experiments (repeat 2 and repeat 3) across different

treatment groups at 8 weeks post-treatment, including Sham, PBS, Liposome@SPP1, @LEV@SPP1, and AF-PTK@LEV@SPP1.
(B)Representative H&E, Safranin O, and immunohistochemical staining of intervertebral disc sections at 8 weeks from repeat 2, corresponding to the treatment groups shown in Fig. 7.
(C)Representative H&E, Safranin O, and immunohistochemical staining of intervertebral disc sections at 8 weeks from repeat 3, corresponding to the treatment groups shown in Fig. 7.These repeats are provided to demonstrate reproducibility and were not pooled with the primary dataset for statistical analysis.

Reviewer #5 (Remarks to the Author):
I co-reviewed this manuscript with one of the reviewers who provided the listed reports. This is part of the Nature Communications initiative to facilitate training in peer review and to provide appropriate recognition for Early Career Researchers who co-review manuscripts.
RE:We sincerely thank the reviewer for your time and contribution to the peer-review process. We appreciate Nature Communications ’ initiative to support the training and recognition of Early Career Researchers in peer review.We attach great importance to the opinions of reviewer #4 and have made corresponding modifications.

REVIEWER COMMENTS

Reviewer #4 (Remarks to the Author):

Although the authors have adequately addressed many of the concerns, I believe that an increased number of biological replicates remains necessary. This study could be considered for acceptance if the authors provide additional data with an adequate sample size.

RE:We sincerely thank the reviewer for emphasizing the importance of biological replication in the in vivo experiments. We fully agree that an adequate sample size is essential for ensuring the robustness and reproducibility of the conclusions. We also greatly appreciate this constructive suggestion.

Importantly, after receiving the reviewer's previous comments regarding biological replication, we immediately initiated additional in vivo experiments to increase the number of animals and strengthen the statistical robustness of the study.

In the revised manuscript, the in vivo experiments have been repeated with an expanded sample size. Specifically, the number of animals in each experimental group has been increased from three to six independent rats (n = 6). All key in vivo analyses—including X-ray imaging, MRI, micro-CT, histological staining (H&E and Safranin O/Fast Green), and immunohistochemical analyses of Col II, MMP13,ARG1 and iNOS —have been repeated using the expanded cohort.

The updated experimental results and corresponding quantitative analyses are now presented in the revised Figure 7, where statistical analyses are based on six independent animals per group. The expanded dataset reproduces the same therapeutic trends observed in the original experiments: AF-PTK@LEV@SPP1 significantly preserves disc height, reduces Pfirrmann degeneration scores, restores extracellular matrix homeostasis (increased COL II expression), and suppresses matrix degradation (reduced MMP13 expression).This consistency across the expanded sample further reinforces the reliability of our findings, ensuring that the therapeutic effects of AF-PTK@LEV@SPP1 in mitigating intervertebral disc degeneration are both robust and reproducible.

These additional biological replicates further confirm the robustness and reproducibility of the therapeutic effects of AF-PTK@LEV@SPP1 in mitigating intervertebral disc degeneration. We sincerely appreciate the reviewer for this valuable suggestion, which has significantly improved the rigor and statistical strength of our study.We are grateful for the opportunity to enhance the validity of our conclusions and further demonstrate the potential of this therapeutic strategy.

We have revised the description of our results in Page34 as follow:

"To evaluate the therapeutic efficacy of AF-PTK@LEV@SPP1 in mitigating intervertebral disc (IVD) degeneration, we established a rat model of acupuncture-induced disc injury and administered nanoparticle treatment over an 8-week period (Figure 7A). Six independent rats were included in each experimental group to ensure adequate biological replication.

Radiographic analysis revealed pronounced disc collapse and narrowing in PBS-treated animals compared with the sham group, confirming successful induction of disc degeneration. In contrast, rats treated with AF-PTK@LEV@SPP1 exhibited marked preservation of disc height and disc morphology (Fig. 7B). Quantitative analysis of the disc height index (DHI) showed a significant reduction in the PBS group (DHI ~50) compared with sham controls (DHI ~100). In contrast, AF-PTK@LEV@SPP1 treatment significantly restored the disc height (DHI ~90), demonstrating its therapeutic efficacy in preventing disc degeneration (Figure 7C). Although Liposome@SPP1 and @LEV@SPP1 showed modest improvements, the AF-PTK@LEV@SPP1 group displayed the most pronounced recovery of disc height among all treatment groups.

MRI imaging further corroborated these findings. Degenerated discs in PBS-treated rats exhibited markedly reduced nucleus pulposus signal intensity, indicating severe dehydration and structural deterioration. By contrast, discs treated with AF-PTK@LEV@SPP1 retained higher MRI signal intensity, suggesting improved hydration and tissue integrity (Fig. 7D). Consistent with the radiographic and MRI data, Pfirrmann grading scores were significantly higher in PBS-treated discs (grade 5), indicating severe degeneration. In contrast, AF-PTK@LEV@SPP1 treatment resulted in significantly lower degeneration scores (grade 2-3), demonstrating effective protection of disc structure (Figure 7E). Liposome@SPP1 and @LEV@SPP1 treatments produced partial improvements but remained substantially inferior to AF-PTK@LEV@SPP1.High-resolution micro-CT imaging further revealed severe osteophyte formation and endplate remodeling in PBS-treated discs. In contrast, AF-PTK@LEV@SPP1 treatment preserved vertebral endplate architecture and markedly reduced osteophyte formation (Fig. 7F), indicating improved structural stability of the intervertebral disc.

Histological examination provided additional evidence of therapeutic efficacy. H&E and Safranin O/Fast Green staining demonstrated severe disruption of the annulus fibrosus – nucleus pulposus (AF – NP) interface and loss of proteoglycan-rich extracellular matrix in PBS-treated discs. In contrast, discs treated with AF-PTK@LEV@SPP1 retained a well-organized AF – NP structure and abundant proteoglycan deposition, closely resembling the morphology observed in the sham group (Fig. 7G).Immunohistochemical analysis further confirmed the restoration of extracellular matrix homeostasis. The percentage of COL II – positive cells, a marker of matrix synthesis, was markedly reduced in PBS-treated discs but significantly increased following AF-PTK@LEV@SPP1 treatment. Conversely, the catabolic marker MMP13 was highly expressed in degenerative discs but substantially decreased in the AF-PTK@LEV@SPP1 group. Compared with Liposome@SPP1 and @LEV@SPP1 treatments, AF-PTK@LEV@SPP1 achieved the most pronounced restoration of anabolic – catabolic balance within the disc matrix(Figures 7H – K).Together, these in vivo imaging, histological, and molecular analyses demonstrate that AF-PTK@LEV@SPP1 effectively attenuates intervertebral disc degeneration, preserves disc structure, and promotes extracellular matrix regeneration.”

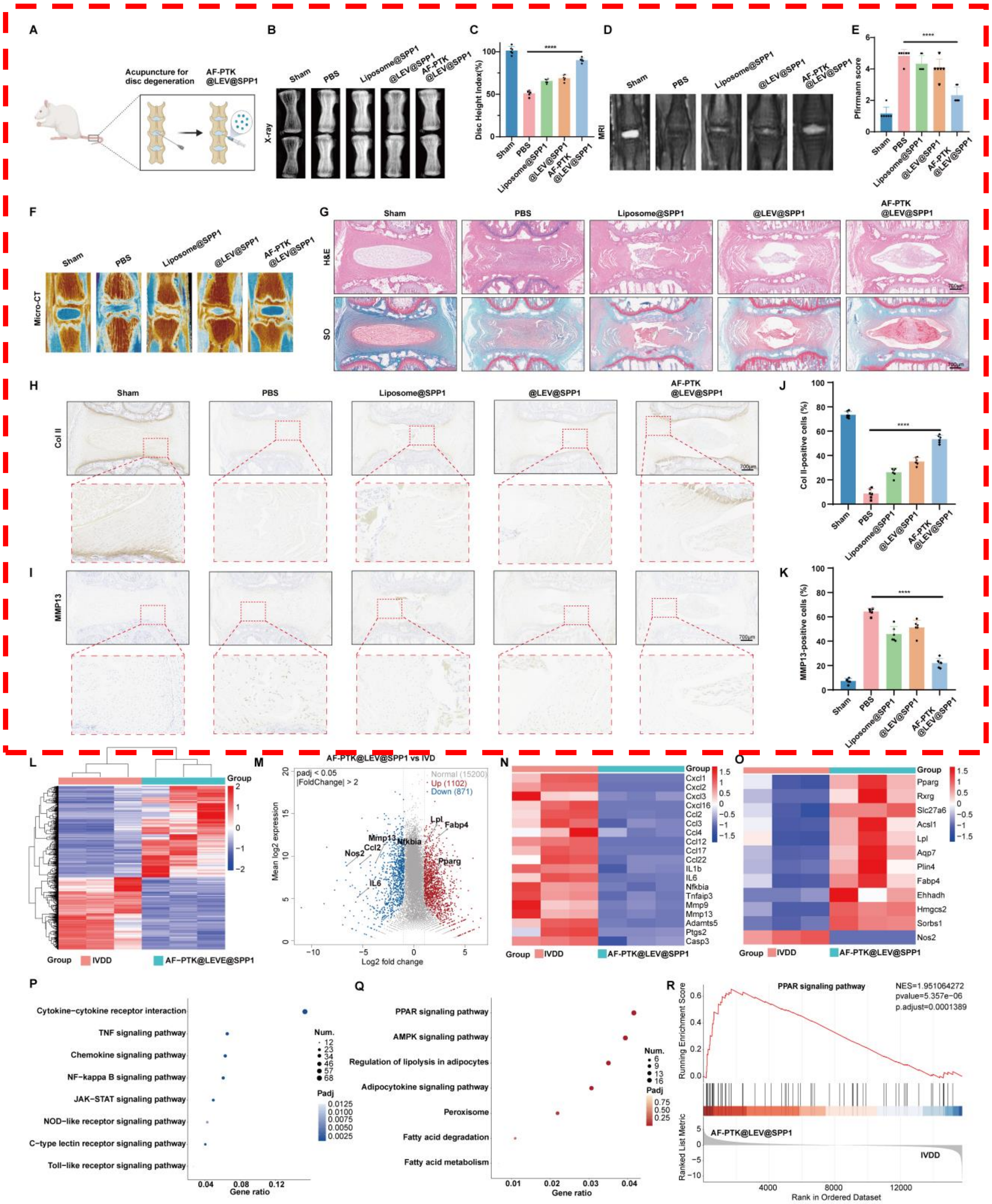

Figure 7. AF-PTK@LEV@SPP1 alleviates intervertebral disc degeneration by reducing inflammation and modulating lipid metabolism.

(A)Schematic illustration of AF-PTK@LEV@SPP1 treatment for acupuncture-induced intervertebral disc degeneration.

(B)Representative X-ray images of intervertebral discs across different treatment groups after 8 weeks (Sham, PBS, Liposome@SPP1, @LEV@SPP1, AF-PTK@LEV@SPP1).

(C)Quantification of disc height index from X-ray data in different treatment groups.

(D)Representative MRI images of intervertebral discs in different treatment groups.

(E)Quantification of Pfirrmann scores from different treatment groups indicating degeneration severity.
(F)Micro-CT images showing bone architecture changes in intervertebral discs across treatment groups.
(G)H&E and Safranin O/Fast Green staining of intervertebral discs at 8 weeks in different treatment groups.
(H, I) Representative immunohistochemical staining of Col II and MMP13 in intervertebral discs from different treatment groups.
(J, K) Quantification of Col II-positive and MMP13-positive cells in intervertebral discs.
(L) Heatmap showing global mRNA expression profiles of rat intervertebral discs following acupuncture-induced degeneration and subsequent treatment with PBS (IVDD) or AF-PTK@LEV@SPP1. Distinct clustering demonstrates transcriptomic divergence between groups.
(M) Volcano plot illustrating significantly upregulated (red) and downregulated (blue) genes in AF-PTK@LEV@SPP1-treated discs compared with the IVDD group ($|\log_2 FC| > 2$, $p < 0.05$). Key genes involved in inflammation (Nos2, Ccl2, Il6, Mmp13) and lipid metabolism (Pparg, Fabp4, Lpl, Nfkbia) are highlighted.
(N) Heatmap of inflammation- and extracellular matrix (ECM)-related genes (Cxcl1, Cxcl2, Cxcl3, Cxcl16, Ccl2, Ccl3, Ccl4, Ccl12, Ccl17, Ccl22, Il1b, Il6, Nfkbia, Tnfaip3, Mmp9, Mmp13, Adamts5, Ptgs2, Casp3), showing broad downregulation in AF-PTK@LEV@SPP1-treated discs compared to IVDD controls.
(O) Heatmap of lipid metabolism and PPAR signaling associated genes (Pparg, Fabp4, Lpl, Plin4, Acsl1, Aqp7, Hmgcs2, Sorbs1, Rxrg, Slc27a6, Ehhadh), which were upregulated in AF-PTK@LEV@SPP1-treated discs, whereas the M1 marker Nos2 was reduced, indicating a shift toward anti-inflammatory and metabolic homeostasis.
(P) KEGG enrichment analysis of downregulated genes highlighting suppressed inflammatory pathways, including Cytokine – cytokine receptor interaction, NF-κB signaling, TNF signaling, Chemokine signaling, JAK – STAT, Toll-like receptor, NOD-like receptor, and C-type lectin receptor signaling.
(Q) KEGG enrichment analysis of upregulated genes identifying activation of PPAR signaling, AMPK signaling, adipocytokine signaling, regulation of lipolysis in adipocytes, fatty acid metabolism and degradation, and peroxisome pathways.
(R)Gene set enrichment analysis (GSEA) showing positive enrichment of the PPAR signaling pathway (NES = 1.95, $p = 5.36 \times 10^{-6}$, $q = 1.4 \times 10^{-3}$) in the AF-PTK@LEV@SPP1 group compared to IVDD controls, confirming activation of lipid-metabolic and anti-inflammatory signaling.
Representative images are shown. Quantitative analyses were performed using six independent animals per group (n = 6) unless otherwise indicated. Data are presented as mean ± SD. Statistical significance was determined using one-way ANOVA followed by appropriate post hoc tests. Bulk RNA-seq analysis was performed using intervertebral disc tissues from three independent animals per group (n = 3)

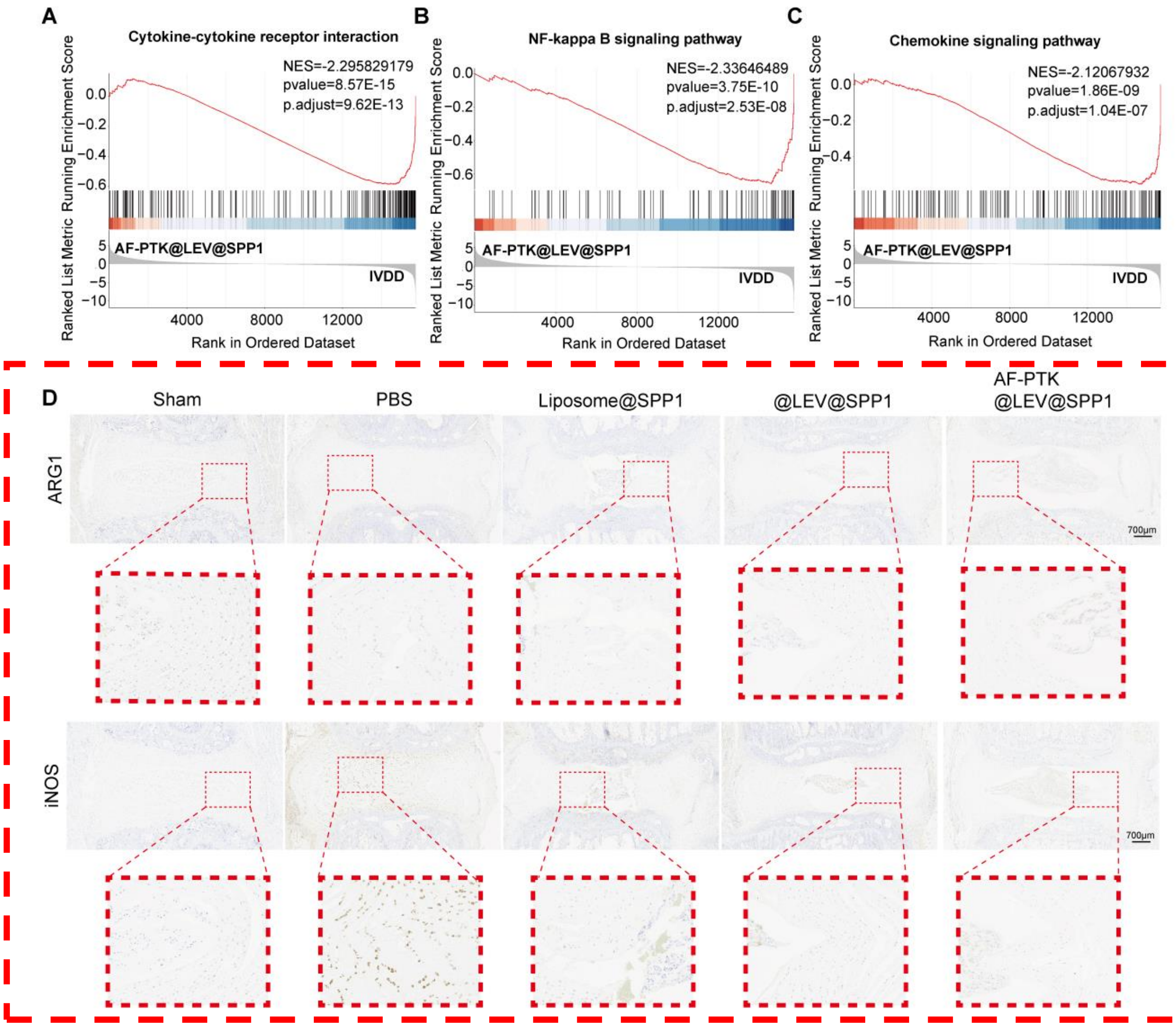

Supplementary Fig. 9. AF-PTK@LEV@SPP1 alleviates intervertebral disc degeneration by suppressing inflammatory signaling and reprogramming macrophage polarization.

(A-C) GSEA plots showing negative enrichment (NES < 0) of Cytokine–cytokine receptor, NF-κB, and Chemokine signaling pathways in AF-PTK@LEV@SPP1-treated discs, consistent with global suppression of inflammatory transcriptional programs.

(D) Immunohistochemical staining of ARG1 (M2 macrophage marker) and iNOS (M1 marker) in intervertebral discs from different treatment groups (Sham, PBS, Liposome@SPP1, @LEV@SPP1, AF-PTK@LEV@SPP1). Enhanced ARG1 and reduced iNOS expression in AF-PTK@LEV@SPP1-treated tissues confirm macrophage polarization toward an anti-inflammatory M2 phenotype(n=6).

The manuscript by Li et al. reports on the effects of OMV-liposomes targeting macrophage polarization in disc degeneration. The authors developed siRNA (Spp1)-loaded liposomes incorporating bacterial extracellular vehicles (EVs) to enhance macrophage uptake. An evaluation was made of whether the points raised by the reviewer #3 had been satisfactorily addressed. The authors have made efforts to revise their manuscript; however, some concerns remain that need to be addressed.

1. Significant revision of the Introduction section is necessary, as it is too lengthy and insufficiently focused.
2. The methods and experimental procedures were strengthened through the inclusion of additional details provided on compound dosage and administration, as well as on the binding peptide.
3. Regarding the rationale of the study, how did the authors select SPP1 as the target and determine its correlation with macrophage polarization? SPP1 is well known to promote macrophage polarization toward the M2 phenotype. Silencing SPP1 in M2 macrophages reverts them to an M1-like phenotype and inhibits tumor progression. Therefore, stronger supporting data are needed to link the authors' findings to macrophage polarization. Although several studies have reported that SPP1 promotes M2 polarization in cancer, pulmonary fibrosis, and related diseases, relatively few have associated SPP1 with inflammatory (M1-like) macrophages. This discrepancy should be addressed experimentally and thoroughly discussed in the manuscript.
4. There is concern about the polarization assay. A macrophage polarization assay in the presence or absence of recombinant SPP1 is recommended, using LPS and IFN-γ to induce M1 polarization and IL-4 to induce M2 polarization. Alternatively, the constructed liposome could be used in these assays. In addition, Western blot analysis of transcription factors and M2-specific markers should be performed in parallel. The current data, showing only CD68 and iNOS expression, are insufficient to support conclusions regarding macrophage polarization.
5. The authors highlighted the distribution of macrophages in the IVD, and the figure has been improved by adding of arrows.
6. The bulk RNA-seq data presented in Figure 7 require further clarification. The presented results are confusing and inconsistent with the authors' statements. While the authors report that *Arg1* and *Timp1* are upregulated in the treated group, the figure clearly shows that they are downregulated. Only *PPARγ* appears to be slightly upregulated. Based on these data, silencing *SPP1*

seems to enhance inflammation, as NFKB and RA and Il-17 signaling pathways are among the most significantly enriched in the pathway analysis. For the GSEA analysis, please provide the exact *p*-values and clearly indicate the positions of the control and treated groups with the enrichment plots. Furthermore, the GSEA results are inconsistent with the pathway enrichment analysis, which showed upregulation of inflammation-related terms. This discrepancy should be carefully addressed, and the corresponding statements revised accordingly. In addition, the quality of the figure should be improved. The RNA-seq findings need to be supported by immunohistochemistry (IHC), particularly with data demonstrating polarized macrophages in treated mice, which is essential for supporting the study's conclusions. Finally, a heatmap of genes enriched in the top pathways should be provided to enhance clarity and transparency.

7. There is concern about the reproducibility of the results, as the *in vivo* treatment experiments were conducted with only n = 3 per group. The authors stated that "sample sizes were determined based on prior power analysis and previously published studies of similar design". However, such a small sample size is insufficient to draw robust conclusions. Increasing the number of biological replicates is necessary to ensure statistical reliability, particulaly for *in vivo* experiments (Figure 7).
