# Supplementary material for: An annulus fibrosus anchored nanovector reprograms osteopontin driven macrophages in preclinical disc degeneration models
Source: Nat Commun. 2026 Jul 21;17:8887. doi: 10.1038/s41467-026-75720-3 (PMC13500848; doi:10.1038/s41467-026-75720-3)
Supplement: Supplementary file 3 [file 41467_2026_75720_MOESM3_ESM.pdf]

nature portfolio

Corresponding author(s): Ning Zhang

Last updated by author(s): Jun 23, 2026

Reporting Summary

Nature Portfolio wishes to improve the reproducibility of the work that we publish. This form provides structure for consistency and transparency in reporting. For further information on Nature Portfolio policies, see our Editorial Policies and the Editorial Policy Checklist.

Statistics

For all statistical analyses, confirm that the following items are present in the figure legend, table legend, main text, or Methods section.

n/a	Confirmed	
☐	☒	The exact sample size (*n*) for each experimental group/condition, given as a discrete number and unit of measurement
☐	☒	A statement on whether measurements were taken from distinct samples or whether the same sample was measured repeatedly
☐	☒	The statistical test(s) used AND whether they are one- or two-sided<br>*Only common tests should be described solely by name; describe more complex techniques in the Methods section.*
☒	☐	A description of all covariates tested
☐	☒	A description of any assumptions or corrections, such as tests of normality and adjustment for multiple comparisons
☐	☒	A full description of the statistical parameters including central tendency (e.g. means) or other basic estimates (e.g. regression coefficient) AND variation (e.g. standard deviation) or associated estimates of uncertainty (e.g. confidence intervals)
☐	☒	For null hypothesis testing, the test statistic (e.g. *F*, *t*, *r*) with confidence intervals, effect sizes, degrees of freedom and *P* value noted<br>*Give P values as exact values whenever suitable.*
☒	☐	For Bayesian analysis, information on the choice of priors and Markov chain Monte Carlo settings
☒	☐	For hierarchical and complex designs, identification of the appropriate level for tests and full reporting of outcomes
☒	☐	Estimates of effect sizes (e.g. Cohen's *d*, Pearson's *r*), indicating how they were calculated

Our web collection on statistics for biologists contains articles on many of the points above.

Software and code

Policy information about availability of computer code

Data collection	Data were collected using standard laboratory equipment. No commercial or custom software was used during the data collection process.
Data analysis	Data analysis was performed using standard commercial and open-source software, including GraphPad Prism 10.0, ImageJ (Fiji), FlowJo v10, Seurat (v4.0, R), Cell Ranger, and GSEA/KEGG tools, with no custom code employed.

For manuscripts utilizing custom algorithms or software that are central to the research but not yet described in published literature, software must be made available to editors and reviewers. We strongly encourage code deposition in a community repository (e.g. GitHub). See the Nature Portfolio guidelines for submitting code & software for further information.

Data

Policy information about availability of data

All manuscripts must include a data availability statement. This statement should provide the following information, where applicable:

- Accession codes, unique identifiers, or web links for publicly available datasets
- A description of any restrictions on data availability
- For clinical datasets or third party data, please ensure that the statement adheres to our policy

The original contributions presented in this study are included in the article and its Supplementary Material. Further inquiries can be directed to the corresponding authors. The raw single-cell RNA sequencing data generated in this study have been deposited in the Genome Sequence Archive for Human (GSA-Human) under accession codes HRA010009 and HRA018950. The processed single-cell RNA-seq expression matrix files have been deposited in OMIX under accession code

OMIX017533. The bulk RNA sequencing data generated in this study have been deposited in the NCBI Gene Expression Omnibus (GEO) under accession code GSE289741. These datasets are publicly available. Source data are provided with this paper.

Research involving human participants, their data, or biological material

Policy information about studies with human participants or human data. See also policy information about sex, gender (identity/presentation), and sexual orientation and race, ethnicity and racism.

Reporting on sex and gender	Sex was determined based on self-report during patient enrollment. All participants were biologically male or female and this information was recorded. However, sex-based analyses were not performed in this study, as the primary focus was on immune microenvironment profiling within degenerated intervertebral discs rather than sex-based differences.
Reporting on race, ethnicity, or other socially relevant groupings	Race and ethnicity data were not collected or used in the analysis. The study focused on molecular and cellular mechanisms of intervertebral disc degeneration and did not include socially constructed demographic groupings in the study design.
Population characteristics	Human samples were obtained from five patients aged 65–80 years who underwent surgery for intervertebral disc degeneration. All participants were clinically diagnosed with lumbar disc degeneration and provided written informed consent prior to sample collection. No behavioral, psychiatric, or genetic subgrouping was involved.
Recruitment	Participants were recruited from the Second Affiliated Hospital of Zhejiang University School of Medicine based on clinical diagnosis and eligibility for surgical intervention for intervertebral disc degeneration. All participants gave written informed consent. There was no targeted selection based on sex, ethnicity, or socioeconomic status.
Ethics oversight	The study protocol was approved by the Institutional Review Board of the Second Affiliated Hospital of Zhejiang University (approval number: 2024SQXB002446). All procedures were conducted in accordance with the Declaration of Helsinki.

Note that full information on the approval of the study protocol must also be provided in the manuscript.

Field-specific reporting

Please select the one below that is the best fit for your research. If you are not sure, read the appropriate sections before making your selection.

☒ Life sciences ☐ Behavioural & social sciences ☐ Ecological, evolutionary & environmental sciences

For a reference copy of the document with all sections, see nature.com/documents/nr-reporting-summary-flat.pdf

Life sciences study design

All studies must disclose on these points even when the disclosure is negative.

Sample size	Sample sizes were based on previous studies in the field of intervertebral disc degeneration and nanomedicine. No formal sample size calculation was performed. The number of biological replicates (n ≥ 3 for cell experiments; n ≥ 3 for animal studies) was chosen to ensure reproducibility and statistical validity.No formal statistical methods were used to predetermine sample sizes. Sample sizes were selected based on commonly used practices in the field and our previous experience with similar experimental models, ensuring sufficient statistical power to detect biologically relevant differences. The chosen sample sizes are consistent with those reported in previous studies using comparable approaches.
Data exclusions	No data were excluded from the analyses. All samples and data points collected were included in the final analysis. Pre-established criteria were not applied for exclusion.
Replication	All key experiments were independently repeated at least three times with consistent results. Animal studies included multiple biological replicates across treatment groups to ensure reproducibility.
Randomization	Animals were randomly assigned to experimental groups using a simple randomization method. Randomization was applied to reduce potential allocation bias in treatment administration and outcome assessment.
Blinding	Investigators were not blinded during data collection for most experiments due to the nature of the procedures. However, data analysis was performed using objective and, where possible, blinded quantification.

Reporting for specific materials, systems and methods

We require information from authors about some types of materials, experimental systems and methods used in many studies. Here, indicate whether each material, system or method listed is relevant to your study. If you are not sure if a list item applies to your research, read the appropriate section before selecting a response.

Materials & experimental systems

n/a	Involved in the study	
☐	☒	Antibodies
☒	☐	Eukaryotic cell lines
☒	☐	Palaeontology and archaeology
☐	☒	Animals and other organisms
☒	☐	Clinical data
☒	☐	Dual use research of concern
☒	☐	Plants

Methods

n/a	Involved in the study	
☒	☐	ChIP-seq
☐	☒	Flow cytometry
☒	☐	MRI-based neuroimaging

Antibodies

Antibodies used

The following primary antibodies were used in Western blot:
Aggrecan(ABclonal,A8536,1:500),MMP13(abcam,ab39012,1:2000),ADAMTS5(ABclonal,A23125,1:2000),Collagen II(Proteintech,28459-1-AP,1:500),SOX2(ABclonal,A0561,1:500),OCT4(ABclonal,A25315,1:1000),c-MYC(ABclonal,A19032,1:1000),STAT1(Affinity,AF6300,1:500),STAT6(Affinity,AF6302,1:500),Phospho-STAT1(Affinity,AF3300,1:500),Phospho-STAT6(Affinity,AF3301,1:500),ARG1(Affinity,DF6657,1:500),iNOS(Affinity,AF0199,1:500),CD206(Affinity,DF4149,1:500),CD86(Affinity,DF6332,1:500),p65(Affinity,AF5006,1:500),Phospho-p65 (Affinity,AF3389,1:500),β-actin (Abcam,ab8227,1:1000)
Immunohistochemistry:
Col II (Abcam,ab307674,1:100) , MMP13 (Affinity,AF5355,1:100),ARG1(Affinity,DF6657,1:100),iNOS(Affinity,AF0199,1:100)

Validation

All antibodies used for Western blot were validated by the manufacturers for the target species and applications. Validation information is available from the Abcam ,Proteintech ,ABclonal and Affinity Biosciences websites. Experimental specificity was confirmed by expected molecular weight and consistent expression patterns.

Animals and other research organisms

Policy information about studies involving animals; ARRIVE guidelines recommended for reporting animal research, and Sex and Gender in Research

Laboratory animals

C57BL/6J mice (8 weeks old), Sprague-Dawley rats (male, 8-12 weeks old), and Lyz2-Cre;DTR mice (8-10 weeks old) were used in this study. C57BL/6J mice and Sprague-Dawley rats were purchased from Shanghai SLAC Laboratory Animal Co., Ltd. (Shanghai, China). Lyz2-Cre;DTR mice were generated in-house by crossing Lyz2-Cre transgenic mice with DTR-floxed mice.All animals were housed under specific pathogen-free conditions in a controlled environment (22 ± 1°C, 50 ± 10% humidity, 12 h light/dark cycle) with ad libitum access to standard laboratory chow and autoclaved water. The number of animals used in each experiment is indicated in the corresponding figure legends.Both male and female mice were included, whereas all rats were male.

Wild animals

This study did not involve wild animals.

Reporting on sex

Both male and female mice were included, whereas all rats were male. Sex was not considered as a biological variable, and no analyses were performed to assess sex differences. The number of animals used in each experiment is indicated in the corresponding figure legends.

Field-collected samples

This study did not involve samples collected from the field.

Ethics oversight

Animal experiments were approved by the Institutional Animal Care and Use Committee of the Second Affiliated Hospital of Zhejiang University School of Medicine (approval No. 2024-351).Human studies were approved by the Institutional Review Board of the Second Affiliated Hospital of Zhejiang University (approval No. 2024SQXB002446). Written informed consent was obtained from all participants.

Note that full information on the approval of the study protocol must also be provided in the manuscript.

Plants

Seed stocks

This study did not involve the use of any plant or seed stocks.

Novel plant genotypes

No novel plant genotypes were used or generated in this study.

Authentication

Not applicable. Plant material and associated authentication were not part of this study.

Flow Cytometry

Plots

Confirm that:

☒ The axis labels state the marker and fluorochrome used (e.g. CD4-FITC).

☒ The axis scales are clearly visible. Include numbers along axes only for bottom left plot of group (a 'group' is an analysis of identical markers).

☐ All plots are contour plots with outliers or pseudocolor plots.

☒ A numerical value for number of cells or percentage (with statistics) is provided.

Methodology

Sample preparation	Bone marrow–derived macrophages (BMDMs) were harvested from mice and washed with cold PBS. Fc receptors were blocked using anti-CD16/32 (TruStain FcX™, BioLegend, CAT# 101319) for 10 min at 4 °C. Live/dead discrimination was performed with Zombie Aqua viability dye. Surface staining with fluorophore-conjugated anti-CD86 was followed by fixation and permeabilization using True-Nuclear™ Buffer Set (BioLegend, CAT# 424401), and intracellular staining for iNOS.
Instrument	Beckman Coulter CytoFLEX LX flow cytometer
Software	Data were acquired using CytExpert v2.4 and analyzed with FlowJo v10.
Cell population abundance	$CD86^+iNOS^+$ double-positive macrophage populations were quantified as a percentage of total BMDMs. Representative plots and quantification results are shown in Figure 5.
Gating strategy	Initial gating was performed on FSC/SSC to exclude debris and select viable single cells. Zombie Aqua–negative cells were considered viable. CD86-positive populations were gated on surface staining, followed by gating of iNOS-positive cells within the $CD86^+$ population. Positive/negative boundaries were set based on fluorescence-minus-one (FMO) controls.

☒ Tick this box to confirm that a figure exemplifying the gating strategy is provided in the Supplementary Information.
